# *Plasmodium falciparum* Heterochromatin Protein 1 Marks Genomic Loci Linked to Phenotypic Variation of Exported Virulence Factors

**DOI:** 10.1371/journal.ppat.1000569

**Published:** 2009-09-04

**Authors:** Christian Flueck, Richard Bartfai, Jennifer Volz, Igor Niederwieser, Adriana M. Salcedo-Amaya, Blaise T. F. Alako, Florian Ehlgen, Stuart A. Ralph, Alan F. Cowman, Zbynek Bozdech, Hendrik G. Stunnenberg, Till S. Voss

**Affiliations:** 1 Department of Medical Parasitology and Infection Biology, Swiss Tropical Institute, Basle, Switzerland; 2 Department of Molecular Biology, Nijmegen Center of Molecular Life Sciences, Radboud University, Nijmegen, The Netherlands; 3 Division of Infection and Immunity, The Walter and Eliza Hall Institute of Medical Research, Melbourne, Australia; 4 Department of Biochemistry and Molecular Biology, Bio21 Molecular Science and Biotechnology Institute, The University of Melbourne, Victoria, Australia; 5 School of Biological Sciences, Nanyang Technological University, Singapore; Weill Medical College of Cornell University, United States of America

## Abstract

Epigenetic processes are the main conductors of phenotypic variation in eukaryotes. The malaria parasite *Plasmodium falciparum* employs antigenic variation of the major surface antigen PfEMP1, encoded by 60 *var* genes, to evade acquired immune responses. Antigenic variation of PfEMP1 occurs through *in situ* switches in mono-allelic *var* gene transcription, which is PfSIR2-dependent and associated with the presence of repressive H3K9me3 marks at silenced loci. Here, we show that *P. falciparum* heterochromatin protein 1 (PfHP1) binds specifically to H3K9me3 but not to other repressive histone methyl marks. Based on nuclear fractionation and detailed immuno-localization assays, PfHP1 constitutes a major component of heterochromatin in perinuclear chromosome end clusters. High-resolution genome-wide chromatin immuno-precipitation demonstrates the striking association of PfHP1 with virulence gene arrays in subtelomeric and chromosome-internal islands and a high correlation with previously mapped H3K9me3 marks. These include not only *var* genes, but also the majority of *P. falciparum* lineage-specific gene families coding for exported proteins involved in host–parasite interactions. In addition, we identified a number of PfHP1-bound genes that were not enriched in H3K9me3, many of which code for proteins expressed during invasion or at different life cycle stages. Interestingly, PfHP1 is absent from centromeric regions, implying important differences in centromere biology between *P. falciparum* and its human host. Over-expression of PfHP1 results in an enhancement of variegated expression and highlights the presence of well-defined heterochromatic boundaries. In summary, we identify PfHP1 as a major effector of virulence gene silencing and phenotypic variation. Our results are instrumental for our understanding of this widely used survival strategy in unicellular pathogens.

## Author Summary

*Plasmodium falciparum* causes the most severe form of malaria in humans. The high virulence of this unicellular parasite is in part related to the selective expression of members of falciparum-specific gene families. These genes encode proteins that are exported into the cytoplasm and onto the surface of infected red blood cells. To avoid recognition by the host's immune system, *P. falciparum* employs sequential expression of antigenically different variants of these surface proteins. While the epigenetic mechanisms responsible for such clonal expression have been studied in some detail for the major virulence gene family *var*, the regulation and function of other exported protein families remain elusive. Here, we identify *P. falciparum* heterochromatin protein 1 as a major structural component of virulence gene islands throughout the parasite genome. This factor binds specifically to a reversible histone modification, which marks these virulence loci for transcriptional silencing. Our observations suggest a unifying epigenetic strategy in the regulation of host–parasite interactions and immune evasion in *P. falciparum*. Furthermore, these findings have important implications for the future study of hitherto uncharacterized exported proteins with roles in parasite virulence.

## Introduction

*Plasmodium falciparum* causes the most severe form of malaria in humans with over one million deaths annually [1]. Severe and fatal outcomes of infections with this protozoan parasite result from a multitude of syndromes triggered by repeated rounds of asexual reproduction within erythrocytes. After invasion into red blood cells (RBCs), the parasite initiates a dramatic host cell remodeling process, culminating in the export of parasite virulence factors onto the surface of infected RBCs (iRBCs) [2]. The majority of these proteins is encoded by species-specific subtelomeric gene families, some of which underwent massive expansion during the evolution of the *P. falciparum* lineage [3]. One of the direct consequences of their concerted expression is the sequestration of iRBCs in the microvasculatory system, a process that is linked to severe complications including cerebral and placental malaria [4]–[6]. Sequestration occurs due to interactions of *P. falciparum* erythrocyte membrane protein 1 (PfEMP1) with various receptors on endothelial cells and uninfected erythrocytes [7]–[10]. 60 PfEMP1 variants are encoded by individual members of the *var* gene family [11]–[13]. Importantly, only one *var* gene is transcribed by a single parasite (mutual exclusion) [14]. Switches in *var* gene transcription occur *in situ* in absence of any apparent recombination events and result in antigenic variation of PfEMP1 [15]. This clonal phenotypic variation allows the parasite to evade variant-specific humoral immune responses and to sequester in various tissues [16]. Members of some other gene families (*rif*, *stevor*, *Pfmc-2tm*) are also expressed in a restrictive manner [17]–[19], however, their role in parasite biology and the underlying regulatory mechanisms remain unclear.

Multiple *var* genes are located in most subtelomeric regions directly downstream of telomere-associated repeat elements (TAREs) and in internal clusters on some chromosomes [20]. At either location *var* genes occur in close association with other variably expressed multi-gene families [13]. Several recent studies investigating the nature of the epigenetic mechanisms involved in the control of mono-allelic *var* gene transcription revealed an important role of the conserved promoter and intron sequences [21]. *var* promoters are silenced by default and, notably, activation of an episomal *var* promoters caused silencing of the entire repertoire of native *var* genes [22]–[24]. In addition to the 5′ upstream sequences the *var* gene intron is involved in silencing [25] and, although its exact role in this process remains controversial, this finding has been confirmed several times [26]–[28]. Fluorescent *in situ* hybridisation (FISH) experiments revealed that *P. falciparum* chromosome ends occur in perinuclear clusters [29],[30]. Consequently, subtelomeric *var* genes are inherently positioned at the nuclear periphery which is linked to enhanced transcriptional silencing in other eukaryotes [31]–[33]. Moreover, this spatial association was also demonstrated for chromosome-internal *var* genes [23],[34]. Transgenes inserted into subtelomeric repeat regions were transcriptionally silenced in a metastable fashion [35], similar to position-effect variegation in yeasts and higher eukaryotes [36]. The process of *var* gene activation thus appears to be linked to nuclear re-positioning of a silenced locus into a transcriptionally active zone lending support for the existence of a specialized perinuclear region dedicated to mutually exclusive *var* transcription [23],[35],[37]. The perinuclear location of *var* genes is clearly independent of their transcriptional state, however, conflicting results exist as to whether *var* gene activation occurs within or outside chromosome end clusters [23],[30],[34],[35],[37]. Together, this complex architectural setup provides a dynamic foundation for the heritable and variegated silencing of *var* genes and other variably expressed gene families by epigenetic control processes.

The current data is consistent with facultative heterochromatin-based silencing where dynamic alterations in local chromatin structure act as the major regulatory mechanism. Many of the reversible histone modifications characteristic for active or silenced chromatin in other eukaryotes have been described in *P. falciparum* [38]–[40]. Recent studies addressed their role in *var* gene regulation and uncovered the first indications for a distinct histone code linked to variegated *var* gene expression [41]. Active *var* genes are associated with histone 3 acetylation (H3K9ac) and methylation (H3K4me2, H3K4me3) and silenced *var* genes are enriched in H3K9me3 [39],[42]. The *P. falciparum* genome also encodes a core set of histone-modifying enzymes including histone deacetylases and methyltransferases (HMTs) [43],[44]. Interestingly, different subsets of *var* and *rif* genes show increased transcription in mutant parasite lines lacking either one of the two PfSIR2 paralogs, demonstrating a direct role for these histone deacetylases in virulence gene silencing [35],[45]. Recently, H3K9me3 has been mapped to the full set of *var* genes and to members of other subtelomeric gene families on a genome-wide scale [37],[46]. The presence of H3K9me3 at silenced *var* loci provides important clues about the possible control strategy underlying epigenetic *var* regulation. This particular histone modification is a conserved hallmark of heterochromatic silencing and serves as a docking site for heterochromatin protein 1 (HP1) to nucleate the propagation of heterochromatin along the chromosome fiber [47],[48]. Indeed, PfHP1, the *P. falciparum* ortholog of HP1, binds to H3K9me3 and was shown to be involved in variegated expression of a particular *var* gene [49]. Although these findings justify the assumption that PfHP1 may be associated with H3K9me3-enriched loci, the genome-wide localization of PfHP1 is unknown and cannot be inferred directly from these data; HP1 proteins interact with a multitude of other proteins [50], proteins other than HP1 bind to H3K9me3 [51], H3K9me3 is not necessarily sufficient to recruit HP1 [52],[53], and HP1 can be recruited to heterochromatin in an H3K9me3-independent manner [54],[55].

In this study, we provide a comprehensive analysis of PfHP1 on the level of protein function and genome-wide distribution. We show that PfHP1 binds specifically to H3K9me3 but not to other repressive histone methylation marks. Using nuclear fractionation and detailed immuno-localization experiments, we demonstrate that PfHP1 constitutes a major heterochromatin component with confined localization to perinuclear foci. High-resolution analysis by genome-wide chromatin immunoprecipitation (ChIP-on-chip) revealed a striking PfHP1 occupancy pattern restricted to 425 genes, most of which are members of *P. falciparum*-specific exported virulence families. The majority of these genes were also enriched in H3K9me3 underscoring the biological significance of this interaction in virulence gene expression. In addition, we detected 38 PfHP1-bound genes not enriched in H3K9me3. Many of these genes code for invasion proteins or proteins specifically expressed in different life-cycle stages suggesting a previously unrecognized role for PfHP1 in invasion pathway switching and life cycle progression. Furthermore, we show that PfHP1 is not associated with centromeric regions implying important differences in centromere biology compared to other eukaryotes. Consistent with a role of PfHP1 in virulence gene silencing we find that over-expression of PfHP1 leads to downregulation of 78 genes, the majority of which are located in heterochromatic domains. In summary, our results attribute an important role to PfHP1 in parasite biology and suggest a unifying PfHP1-dependent mechanism by which *P. falciparum* regulates the variegated expression of proteins involved in virulence and phenotypic variation.

## Results

### Identification and Functional Characterisation of *P. Falciparum* Heterochromatin Protein 1

HP1 belongs to a family of conserved chromatin proteins found from fission yeast to humans and is characterised by an N-terminal chromodomain, which binds H3K9me3, and a C-terminal chromoshadow domain implicated in homo- and heterodimerisation [56]. The *P. falciparum* genome encodes three putative chromodomain proteins amongst which PFL1005c was recently identified as the *P. falciparum* ortholog of HP1 (PfHP1) [49]. A multiple sequence alignment based on structural information on HP1 revealed a remarkable similarity between the chromodomains in PfHP1 and those in HP1 from various eukaryotes, including conservation of residues critical for interaction with H3K9me3 [57],[58] (Figure S1). Similarly, most of the conserved residues important for the C-terminal chromoshadow domain and homo- and hetero-dimerisation are also present [57], [59]–[61]. To confirm that PfHP1 indeed displays these structural and functional HP1-like properties we expressed PfHP1 in *E. coli*. Using pull down experiments, we showed that PfHP1-HIS binds specifically to H3K9me3 but not to unmethylated H3 (Figure S2). Furthermore, we experimentally verified homo-dimerisation of PfHP1 by mixed incubation of PfHP1-HIS with nuclear extracts prepared from 3D7/HP1-Ty parasites expressing a 2×Ty-tagged version of PfHP1 (Figure S2). These findings provide an independent confirmation of the results obtained by Perez-Toledo and co-workers [49] identifying PFL1005c as the *P. falciparum* ortholog of heterochromatin protein.

**Figure 1:**
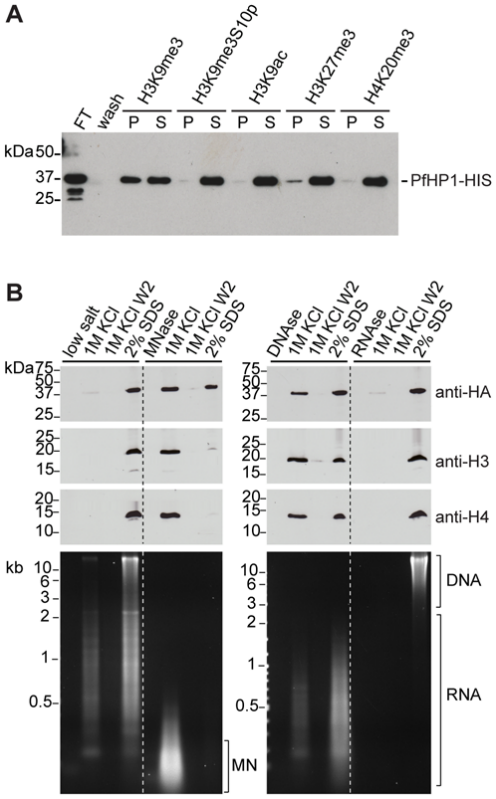
PfHP1 binds specifically to H3K9me3 and is associated with parasite heterochromatin. (A) Peptide competition demonstrates the specific binding of PfHP1 to H3K9me3. Recombinant PfHP1-HIS bound to a biotinylated H3K9me3 peptide was immobilized on streptavidin agarose beads. PfHP1 was only eluted by competition with the H3K9me3 peptide (lane 3), whereas peptides H3K9me3S10p (lane 5), H3K9ac (lane 7) and H4K20me3 (lane 11) were unable to compete. PfHP1 had weak affinity for H3K27me3 (lane 9). After elution with peptides, remaining PfHP1-HIS was eluted with high salt (lanes 4, 6, 8, 10, 12). P, peptide elution; S, high salt elution; FT, Flow-through after coupling of PfHP1-HIS; wash, last wash prior to peptide elution. The Western blot was probed with anti-6×HIS antibodies. (B) Solubility of PfHP1, H3, H4, gDNA and RNA after treatment of parasite nulcei with nucleases. Isolated nuclei were extracted either with low-salt, MNAse, DNAse or RNAse, followed by serial treatment of the insoluble pellets with high-salt and SDS. Lanes 1–4: PfHP1 and H3/H4 were tightly associated with the salt-insoluble fraction. Partially degraded RNA and undigested gDNA were apparent in the high-salt and SDS-soluble fractions. Lanes 5–8: Digestion with MNAse solubilizes approx. 50% of PfHP1 and the entire pool of H3/H4. RNA was degraded by the single-stranded nuclease activity of MNAse. Mononucleosomes (MN) were completely extracted with 1 M KCl. Lanes 9–12: After digestion of nuclei with DNAseI, PfHP1 and H3/H4 extracted equally with high-salt and SDS. Genomic DNA was completely digested and partially degraded RNA extracted with high-salt and SDS. Lanes 13–16: Treatment with RNAse A did not affect the extractablity of PfHP1 and H3/H4. RNA was completely digested by RNAse A whereas intact gDNA remained associated with the insoluble fraction. Equal amounts were analysed for each protein and nucleic acid sample. RNA in ethidium bromide stained gels was identified by re-examination of the gel after incubation in RNAse A-containing buffer for 1 hr at RT. W2, second wash after extraction with 1 M KCl.

To further test the specificity of the PfHP1-H3K9me3 interaction, we investigated binding of PfHP1 to a set of alternative histone modifications. Methylation of two other lysine residues in H3 and H4, H3K27me3 and H4K20me3, respectively, are commonly associated with transcriptional repression [62],[63]. Furthermore, phosphorylation of serine 10 adjacent to K9me3 (H3K9me3S10p) was found to counteract the repressive effect of H3K9me3 by preventing HP1 binding [64],[65]. Using a peptide competition assay, we show that PfHP1 interacts specifically with H3K9me3, whereas H3K9me3S10p, H3K9ac and H4K20 peptides were unable to interact with PfHP1 (Figure 1A). H3K27me3 interfered weakly with PfHP1-binding to H3K9me3 which was also observed for mouse HP1β [58]. The H3K9me3S10p and H3K27me3 marks have not been detected in *P. falciparum* to date [40] and therefore the biological significance of these findings remains to be determined. However, it is tempting to speculate that *P. falciparum* may use phosphorylation of H3S10 to reverse H3K9me3-mediated repression.

Consistent with its localization to heterochromatic regions, PfHP1 was insoluble after serial extraction of isolated parasite nuclei with low and high salt buffers (Figure 1B). After complete digestion of native chromatin with micrococcal nuclease (MNAse) or DNAseI a substantial fraction of PfHP1 remained associated with the high salt-insoluble pellet. Interestingly, the association of PfHP1 with the insoluble nuclear fraction was not sensitive to treatment with RNAse suggesting that in contrast to other eukaryotes, binding of PfHP1 to chromatin does not require RNA components [66]–[68]. These results demonstrate a tight association of PfHP1 with highly compact heterochromatic structures and/or the nuclear matrix.

### PfHP1 Localizes to Chromosome Ends in Discrete Foci at the Nuclear Periphery

**Figure 2:**
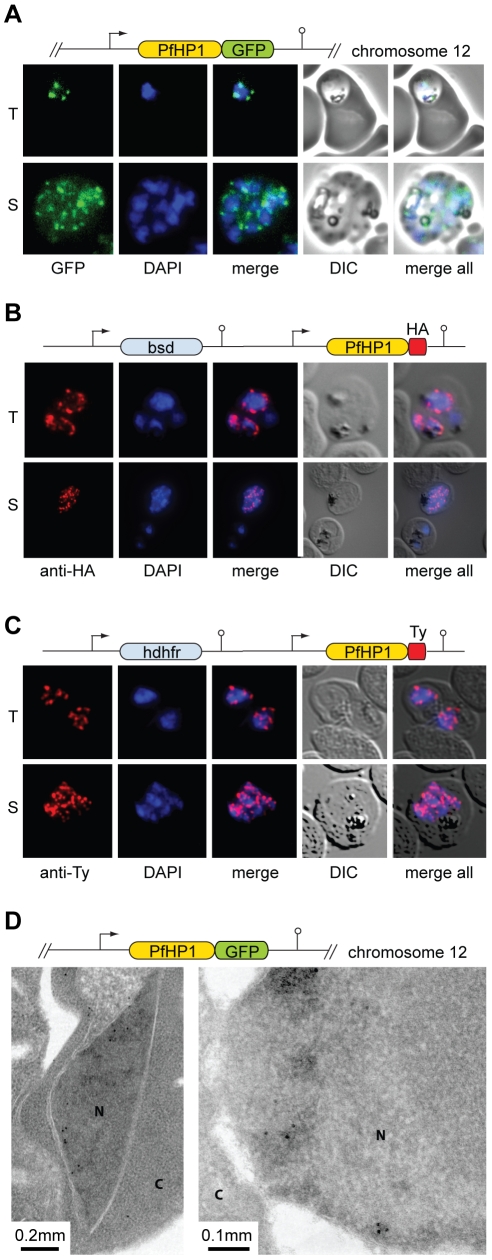
PfHP1 localizes to discrete regions at the nuclear periphery. (A) PfHP1-GFP localization in unfixed 3D7/HP1-GFP parasites. (B) Localization of PfHP1-HA and (C) PfHP1-Ty in methanol-fixed parasites visualized by IFA. T, trophozoites. S, schizonts. (D) Localization of PfHP1-GFP by electron microsopy. Antibodies specific to GFP detect PfHP1-GFP at the nuclear periphery, reminiscent of the pattern seen by fluorescence microscopy (N, nucleus; C, cytoplasm). Schematic maps of the transfection constructs are shown above each panel.

**Figure 3:**
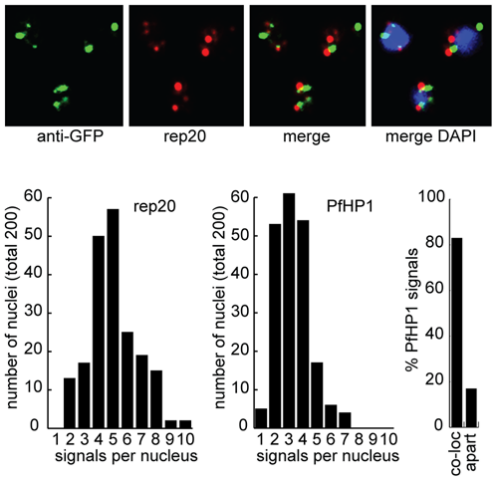
PfHP1 associates with chromosome end clusters. Combined IFA/FISH using 3D7/HP1-GFP ring stage parasites for simultaneous detection of chromosome end clusters and PfHP1-GFP. Fixed parasites were sequentially probed with anti-GFP antibodies (green) and the subtelomeric repeat sequence rep20 (TARE 6) (red). The distribution of GFP and rep20 signal numbers detected in 200 randomly selected nuclei and the frequency of GFP signals (659 in total) co-localising with, or directly adjacent to, rep20 signals are indicated.

Since *var* genes are dynamically associated with chromosome end clusters, we expected PfHP1 to be located in such defined perinuclear regions. To test this hypothesis we used three independent transgenic parasite lines. 3D7/HP1-GFP expresses a PfHP1-GFP fusion protein from its endogenous promoter, and 3D7/HP1-HA and 3D7/HP1-Ty express epitope-tagged versions of PfHP1 from episomal plasmids. Live cell imaging revealed that PfHP1-GFP located to the nucleus in a punctate perinuclear pattern reminiscent of chromosome end clusters (Figure 2A). 3D reconstruction verified the position of PfHP1 foci to the nuclear periphery (Video S1). Indirect immunofluorescence assays (IFA) confirmed these results and accentuated the localization of PfHP1 to discrete and well-defined regions at the nuclear periphery (Figures 2B and C). On average we observed 3.6 PfHP1-HA signals per trophozoite stage nucleus (3.64 (mean)±1.34 (s.d.); 302 nuclei counted). A detailed IFA experiment confirmed this restricted localization pattern in parasites carrying a single or multiple nuclei throughout intra-erythrocytic development (Figure S3). Next, we used immunoelectron microscopy to characterise PfHP1 localization at the ultrastructural level in 3D7/HP1-GFP parasites. PfHP1 was dominantly located at the nuclear periphery within and adjacent to previously described electron-dense regions reflecting perinuclear heterochromatin (Figure 2D) [34]. These high-resolution immunolocalizations corroborated and extended the light microscopy results, and together these data support the presence of PfHP1 at heterochromatic regions in the nuclear periphery. The PfHP1 localization pattern is consistent with multiple punctate foci of HP1 observed in *S. pombe*, plant and mammalian nuclei [69]–[71]. Finally, we asked if this discrete perinuclear localization pattern corresponds to chromosome end clusters by testing for a direct association of PfHP1 with subtelomeric DNA using combined IFA/FISH. In greater than 80% of cases PfHP1-GFP signals occurred directly adjacent to, or overlapped with, the subtelomeric repeat probe rep20 suggesting that PfHP1 is a major component of telomeric clusters (Figure 3). Surprisingly, we obtained a higher number of signals per nucleus for rep20 (5.02 (mean)±1.66 (s.d.)) compared to PfHP1 (3.3±1.21). In summary, our microscopy-based data identify PfHP1 as a major component of chromosome end clusters suggesting a preferential association of PfHP1 with *P. falciparum* subtelomeric regions.

### PfHP1 is Predominantly Associated with *P. falciparum*-specific Virulence Gene Clusters Throughout the Genome

**Figure 4:**
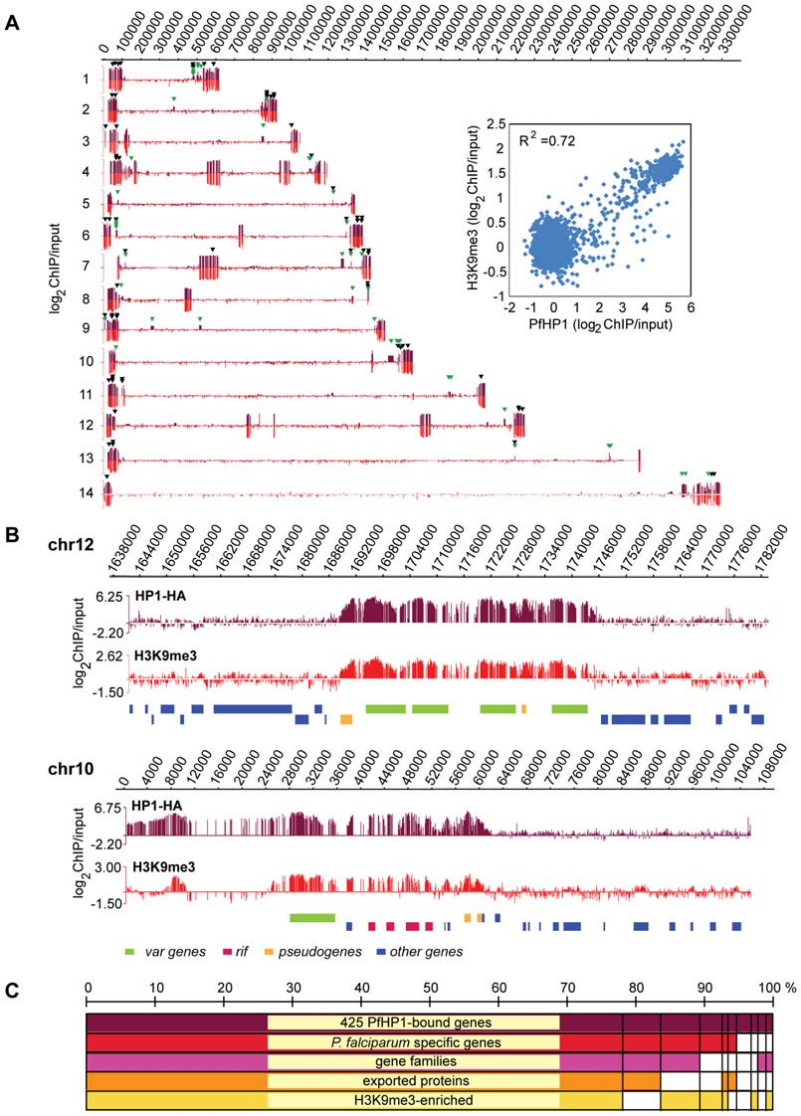
PfHP1 occupies virulence gene clusters throughout the genome and shows a high positive correlation with H3K9me3. (A) Genome-wide PfHP1 occupancy as determined by high-density ChIP-on-chip. Schematic display (SignalMap) and localization of genomic regions bound by PfHP1-HA on all 14 chromosomes in 3D7/HP1-HA schizont stage parasites. PfHP1-HA occupancy for each gene was calculated as the average of log_2_ ratios of hybridization values for immunoprecipitated and input chromatin. Genome-wide H3K9me3 localization data obtained with the same NimbleGen platform [46] are displayed on a negative scale. Chromosome numbers are indicated on the left, chromosomal positions on top. Green and black arrowheads identify PfHP1-bound loci that were either not enriched in H3K9me3 in two recent ChIP-on-chip studies, or for which no H3K9me3 data exist in the Lopez-Rubio study, respectively [37],[46]. The dot plot visualizes the high positive correlation between PfHP1 and H3K9me3 [46] for all genes in the 3D7 genome (R^2^ = 0.72). (B) PfHP1-binding demarcates *P. falciparum* virulence gene clusters that are also enriched in H3K9me3. Regional zoom-in of ChIP-on-chip profiles of PfHP1 and H3K9me3 occupancy [46] at a chromosome-central *var* gene cluster on chromosome 12 and the left end of chromosome 10. Chromosomal coordinates are according to PlasmoDB v5.5 annotation (www.plasmodb.org). PfHP1-bound gene types are color-coded below. (C) 387 of 425 PfHP1-bound genes are also enriched in H3K9me3 and are mostly members of lineage-specific exported gene families. Half of the 38 PfHP1-bound genes not enriched in H3K9me3 code for single copy genes. Clustering was performed according to information retrieved from PlasmoDB v5.5 (www.plasmodb.org) and the classification of exported proteins [3].

In light of the above results and the recently published genome-wide H3K9me3 patterns [37],[46] it was tempting to speculate that PfHP1 plays a dominant role in subtelomeric virulence gene silencing. To test this hypothesis and to identify additional potential PfHP1 target loci we performed genome-wide chromatin immunoprecipitation (ChIP-on-chip) using a high-density whole genome tiling array (NimbleGen Systems Inc.) [46]. We observed a striking association of PfHP1 with subtelomeric regions on all parasite chromosomes (Figure 4A, Tables S1 and S2). These domains covered TARE repeat blocks and extended inwards including all subtelomeric *var* genes. Additional internal PfHP1-bound islands were identified on chromosomes 4, 6, 7, 8 and 12, which in most cases defined chromosome-central *var* gene clusters. Hence, PfHP1 binds to the full complement of subtelomeric and chromosome-internal *var* genes. PfHP1 occupancy was not restricted to *var* loci but instead covered extended regions including all members of other gene families shown to be expressed in a clonally variant manner, including *rif*, *stevor* and *Pfmc-2tm* [17]–[19] (Figure 4B). This peculiar association is even more striking considering that PfHP1 was hardly detected at loci falling outside these chromosomal regions. In fact, the immediate boundaries between PfHP1-occupied and PfHP1-free regions delineate the sites of species-specific indels or synteny breakpoints between *P. falciparum* and other *Plasmodium* species [3],[72]. 95% of all 425 PfHP1-bound genes code for *P. falciparum*-specific proteins most of which are involved in host-parasite interactions (Figures 4C, S4 and Table S2). Almost all of these genes are members of subtelomeric gene families coding for proteins exported to the erythrocyte (*var*, *rif*, *stevor*, *pfmc-2tm*, *surfin*, *pfacs*, *fikk* kinases) or predicted to be exported (*phista*, *phistb*, *phistc*, *dnajI*, *dnajIII*, *a/b hydrolases*, *hyp1 to hyp17*) (Figure 4C and Table 1).

**Table 1:** Association of PfHP1 with members of *P. falciparum* gene families coding for exported proteins.

Gene family	# of genes^a^	PfHP1 pos.	H3K9me3 pos.^b^	Protein localization	Clonal variation^c^	Orthologs in other Plasmodia^d^	Refs
*var*	60	all	yes/yes	RBC surface	yes	-	[11],[12],[14],[15]
*rif*	165	all	yes/yes	RBC surface	yes	-	[18], [112]–[116]
*stevor*	31	all	yes/yes	RBC surface, MC	yes	-	[19], [116]–[118]
*pfmc-2tm*	10	all	yes/yes	MC	yes	-	[17],[119]
*surfin*	11	8/11	-/yes	RBC surface	yes	-	[91],[120]
*pfacs*	11	5/11	yes/yes	RBC	yes	-	[121],[122]
*fikk kinase*	20	2/20	-/yes	MC	yes	-	[123],[124]
*gbph*	2	all	-/yes	RBC	yes	-	[18],[125]
*phista*	25	17/25	yes/yes	export predicted	yes	-	[3],[18]
*phistb*	25	5/25	yes/yes	export predicted	n.d.	-	[3]
*phistb (dnaj)*	7	1/7	yes/-	export predicted	n.d.	-	[3]
*phistc*	16	1/16	-/yes	export predicted	n.d.	yes (16)	[3]
*dnajI*	3	-	-	export predicted	n.d.	yes (1)	[3]
*dnajIII*	16	5/16	-/yes	export predicted	n.d.	-	[3]
*a/b hydr.*	8	1/8	-/yes	export predicted	n.d.	-	[3]
*hyp1*	2	-	-	export predicted	n.d.	-	[3]
*hyp2*	3	-	-	export predicted	n.d.	-	[3]
*hyp4*	8	1/8	-/yes	export predicted	n.d.	-	[3]
*hyp5*	8	-	-	export predicted	n.d.	-	[3]
*hyp6*	3	2/3	yes/yes	export predicted	n.d.	-	[3]
*hyp7*	3	all	-/yes	export predicted	n.d.	-	[3]
*hyp8*	2	-	-	export predicted	n.d.	-	[3]
*hyp9*	5	-	-	export predicted	n.d.	-	[3]
*hyp10*	2	all	-/yes	export predicted	n.d.	-	[3]
*hyp11*	5	-	-	export predicted	n.d.	yes (4)	[3]
*hyp12*	3	-	-	export predicted	n.d.	-	[3]
*hyp13*	2	1/2	-/yes	export predicted	n.d.	-	[3]
*hyp15*	4	2/4	yes/yes	export predicted	n.d.	-	[3]
*hyp16*	2	-	-	export predicted	n.d.	yes (2)	[3]
*hyp17*	2	all	yes/yes	export predicted	n.d.		[3]
*PFD0075-like*	10	6/10	yes/yes	n.d.	n.d.	-	[37]
*MAL8P1.335-like*	4	all	yes/yes	n.d.	n.d.	-	[37]

MC, Maurer's clefts; n.d., not determined; in addition to the references listed in the last column, gene data were retrieved from PlasmoDB (www.plasmodb.org).

a Data retrieved from PlasmoDB and [3].

b Data retrieved from refs [37] (Lopez-Rubio et al.)/ [46] (Salcedo-Amaya et al.).

c For some of the gene families microarray data indicates variegated expression [87],[88].

d Numbers in brackets represent the number of *P. falciparum* genes in each family that have an ortholog in the *P. vivax/knowlesi* or rodent *Plasmodium* species, or both [3].

In addition, some other lineage-specific genes were also occupied by PfHP1: invasion-related genes *Pfrh1* [73] and *Pfrh3* (pseudogene) [74], liver stage antigen 1 (*lsa1*) [75], the gametocyte-specific gene *Pf11-1* [76], non-syntenic tRNA and rRNA loci, and a number of genes coding for hypothetical proteins. Surprisingly few PfHP1-bound loci code for proteins with orthologs in other *Plasmodium* species. These include members of the *rhoph1/clag* family (*clag2*, *clag3.1*, *clag3.2*) involved in erythrocyte invasion [77]; genes implicated in the development of sexual stages: *ccp1* [78],[79], *pfs230* [80],[81], putative dynein heavy chains [82]; *crmp1* and *crmp4* expressed in sporozoites [83]; and PFL1085w, coding for an ApiAP2 transcription factor [84].

Compared to Salcedo-Amaya and colleagues [46], who used the same NimbleGen array for genome-wide H3K9me3 mapping, we found a high level of local as well as genome wide correlation between the presence of this repressive mark and PfHP1 (R^2^ = 0.72) (Figure 4A and B). Out of 425 PfHP1-bound genes, we found only 44 genes that were not classified as H3K9me3-enriched, and one H3K9me3-associated gene was devoid of PfHP1-binding. In contrast, 155 PfHP1-associated genes were not classified as H3K9me3-enriched in the study by Lopez-Rubio et al. (68 of which were not represented on their array) [37], while all but six genes enriched in H3K9me3 were also occupied by PfHP1. By lowering the enrichment threshold for the latter study, PfHP1-occupancy correlated well with both genome-wide H3K9me3 localization datasets showing that 387 out of 425 PfHP1-bound loci were consistently enriched in H3K9me3 (Table S2). Most of these genes are members of gene families coding for proteins exported to the erythrocyte and implicated in parasite virulence (Figure 4C). Surprisingly, about half of the genes bound by PfHP1 but devoid of the H3K9me3 mark are single copy genes and members of small gene families coding for invasion proteins or proteins expressed in different life-cycle stages (Figure 4C and Table S2). This raises the interesting possibility that PfHP1 may be recruited to these loci in an H3K9me3-independent manner. Alternatively, this discrepancy may be related to overexpression of PfHP1, or to the use of different parasite lines and/or ChIP protocols.

**Figure 5:**
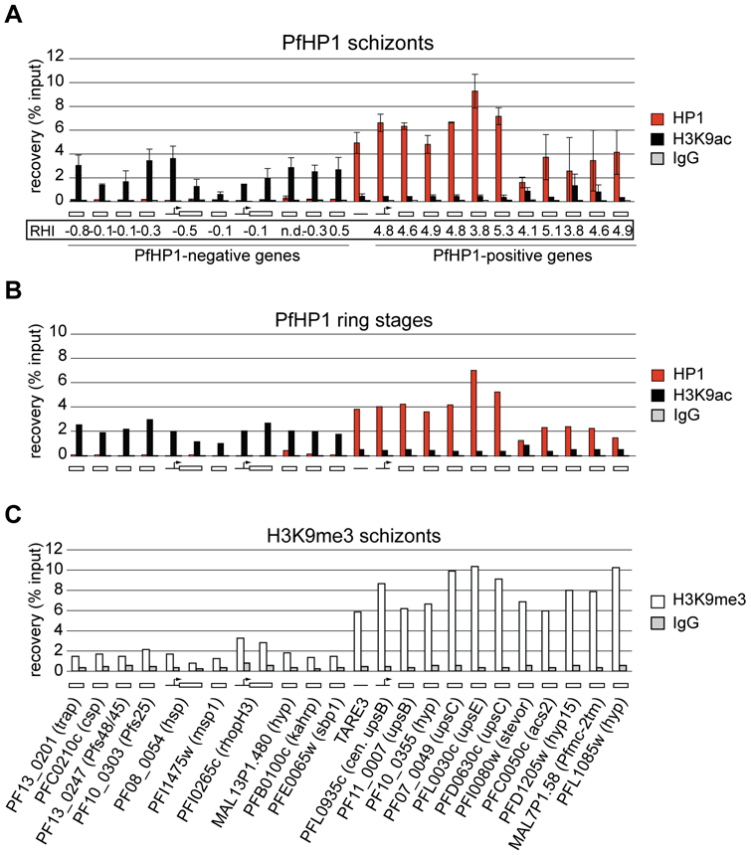
Validation of ChIP-on-chip results and mutually exclusive association of PfHP1/H3K9me3 and H3K9ac at *P. falciparum* virulence gene loci. (A) Targeted ChIP-qPCR analysis demonstrates mutually exclusive presence of PfHP1/H3K9me3 and H3K9ac at selected loci. Recovery values of cross-linked PfHP1- and H3K9ac-associated chromatin from ten and twelve randomly selected loci tested negative or positive for PfHP1 in ChIP-on-chip, respectively, represent the mean from three independent experiments on schizont stage samples (twice 3D7/HP1-HA, once 3D7/HP1-GFP). Relative hybridisation intensities (RHI) from the ChIP-on-chip analysis are shown below each locus and indicate log_2_ ratios of recovered chromatin over input. (B) PfHP1 and H3K9ac profiles at the same loci in ring stage parasites are comparable to schizont stages. (C) H3K9me3-association in schizont stages was determined by ChIP-qPCR using non-cross-linked chromatin (native ChIP). Values derive from one of two independent experiments (3D7/HP1-HA and 3D7 wild-type) yielding similar results. qPCR primers (Table S4) targeted coding regions (open box) or non-coding promoter (solid line with arrow) or subtelomeric repeat (solid line) regions. Gene accession numbers are indicated below the bottom graph. Normal rabbit polyclonal IgG was used as negative control for ChIP.

To validate the ChIP-on-chip results we investigated the association of PfHP1 with individual loci by ChIP-qPCR. We targeted ten and twelve randomly selected loci, which showed either a negative or a positive association with PfHP1 in the ChIP-on-chip experiment, respectively (Figure 5 and Figure S5). These results confirmed the ChIP-on-chip findings in all instances, showing that PfHP1 is associated with subtelomeric and internal virulence genes but not with genes that showed no association with PfHP1 in the ChIP-on-chip experiment (Figure 5A). No chromatin fragments from transgenic parasite lines were recovered with rabbit IgG control antibodies or anti-HA/anti-GFP antibodies used on 3D7 wild-type parasites (data not shown), demonstrating the specificity of these results. Importantly, and consistent with a role of PfHP1 in stably inherited heterochromatic silencing, PfHP1-occupancy was present at the same loci in next generation ring stage parasites (Figure 5B). As expected, PfHP1-positive genes were also enriched in H3K9me3, which confirms the genome-wide colocalization and underscores the *in vivo* relevance of the PfHP1/H3K9me3 interaction in virulence gene silencing and the mutually exclusive presence of H3K9ac and H3K9me3 (Figure 5C). In contrast, genes not bound by PfHP1 were generally enriched in H3K9ac although this association did not necessarily correlate with active transcription of these loci. This is not surprising in light of recent findings demonstrating that H3K9ac occupancy did not differ markedly between the coding regions of active and inactive genes in *P. falciparum* [46].

In summary, our results demonstrate an extraordinarily confined localization of PfHP1 throughout the genome, and an extensive colocalization with the repressive histone mark, H3K9me3. This implies an important role for PfHP1 in epigenetic regulation of exported virulence factors and indicates that variegated expression and phenotypic variation may represent a general, rather than exceptional, feature of most *P. falciparum*-specific or expanded gene families.

### PfHP1 is Not Enriched at *P. falciparum* Centromeres

In other eukaryotes like *S. pombe* and *D. melanogaster*, HP1 is an important factor in centromere function and a major constituent of pericentromeric heterochromatin [48]. We observed no evident presence of PfHP1 in these domains, albeit the average level of PfHP1 ChIP-on-chip signal over genes directly adjacent to centromeres was somewhat higher as compared to the rest of the genome (Figure S6 and Table S1). To test if PfHP1 was indeed enriched at centromeres we performed ChIP-qPCR experiments targeting the centromeres on eight chromosomes and six genes directly up- or downstream of centromeres which displayed low-level PfHP1-binding in ChIP-on-chip. We were unable to detect binding of PfHP1 to these regions as none of the loci tested showed any sign of PfHP1 enrichment (Figure S6). These findings are in line with the observed absence of H3K9me3 marks at centromeric regions and suggest that *P. falciparum* centromere biology and chromosome segregation are independent of PfHP1.

### PfHP1 Mediates Virulence Gene Silencing

HP1 has been implicated in gene silencing [85],[86] and hence we were interested in testing this proposed function of PfHP1 in *P. falciparum*. Although recent whole transcriptome analyses strongly suggest that most subtelomeric gene families are expressed in a restricted manner [87],[88], a formal demonstration of a direct link between PfHP1 and gene expression is lacking. We therefore focused on a possible genome-wide correlation by transcriptional profiling using RNA isolated at four consecutive timepoints across the intra-erythrocytic developmental cycle (IDC) from two biological replicates of the PfHP1-overexpressing line (Table S3). PfHP1 target genes displayed significantly lower absolute expression levels as compared to all other genes at all IDC stages (p<0.001, Wilcoxon ranksum test) (Figure S7). It is noteworthy that many PfHP1-negative genes are also weakly or not expressed during the IDC, stressing the notion that PfHP1 is not a general marker for inactive genes and that other processes such as gene-specific regulation participate in developmental and cell-cycle-dependent transcriptional control.

**Figure 6:**
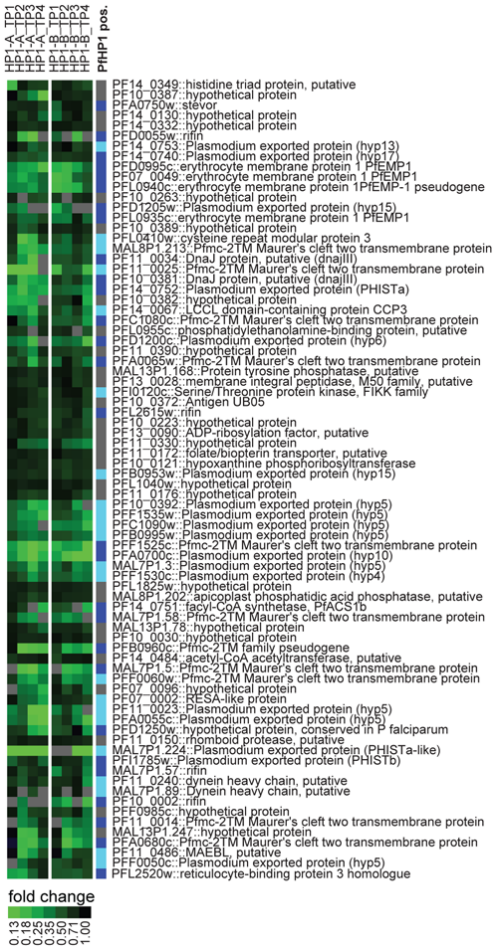
PfHP1 participates in silencing of variegated genes. Significance Analysis for Microarrays (SAM) revealed 78 genes consistently down-regulated in two biological replicates (A and B) upon over-expression of PfHP1. The heat map compares the relative transcript abundances at four timepoints across the IDC (early ring stage (TP1), late ring stage (TP2), trophozoites (TP3), schizonts (TP4)) between the PfHP1-overexpressing and control lines. The right column highlights genes bound by PfHP1 in the ChIP-on-chip experiment (dark blue boxes), or genes that are members of PfHP1-bound gene families (light blue boxes). Gene accession numbers and annotations are according to PlasmoDB v5.5 (www.plasmodb.org).

We were also interested in testing the effect of perturbations in PfHP1 expression on global gene transcription. Several attempts to generate a PfHP1-null mutant failed suggesting an essential role for this protein in parasite biology. We therefore investigated if over-expression of PfHP1 had any effect on gene transcription by comparing mRNA levels of all genes in the transfected lines to those in a control line. We detected 78 genes that were consistently down-regulated in two biological replicates and none that were upregulated (Figure 6). Of these, 50 are members of PfHP1-demarcated gene families, 28 of which showed a greater than three-fold enrichment for PfHP1 in the ChIP-on-chip experiment (p-value 7.76E-36), including nine of the ten *pfmc-2tm* family members. Importantly, this analysis identified additional PfHP1 target genes that were either not detected, or classified as below three-fold enriched, in the ChIP-on-chip experiment and showed no sign for H3K9me3 enrichment. These include all members of the *hyp5* family and additional members of the *crmp*, *ccp*, *eba* and dynein heavy chain families. At this stage, however, it remains unknown if the down-regulation of these genes is due to a direct or indirect effect of PfHP1 over-expression. Hence, increased levels of PfHP1 enhanced silencing of variegated genes and had only minor effects on global gene transcription. These results are consistent with a dosage-dependent effect of PfHP1 and suggest further that unwanted heterochromatin spreading is efficiently prevented by defined boundary structures. Furthermore, our approach demonstrates that over-expression studies by transcriptional profiling may be employed to investigate the function of regulatory proteins in *P. falciparum* gene expression.

## Discussion

In this study we present a comprehensive analysis of *P. falciparum* HP1 and describe the first genome-wide binding profile of a non-histone chromatin component in this important pathogen. Our findings reveal important insights into the regulatory strategy employed to control the variegated expression of a large class of highly specialized virulence genes. This knowledge will be instrumental for future investigations to understand parasite virulence and survival.

We have shown that PfHP1 binds specifically to H3K9me3 and forms stable homodimers *in vitro*, which are both conserved features of HP1 in other eukaryotes. A recent study used similar approaches to demonstrate these biochemical features for PfHP1 [49]. *In vivo*, PfHP1 associates extensively with subtelomeric repeats and genes encoding virulence factors in both subtelomeric and chromosome-internal loci. The conserved organisation of subtelomeric regions into blocks of distinct subtelomeric repeat units followed by multiple members of various gene families is a hallmark feature of *P. falciparum* chromosome ends. Unknown protein(s) mediate physical linking of chromosome ends in the formation of telomeric clusters [30]. The findings presented here identified PfHP1 as a major constituent of these chromosome end clusters. Our nuclear fractionation results suggest that PfHP1 is a candidate protein responsible, at least in part, for the physical clustering of chromosome ends through interactions between the chromoshadow domain and other structural components.

Whether chromosome-internal heterochromatic domains are an integral part of chromosome end clusters, or rather represent physically distinct entities at the nuclear periphery remains a matter of debate. Our ChIP-on-chip results demonstrated that PfHP1 associates with all subtelomeric regions. Hence, if chromosome-internal PfHP1-enriched regions form entities distinct from telomeric clusters one would expect a higher number of perinuclear PfHP1 domains compared to the four to seven chromosome end clusters usually detected by FISH [29],[30], which we never observed. Furthermore, in our IFA/FISH experiments the average number of rep20 signals was higher than that of PfHP1. At this stage, we don't know if this observation is due to the actual absence of PfHP1 from some chromosome end clusters or to differential sensitivities of IFA- and FISH-based target detection. However, both results clearly argue against a location of central heterochromatic domains separate from telomeric clusters. On the other hand, our IFA/FISH results may also be consistent with the idea that the few perinuclear PfHP1 foci not co-localising with rep20 reflect chromosome-internal heterochromatic regions that are physically distinct from chromosome end clusters, as has been suggested by others [37]. We believe that both opposing hypotheses are related to technical limitations inherent to FISH and IFA/FISH experiments resulting in a failure to detect the full complement of DNA and protein targets simultaneously. To know which scenario reflects the *in vivo* situation, more refined approaches such as locus tagging, confocal microscopy and/or 3C and 4C chromosome conformation capture techniques need to be applied.

We have shown that the majority of heterochromatic protein-coding genes are located subtelomerically directly adjacent and in smooth transition to the non-coding TARE region. A number of additional PfHP1 islands are also found at chromosome-internal clusters. In total, PfHP1 binds to 425 genes reflecting 7.5% of the parasite's coding genome. Notably, all heterochromatic coding domains are contained within sharply defined boundaries, which in most cases reflect non-syntenic regions. In other words, nearly all of PfHP1-bound genes code for proteins that do not have orthologs in other organisms and are thus specific to the *P. falciparum* lineage. This set of PfHP1-bound genes compares well with the genome-wide pool of H3K9me3-enriched loci described recently [37],[46]. This strong correlation is highly relevant for our understanding of the *in vivo* role of the PfHP1/H3K9me3 interaction and underscores its significance in *P. falciparum* virulence gene silencing. Some genes associated with PfHP1 were enriched in H3K9me3 in the Salcedo-Amaya study [46] but not in the Lopez-Rubio study [37]. These include members of the *surfin*, *fikk* kinase and *gbph* families as well as members of uncharacterised gene families such as *hypxx* and exported co-chaperones [3] (see also Table 1). We attribute this discrepancy to an improved performance of native versus formaldehyde-crosslinked H3K9me3 ChIP rather than to the actual absence of this histone mark at these loci.

The vast majority of PfHP1-demarcated gene families code for proteins that are exported into the host erythrocyte to participate in the processes of host cell remodeling, immune evasion and cytoadherence [3],[89],[90]. A hallmark in the epigenetic regulation of *var* gene transcription is the strict mutually exclusive expression of a single family member. The demonstrated association of PfHP1/H3K9me3 with *var* genes significantly advances our knowledge of the mechanisms underlying mutually exclusive *var* gene expression and may serve as a model system to understand the regulation and biological role of other virulence gene families. Clonal variation was also experimentally demonstrated for expression of a subset of PfHP1-bound gene families including *rif*, *stevor*, *pfmc-2tm* and *surfin* [17]–[19],[91]. Furthermore, several transcriptional profiling studies indicate restricted transcription of additional exported gene families that are also associated with PfHP1/H3K9me3. In view of the well-described role of HP1 in regional gene silencing, this remarkable association hints at an overall strategy to control phenotypic variation of a large pool of protein families that evolved to facilitate survival in a hostile environment. The expansion of lineage-specific exported protein families is much more pronounced in *P. falciparum* compared to other *Plasmodia* [3]. This observation is most likely related to the trafficking of PfEMP1 and other proteins to the erythrocyte surface and probably associated with the high virulence of *P. falciparum*. It is therefore tempting to speculate that the continuous expansion of *P. falciparum* exported gene families from single ancestral gene types ultimately required the parallel evolution of an epigenetic system to ensure phenotypic variation and avoid premature exhaustion of the antigenic repertoire. It is noteworthy that all but one (PFI1780w, *phistc*) of the genes coding for the core complement of 36 exported proteins shared between different *Plasmodium* species [3] are not associated with PfHP1. This is indicative for conserved essential functions of these ancestral proteins in the trafficking of exported proteins that evolved before lineage-specific expansion of virulence gene families.

The multi-step process of merozoite invasion into erythrocytes is characterised by the sequential action of apically located proteins encoded by gene families such as *eba*, *Pfrh* and *rhopH/clag* [92]. Variations in the expression of these genes are linked to alternative invasion pathways involving different ligand-receptor interactions. For instance, in isogenic 3D7 lines using either a sialic-acid dependent or independent invasion pathway, *clag2*, *clag3.1* and *clag3.2* show clonal variation and, for the latter two genes, are transcribed in a mutually exclusive manner [93]. Similarly, members of the *Pfrh* and *eba* families were shown to be differentially expressed in different parasite strains [94]–[97]. The association of PfHP1 with invasion gene families provides an explanation for these observations and important information about the epigenetic mechanisms responsible for invasion pathway switching. Our results further indicate that some genes expressed at different life cycle stages are controlled by PfHP1, including genes important during gametocyte maturation, sporozoite targeting to the salivary glands, or intra-hepatic development. However, these genes reflect only a small fraction compared to the full complement of developmentally regulated genes in *P. falciparum* indicating that life cycle stage conversion is mostly regulated by other mechanisms.

Telomeric and pericentromeric heterochromatin represents a conserved structural feature important in genome stability, proper segregation of chromosomes and prevention of telomere fusions in *S. pombe* and higher eukaryotes [98]. At both locations, heterochromatin is strictly dependent on repetitive DNA and physical interactions with siRNA and the RNA-induced transcriptional silencing (RITS) complex [99]–[101]. Our and other recent findings suggest that pericentromeric heterochromatin does not exist in *P. falciparum*. First, while PfHP1 is clearly enriched in subtelomeric repeats, we were unable to detect PfHP1 at centromeres or in pericentromeric regions by ChIP-qPCR, and H3K9me3 is also absent [37],[46]. Second, we provide evidence that the binding of PfHP1 to chromatin is not mediated by an RNA component. Third, components of the RNAi machinery and the RITS complex are absent in the *P. falciparum* proteome [102]. Therefore, although transcription of non-coding RNAs from *P. falciparum* centromeric regions has been reported [103], their role in formation of pericentromeric heterochromatin in the absence of PfHP1, H3K9me3 and a discernable RITS complex seems unlikely. Together, these observations indicate that PfHP1 plays no role in centromere biology and chromosome segregation and suggests a striking difference between *P. falciparum* and other eukaryotes including the human host.

We have shown that the presence of PfHP1 is directly linked to low expression of target genes. Furthermore, most of the genes down-regulated upon PfHP1 over-expression are members of variegated gene families, an effect that was also reported in *D. melanogaster* [104],[105]. These findings highlight the role of PfHP1 in variegated virulence gene expression. The sudden drop in PfHP1 occupancy at the boundaries of all heterochromatic regions is striking and supports the notion of functional genome partitioning in *P. falciparum* to secure expression of essential genes outside of these domains. The *cis*-acting sequences involved in the formation of these boundaries are unknown, but fine-mapping of the regions identified in this study will help to identify such elements. In *S. pombe*, tRNA loci are implicated in the boundaries of pericentromeric heterochromatin [106]. Interestingly, the three tRNA loci where ChIP-on-chip data were available were all enriched in PfHP1, two of which map exactly to the borders of heterochromatic domains on chromosome 7. Similarly, two tRNA loci on chromsomes 4 and 13 were shown to be enriched in H3K9me3 [37]. We were unable to analyse a PfHP1 loss-of-function phenotype due to the refractoriness of PFL1005c to gene disruption. It is noteworthy that compared to *S. pombe*, which encodes two HP1 variants and is viable after deletion of Swi6/HP1 [56], *P. falciparum* encodes a single HP1 protein only, suggesting that PfHP1 is essential for parasite survival. It is conceivable that one deleterious effect of PfHP1 removal would be caused by flooding of the parasite with large numbers of exported proteins. However, we also predict essential roles for PfHP1 in the aforementioned aspects of genome organisation and telomere integrity.

To the best of our knowledge, the remarkable distribution of PfHP1 to a large complement of functionally clustered genes has not been described in any other organism. In *D. melanogaster*, HP1 and the cognate histone methyltransferase SU(VAR)3-9 are associated with genes specifically expressed during embryogenesis or male development [107]. Likewise, genome-wide Swi6/HP1-association mapping in *S. pombe* identified only a small number of mostly meiotically expressed target genes [106]. In conclusion, the important role of PfHP1 in controlling parasite virulence uncovers a novel aspect of HP1 function. We hypothesise that other *Plasmodium* species use a similar strategy, and it will be interesting to see if other apicomplexan parasites and pathogenic fungi also employ HP1 for variegated expression of contingency gene families. Furthermore, our results set the stage for the identification of additional heterochromatin components and regulatory factors involved in epigenetic control of *P. falciparum* virulence gene expression. Detailed knowledge of these processes will be important for our understanding of this widely used survival strategy of pathogens and may uncover novel ways to interfere with pathogenesis and disease.

## Materials and Methods

### Parasite culture and transfection

*P. falciparum* 3D7 parasites were cultured, synchronised and transfected as described [23]. Transfection constructs were generated according to standard procedures (Protocol S1). GFP-tagged endogenous PfHP1 was obtained by single crossover integration. Parasites were cloned by limiting dilution.

### Recombinant protein expression

Sequences were amplified from 3D7 gDNA and cloned into pET24a(+) (Novagen). PfHP1-HIS was expressed in *E. coli* Tuner (DE3) (Novagen) at 37°C in TB containing 1% glucose and induced at OD = 1.0 for 4 hrs with 1 mM IPTG. Soluble extracts were prepared by freeze/thaw lysis.

### PfHP1 pull-down, peptide competition and dimerisation assays

*E. coli* lysate containing PfHP1-6×HIS was incubated with biotinylated H3 peptides (Upstate) immobilized on streptavidin agarose beads (Pierce) at room temperature for 1 hr in 250 µl BB (20 mM Tris-HCl (pH 8), 150 mM NaCl, 1 mM EDTA, 0.1% Triton X-100). After six wash steps in BB (250 mM NaCl) bound proteins were eluted in Laemmli buffer and analysed by SDS-PAGE. For peptide competition assays, PfHP1-HIS bound to biotinylated H3K9me3 peptide immobilized on streptavidin beads was eluted using a 10-fold excess of non-biotinylated histone peptides H3K9me3, H3K9me3S10p, H3K9ac, H3K27me3, H4K20me3 (Diagenode, sp-056-050, sp-128-050, sp-004-050, sp-069-050, sp-057-050) in buffer BB (250 mM NaCl). After peptide elution, beads were treated with 1 M NaCl to elute remaining PfHP1-HIS and all supernatants were analysed by SDS-PAGE and Western. Homo-dimerisation of PfHP1 was tested by co-incubation of a 1 M KCl nuclear extract prepared from 3D7/HP1-Ty with *E. coli* lysate containing PfHP1-6×HIS or non-transformed *E. coli* lysate in presence of 1% sarkosyl. Proteins were diluted six times with DB (20 mM Tris-HCl (pH 8), 250 mM NaCl, 20 mM imidazol, 0.5% Tween20), and combined with 10 µl Ni-agarose. Beads were washed four times in DB and bound proteins were eluted as above.

### Nuclear fractionation

Nuclear fractionation involved sequential extraction of proteins from isolated parasite nuclei with low salt, digestion with either DNAseI, MNAse, or RNAseA, followed by extraction in 1 M KCl and finally 2%SDS (for details see Protocol S2). The nuclear fractions were analysed by Western blot.

### Western blot analysis

Primary antibody dilutions were: anti-HA 3F10 (Roche Diagnostics) 1∶1,000; anti-Ty BB2 (kind gift of K. Gull) 1∶5,000; anti-6×HIS (R&D Systems) 1∶5,000; anti-H3 (Abcam, ab1791) 1∶40,000; anti-H4 (Abcam, ab10158) 1∶10,000.

### Fluorescence microscopy and 3D reconstruction

Methanol-fixed cells were analysed using rat anti-HA 3F10 (1∶100) or mouse anti-Ty (1∶1,000). Alexa-Fluor® 568-conjugated anti-rat IgG (Molecular Probes) 1∶500; FITC-conjugated anti-mouse IgG (Kirkegaard Perry Laboratories) 1∶300. Images were taken on a Leica DM 5000B microscope with a Leica DFC 300 FX camera and acquired via the Leica IM 1000 software. Nuclei of unfixed 3D7/HP1-GFP cells were stained with DAPI before mounting onto a glass slide. Images were taken on a Carl Zeiss Axioskop microscope with a PCO SensiCam camera. 3D nuclear reconstruction was achieved by taking sequential z-stack series using a Carl Zeiss Axiovert 200 M microscope with an AxioCam MRm camera. Images were deconvolved and 3D reconstruction was performed using Axiovision v4.2 software. Images were processed using Adobe Photoshop CS2.

### Combined IFA/FISH

Parasites were fixed with 4% formaldehyde and 0.0075% glutaraldehyde and permeabilized with 0.1% Triton-X. Cells were incubated with anti-GFP antibody (kind gift of E. Handman) (1∶200) followed by anti-rabbit IgG-fluorescein (Invitrogen) (1∶200). FISH was carried out using a rep20 probe as previously described [30],[35].

### Transmission electron microscopy

3D7/HP1-GFP were fixed in 1% glutaraldehyde for 1 h at 4°C, dehydrated, and embedded in LR Gold resin (Electron Microscopy Sciences, Fort Washington, PA). Ultrathin sections were cut using a Leica Ultracut R microtome, labeled with polyclonal rabbit anti-GFP (Sigma) and 10 nm colloidal gold goat-anti-rabbit IgG (SPI). Sections were poststained with uranyl acetate and lead citrate and observed using a Philips CM120 BioTwin Transmission Electron Microscope.

### Chromatin immunoprecipitation (ChIP)

ChIPs were carried out using formaldehyde crosslinked chromatin, except for anti-H3K9me3, which was analyzed using native MNase-digested chromatin [46]. Cross-linked chromatin was prepared by adding 1% formaldehyde to synchronized parasite cultures (5×10E8 schizonts or 2×10E9 ring stages) and incubated for 10 min at 37°C. Crosslinking was terminated by addition of 0.125 M glycine final concentration. After saponin lysis, nuclei were separated using a 0.25 M sucrose buffer cushion and sheared by sonication in a Bioruptor UCD-200 (Diagenode) for 15 min at 30 sec intervals (size range 100–500 bp). 400–500 ng DNA-containing chromatin was incubated with 1 µg antibody (anti-HA 3F10 (Roche); anti-GFP (AbCam, ab290); H3K9ac (Diagenode); IgG rabbit polyclonal (UpState 12–370) (used as a negative control)) in presence of 10 µl A/G sepharose beads (Santa Cruz Biotechnology) overnight at 4°C. After extensive washes immunoprecipitated chromatin was eluted with 1% SDS and 0.1 M NaHCO_3_ and de-crosslinked at 65°C for 4 hrs. DNA was purified using PCR purification columns (Qiagen).

Native chromatin was prepared from freshly isolated nuclei by MNase digestion and subsequent extraction with salt-free buffers (10 mM Tris pH 7.4, 1 mM EDTA; 1 mM Tris pH 7.4, 0.2 mM EDTA). Chromatin was diluted in 2×ChIP incubation buffer (100 mM NaCl, 20 mM Tris pH 7.4, 6 mM EDTA, 1% Triton X-100, 0.1% SDS). 400 ng DNA-containing chromatin was incubated with 1 µg antibody (anti-H3K9me^3^ rabbit polyclonal #4861 [108] overnight at 4°C followed by the addition of 10 µl A/G beads and further incubation for 2 h. After washing with buffers containing 100, 150 and 250 mM NaCl, immuno-precipitated DNA was eluted and purified as described above (without de-crosslinking).

The efficiency of ChIP at specific genomic locations was tested by quantitative PCR (qPCR) (MyIQ sequence detector, BioRad) using primer sets described in Table S4. “Negative” and “positive” genes were randomly selected from the group of genes that showed ChIP/input ratios lower or greater than 1.6 (log 2) in the ChIP-on-chip experiment. The amount of target DNA recovered after immuno-precipitation was directly compared to a ten-fold dilution series of input DNA, and defined as percentage of input for each locus.

### ChIP-on-CHIP

For genome-wide analysis 24 individual anti-HA (AbCam, ab9110) ChIP reactions were combined using formaldehyde-crosslinked material. Immunoprecipitated DNA was amplified by a modified T7 linear amplification method [46]. Briefly, DNA fragments were dephosphorylated using calf intestinal alkaline phosphatase (NEB) and subsequently G-tailed with terminal transferase (NEB). T7 promoter was incorporated using Klenow polymerase (NEB) and T7C9B primer. RNA was synthesized by T7 polymerase (Ambion T7 megascript kit) and subsequently reverse transcribed using N6 primers. Amplified dsDNA was labeled with Cy3- (ChIP) or Cy5- (input) coupled random heptamers and hybridized to a tiling array (based on the May 2005 NCBI sequence of the *P. falciparum* genome; 385,000 probes with a median spacing of 48 bp, Roche NimbleGen) [46]. Log_2_ ratios were computed for each sample pair and after Tukey bi-weight normalization visualized by SignalMap software (Roche NimbleGen). Probes were mapped to the latest genome assembly and visualized in the context of PlasmoDB v5.5 genome annotation.

### Transcriptional profiling and data analysis

Growth of two independant transfectants over-expressing PfHP1 (PfHP1-A and -B) and the mock transfectant 3D7/camHG (K. Witmer et al., unpublished), was tightly synchronised in parallel three times by sorbitol treatment to achieve a 10 hr growth window. Total RNA was isolated at four timepoints across the IDC at early ring stages (4–14 hours post-invasion (hpi)), late ring stages (14–24 hpi), trophozoites (24–34 hpi) and schizonts (32–42 hpi) by lysis of pelleted RBCs in TriReagent (Sigma). RNA samples were analyzed using a *P. falciparum* microarray as previously described [109]. RNA from each time point and parasite line was labeled with Cy5 and hybridized against a RNA pool assembled from equal amounts of total RNA collected from the 3D7 strain at every 8 hrs. Absolute transcript abundance was determined as a mean of the sums of median Cy5 signal intensity on each microarray gene spot in all four time points. The relative abundance of individual transcripts was analyzed by Significance Analysis for Microarrays (SAM) as implemented by the MEV version 4.3 [110]. SAM (delta = 0.75) revealed 217 and 599 genes downregulated in PfHP1-A and PfHP1-B, respectively. Only two genes were found up-regulated in PfHP1-B.

### Accession numbers

The raw ChIP-on-chip data discussed in this publication have been deposited in NCBI's Gene Expression Omnibus [111] and are accessible through GEO Series accession number GSE17029 (http://www.ncbi.nlm.nih.gov/geo/query/acc.cgi?acc=GSE17029). Data remapped to the latest genome annotation has been submitted to PlasmoDB (www.plasmodb.org).

The PlasmoDB accession numbers for genes and proteins discussed in this publication are: PfHP1 (PFL1005c); *rh1* (PFD0110w); *rh3* (PFL2520w); *eba-165* (PFD1155w); *lsa1* (PF10_0356); *pf11-1* (PF10_0374); *clag2* (PFB0935w); *clag3.1* (PFC0120w); *clag3.2* (PFC0110w); *ccp1* (PF14_0723); *pfs230* (PFB0405w); dynein heavy chains (PFI0260c, MAL7P1.162); *crmp1* (PFI0550w); *crmp4* (PF14_0722); ApiAP2 protein (PFL1085w)

## Supporting Information

Figure S1 Multiple sequence alignment of PfHP1 with chromo (A) and chromoshadow domains (B) of HP1 orthologs from various species. The alignment was performed using ClustalW (www.ebi.ac.uk/clustalw) and manually adjusted according to published structural information [57]–[59],[61]. Amino acids conserved in the CD and CSD are colored green and pink, respectively, and those important in overall domain structure are colored orange. Asterisks above the chromodomain alignment denote residues important for methyl lysine recognition. In the chromoshadow domain, boxed residues are important for homo-dimerisation and the non-polar amino acid indicated by an asterisk has been shown to be essential in this interaction [57]. Residues denoted by a (+) are important for interaction with the semi-conserved PXVXL motif in HP1-interacting proteins. Sp, *S. pombe* Swi6/HP1 (NP_593449); Dd, *D. discoideum* HP1α (XP_639321); Nc, *N. crassa* HP1 (AAR19291); Hs, *H. sapiens* HP1α (NP_001120793); Mm, *M. musculus* HP1β (NP_031648); Dm, *D. melanogaster* Su(var)205 (NP_723361); Ce, *C. elegans* HP1-like (NP_510199). Amino acid positions within each sequence are indicated to the left and right of the alignment.
(0.30 MB PDF)

Figure S2 PfHP1-specific binding to H3K9me3 and PfHP1 homo-dimerisation. (A) Recombinant PfHP1 binds specifically to H3K9me3. Left panel: Coomassie-stained gel demonstrating the efficient coupling of biotinylated histone peptides (b-H3K9me3 and b-H3) to streptavidin agarose beads (lanes 2–5) and the specific pull-down of PfHP1-HIS from *E. coli* lysates with H3K9me3 (lane 7) but not with unmodified H3 peptide (lane 9). Right panel: Western blot using anti-6×HIS antibodies of the input, supernatant and bound fractions from the pull-down experiment confirm the specific binding of PfHP1 to H3K9me3. (B) Homo-dimerisation of PfHP1. Anti-Ty Western blot showing that PfHP1-HIS efficiently purifies PfHP1-Ty from parasite nuclear extracts.
(2.05 MB PDF)

Figure S3 PfHP1 localization across intra-erythrocytic development of *P. falciparum*. Merged DAPI/anti-HA/DIC images of methanol-fixed 3D7/HP1-HA parasites from early ring stages to segmented schizonts demonstrates a punctate perinuclear localization of PfHP1 throughout the IDC. A schematic of the transfection construct is shown on top.
(4.86 MB PDF)

Figure S4 The majority of PfHP1-bound genes are directly involved in host-parasite interactions. (A) Pie chart highlighting the proportion of PfHP1-bound genes falling into annotated/predicted Gene Ontology pathway groups. (B) All PfHP1-occupied genes were clustered according to their annotated/predicted participation in GO pathways (x-axis). The proportion of PfHP1-bound genes in each cluster (red bars) is compared to the proportion of genes in the entire genome falling into the same clusters (white bars).
(0.24 MB PDF)

Figure S5 Comparison of average ChIP-qPCR recovery values presented in Figure 5 (validation of genome-wide ChIP results by targeted ChIP). (A) Intimate link between PfHP1-occupancy and the presence of H3K9me3 in chromatin at selected loci. Averaged PfHP1 or H3K9me3 recovery values as determined by qPCR for ten and twelve loci tested negative or positive for PfHP1 in the ChIP-on-chip analysis, respectively (see Figure 5). (B) Inverse correlation between the presence of PfHP1/H3K9me3 and H3K9ac at the same loci. Genes devoid of PfHP1/H3K9me3 are enriched in H3K9ac and vice versa. The average recovery values for PfHP1 and H3K9me3 are compared to those obtained after immunoprecipitation with anti-H3K9ac antibodies. Values represent the mean±s.d. Normal rabbit IgG was used as negative control. The amount of target DNA recovered after immuno-precipitation was directly compared to a ten-fold dilution series of input DNA, and defined as percentage of input.
(0.26 MB PDF)

Figure S6 PfHP1 is not associated with centromeric and pericentromeric chromatin in *P. falciparum*. (A) ChIP-on-chip analyses indicates a significant but low-level enrichment of PfHP1 on centromere-adjacent genes. Average log_2_ ratios of PfHP1-recovered chromatin over input are displayed for all 425 genes with greater than 1.6-fold enrichment (log_2_) (PfHP1 pos.), all genes with recovery values below 1.6-fold enrichment (log_2_) (PfHP1 neg.), and all genes directly up- and downstream of *P. falciparum* centromeres [126] (cen-adj.) (these genes are highlighted in Table S1). (Values represented are the mean±s.d.; p<0.001 in all cases, Wilcoxon ranksum test). (B) Validation of ChIP-on-chip results by targeted ChIP fails to confirm enrichment of PfHP1 at centromeric regions in 3D7/HP1-HA (top panel) and 3D7/HP1-GFP (bottom panel) schizont stage parasites. ChIP-qPCR analysis targeting centromeres on chromosomes 1, 2, 3, 4, 9, 11, 12, 14; and 6 genes directly up- or downstream of the centromeres on chromosomes 1, 5, 6, 8, 12 (centromere-adjacent genes) demonstrates that PfHP1 is not associated with these regions in both independent transgenic cell lines. Two PfHP1-bound genes (PFC0050c and PF11_0007) and two genes not bound by PfHP1 (PFI0265c and PF10_0303 were used as positive (PfHP1-pos.) and negative (PfHP1-neg.) controls, respectively. Anti-H3K9ac and normal rabbit IgG antibodies were used as positive and negative controls for ChIP, respectively. Relative hybridisation intensities (RHI) from the ChIP-on-chip analysis are shown for each gene and indicate log_2_ ratios of recovered chromatin over input. Gene accession numbers are indicated below each graph. Primers used for qPCR are listed in Table S4.
(0.31 MB PDF)

Figure S7 PfHP1 target genes are expressed at lower levels compared to the rest of the coding genome. Absolute transcript levels of all genes (averaged from two replicates A and B from PfHP1-overexpressing lines) were clustered into two groups: PfHP1 ChIP-on-CHIP recovery over input below four (<4×) or greater than four (>4×). Values represent the median±s.d.. The differences in PfHP1-occupancy was significant at all timepoints across the IDC (p<0.001, Wilcoxon ranksum test). Outliers are not plotted. ER, early ring stage; LR, late ring stage; T, trophozoites; S, schizonts; hpi, hours post-invasion.
(0.23 MB PDF)

Table S1 log_2_ ratios of genome-wide PfHP1 ChIP over input obtained by ChIP-on-chip analysis. Gene IDs, chromosomal location and annotation are according to PlasmoDB v5.5 (www.plasmodb.org). Values in column F represent log_2_ ratios of PfHP1-HA-precipitated chromatin over input. Genes with a log_2_ ratio >1.6 are highlighted in red. Column G lists the genes immediately upstream and downstream of mapped *P. falciparum* centromeres [126].
(0.24 MB PDF)

Table S2 425 PfHP1-enriched genes are mostly members of *P. falciparum* lineage-specific gene families coding for exported proteins. The table lists all 425 genes bound by PfHP1 (>1.6 (log_2_) recovery over input). Information contained in columns A-H was retrieved from PlasmoDB v5.5 (www.plasmodb.org). Clustering of genes into the class of predicted exported protein families (columns I and J) was done according to previously published information [3],[89],[90]. Column K lists the log_2_ ratios of PfHP1 ChIP over input for each gene in the list. Column L lists the log_2_ ratios of H3K9me3 ChIP over input for each PfHP1-bound gene reported recently by Salcedo-Amaya and co-workers using the same NimbleGen whole genome microarray (385,000 probes; 48b median spacing) [46]. Genes without H3K9me3-association are highlighted in green. Column M lists the log_2_ ratios of H3K9me3 ChIP over input for each PfHP1-bound gene published by Lopez-Rubio et al. [37]. Lopez-Rubio and colleagues used a NimbleGen platform based on a different probe set containing 145,000 features. Genes without H3K9me3-association are highlighted in bright yellow (not represented on the array) and pale yellow (below threshold).
(0.04 MB PDF)

Table S3 Absolute microarray signal intensities for each gene in two independent PfHP1 over-expressing lines and the control. Median intensity of the microarray signal was extracted for all microarray elements (oligonucleotide spot) for CY5 signal (e.g. total mRNA signal in each time point from the PfHP1 overexpressing lines A and B and the mock transfectant). For genes represented by multiple oligonucleotides [109] the signal was averaged for these microarray elements. The final median value of the spot intensity across the three experiments time courses (overall; median) reflects the overall expression level of each gene in the studied parasite cells. Standard deviation was calculated for each gene intensity profile to estimate the fidelity of the transcript level measurements.
(1.19 MB XLS)

Table S4 Sequences and additional information of oligonucleotides used for qPCR.
(0.04 MB XLS)

Protocol S1 Generation of transfection constructs.
(0.13 MB PDF)

Protocol S2 Nuclear fractionation.
(0.09 MB PDF)

Video S1 3D reconstruction using sequential z-stack series demonstrates the localisation of PfHP1-GFP to perinuclear clusters.
(0.82 MB MOV)
