# Supplementary material for: Plasmodium falciparum Heterochromatin Protein 1 Marks Genomic Loci Linked to Phenotypic Variation of Exported Virulence Factors
Source: PLoS Pathog. 2009 Sep 4;5(9):e1000569. doi: 10.1371/journal.ppat.1000569 (PMC2731224; doi:10.1371/journal.ppat.1000569)
Supplement: Figure S1 — Multiple sequence alignment of PfHP1 with chromo (A) and chromoshadow domains (B) of HP1 orthologs from various species. The alignment was performed using ClustalW (www.ebi.ac.uk/clustalw) and manually adjusted according to published structural information [57]–[59],[61]. Amino acids conserved in the CD and CSD are colored green and pink, respectively, and those important in overall domain structure are colored orange. Asterisks above the chromodomain alignment denote residues important for methyl lysine recognition. In the chromoshadow domain, boxed residues are important for homo-dimerisation and the non-polar amino acid indicated by an asterisk has been shown to be essential in this interaction [57]. Residues denoted by a (+) are important for interaction with the semi-conserved PXVXL motif in HP1-interacting proteins. Sp, S. pombe Swi6/HP1 (NP_593449); Dd, D. discoideum HP1α (XP_639321); Nc, N. crassa HP1 (AAR19291); Hs, H. sapiens HP1α (NP_001120793); Mm, M. musculus HP1β (NP_031648); Dm, D. melanogaster Su(var)205 (NP_723361); Ce, C. elegans HP1-like (NP_510199). Amino acid positions within each sequence are indicated to the left and right of the alignment. (0.30 MB PDF) [file ppat.1000569.s001.pdf]

Supplementary Figure 1 Flueck et al

A

HP1 chromo domain

```
        *                      *  *      * *   *
Sp  81  YVVEKVLKHRMARKGGGYEYLLKWEGYDDPSDNTWSSEADCSG...CKQLIEAY 131
Dd  22  FEVEKILDKRVQH..GRIQYSIRWKGFTA.DYDTWEDEDNVAG...CPELVKEF 69
Nc  76  YVVEKILDHMLNDD.NEPLFLVKWEGYEKKSDQTWEPEDTLIE..GASERLKEY 126
Hs  20  YVVEKVLDRRVVK..GQVEYLLKWKGFSE.EHNTWEPEKNLD....CPELISEF 66
Mm  21  YAVEKIIDRRVRK..GKVEYYLKWKGYPE.TENTWEPENNLD....CQDLIQQY 67
Dm  24  YVVEKVLDRRVVK..GKVEYLLKWKGFSD.EDNTWEPEENLD....CPDLIAEF 70
Ce  37  FVVEKVLNKRLTR..GGSEYYIKWQGFPE.SECSWEPIENLQ....CDRMIQEY 83
Pf  8   FEIGDILEIKKKK..NGFIYLVKWKGYSD.DENTWEPESNLIHLTTFKKKMESL 58
```

B

HP1 chromoshadow domain

```
                                                                                 *  ++   ++ +
Sp  273 VSSIDTIERKD.DGTLEIYLT...WK....................N..GAISH.HPSTITNKKC....PQKMLQ.FY.ESHLTF 324
Dd  165 VEEI..IGCKP..GQDNTLFFFVKWR....................GQ.EKLSW.VLNEILKHKE....PLQLIE.FY.ENRLKF 218
Nc  199 IAQLDACEDED.THKLMVYLT...WK....................N..GHKTQ.HTTDVIYKRC....PQKMLQ.FY.ERHVRI 250
Hs  123 PEKI..IGATDSCGDLMFLMK...WK....................DT.DEADL.VLAKEANVKC....PQIVIA.FY.EERLTW 174
Mm  119 PERI..IGATDSSGELMFLMK...WK....................NS.DEADL.VPAKEANVKC....PQVVIS.FY.EERLTW 170
Dm  149 AEKI..LGASDNNGRLTFLIQ...FK....................GV.DQAEM.VPSSVANEKI....PRMVIH.FY.EERLSW 200
Ce  122 LKTI..IGITKAPGELHFLCK...FS....................D..DSVHL.IPLREANVRF....PSQVIK.FY.ETRLVL 172
Pf  178 VEE.TNIRRT...GHLNIKVND..FKRYVRRKKSSRGNRIVIKNLHNVGDELYISVIHN.INNKEIHSLYPSKVIEYIYPQELLNF 256
```
