# Supplementary figures and images for: Plasmodium falciparum Heterochromatin Protein 1 Marks Genomic Loci Linked to Phenotypic Variation of Exported Virulence Factors
Source: PLoS Pathog. 2009 Sep 4;5(9):e1000569. doi: 10.1371/journal.ppat.1000569 (PMC2731224; doi:10.1371/journal.ppat.1000569)

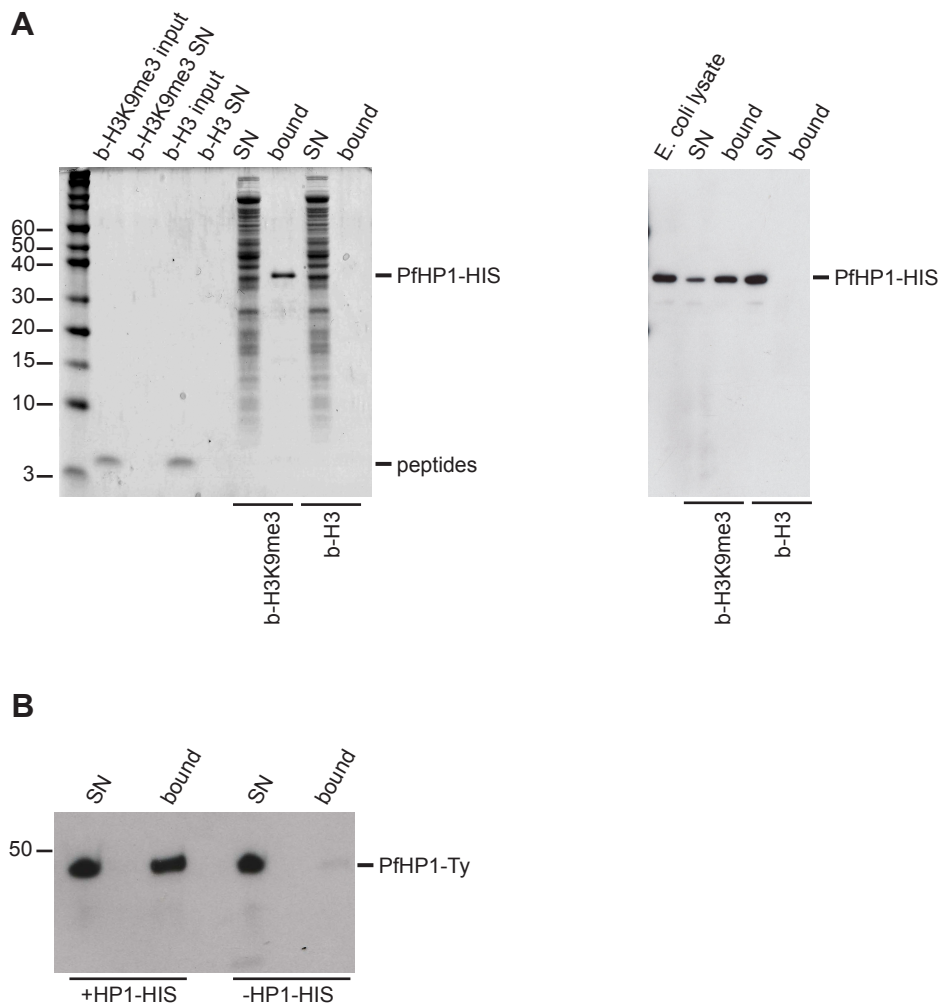

Supplement: Figure S2 — PfHP1-specific binding to H3K9me3 and PfHP1 homo-dimerisation. (A) Recombinant PfHP1 binds specifically to H3K9me3. Left panel: Coomassie-stained gel demonstrating the efficient coupling of biotinylated histone peptides (b-H3K9me3 and b-H3) to streptavidin agarose beads (lanes 2–5) and the specific pull-down of PfHP1-HIS from E. coli lysates with H3K9me3 (lane 7) but not with unmodified H3 peptide (lane 9). Right panel: Western blot using anti-6×HIS antibodies of the input, supernatant and bound fractions from the pull-down experiment confirm the specific binding of PfHP1 to H3K9me3. (B) Homo-dimerisation of PfHP1. Anti-Ty Western blot showing that PfHP1-HIS efficiently purifies PfHP1-Ty from parasite nuclear extracts. (2.05 MB PDF) [file ppat.1000569.s002.pdf]

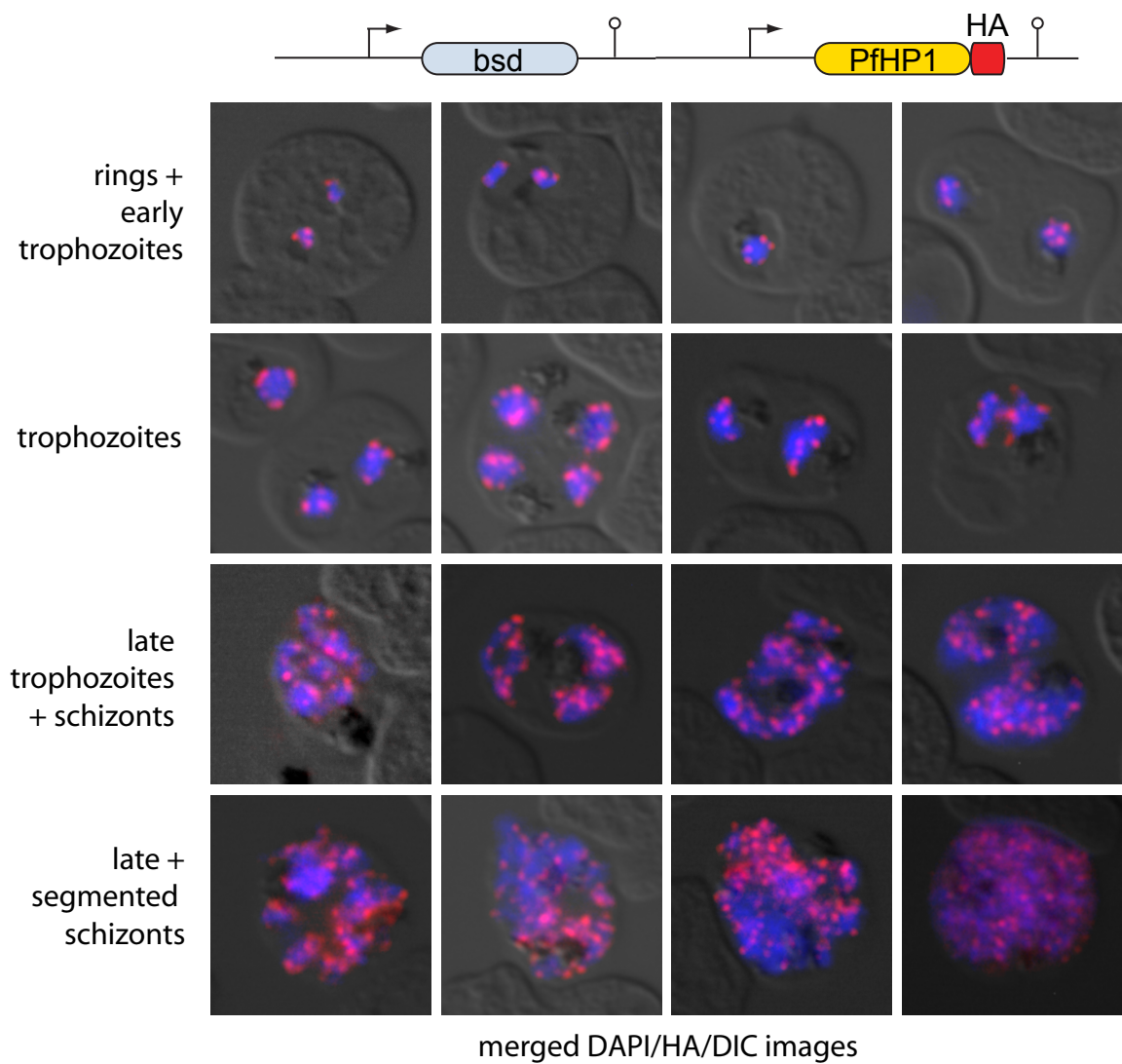

Supplement: Figure S3 — PfHP1 localization across intra-erythrocytic development of P. falciparum. Merged DAPI/anti-HA/DIC images of methanol-fixed 3D7/HP1-HA parasites from early ring stages to segmented schizonts demonstrates a punctate perinuclear localization of PfHP1 throughout the IDC. A schematic of the transfection construct is shown on top. (4.86 MB PDF) [file ppat.1000569.s003.pdf]

A

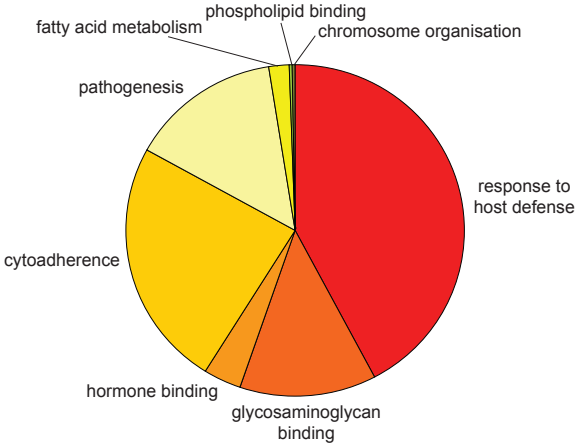

B

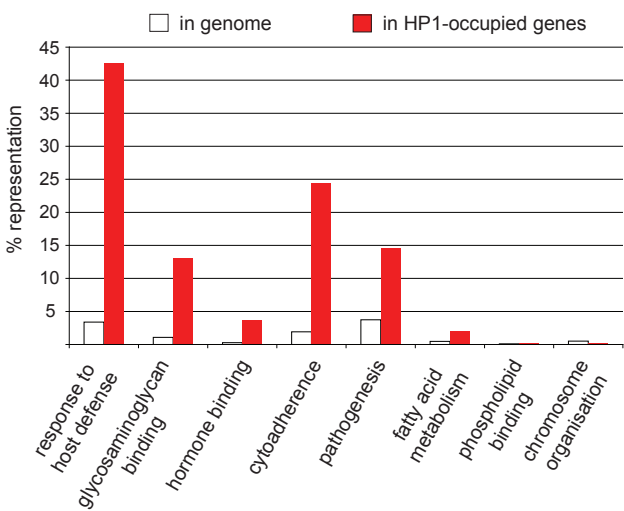

Supplement: Figure S4 — The majority of PfHP1-bound genes are directly involved in host-parasite interactions. (A) Pie chart highlighting the proportion of PfHP1-bound genes falling into annotated/predicted Gene Ontology pathway groups. (B) All PfHP1-occupied genes were clustered according to their annotated/predicted participation in GO pathways (x-axis). The proportion of PfHP1-bound genes in each cluster (red bars) is compared to the proportion of genes in the entire genome falling into the same clusters (white bars). (0.24 MB PDF) [file ppat.1000569.s004.pdf]

**A** HP1  
targeted ChIP

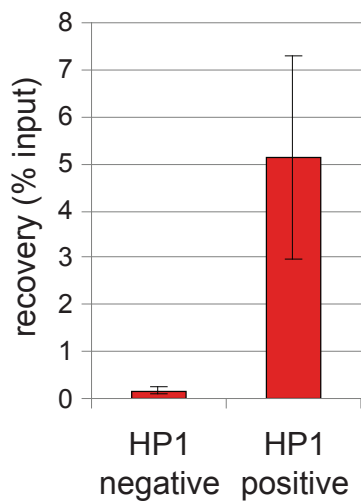

H3K9me3  
targeted ChIP

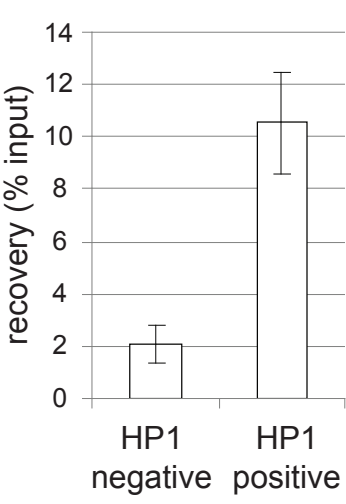

**B**

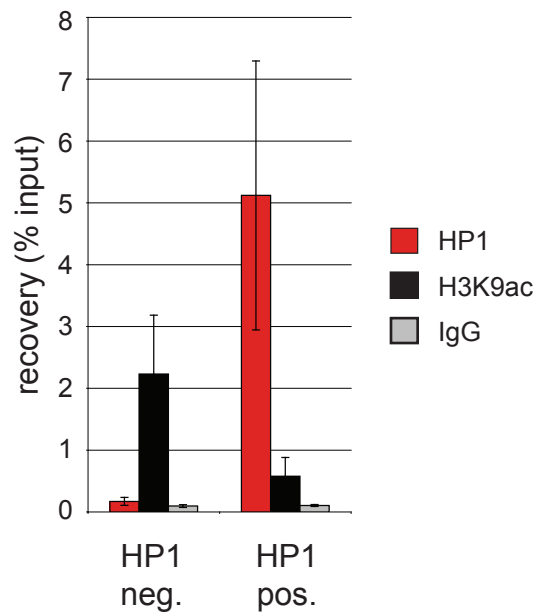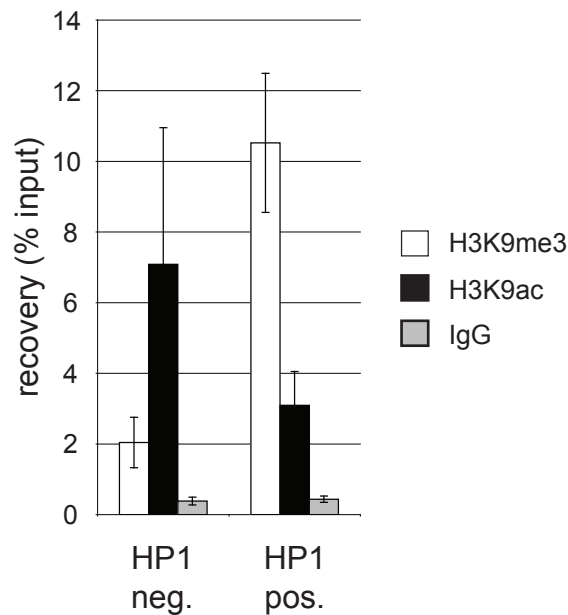

Supplement: Figure S5 — Comparison of average ChIP-qPCR recovery values presented in Figure 5 (validation of genome-wide ChIP results by targeted ChIP). (A) Intimate link between PfHP1-occupancy and the presence of H3K9me3 in chromatin at selected loci. Averaged PfHP1 or H3K9me3 recovery values as determined by qPCR for ten and twelve loci tested negative or positive for PfHP1 in the ChIP-on-chip analysis, respectively (see Figure 5). (B) Inverse correlation between the presence of PfHP1/H3K9me3 and H3K9ac at the same loci. Genes devoid of PfHP1/H3K9me3 are enriched in H3K9ac and vice versa. The average recovery values for PfHP1 and H3K9me3 are compared to those obtained after immunoprecipitation with anti-H3K9ac antibodies. Values represent the mean±s.d. Normal rabbit IgG was used as negative control. The amount of target DNA recovered after immuno-precipitation was directly compared to a ten-fold dilution series of input DNA, and defined as percentage of input. (0.26 MB PDF) [file ppat.1000569.s005.pdf]

A

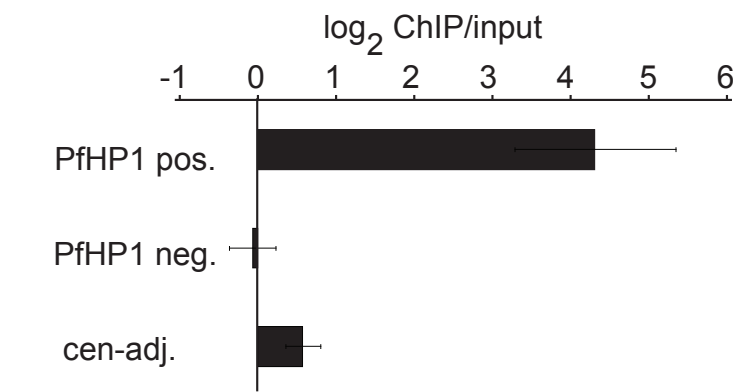

B

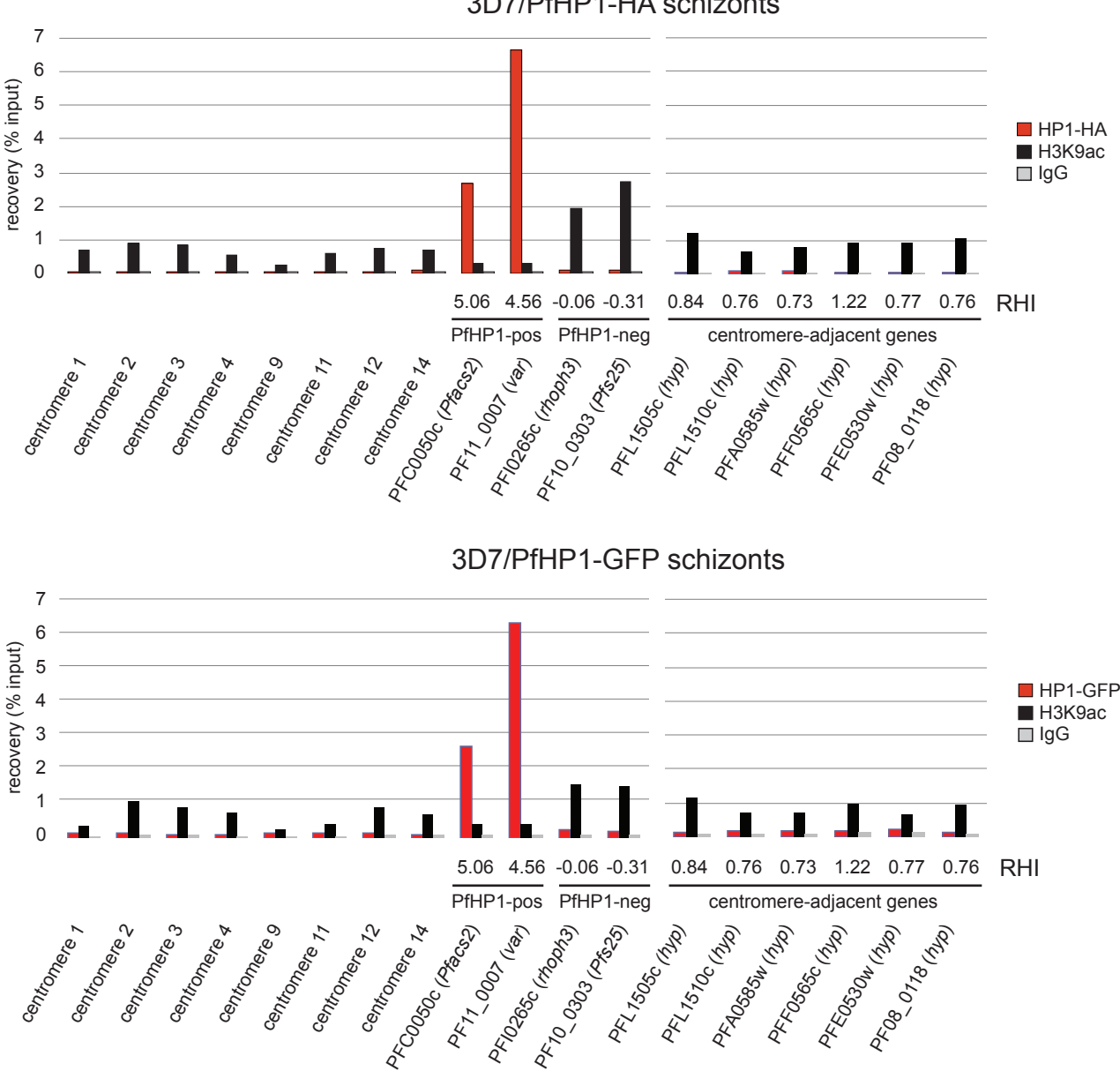

Supplement: Figure S6 — PfHP1 is not associated with centromeric and pericentromeric chromatin in P. falciparum. (A) ChIP-on-chip analyses indicates a significant but low-level enrichment of PfHP1 on centromere-adjacent genes. Average log2 ratios of PfHP1-recovered chromatin over input are displayed for all 425 genes with greater than 1.6-fold enrichment (log2) (PfHP1 pos.), all genes with recovery values below 1.6-fold enrichment (log2) (PfHP1 neg.), and all genes directly up- and downstream of P. falciparum centromeres [126] (cen-adj.) (these genes are highlighted in Table S1). (Values represented are the mean±s.d.; p<0.001 in all cases, Wilcoxon ranksum test). (B) Validation of ChIP-on-chip results by targeted ChIP fails to confirm enrichment of PfHP1 at centromeric regions in 3D7/HP1-HA (top panel) and 3D7/HP1-GFP (bottom panel) schizont stage parasites. ChIP-qPCR analysis targeting centromeres on chromosomes 1, 2, 3, 4, 9, 11, 12, 14; and 6 genes directly up- or downstream of the centromeres on chromosomes 1, 5, 6, 8, 12 (centromere-adjacent genes) demonstrates that PfHP1 is not associated with these regions in both independent transgenic cell lines. Two PfHP1-bound genes (PFC0050c and PF11_0007) and two genes not bound by PfHP1 (PFI0265c and PF10_0303 were used as positive (PfHP1-pos.) and negative (PfHP1-neg.) controls, respectively. Anti-H3K9ac and normal rabbit IgG antibodies were used as positive and negative controls for ChIP, respectively. Relative hybridisation intensities (RHI) from the ChIP-on-chip analysis are shown for each gene and indicate log2 ratios of recovered chromatin over input. Gene accession numbers are indicated below each graph. Primers used for qPCR are listed in Table S4. (0.31 MB PDF) [file ppat.1000569.s006.pdf]

Supplementary Figure 7 Flueck et al

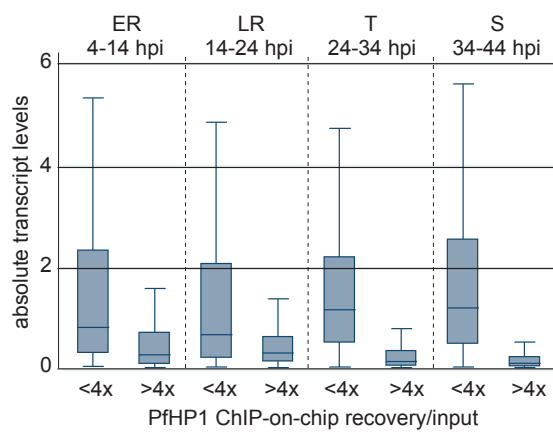

Supplement: Figure S7 — PfHP1 target genes are expressed at lower levels compared to the rest of the coding genome. Absolute transcript levels of all genes (averaged from two replicates A and B from PfHP1-overexpressing lines) were clustered into two groups: PfHP1 ChIP-on-CHIP recovery over input below four (<4×) or greater than four (>4×). Values represent the median±s.d.. The differences in PfHP1-occupancy was significant at all timepoints across the IDC (p<0.001, Wilcoxon ranksum test). Outliers are not plotted. ER, early ring stage; LR, late ring stage; T, trophozoites; S, schizonts; hpi, hours post-invasion. (0.23 MB PDF) [file ppat.1000569.s007.pdf]
