# Supplementary material for: Plasmodium falciparum Heterochromatin Protein 1 Marks Genomic Loci Linked to Phenotypic Variation of Exported Virulence Factors
Source: PLoS Pathog. 2009 Sep 4;5(9):e1000569. doi: 10.1371/journal.ppat.1000569 (PMC2731224; doi:10.1371/journal.ppat.1000569)
Supplement: Table S1 — log2 ratios of genome-wide PfHP1 ChIP over input obtained by ChIP-on-chip analysis. Gene IDs, chromosomal location and annotation are according to PlasmoDB v5.5 (www.plasmodb.org). Values in column F represent log2 ratios of PfHP1-HA-precipitated chromatin over input. Genes with a log2 ratio >1.6 are highlighted in red. Column G lists the genes immediately upstream and downstream of mapped P. falciparum centromeres [126]. (0.24 MB PDF) [file ppat.1000569.s008.pdf]

Chr	Start	End	Gene ID	PlasmoDB reannotation v5.5	log2 ratio PfHP1/input	centromere adjacent genes
chr1	29733	37349	PFA0005w	erythrocyte membrane protein 1 %28PfEMP1%29	4.648	
chr1	39205	40430	PFA0010c	rifin	3.763	
chr1	42590	46730	PFA0015c	var-like protein	5.042	
chr1	50586	51859	PFA0020w	rifin	3.721	
chr1	54001	55229	PFA0030c	rifin	4.398	
chr1	60005	61236	PFA0040w	rifin	5.190	
chr1	62420	63633	PFA0045c	rifin	5.355	
chr1	66050	67222	PFA0050c	rifin	5.214	
chr1	74796	75599	PFA0065w	Plasmdoium falciparum Maurer%27s Cleft 2 transmembr	4.980	
chr1	76215	77042	PFA0070c	hypothetical protein pseudogene	4.500	
chr1	81998	83339	PFA0080c	rifin	4.155	
chr1	85024	86385	PFA0085c	erythrocyte membrane protein 1 %28PfEMP1%29 pseud	5.384	
chr1	87436	88410	PFA0090c	stevor	5.430	
chr1	90475	91653	PFA0095c	rifin	5.051	
chr1	93346	94066	PFA0100c	hypothetical protein conserved in P. falciparum	4.692	
chr1	95257	96046	PFA0105w	stevor	4.366	
chr1	99052	102515	PFA0110w	ring-infected erythrocyte surface antigen	0.836	
chr1	104936	105441	PFA0115w	hypothetical protein	0.285	
chr1	107429	108580	PFA0120c	hydrolase putative	0.561	
chr1	110984	116033	PFA0125c	erythrocyte binding antigen-181	1.174	
chr1	119275	121648	PFA0130c	Serine%2FThreonine protein kinase FIKK family	-0.144	
chr1	124752	125719	PFA0135w	Merozoite-associated tryptophan-rich antigen putative	-0.207	
chr1	126553	128375	PFA0140c	hypothetical protein conserved	0.303	
chr1	129194	131074	PFA0145c	aspartyl-tRNA synthetase	-0.222	
chr1	132320	133858	PFA0150c	hypothetical protein conserved	-0.008	
chr1	134587	139491	PFA0155c	hypothetical protein conserved	0.018	
chr1	140896	142453	PFA0160c	nucleoside transporter	-0.113	
chr1	143396	146866	PFA0165c	hypothetical protein conserved	-0.111	
chr1	148149	153011	PFA0170c	zinc carboxy peptidase putative	-0.203	
chr1	154410	160660	PFA0175w	hypothetical protein conserved	-0.037	
chr1	161365	166464	PFA0180w	hypothetical protein conserved	-0.098	
chr1	166748	168632	PFA0185w	hypothetical protein conserved	0.152	

chr1	169314	171508	PFA0190c	actin-related protein ARP1	-0.510
chr1	173099	174826	PFA0195w	hypothetical protein conserved	-0.214
chr1	175844	176611	PFA0200w	hypothetical protein conserved	0.119
chr1	178322	181025	PFA0205w	hypothetical protein	-0.064
chr1	183057	184457	PFA0210c	hypothetical protein conserved	-0.136
chr1	190507	197586	PFA0215w	hypothetical protein conserved	0.020
chr1	197855	201468	PFA0220w	ubiquitin carboxyl-terminal hydrolase putative	-0.287
chr1	202774	204381	PFA0225w	LytB protein	-0.519
chr1	204860	205627	PFA0230c	hypthetical protein conserved	-0.339
chr1	206408	210963	PFA0235w	hypthetical protein conserved	0.158
chr1	211108	213243	PFA0240w	novel putative transporter PfNPT	-0.171
chr1	216014	217747	PFA0245w	novel putative transporter PfNPT	-0.469
chr1	221440	221871	PFA0250w	hypothetical protein conserved	0.148
chr1	222492	224198	PFA0255c	hypothetical protein conserved	-0.194
chr1	225348	226017	PFA0260c	hypothetical protein c onserved	0.021
chr1	226919	227523	PFA0265c	hypothetical protein conserved	0.039
chr1	228724	229758	PFA0270c	hypothetical protein conserved	-0.138
chr1	230528	234641	PFA0275c	hypothetical protein	0.112
chr1	238905	248096	PFA0280w	asparagine-rich antigen Pfa35-2	-0.345
chr1	249470	252249	PFA0285c	hypothetical protein conserved	-0.190
chr1	253399	255169	PFA0290w	DNA binding protein putative	-0.078
chr1	256014	258987	PFA0295c	hypothetical protein conserved	0.048
chr1	260897	262048	PFA0300c	vacuolar ATP synthase putative	-0.231
chr1	263579	264462	PFA0305c	hypothetical protein conserved	0.007
chr1	265447	269412	PFA0310c	calcium-transporting ATPase	-0.338
chr1	272693	273641	PFA0315w	hypothetical protein conserved	-0.043
chr1	274530	278765	PFA0320w	hypothetical protein conserved	-0.110
chr1	279330	280229	PFA0325w	hypothetical protein	-0.056
chr1	281370	285674	PFA0330w	pfAARP2 protein	-0.224
chr1	287167	289134	PFA0335w	Rab5c GTPase	0.008
chr1	290602	292806	PFA0340w	2-C-methyl-D-erythritol 4-phosphate cytidylyltransferase	0.154
chr1	293860	295186	PFA0345w	centrin putative	0.061
chr1	295518	297149	PFA0350c	hypothetical protein conserved	0.189
chr1	297429	299611	PFA0350w	hypothetical protein conserved	-0.017
chr1	297580	299611	PFA0355w	carbon catabolite repressor protein 4 putative	-0.030

chr1	299776	301082	PFA0360c	hypothetical protein conserved in P. falciparum	0.023
chr1	304091	304552	PFA0370w	hypothetical protein conserved	-0.546
chr1	305516	310411	PFA0375c	lipid%2Fsterol%3AH%2B symporter	-0.197
chr1	315774	320559	PFA0380w	serine%2Fthreonine protein kinase putative	0.000
chr1	321323	322742	PFA0385w	hypothetical protein conserved	0.283
chr1	323526	327226	PFA0390w	DNA repair exonuclease putative	-0.038
chr1	327551	328207	PFA0395w	conserved Plasmodium protein unknown function	-0.072
chr1	329584	330457	PFA0400c	beta3 proteasome subunit putative	-0.599
chr1	331891	333861	PFA0405w	hypothetical protein conserved	0.010
chr1	338321	344986	PFA0410w	hypothetical protein conserved	-0.238
chr1	345398	347693	PFA0415c	mitochondrial carrier protein putative	0.082
chr1	350323	350862	PFA0420w	hypothetical protein conserved	-0.260
chr1	351148	352779	PFA0425c	hypothetical protein conserved	-0.161
chr1	353467	357364	PFA0430c	hypothetical protein conserved	-0.110
chr1	358987	361492	PFA0435w	hypothetical protein conserved	0.252
chr1	364471	365145	PFA0440w	hypothetical protein conserved	-0.389
chr1	366763	369948	PFA0445w	hypothetical protein conserved	-0.111
chr1	370633	371331	PFA0450c	mRNA cleavage factor-like protein putative	-0.385
chr1	372968	374165	PFA0455c	integral membrane protein GNS1%2FSUR4 family puta	-0.064
chr1	375592	376240	PFA0460c	tubulin-specific chaperone a putative	0.133
chr1	377447	378157	PFA0465c	N-terminal acetyltransferase putative	-0.248
chr1	379027	379752	PFA0470c	cold-shock protein putative	0.105
chr1	380234	380960	PFA0475c	hypothetical protein conserved	0.220
chr1	381851	383578	PFA0480w	phenylalanyl-tRNA synthetase beta chain putative	-0.148
chr1	384396	389094	PFA0485w	dolichol kinase	0.150
chr1	389526	390230	PFA0490w	hypothetical protein conserved	-0.478
chr1	390415	393219	PFA0495c	selenocysteine-specific elongation factor selB homologue	-0.129
chr1	393758	394189	PFA0500w	human hepatopoietin-like protein putative	0.159
chr1	395029	395989	PFA0505c	DNA-directed RNA polymerase 2 subunit putative	-0.467
chr1	397745	404760	PFA0510w	hypothetical protein conserved	0.173
chr1	406893	413323	PFA0515w	phosphatidylinositol-4-phosphate 5-kinase putative	-0.093
chr1	414914	416254	PFA0520c	chromatin assembly factor 1 protein WD40 domain puta	-0.453
chr1	419075	420761	PFA0525w	transcription initiation factor TFIIB putative	-0.263
chr1	421447	422007	PFA0530c	adenylate kinase putative	-0.218
chr1	422930	428729	PFA0535c	kinesin putative	-0.117

chr1	431305	432011	PFA0540c	hypothetical protein conserved	0.479	
chr1	432691	436194	PFA0545c	replication factor c protein putative	0.173	
chr1	437026	439716	PFA0550w	hypothetical protein conserved	0.224	
chr1	440804	442570	PFA0555c	UMP-CMP kinase putative	0.142	
chr1	443695	444441	PFA0560c	hypothetical protein conserved	0.495	
chr1	444737	445090	PFA0565c	hypothetical protein conserved	0.674	
chr1	447177	450433	PFA0570w	hypothetical protein conserved	0.229	
chr1	451193	451796	PFA0575c	hypothetical protein conserved	0.077	
chr1	452384	454268	PFA0580c	TatD-like deoxyribonuclease putative	0.331	
chr1	455997	456488	PFA0585w	hypothetical protein conserved	0.730	1cenL
chr1	465876	471344	PFA0590w	ABC transporter%28CT family%29 PfMRP	0.506	1cenR
chr1	474888	477036	MAL1_18s	18s rRNA A-type	1.300	
chr1	477037	477430	MAL1_ITS1	ITS1 A-type	1.553	
chr1	477431	477551	MAL1_5.8s	5.8s rRNA	2.330	
chr1	477552	478427	MAL1_ITS2	ITS2 A-type	2.165	
chr1	478428	482531	MAL1_28s	28s rRNA %28A-type%29	1.699	
chr1	482958	483767	PFA0610c	hypothetical protein conserved in P. falciparum	1.477	
chr1	487649	488335	PFA0615w	hypothetical protein	0.318	
chr1	489041	491276	PFA0620c	glutamic acid-rich protein %28garp%29	0.456	
chr1	496393	501225	PFA0625w	surface-associated interspersed gene %28SURFIN%29	1.895	
chr1	502108	504017	PFA0630c	hypothetical protein	3.455	
chr1	506087	508023	PFA0635c	hypothetical protein	1.554	
chr1	509401	510897	PFA0640c	hypothetical protein	0.228	
chr1	511710	511955	PFA0645c	hypothetical protein	0.607	
chr1	513235	519004	PFA0650w	surface-associated interspersed gene pseudogene %28	1.753	
chr1	522529	524301	PFA0660w	protein with DNAJ domain dnj1%2Fsis1 family	1.063	
chr1	528829	538073	PFA0665w	hypothetical protein conserved in P. falciparum	4.965	
chr1	538831	539747	PFA0670c	hypothetical protein conserved in P. falciparum	0.327	
chr1	544202	548702	PFA0675w	P. falciparum RESA-like protein with DnaJ domain	3.111	
chr1	549319	550102	PFA0680c	Plasmodium falcioparum maurer%27s cleft 2 transmemb	4.525	
chr1	552298	553435	PFA0685c	Plasmodium falciparum Maurer%27s Cleft 2 transmembr	4.076	
chr1	554362	555519	PFA0690w	hypothetical protein conserved in P. falciparum pseudog	4.228	
chr1	555770	556651	PFA0695c	erythrocyte membrane protein 1 %28PfEMP1%29-like p	3.871	
chr1	559923	560430	PFA0700c	hypothetical protein conserved in P. falciparum	3.594	
chr1	562299	563256	PFA0705c	stevor pseudogene putative	4.923	

chr1	565425	566564	PFA0710c	rifin	4.787
chr1	571206	571993	PFA0715c	hypothetical protein conserved in P. falciparum	3.380
chr1	573708	574404	PFA0720w	hypothetical protein	3.381
chr1	579424	585509	PFA0725w	surface-associated interspersed gene %28SURFIN%29	3.434
chr1	589111	590114	PFA0735w	hypothetical protein conserved in P.falciparum	2.887
chr1	593569	594907	PFA0740w	rifin	4.843
chr1	600185	601192	PFA0750w	stevor	5.223
chr1	602412	602844	PFA0755w	erythrocyte membrane protein 1 %28PfEMP1%29 pseud	5.127
chr1	605882	607251	PFA0760w	rifin	4.148
chr1	609110	616613	PFA0765c	erythrocyte membrane protein 1 %28PfEMP1%29	4.815
chr10	28491	36165	PF10_0001	erythrocyte membrane protein 1 %28PfEMP1%29	4.711
chr10	38051	39004	PF10_0002	rifin	3.504
chr10	41774	42964	PF10_0003	rifin	4.850
chr10	44817	46134	PF10_0004	rifin	5.036
chr10	48053	50279	PF10_0005	rifin	4.489
chr10	51350	52600	PF10_0006	rifin	4.040
chr10	54543	54770	PF10_0007	hypothetical protein	3.043
chr10	55124	55508	PF10_0008	hypothetical protein	3.152
chr10	57969	58981	PF10_0009	pseudogene stevor putative	5.330
chr10	60068	60777	PF10_0011	erythrocyte membrane protein 1 %28PfEMP1%29 trunc	3.722
chr10	60889	61366	PF10_0012	erythrocyte membrane protein 1 %28PfEMP1%29 trunc	2.630
chr10	63153	64033	PF10_0013	hypothetical protein	0.256
chr10	67808	68389	PF10_0014	hypothetical protein	0.823
chr10	68690	68962	PF10_0015	acyl CoA binding protein putative	-0.123
chr10	70548	70820	PF10_0016	acyl CoA binding protein putative	0.318
chr10	72935	73918	PF10_0017	hypothetical protein	0.300
chr10	74662	77573	PF10_0018	hypothetical protein	0.082
chr10	81417	81740	PF10_0019	early transcribed membrane protein 10.1 etramp 10.1	-0.563
chr10	86539	89010	PF10_0020	hypothetical protein	0.006
chr10	92574	93527	PF10_0021	hypothetical protein	0.290
chr10	96114	96563	PF10_0022	hypothetical protein	-0.012
chr10	99380	100362	PF10_0023	hypothetical protein	0.126
chr10	103119	104684	PF10_0024	hypothetical protein	0.340
chr10	106654	108663	PF10_0025	PF70 protein	-0.261
chr10	112930	116016	PF10_0026	Tryptophan-rich antigen 3 putative	-0.277

chr10	117243	118654	PF10_0027	hypothetical protein	-0.052
chr10	121116	123292	PF10_0028	RNA binding protein putative	-0.032
chr10	124455	124883	PF10_0029	hypothetical protein	-0.199
chr10	125940	127601	PF10_0030	hypothetical protein	0.017
chr10	129277	132111	PF10_0031	hypothetical protein	-0.050
chr10	132996	137273	PF10_0032	DnaJ protein putative	0.066
chr10	138766	139668	PF10_0033	hypothetical protein	0.426
chr10	142420	148240	PF10_0034	hypothetical protein	-0.016
chr10	149235	150039	PF10_0035	hypothetical protein	-0.276
chr10	150417	150875	PF10_0036	N-acetyltransferase putative	-0.194
chr10	152365	158517	PF10_0037	hypothetical protein	0.144
chr10	160317	160963	PF10_0038	ribosomal protein S20e putative	0.090
chr10	164946	165791	PF10_0039	hypothetical protein	-0.386
chr10	166744	170670	PF10_0040	hypothetical protein	-0.323
chr10	172724	176431	PF10_0041	U5 small nuclear ribonuclear protein putative	-0.580
chr10	177922	178871	PF10_0042	hypothetical protein	-0.064
chr10	179345	180227	PF10_0043	ribosomal protein L13 putative	-0.053
chr10	182245	187101	PF10_0044	hypothetical protein	-0.053
chr10	187670	196955	PF10_0045	hypothetical protein	0.001
chr10	199453	202845	PF10_0046	hypothetical protein	-0.416
chr10	207001	209643	PF10_0047	RNA binding protein putative	-0.423
chr10	213987	214595	PF10_0048	hypothetical protein	0.392
chr10	216208	218703	PF10_0049	hypothetical protein	0.046
chr10	220019	221354	PF10_0050	hypothetical protein	0.186
chr10	221946	223431	PF10_0051	ADP%2FATP carrier protein putative	-0.650
chr10	224249	225208	PF10_0052	hypothetical protein	-0.242
chr10	225457	227706	PF10_0053	tRNA ligase putative	-0.359
chr10	228799	232194	PF10_0054	hypothetical protein	0.226
chr10	232423	232819	PF10_0054a	conserved Plasmodium protein unknown function	0.320
chr10	233286	234474	PF10_0055	hypothetical protein	0.271
chr10	234796	235746	PF10_0056	hypothetical protein	0.281
chr10	237507	242245	PF10_0057	regulator of nonsense transcripts putative	-0.551
chr10	244145	249940	PF10_0058	DnaJ protein putative	-0.128
chr10	251579	252490	PF10_0059	hypothetical protein	-0.101
chr10	253704	255611	PF10_0060	hypothetical protein	-0.612

chr10	256117	257699	PF10_0061	hypothetical protein	-0.267
chr10	259060	260662	PF10_0062	NOT protein putative	0.315
chr10	262150	262997	PF10_0063	DNA%2FRNA-binding protein putative	-0.279
chr10	265883	266485	PF10_0063a	PF10_0063a	-0.327
chr10	267219	269831	PF10_0064	hypothetical protein	-0.320
chr10	270535	271497	PF10_0065	hypothetical protein	-0.222
chr10	272243	272824	PF10_0066	hypothetical protein	-0.097
chr10	273599	275426	PF10_0067	hypothetical protein	0.101
chr10	277621	279168	PF10_0068	RNA binding protein putative	-0.064
chr10	281459	283104	PF10_0069	PPPDE peptidase putative	-0.170
chr10	285135	286302	PF10_0070	hypothetical protein	-0.293
chr10	288383	290826	PF10_0071	rhoGAP protein	-0.198
chr10	293850	295999	PF10_0072	hypothetical protein	-0.250
chr10	296906	298687	PF10_0073	hypothetical protein	0.326
chr10	299280	299609	PF10_0074	hypothetical protein	-0.453
chr10	301126	305919	PF10_0075	asparagine-rich antigen	-0.362
chr10	310763	314516	PF10_0076	hypothetical protein	0.319
chr10	316649	318280	PF10_0077	eukaryotictranslation initiation factor 3 subunit 7 putative	-0.599
chr10	321011	328150	PF10_0078	histone deacetylase putative	-0.152
chr10	331246	342807	PF10_0079	hypothetical protein	-0.118
chr10	343733	344899	PF10_0080	endonuclease putative	-0.060
chr10	347206	348573	PF10_0081	26S proteasome regulatory subunit 4 putative	-0.725
chr10	351703	355593	PF10_0082	hypothetical protein	-0.238
chr10	357345	358463	PF10_0083	hypothetical protein	-0.496
chr10	360625	362480	PF10_0084	tubulin beta chain putative	-0.819
chr10	365737	367146	PF10_0085	nucleolar protein NOP5 putative	-0.454
chr10	369199	370225	PF10_0086	adenylate kinase putative	-0.716
chr10	371293	372117	PF10_0087	diphthine synthase	-0.578
chr10	372519	374072	PF10_0088	hypothetical protein	-0.164
chr10	374864	377611	PF10_0089	hypothetical protein	-0.450
chr10	377906	379708	PF10_0090	hypothetical protein	-0.129
chr10	381361	382449	PF10_0091	hypothetical protein	-0.752
chr10	383384	384793	PF10_0092	metallopeptidase putative	-0.059
chr10	387027	388438	PF10_0093	hypothetical protein	-0.579
chr10	389503	391164	PF10_0094	tubulin-tyrosine ligase putative	-0.251

chr10	393764	397992	PF10_0095	hypothetical protein	0.214
chr10	399206	401777	PF10_0096	hypothetical protein	-0.076
chr10	401875	402710	PF10_0097	hypothetical protein	-0.110
chr10	403678	404574	PF10_0098	hypothetical protein	-0.269
chr10	405016	410535	PF10_0099	hypothetical protein	-0.267
chr10	414731	415327	PF10_0100	hypothetical protein	0.054
chr10	416252	417470	PF10_0101	hypothetical protein	-0.185
chr10	417704	419142	PF10_0102	Ankyrin repeat protein putative	0.249
chr10	420184	421055	PF10_0103	eukaryotictranslation initiation factor 2 beta putative	-0.519
chr10	422960	424364	PF10_0104	hypothetical protein	-0.348
chr10	424865	425764	PF10_0105	hypothetical protein	-0.355
chr10	426823	428145	PF10_0106	hypothetical protein	0.057
chr10	429203	430139	PF10_0107	hypothetical protein	-0.351
chr10	432504	435569	PF10_0108	hypothetical protein	0.100
chr10	436957	437712	PF10_0109	hypothetical protein	0.229
chr10	438445	440360	PF10_0110	hypothetical protein	0.006
chr10	441139	441954	PF10_0111	20S proteasome beta subunit putative	-0.531
chr10	443873	444400	PF10_0112	hypothetical protein	-0.485
chr10	445163	447124	PF10_0113	hypothetical protein	0.255
chr10	449153	450322	PF10_0114	DNA repair protein RAD23 putative	-0.250
chr10	455397	458816	PF10_0115	QF122 antigen	-0.137
chr10	460334	461992	PF10_0116	hypothetical protein	-0.045
chr10	463492	467005	PF10_0117	hypothetical protein	-0.008
chr10	468070	469535	PF10_0118	hypothetical protein	0.058
chr10	470978	471932	PF10_0119	hypothetical protein	0.139
chr10	472545	473385	PF10_0120	ubiquinol-cytochrome c reductase complex subunit puta	-0.305
chr10	475326	476562	PF10_0121	hypoxanthine phosphoribosyltransferase	-0.343
chr10	478757	480538	PF10_0122	phosphoglucomutase putative	-0.599
chr10	482526	484193	PF10_0123	GMP synthetase	-0.492
chr10	487251	491827	PF10_0124	hypothetical protein	-0.003
chr10	492549	495143	PF10_0125	metabolite%2Fvitamin transporter	0.083
chr10	496330	497572	PF10_0126	hypothetical protein conserved	-0.443
chr10	500209	502220	PF10_0127	hypothetical protein	0.143
chr10	502633	506391	PF10_0128	WD repeat protein putative	0.126
chr10	511083	514277	PF10_0129	hypothetical protein	-0.218

chr10	515720	517606	PF10_0130	hypothetical protein	0.398
chr10	518858	522251	PF10_0131	hypothetical protein	0.226
chr10	526839	531383	PF10_0132	phospholipase C-like putative	0.074
chr10	533108	539824	PF10_0133	hypothetical protein	0.132
chr10	540621	541693	PF10_0134	hypothetical protein	0.124
chr10	542977	544555	PF10_0135	hypothetical protein	-0.148
chr10	545678	548902	PF10_0136	Initiation factor 2 subunit family putative	0.020
chr10	551028	552570	PF10_0137	GDP-fucose synthase putative	-0.094
chr10	555159	559526	PF10_0138	hypothetical protein	0.030
chr10	560537	563352	PF10_0139	hypothetical protein	-0.238
chr10	565804	573059	PF10_0140	hypothetical protein	-0.024
chr10	574128	575102	PF10_0141	cdk7 putative	-0.267
chr10	576578	580477	PF10_0142	hypothetical protein	0.317
chr10	581353	589089	PF10_0143	transcriptional activator ADA2 putative	-0.179
chr10	595307	596221	PF10_0144	prohibitin putative	-0.743
chr10	597431	599419	PF10_0145	hypothetical protein	0.027
chr10	600969	607808	PF10_0146	hypothetical protein	-0.047
chr10	611212	612198	PF10_0147	FAD synthetase putative	-0.185
chr10	612684	613874	PF10_0148	hypothetical protein	-0.079
chr10	614871	616795	PF10_0149	cysteine -- tRNA ligase putative	-0.341
chr10	618439	619992	PF10_0150	methionine aminopeptidase putative	-0.164
chr10	621304	623625	PF10_0151	hypothetical protein	-0.091
chr10	624047	625894	PF10_0152	hypothetical protein	0.049
chr10	627043	629039	PF10_0153	hsp60	-0.672
chr10	633681	635285	PF10_0154	ribonucleotide reductase small subunit putative	-0.067
chr10	637137	639010	PF10_0155	enolase	-0.333
chr10	641389	642750	PF10_0156	hypothetical protein	-0.004
chr10	643557	644330	PF10_0157	hypothetical protein	0.076
chr10	646053	647735	PF10_0158	hypothetical protein	0.199
chr10	650897	653566	PF10_0159	glycophorin-binding protein 130 precursor	0.742
chr10	656708	659026	PF10_0160	3.8 protein	-0.402
chr10	661741	666358	PF10_0161	hypothetical protein	0.064
chr10	669201	671852	PF10_0162	hypothetical protein	-0.131
chr10	675292	676416	PF10_0163	hypothetical protein	0.179
chr10	682181	682507	PF10_0164	early transcribed membrane protein 10.3 etramp10.3	-0.583

chr10	685577	688861	PF10_0165	DNA polymerase delta catalytic subunit	-0.386
chr10	689887	690819	PF10_0166	hypothetical protein	-0.129
chr10	692425	694101	PF10_0167	hypothetical protein	-0.222
chr10	694839	696590	PF10_0168	hypothetical protein	-0.130
chr10	697969	699822	PF10_0169	phosphomannomutase putative	0.107
chr10	701745	702421	PF10_0170	hypothetical protein	-0.259
chr10	705536	708451	PF10_0171	hypothetical protein	0.076
chr10	709506	710825	PF10_0172	hypothetical protein	0.078
chr10	711015	714657	PF10_0173	hypothetical protein	0.009
chr10	715877	717436	PF10_0174	26s proteasome subunit p55 putative	-0.525
chr10	718453	720135	PF10_0175	tRNA pseudouridine synthase putative	-0.335
chr10	720550	721398	PF10_0176	hypothetical protein	0.122
chr10	721733	732855	PF10_0177	erythrocyte membrane-associated antigen	-0.141
chr10	735258	735737	PF10_0178	hypothetical protein	-0.455
chr10	735920	736812	PF10_0179	PHF5-like protein putative	-0.518
chr10	747027	748886	PF10_0180	hypothetical protein	-0.200
chr10	750214	752664	PF10_0181	hypothetical protein	0.076
chr10	752759	754909	PF10_0182	hypothetical protein	-0.138
chr10	757794	765157	PF10_0183	hypothetical protein	-0.227
chr10	766167	771953	PF10_0184	hypothetical protein	0.158
chr10	773584	774489	PF10_0185	hypothetical protein	-0.295
chr10	774928	780486	PF10_0186	hypothetical protein	-0.220
chr10	786502	787007	PF10_0187	ribosomal protein L30e putative	-0.622
chr10	787455	787910	PF10_0410	hypothetical protein	-0.034
chr10	789867	793765	PF10_0188	hypothetical protein	-0.193
chr10	796008	799922	PF10_0189	hypothetical protein	-0.034
chr10	800277	803501	PF10_0190	hypothetical protein	-0.041
chr10	804327	807581	PF10_0191	tRNA methyltransferase putative	0.173
chr10	808617	808991	PF10_0193	Autophagy protein putative	0.151
chr10	811105	813483	PF10_0194	hypothetical protein	-0.033
chr10	814351	819220	PF10_0195	hypothetical protein	0.109
chr10	819829	822138	PF10_0196	cytoplasmic dynein intermediate chain putative	-0.525
chr10	822601	824592	PF10_0197	hypothetical protein	-0.310
chr10	825450	826970	PF10_0198	hypothetical protein	-0.423
chr10	827487	834225	PF10_0199	hypothetical protein	0.164

chr10	835291	839699	PF10_0200	ATPase putative	0.011
chr10	840211	843402	PF10_0407	dihydrolipoamide acetyltransferase putative	-0.153
chr10	845085	846191	PF10_0203	ADP-ribosylation factor	-0.593
chr10	848858	849521	PF10_0204	hypothetical protein	-0.358
chr10	849790	856283	PF10_0205	hypothetical protein	0.202
chr10	857671	859740	PF10_0206	hypothetical protein	-0.103
chr10	861512	863271	PF10_0207	hypothetical protein	0.286
chr10	863905	866740	PF10_0208	endomembrane protein 70 putative	-0.144
chr10	867633	869675	PF10_0209	RNA helicase putative	0.243
chr10	871093	871884	PF10_0210	deoxyribose-phosphate aldolase putative	-0.553
chr10	873206	886845	PF10_0211	hypothetical protein	0.282
chr10	887414	903606	PF10_0212	hypothetical protein	0.041
chr10	905471	912343	PF10_0213	10b antigen putative	0.011
chr10	916709	924212	PF10_0214	RNA binding protein putative	0.090
chr10	925279	928551	PF10_0215	hypothetical protein	0.216
chr10	932696	934079	PF10_0216	hypothetical protein	0.514
chr10	939936	942232	PF10_0217	pre-mRNA splicing factor putative	0.301
chr10	944396	946087	PF10_0218	citrate synthase mitochondrial precursor putative	-0.011
chr10	948475	951150	PF10_0219	hypothetical protein	0.211
chr10	951750	952577	PF10_0220	hypothetical protein	-0.274
chr10	955881	958355	PF10_0221	GcpE protein	-0.370
chr10	958937	959894	PF10_0222	hypothetical protein	0.133
chr10	961064	962305	PF10_0223	hypothetical protein	0.061
chr10	963495	980558	PF10_0224	dynein heavy chain putative	0.240
chr10	982229	983200	PF10_0225	orotidine-monophosphate-decarboxylase putative	-0.302
chr10	984390	985328	PF10_0226	hypothetical protein conserved	-0.138
chr10	985348	986849	PF10_0227	HORMA domain protein putative	0.009
chr10	987640	988874	PF10_0228	hypothetical protein conserved	0.196
chr10	988941	989790	PF10_0229	hypothetical protein conserved	0.696
chr10	991145	993418	PF10_0230	hypothetical protein conserved	-0.049
chr10	994320	996786	PF10_0231	hypothetical protein	0.008
chr10	997473	1007459	PF10_0232	Chromodomain-helicase-DNA-binding protein 1 homolog	-0.242
chr10	1014939	1016594	PF10_0233	hypothetical protein	-0.220
chr10	1017199	1020513	PF10_0234	hypothetical protein	0.164
chr10	1021290	1021772	PF10_0235	RNA binding protein putative	-0.525

chr10	1022503	1022895	PF10_0236	hypothetical protein	-0.699
chr10	1023203	1024867	PF10_0237	hypothetical protein	0.029
chr10	1026118	1026912	PF10_0238	hypothetical protein	-0.223
chr10	1027464	1027863	PF10_0239	hypothetical protein	0.272
chr10	1028149	1029365	PF10_0240	hypothetical protein	0.332
chr10	1030042	1031532	PF10_0241	hypothetical protein	-0.305
chr10	1033678	1039077	PF10_0242	hypothetical protein	-0.355
chr10	1040287	1045002	PF10_0243	hypothetical protein	0.167
chr10	1045606	1049758	PF10_0244	hypothetical protein	0.038
chr10	1052842	1055331	PF10_0245	glucosamine--fructose-6-phosphate aminotransferase pu	-0.431
chr10	1055742	1060656	PF10_0246	hypothetical protein	0.137
chr10	1061397	1063949	PF10_0247	hypothetical protein	-0.195
chr10	1065344	1067083	PF10_0248	hypothetical protein	0.002
chr10	1067845	1068779	PF10_0249	hypothetical protein	0.155
chr10	1069222	1073322	PF10_0250	hypothetical protein	0.255
chr10	1073539	1086990	PF10_0251	hypothetical protein	0.169
chr10	1088212	1088415	PF10_0252	Cytochrome C oxidase copper chaperone putative	-0.204
chr10	1088929	1089492	PF10_0253	hypothetical protein	-0.655
chr10	1091434	1093853	PF10_0254	hypothetical protein	-0.168
chr10	1094393	1094650	PF10_0255	hypothetical protein	-0.207
chr10	1095002	1097275	PF10_0256	hypothetical protein	-0.196
chr10	1098453	1099880	PF10_0257	hypothetical protein	0.199
chr10	1101816	1103156	PF10_0258	hypothetical protein	0.236
chr10	1103462	1105228	PF10_0259	hypothetical protein	-0.149
chr10	1105449	1109449	PF10_0260	hypothetical protein	-0.192
chr10	1109792	1111603	PF10_0261	WD repeat protein putative	-0.129
chr10	1113493	1118571	PF10_0262	hypothetical protein	-0.096
chr10	1121637	1121831	PF10_0263	hypothetical protein	0.266
chr10	1122960	1124035	PF10_0264	40S ribosomal protein putative	-0.746
chr10	1125281	1126162	PF10_0409	biotin--acetyl-CoA-carboxylase putative	-0.434
chr10	1126521	1131956	PF10_0265	hypothetical protein	0.373
chr10	1133836	1136154	PF10_0266	hypothetical protein	0.049
chr10	1138000	1139562	PF10_0267	hypothetical protein	-0.263
chr10	1140587	1142600	PF10_0268	merozoite capping protein 1	-0.149
chr10	1145821	1146793	PF10_0269	DNA-directed RNA polymerase II putative	-0.536

chr10	1147864	1148211	PF10_0270	hypothetical protein	0.161
chr10	1148314	1150166	PF10_0412	topoisomerase putative	-0.248
chr10	1150661	1151851	PF10_0271	centrin putative	-0.225
chr10	1153269	1154429	PF10_0272	ribosomal protein L3 putative	-0.753
chr10	1156646	1158978	PF10_0273	DHHC-type zinc finger protein putative	0.004
chr10	1160353	1163419	PF10_0274	hypothetical protein	0.002
chr10	1163784	1165613	PF10_0275	protoporphyrinogen oxidase putative	-0.069
chr10	1166044	1166868	PF10_0276	hypothetical protein	-0.337
chr10	1168008	1168955	PF10_0277	hypothetical protein conserved	0.179
chr10	1169938	1171389	PF10_0278	BRIX domain containig protein putative	-0.323
chr10	1172127	1174995	PF10_0279	hypothetical protein	-0.146
chr10	1176459	1177847	PF10_0280	hypothetical protein	0.013
chr10	1181003	1182499	PF10_0281	hypothetical protein	-0.454
chr10	1183736	1183993	PF10_0282	hypothetical protein	-0.281
chr10	1184726	1185316	PF10_0283	hypothetical protein	-0.470
chr10	1190424	1195014	PF10_0284	hypothetical protein	0.013
chr10	1195675	1197342	PF10_0285	hypothetical protein	-0.041
chr10	1197823	1200030	PF10_0286	hypothetical protein	-0.088
chr10	1200892	1202397	PF10_0287	hypothetical protein	-0.088
chr10	1204344	1204760	PF10_0288	hypothetical protein	0.218
chr10	1206300	1207403	PF10_0289	adenosine deaminase putative	-0.285
chr10	1208196	1210561	PF10_0290	hypothetical protein	0.751
chr10	1211656	1212969	PF10_0291	RAP protein putative	-0.127
chr10	1217346	1222439	PF10_0292	hypothetical protein	-0.244
chr10	1223373	1224165	PF10_0293	transcription factor putative	-0.165
chr10	1224517	1229090	PF10_0294	RNA helicase putative	-0.447
chr10	1230559	1231839	PF10_0295	hypothetical protein	-0.923
chr10	1233665	1237000	PF10_0296	hypothetical protein	-0.090
chr10	1241765	1242975	PF10_0297	hypothetical protein	0.014
chr10	1244213	1245394	PF10_0298	26S proteasome subunit putative	-0.054
chr10	1245878	1247674	PF10_0299	glycoprotease putative	-0.275
chr10	1248531	1249304	PF10_0300	RNA methyltransferase putative	0.048
chr10	1250043	1250681	PF10_0301	calmodulin putative	0.486
chr10	1251149	1251805	PF10_0302	ookinete surface protein Pos28-1 putative	-0.484
chr10	1253415	1254068	PF10_0303	25 kDa ookinete surface antigen precursor %28pfs25%2	-0.305

chr10	1254841	1255065	PF10_0304	hypothetical protein	0.146
chr10	1255405	1255584	PF10_0305	hypothetical protein	0.846
chr10	1257382	1259309	PF10_0306	MORN repeat containing protein	-0.042
chr10	1260308	1263067	PF10_0307	hypothetical protein	-0.031
chr10	1264972	1268783	PF10_0308	OTU-like cysteine protease putative	-0.061
chr10	1270526	1274142	PF10_0309	DEAD%2FDEAH box helicase putative	-0.148
chr10	1275861	1278575	PF10_0310	hypothetical protein	-0.144
chr10	1281250	1281973	PF10_0311	protein phosphatase inhibitor putative	-0.363
chr10	1282403	1284055	PF10_0312	hypothetical protein	-0.109
chr10	1285057	1289965	PF10_0313	hypothetical protein	-0.166
chr10	1293497	1297069	PF10_0314	asparagine-rich antigen	-0.367
chr10	1299792	1302407	PF10_0315	hypothetical protein	0.044
chr10	1302561	1304879	PF10_0316	phosphatidyl inositol glycan class A putative	-0.182
chr10	1306239	1307530	PF10_0317	Der1-like family putative	-0.517
chr10	1308871	1309923	PF10_0318	hypothetical protein conserved	0.194
chr10	1310419	1312080	PF10_0319	hypothetical protein	-0.003
chr10	1313253	1320947	PF10_0320	leucine-rich repeat protein 8 LRR8	0.189
chr10	1321692	1323377	PF10_0321	hypothetical protein	-0.556
chr10	1324130	1328434	PF10_0322	S-adenosylmethionine decarboxylase-ornithine decarbox	-0.437
chr10	1335220	1336287	PF10_0323	early transcribed membrane protein 10.2 etramp 10.2	-0.213
chr10	1338122	1338816	PF10_0324	hypothetical protein	-0.081
chr10	1339760	1340626	PF10_0325	haloacid dehalogenase-like hydrolase putative	-0.335
chr10	1341644	1343773	PF10_0326	WD repeat protein putative	0.008
chr10	1344148	1346895	PF10_0327	Myb2 protein	-0.288
chr10	1348431	1350278	PF10_0328	bromodomain protein putative	-0.225
chr10	1351195	1353282	PF10_0329	aspartyl protease putative	0.046
chr10	1354452	1355569	PF10_0330	ubiquitin-conjugating enzyme putative	-0.470
chr10	1359315	1362674	PF10_0331	Sec1 family protein putative	0.025
chr10	1363471	1364115	PF10_0332	ribosomal protein L27 putative	-0.176
chr10	1365096	1366688	PF10_0333	hypothetical protein	0.031
chr10	1367291	1369186	PF10_0334	flavoprotein subunit of succinate dehydrogenase	-0.836
chr10	1370728	1376128	PF10_0335	hypothetical protein	-0.277
chr10	1376824	1377756	PF10_0336	hypothetical protein	0.286
chr10	1378756	1379292	PF10_0337	ADP-ribosylation factor-like protein	-0.401
chr10	1380364	1381018	PF10_0338	hypothetical protein	-0.232

chr10	1381241	1382136	PF10_0339	hypothetical protein	0.179
chr10	1383447	1386116	PF10_0340	methionine -- tRNA ligase putative	-0.431
chr10	1386646	1389627	PF10_0341	hypothetical protein conserved	0.168
chr10	1391442	1393127	PF10_0342	hypothetical protein	-0.028
chr10	1394836	1396593	PF10_0343	S-antigen	0.237
chr10	1399192	1402893	PF10_0344	glutamate-rich protein	-0.104
chr10	1404192	1405256	PF10_0345	merozoite surface protein 3	0.465
chr10	1407273	1408388	PF10_0346	merozoite surface protein 6	0.105
chr10	1409278	1410552	PF10_0347	merozoite surface protein putative	-0.148
chr10	1413197	1415290	PF10_0348	Erythrocyte membrane protein putative	-0.090
chr10	1416922	1417083	PF10_0349	hypothetical protein	0.155
chr10	1420530	1422668	PF10_0350	hypothetical protein	0.026
chr10	1423980	1425680	PF10_0351	hypothetical protein	0.797
chr10	1427124	1428341	PF10_0352	merozoite surface protein putative	0.347
chr10	1430152	1430364	PF10_0354	hypothetical protein	3.548
chr10	1432495	1434783	PF10_0355	Erythrocyte membrane protein putative	4.882
chr10	1436313	1437068	PF10_0356	liver stage antigen-1	3.320
chr10	1441900	1443567	PF10_0357	hypothetical protein	0.084
chr10	1444497	1445528	PF10_0358	hypothetical protein	-0.046
chr10	1446285	1446530	PF10_0359	hypothetical protein	0.176
chr10	1448617	1451645	PF10_0360	acetyl-coA transporter	-0.270
chr10	1452598	1458005	PF10_0361	hypothetical protein	-0.071
chr10	1462350	1469554	PF10_0362	DNA polymerase zeta catalytic subunit putative	0.061
chr10	1469989	1472226	PF10_0363	pyruvate kinase putative	-0.064
chr10	1473513	1474097	PF10_0364	hypothetical protein	0.159
chr10	1474469	1476859	PF10_0365	hypothetical protein	-0.018
chr10	1477939	1478844	PF10_0366	ADP%2FATP transporter on adenylate translocase	-0.227
chr10	1481762	1485352	PF10_0367	hypothetical protein	0.322
chr10	1487958	1490087	PF10_0368	dynamin protein putative	-0.173
chr10	1491413	1494377	PF10_0369	helicase putative	-0.331
chr10	1495972	1496657	PF10_0370	Enhancer of rudimentary homolog putative	0.320
chr10	1499861	1502154	PF10_0371	hypothetical protein	0.456
chr10	1502954	1504321	PF10_0415	hypothetical protein conserved	0.310
chr10	1505834	1506895	PF10_0372	Antigen UB05	0.213
chr10	1507581	1510523	PF10_0373	hypothetical protein	0.139

chr10	1512468	1514280	PF10_0420	hypothetical protein	0.129
chr10	1519020	1547824	PF10_0374	Pf 11-1 protein	2.565
chr10	1548454	1549730	PF10_0375	hypothetical protein	0.664
chr10	1551496	1553171	PF10_0376	hypothetical protein	0.281
chr10	1554528	1555367	PF10_0377	hypothetical protein	0.365
chr10	1557815	1560695	PF10_0378	DnaJ protein putative	0.112
chr10	1562808	1563887	PF10_0379	phospholipase putative	0.076
chr10	1568708	1571814	PF10_0380	trophozoite antigen R45 putative	1.972
chr10	1574059	1575422	PF10_0381	DnaJ protein putative	3.562
chr10	1581697	1582710	PF10_0383	hypothetical protein conserved	3.012
chr10	1591577	1592366	PF10_0390	Plasmodium falciparum Maurer%27s Cleft 2 transmembr	4.742
chr10	1599899	1601076	PF10_0393	rifin	5.406
chr10	1602934	1603988	PF10_0394	rifin	4.988
chr10	1605929	1606952	PF10_0395	stevor putative	5.227
chr10	1609062	1610421	PF10_0396	rifin	5.022
chr10	1612529	1613734	PF10_0397	rifin	4.869
chr10	1615803	1617069	PF10_0398	rifin	5.155
chr10	1619085	1620243	PF10_0399	rifin	5.113
chr10	1622367	1623683	PF10_0400	rifin	5.212
chr10	1625771	1626823	PF10_0401	rifin	5.238
chr10	1628913	1630022	PF10_0402	rifin	5.222
chr10	1632397	1633664	PF10_0403	rifin	5.151
chr10	1635595	1636778	PF10_0404	rifin	4.754
chr10	1639170	1640517	PF10_0405	rifin	4.495
chr10	1642400	1649947	PF10_0406	erythrocyte membrane protein 1 %28PfEMP1%29	4.860
chr11	24160	31598	PF11_0007	erythrocyte membrane protein 1 %28PfEMP1%29	4.564
chr11	32666	42386	PF11_0008	erythrocyte membrane protein 1 %28PfEMP1%29	4.960
chr11	45056	46233	PF11_0009	rifin	5.139
chr11	48112	49307	PF11_0010	rifin	5.120
chr11	51623	52751	PF11_0011	rifin	4.922
chr11	56792	57840	PF11_0013	stevor putative degenerate	4.857
chr11	59550	60328	PF11_0014	Plasmodium falciparum Maurer%27s Cleft 2 transmembr	4.602
chr11	60928	61350	PF11_0015	hypothetical protein	4.690
chr11	61500	61718	PF11_0016	hypothetical protein	5.174
chr11	66686	67965	PF11_0529	rifin	4.365

chr11	70081	71289	PF11_0020	rifin	3.551
chr11	73244	74442	PF11_0021	rifin	3.374
chr11	96964	99609	PF11_0033	hypothetical protein	3.227
chr11	105357	107235	PF11_0034	hypothetical protein	4.463
chr11	110028	111002	PF11_0035	hypothetical protein	2.769
chr11	114592	116574	PF11_0036	hypothetical protein conserved	0.147
chr11	119413	121507	PF11_0037	hypothetical protein	0.281
chr11	123819	124737	PF11_0038	hypothetical protein	0.200
chr11	128790	129065	PF11_0039	early transcribed membrane protein 11.1 etramp11.1	-0.803
chr11	131704	131988	PF11_0040	early transcribed membrane protein 11.2 etramp11.2	-1.063
chr11	136931	137443	PF11_0041	hypothetical protein	0.156
chr11	138647	139035	PF11_0042	hypothetical protein	0.027
chr11	140049	140802	PF11_0043	60S acidic ribosomal protein p1 putative	-0.237
chr11	146428	150816	PF11_0044	hypothetical protein	-0.050
chr11	151197	152010	PF11_0045	hypothetical protein	0.069
chr11	153193	154520	PF11_0046	hypothetical protein	0.073
chr11	156615	158114	PF11_0047	hypothetical protein	0.034
chr11	160116	160853	PF11_0048	casein kinase II beta chain putative	-0.383
chr11	163986	174101	PF11_0049	hypothetical protein conserved	-0.131
chr11	175249	175434	PF11_0050	hypothetical protein	-0.311
chr11	175609	177779	PF11_0051	phenylalanine -- tRNA ligase putative	-0.229
chr11	179152	180913	PF11_0052	syntaxin putative	0.096
chr11	182384	187224	PF11_0053	PfSNF2L	-0.264
chr11	190801	194046	PF11_0054	hypothetical protein	0.141
chr11	196768	198356	PF11_0055	hypothetical protein	-0.329
chr11	199104	201038	PF11_0056	hypothetical protein	0.037
chr11	202331	204262	PF11_0057	hypothetical protein	-0.137
chr11	204594	206008	PF11_0058	rna polymerase subunit putative	0.106
chr11	207441	209733	PF11_0059	metabolite%2Fdrug transporter	-0.248
chr11	214826	219688	PF11_0060	protein kinase	-0.099
chr11	221819	222130	PF11_0061	histone H4 putative	0.429
chr11	226066	226419	PF11_0062	histone H2B	0.538
chr11	229781	231015	PF11_0063	hypothetical protein	-0.023
chr11	232095	234239	PF11_0064	hypothetical protein	0.159
chr11	235598	236536	PF11_0065	ribosomal protein S4 putative	-0.096

chr11	239687	240613	PF11_0066	caltractin %28centrin%29 putative	-0.214
chr11	241184	243460	PF11_0067	hypothetical protein	-0.327
chr11	247451	248971	PF11_0068	hypothetical protein conserved	-0.671
chr11	251058	252872	PF11_0069	hypothetical protein	0.088
chr11	254501	255061	PF11_0070	hypothetical protein	-0.420
chr11	255714	257141	PF11_0071	RuvB DNA helicase putative	-0.370
chr11	258779	259981	PF11_0072	hypothetical protein	0.034
chr11	260867	263311	PF11_0073	hypothetical protein	0.155
chr11	263744	266570	PF11_0074	exonuclease putative	-0.134
chr11	267547	267897	PF11_0075	hypothetical protein	0.245
chr11	268456	274552	PF11_0076	hypothetical protein	-0.028
chr11	275726	279667	PF11_0077	hypothetical protein	0.011
chr11	279920	282994	PF11_0078	hypothetical protein	-0.097
chr11	283869	289224	PF11_0079	hypothetical protein	-0.061
chr11	289667	290286	PF11_0080	hypothetical protein	-0.177
chr11	291673	292502	PF11_0524	U6 snRNA associated Sm-like protein Ls%3B U6 snRNA	0.065
chr11	294151	296843	PF11_0083	nucleic acid binding factor putative	-0.200
chr11	297823	298455	PF11_0084	hypothetical protein	-0.160
chr11	299225	300734	PF11_0085	hypothetical protein	-0.177
chr11	301283	311287	PF11_0086	hypothetical protein	-0.085
chr11	317819	319217	PF11_0087	Rad51 homolog putative	-0.449
chr11	320743	321205	PF11_0088	hypothetical protein	-0.525
chr11	321886	325323	PF11_0089	hypothetical protein	0.208
chr11	326881	328812	PF11_0090	hypothetical protein	-0.298
chr11	329867	335601	PF11_0091	hypothetical protein	-0.369
chr11	343913	349351	PF11_0092	hypothetical protein	-0.109
chr11	352556	354262	PF11_0093	hypothetical protein	-0.094
chr11	356083	360434	PF11_0094	hypothetical protein	-0.279
chr11	361408	361902	PF11_0095	hypothetical protein conserved	0.002
chr11	362421	362890	PF11_0095a	pterin-4a-carbinolamine dehydratase	0.157
chr11	365093	366100	PF11_0096	casein kinase II alpha subunit putative	-0.681
chr11	367316	368823	PF11_0097	succinyl-CoA synthetase alpha subunit putative	-0.425
chr11	369675	370706	PF11_0098	endoplasmic reticulum-resident calcium binding protein	-0.119
chr11	373238	374860	PF11_0099	heat shock protein DnaJ homologue Pfj2	-0.271
chr11	377740	378396	PF11_0100	hypothetical protein	0.208

chr11	379247	380638	PF11_0101	hypothetical protein	-0.090
chr11	382569	389452	PF11_0540	hypothetical protein	0.261
chr11	390062	393331	PF11_0105	hypothetical protein	0.000
chr11	397383	397775	PF11_0106	60S Ribosomal protein L36 putative	-0.297
chr11	398659	399226	PF11_0550	hypothetical protein conserved	-0.027
chr11	400686	403904	PF11_0107	hypothetical protein	-0.004
chr11	405495	409717	PF11_0108	hypothetical protein conserved	-0.717
chr11	410202	410720	PF11_0109	hypothetical protein	0.367
chr11	414797	419556	PF11_0111	asparagine-rich antigen	-0.327
chr11	422057	425418	PF11_0112	vacuolar sorting protein 35 putative	-0.221
chr11	425904	426864	PF11_0113	ribosomal protein L11 putative	-0.474
chr11	428362	429902	PF11_0114	actin-like protein homolog ALP1 homolog	-0.503
chr11	431906	433645	PF11_0115	hypothetical protein	0.002
chr11	434802	436541	PF11_0116	hypothetical protein	-0.005
chr11	437171	438220	PF11_0117	replication factor C subunit 5 putative	-0.336
chr11	439698	441680	PF11_0118	hypothetical protein	0.198
chr11	442959	443828	PF11_0119	syntaxin putative	0.304
chr11	445233	445799	PF11_0120	hypothetical protein	0.052
chr11	446020	446559	PF11_0121	Acylphosphatase putative	-0.408
chr11	448912	451766	PF11_0122	hypothetical protein	-0.314
chr11	452459	454861	PF11_0123	hypothetical protein	0.208
chr11	455669	457258	PF11_0124	hypothetical protein	0.215
chr11	457936	459197	PF11_0125	hypothetical protein	0.440
chr11	459776	460050	PF11_0126	hypothetical protein	0.291
chr11	462290	469672	PF11_0127	hypothetical protein	0.014
chr11	470541	471608	PF11_0128	coq4 homolog putative	-0.046
chr11	471946	474674	PF11_0129	hypothetical protein	0.310
chr11	475880	476455	PF11_0130	hypothetical protein	-0.274
chr11	477484	478320	PF11_0526	hypothetical protein	0.244
chr11	478823	480946	PF11_0131	hypothetical protein	-0.175
chr11	481851	485831	PF11_0527	hypothetical protein	0.091
chr11	486329	487666	PF11_0134	hypothetical protein	0.057
chr11	488480	489045	PF11_0135	hypothetical protein	-0.158
chr11	489834	490154	PF11_0136	hypothetical protein	0.323
chr11	492090	510737	PF11_0528	hypothetical protein	0.118

chr11	511104	511760	PF11_0139	protein tyrosine phosphatase putative	-0.777
chr11	517310	517984	PF11_0140	hypothetical protein	0.312
chr11	519188	520219	PF11_0141	UDP-galactose transporter putative	-0.094
chr11	521121	522823	PF11_0142	hypothetical protein conserved	-0.445
chr11	524491	526587	PF11_0143	hypothetical protein	-0.199
chr11	527571	528701	PF11_0144	hypothetical protein	-0.958
chr11	529492	530562	PF11_0145	glyoxalase I putative	-0.232
chr11	531176	533284	PF11_0146	hypothetical protein	0.396
chr11	534972	536498	PF11_0147	mitogen-activated protein kinase 2	-0.256
chr11	537646	538296	PF11_0148	dynein light chain type 2 putative	-0.354
chr11	539902	540371	PF11_0149	hypothetical protein	-0.219
chr11	540602	541386	PF11_0150	rhomboid protease putative	-0.900
chr11	542329	543983	PF11_0151	GTPase activating protein GAP	0.040
chr11	544404	544764	PF11_0152	hypothetical protein	-0.219
chr11	547382	549295	PF11_0153	hypothetical protein	0.280
chr11	549865	551151	PF11_0154	hypothetical protein	0.135
chr11	552258	553265	PF11_0155	hypothetical protein	-0.243
chr11	555513	558667	PF11_0156	serine%2Fthreonine protein kinase	-0.584
chr11	558923	561031	PF11_0157	glycerol-3-phosphate dehydrogenase putative	-0.144
chr11	562016	569458	PF11_0158	hypothetical protein	0.134
chr11	570016	570709	PF11_0159	hypothetical protein	0.062
chr11	571140	571445	PF11_0525	hypothetical protein	-0.549
chr11	571780	573251	PF11_0535	hypothetical protein conserved	-0.238
chr11	576772	580275	PF11_0160	hypothetical protein	-0.155
chr11	581249	582697	PF11_0161	falcipain-2 precursor putative	-0.062
chr11	584327	585805	PF11_0162	falcipain-3	-0.284
chr11	588168	589399	PF11_0163	hypothetical protein	0.118
chr11	589706	590628	PF11_0164	peptidyl-prolyl cis-trans isomerase	-0.299
chr11	592129	593583	PF11_0165	falcipain 2 precursor	0.220
chr11	596288	597202	PF11_0166	hypothetical protein	-0.542
chr11	600175	602214	PF11_0167	hypothetical protein	-0.069
chr11	603294	613393	PF11_0168	hypothetical protein	-0.162
chr11	615957	616579	PF11_0169	hypothetical protein	-0.298
chr11	618311	619777	PF11_0170	cyclophilin putative	-0.243
chr11	619892	621349	PF11_0171	hypothetical protein	-0.440

chr11	624369	625736	PF11_0172	folate%2Fbiopterin transporter putative	-0.323
chr11	626708	629431	PF11_0173	oligosacharyl transferase STT3 subunit putative	-0.343
chr11	630778	632880	PF11_0174	cathepsin C homolog	-0.535
chr11	636093	639510	PF11_0175	heat shock protein 101 putative	-0.298
chr11	646921	651342	PF11_0176	hypothetical protein	-0.089
chr11	652578	653975	PF11_0177	ubiquitin C-terminal hydrolase family 1 putative	-0.214
chr11	654870	660419	PF11_0178	hypothetical protein	-0.010
chr11	661999	662385	PF11_0179	hypothetical protein	-0.024
chr11	664162	665355	PF11_0180	hypothetical protein	-0.228
chr11	665647	667721	PF11_0181	tyrosine --tRNA ligase putative	0.757
chr11	668367	669490	PF11_0182	hypothetical protein	-0.114
chr11	670710	671514	PF11_0183	GTP-binding nuclear protein ran%2Ftc4	-0.335
chr11	674298	677348	PF11_0184	DNA mismatch repair protein MLH1 putative	-0.356
chr11	678751	683280	PF11_0185	hypothetical protein	-0.172
chr11	683859	684615	PF11_0186	hypothetical protein	-0.322
chr11	687234	687780	PF11_0187	clathrin assembly protein AP19 putative	-0.622
chr11	688765	691557	PF11_0188	heat shock protein 90 putative	-0.351
chr11	694654	699120	PF11_0189	hypothetical protein	-0.191
chr11	699868	700788	PF11_0190	hypothetical protein	-0.268
chr11	703174	704958	PF11_0191	hypothetical protein	-0.393
chr11	705555	707381	PF11_0192	hypothetical protein	-0.261
chr11	709174	710688	PF11_0193	hypothetical protein	0.225
chr11	710809	712862	PF11_0194	hypothetical protein	0.076
chr11	714116	716286	PF11_0195	hypothetical protein	-0.145
chr11	716794	718411	PF11_0196	hypothetical protein	-0.071
chr11	718649	719764	PF11_0197	hypothetical protein	-0.291
chr11	720991	722272	PF11_0198	hypothetical protein conserved	0.029
chr11	722676	723288	PF11_0199	hypothetical protein	0.176
chr11	724854	725738	PF11_0200	U2 snRNP auxiliary factor small subunit putative	-0.713
chr11	727895	734338	PF11_0201	hypothetical protein	0.030
chr11	736068	737734	PF11_0202	clathrin coat assembly protein putative	-0.029
chr11	738512	741670	PF11_0203	peptidase	-0.361
chr11	744873	745931	PF11_0204	hypothetical protein	-0.221
chr11	746853	747455	PF11_0205	hypothetical protein	-0.178
chr11	749151	754400	PF11_0206	hypothetical protein	0.005

chr11	756191	759518	PF11_0207	hypothetical protein	0.066	
chr11	761694	762817	PF11_0208	phosphoglycerate mutase putative	-0.461	
chr11	765032	765217	PF11_0209	hypothetical protein	-0.460	
chr11	766851	768459	PF11_0210	hypothetical protein	-0.059	
chr11	769251	770599	PF11_0211	hypothetical protein conserved	-0.152	
chr11	772538	775041	PF11_0212	hypothetical protein	0.177	
chr11	775254	783514	PF11_0213	hypothetical protein	0.117	
chr11	784591	788349	PF11_0214	hypothetical protein	0.403	
chr11	790010	790261	PF11_0215	hypothetical protein	-0.418	
chr11	790266	790607	PF11_0216	hypothetical protein	-0.946	
chr11	792196	793944	PF11_0217	hypothetical protein	-0.175	
chr11	794253	798818	PF11_0218	hypothetical protein	0.186	
chr11	800780	801019	PF11_0219	hypothetical protein	-0.791	
chr11	801248	807080	PF11_0220	protein kinase	0.159	
chr11	810209	810397	PF11_0221	hypothetical protein	-0.171	
chr11	810733	812289	PF11_0222	hypothetical protein	-0.198	
chr11	812582	814129	PF11_0223	hypothetical protein	0.166	
chr11	815385	816321	PF11_0224	circumsporozoite-related antigen	-0.515	
chr11	820072	822519	PF11_0225	PfGCN20	-0.202	
chr11	823238	829312	PF11_0226	petidase M16 family	0.614	11cenL
chr11	835888	837465	PF11_0227	serine%2Fthreonine protein kinase puative	0.650	11cenR
chr11	838379	840099	PF11_0228	hypothetical protein	0.341	
chr11	840915	842449	PF11_0229	hypothetical protein	0.234	
chr11	844374	844973	PF11_0230	hypothetical protein	-0.046	
chr11	846928	848307	PF11_0231	hypothetical protein	0.455	
chr11	849334	852569	PF11_0232	hypothetical protein	0.094	
chr11	853354	859224	PF11_0233	hypothetical protein	0.081	
chr11	860133	862760	PF11_0234	hypothetical protein	0.102	
chr11	862851	863958	PF11_0235	hypothetical protein	0.120	
chr11	864183	867048	PF11_0236	hypothetical protein	0.042	
chr11	867313	867721	PF11_0237	hypothetical protein	0.191	
chr11	868387	869151	PF11_0238	hypothetical protein	-0.024	
chr11	872030	877677	PF11_0239	protein kinase FIKK family	-0.145	
chr11	878968	895272	PF11_0240	dynein heavy chain putative	0.112	
chr11	895966	903369	PF11_0241	hypothetical protein	0.196	

chr11	909365	916323	PF11_0242	protein kinase	-0.178
chr11	918227	921068	PF11_0243	leucine-rich repeat protein 11 LRR11	-0.181
chr11	921822	923891	PF11_0244	hypothetical protein	-0.050
chr11	925727	927523	PF11_0245	translation elongation factor EF-1 subunit alpha putative	-0.813
chr11	929353	933363	PF11_0246	hypothetical protein	-0.215
chr11	936083	938221	PF11_0247	hypothetical protein	-0.262
chr11	938949	939611	PF11_0248	hypothetical protein	-0.363
chr11	940526	944332	PF11_0249	hypothetical protein	0.003
chr11	945469	946147	PF11_0250	high mobility group-like protein NHP2 putative	-0.484
chr11	948590	950581	PF11_0251	endoplasmic reticulum oxidoreductin putative	0.025
chr11	951381	956342	PF11_0252	hypothetical protein	-0.145
chr11	957513	958841	PF11_0253	hypothetical protein	-0.472
chr11	962155	963957	PF11_0254	hypothetical protein	-0.378
chr11	964674	965359	PF11_0255	hypothetical protein	-0.332
chr11	966979	968805	PF11_0256	pyruvate dehydrogenase E1 component alpha subunit p	-0.338
chr11	970104	971375	PF11_0257	ethanolamine kinase putative	-0.177
chr11	974653	975537	PF11_0258	co-chaperone GrpE putative	-0.099
chr11	976879	977935	PF11_0259	hypothetical protein	-0.028
chr11	981944	982765	PF11_0260	ribosomal protein L35 putative	-0.143
chr11	983950	985618	PF11_0261	hypothetical protein	-0.198
chr11	986629	988119	PF11_0262	hypothetical protein	0.106
chr11	989474	990660	PF11_0263	hypothetical protein	-0.013
chr11	991862	996457	PF11_0264	DNA-dependent RNA polymerase	-0.328
chr11	997922	999103	PF11_0265	mitochondrial import inner membrane translocase putati	-0.580
chr11	1001181	1001561	PF11_0266	small nuclear ribonucleoprotein D1 putative	-0.456
chr11	1004779	1007353	PF11_0267	hypothetical protein	-0.355
chr11	1008408	1010852	PF11_0268	hypothetical protein	-0.412
chr11	1013465	1013746	PF11_0269	hypothetical protein	-0.550
chr11	1014333	1017374	PF11_0270	threonine -- tRNA ligase putative	-0.277
chr11	1018365	1022494	PF11_0271	hypothetical protein conserved	-0.079
chr11	1023789	1024495	PF11_0272	ribosomal protein S18 putative	-0.394
chr11	1026974	1029565	PF11_0273	hypothetical protein	0.248
chr11	1030677	1031479	PF11_0274	hypothetical protein	-0.102
chr11	1033060	1037166	PF11_0275	hypothetical protein	-0.151
chr11	1037641	1039689	PF11_0276	hypothetical protein	0.003

chr11	1040788	1041840	PF11_0277	hypothetical protein	-0.002
chr11	1042175	1046085	PF11_0278	hypothetical protein	0.339
chr11	1049919	1052663	PF11_0279	hypothetical protein	-0.241
chr11	1053812	1054275	PF11_0280	small nuclear ribonucleoprotein F putative	-0.262
chr11	1056196	1058052	PF11_0281	hypothetical protein	0.050
chr11	1060260	1060781	PF11_0282	deoxyuridine 5%27-triphosphate nucleotidohydrolase pu	-0.782
chr11	1062716	1062967	PF11_0283	hypothetical protein	0.320
chr11	1063668	1064549	PF11_0284	methyltransferase putative	0.335
chr11	1065783	1066567	PF11_0285	hypothetical protein	0.306
chr11	1067195	1070217	PF11_0286	hypothetical protein conserved	0.016
chr11	1071907	1073811	PF11_0287	hypothetical protein	0.007
chr11	1075186	1076403	PF11_0288	hypothetical protein	-0.019
chr11	1077286	1081548	PF11_0289	hypothetical protein	0.138
chr11	1083319	1085060	PF11_0290	hypothetical protein	0.032
chr11	1087312	1093103	PF11_0291	hypothetical protein	-0.038
chr11	1094907	1095929	PF11_0292	hypothetical protein	-0.065
chr11	1097115	1097525	PF11_0293	multiprotein bridging factor type 1 putative	-0.160
chr11	1098165	1103553	PF11_0294	ATP-dependent phosphofructokinase putative	-0.125
chr11	1104152	1106338	PF11_0295	farnesyl pyrophosphate synthase putative	-0.193
chr11	1107784	1109940	PF11_0296	hypothetical protein	-0.176
chr11	1110434	1112113	PF11_0297	hypothetical protein	-0.255
chr11	1113994	1115475	PF11_0298	GPI8p transamidase	-0.123
chr11	1116597	1117269	PF11_0299	hypothetical protein	0.464
chr11	1118247	1120683	PF11_0300	hypothetical protein	-0.273
chr11	1121928	1123375	PF11_0301	spermidine synthase	0.003
chr11	1125950	1127308	PF11_0302	hypothetical protein	-0.367
chr11	1128772	1130558	PF11_0303	26S proteasome regulatory complex subunit putative	-0.237
chr11	1131292	1136832	PF11_0304	hypothetical protein	0.065
chr11	1138073	1139977	PF11_0305	hypothetical protein	-0.116
chr11	1141484	1143908	PF11_0306	A%2FG-specific adenine glycosylase putative	-0.224
chr11	1144752	1148768	PF11_0307	hypothetical protein	-0.257
chr11	1149974	1150516	PF11_0308	hypothetical protein	0.186
chr11	1152377	1153225	PF11_0309	hypothetical protein	-0.260
chr11	1156435	1158264	PF11_0310	transporter putative	-0.722
chr11	1159364	1162186	PF11_0311	N-acetyl glucosamine phosphate mutase putative	-0.194

chr11	1163092	1163355	PF11_0312	ribosomal protein L38e	-0.271
chr11	1165219	1166169	PF11_0313	ribosomal phosphoprotein P0	-0.492
chr11	1168877	1170196	PF11_0314	26S protease subunit regulatory subunit 6a putative	-0.561
chr11	1171678	1175722	PF11_0315	hypothetical protein	0.055
chr11	1176439	1179703	PF11_0316	hypothetical protein	0.016
chr11	1180158	1185614	PF11_0317	structural maintenance of chromosome protein putative	0.195
chr11	1187602	1188990	PF11_0318	PF16 protein putative	-0.263
chr11	1190121	1194333	PF11_0319	hypothetical protein	0.389
chr11	1194522	1195034	PF11_0320	hypothetical protein	-0.232
chr11	1195986	1198307	PF11_0321	hypothetical protein	0.720
chr11	1199251	1201803	PF11_0322	hypothetical protein	0.328
chr11	1201920	1203617	PF11_0323	hypothetical protein	0.464
chr11	1204295	1209585	PF11_0324	hypothetical protein	0.176
chr11	1210684	1211454	PF11_0325	hypothetical protein	-0.593
chr11	1216519	1224810	PF11_0326	hypothetical protein	1.272
chr11	1227218	1231948	PF11_0327	hypothetical protein	-0.429
chr11	1234059	1236308	PF11_0328	hypothetical protein	-0.169
chr11	1239898	1242546	PF11_0329	hypothetical protein	-0.285
chr11	1243224	1246616	PF11_0330	hypothetical protein	-0.003
chr11	1248308	1250230	PF11_0331	t-complex protein 1 alpha subunit putative	-0.759
chr11	1253071	1253892	PF11_0332	hypothetical protein	-0.138
chr11	1255284	1260442	PF11_0333	hypothetical protein	0.063
chr11	1261757	1264625	PF11_0334	amino acid transporter putative	0.085
chr11	1265123	1266076	PF11_0335	hypothetical protein	0.133
chr11	1266248	1267582	PF11_0336	hypothetical protein conserved	-0.043
chr11	1268453	1269454	PF11_0337	50S ribosomal protein L2 putative	-1.291
chr11	1271202	1271978	PF11_0338	Aquaglyceroporin	-0.538
chr11	1273405	1274007	PF11_0339	hypothetical protein	-0.123
chr11	1275586	1275711	PF11_0340	hypothetical protein	-0.130
chr11	1277344	1280635	PF11_0341	hypothetical protein	0.091
chr11	1281282	1287631	PF11_0342	hypothetical protein	-0.020
chr11	1290499	1291599	PF11_0343	hypothetical protein conserved	-0.208
chr11	1293854	1295722	PF11_0344	apical membrane antigen 1 AMA1	-0.753
chr11	1296799	1298197	PF11_0345	hypothetical protein	0.052
chr11	1298472	1300598	PF11_0346	hypothetical protein	0.192

chr11	1302171	1305985	PF11_0347	hypothetical protein	-0.154
chr11	1309211	1311791	PF11_0348	hypothetical protein	0.006
chr11	1313255	1313554	PF11_0349	hypothetical protein	-0.471
chr11	1313989	1317171	PF11_0350	hypothetical protein	-0.175
chr11	1320112	1322103	PF11_0351	heat shock protein hsp70 homologue	-0.898
chr11	1323041	1324312	PF11_0352	protein disulfide isomerase related protein	-0.138
chr11	1326745	1332162	PF11_0353	hypothetical protein	0.205
chr11	1333534	1340217	PF11_0354	hypothetical protein	-0.227
chr11	1340666	1341626	PF11_0355	hypothetical protein	0.090
chr11	1344301	1349394	PF11_0356	hypothetical protein	0.328
chr11	1350619	1355787	PF11_0357	hypothetical protein	0.088
chr11	1359362	1364111	PF11_0358	DNA-directed RNA polymerase beta subunit putative	-0.168
chr11	1367184	1368807	PF11_0359	coatomer delta subunit putative	0.078
chr11	1369617	1371665	PF11_0360	hypothetical protein	-0.026
chr11	1372309	1373847	PF11_0361	hypothetical protein	0.036
chr11	1374590	1376659	PF11_0362	protein phosphatase putative	-0.623
chr11	1381013	1382242	PF11_0363	hypothetical protein	0.018
chr11	1384376	1385113	PF11_0364	hypothetical protein	-0.094
chr11	1387569	1388907	PF11_0365	hypothetical protein	0.160
chr11	1390062	1391075	PF11_0366	hypothetical protein	0.110
chr11	1391614	1392051	PF11_0367	hypothetical protein	-0.431
chr11	1392158	1396171	PF11_0368	hypothetical protein	0.244
chr11	1397504	1397983	PF11_0369	hypothetical protein	0.129
chr11	1398692	1400599	PF11_0370	hypothetical protein	0.022
chr11	1403904	1414510	PF11_0371	hypothetical protein	-0.015
chr11	1415404	1417212	PF11_0372	hypothetical protein	0.035
chr11	1417464	1420068	PF11_0373	hypothetical protein	-0.174
chr11	1422741	1426198	PF11_0374	hypothetical protein	-0.302
chr11	1427008	1429668	PF11_0375	hypothetical protein	0.344
chr11	1433138	1435167	PF11_0377	casein kinase 1 PfCK1	-0.300
chr11	1441500	1445468	PF11_0378	hypothetical protein	-0.199
chr11	1446486	1448502	PF11_0379	hypothetical protein	-0.047
chr11	1450638	1451447	PF11_0380	hypothetical protein	-0.293
chr11	1456148	1460316	PF11_0381	subtilisin-like protease 2	-0.378
chr11	1462165	1463067	PF11_0382	ribosomal protein S9 putative	-0.509

chr11	1463808	1465055	PF11_0383	hypothetical protein	0.094
chr11	1466194	1468263	PF11_0384	hypothetical protein conserved	0.117
chr11	1470597	1471874	PF11_0385	hypothetical protein	-0.373
chr11	1473502	1474113	PF11_0386	30S ribosomal protein S14 putative	-0.627
chr11	1474664	1476319	PF11_0387	hypothetical protein	-0.215
chr11	1476443	1476835	PF11_0388	hypothetical protein	0.322
chr11	1477640	1479847	PF11_0389	hypothetical protein	-0.018
chr11	1480899	1481529	PF11_0545	hypothetical protein	-0.408
chr11	1483372	1485357	PF11_0390	reticulocyte binding protein putative	-0.119
chr11	1485643	1485855	PF11_0391	hypothetical protein	0.344
chr11	1487591	1496140	PF11_0392	hypothetical protein	-0.032
chr11	1497131	1497511	PF11_0393	hypothetical protein conserved	0.210
chr11	1498205	1498966	PF11_0394	hypothetical protein	0.013
chr11	1499134	1500250	PF11_0530	hypothetical protein	-0.044
chr11	1501008	1513688	PF11_0395	guanylyl cyclase	-0.170
chr11	1518305	1521235	PF11_0396	Protein phosphatase 2C	-0.411
chr11	1522596	1524343	PF11_0397	hypothetical protein conserved	0.149
chr11	1525651	1531741	PF11_0398	hypothetical protein	0.256
chr11	1532780	1533927	PF11_0399	hypothetical protein	-0.037
chr11	1534166	1535167	PF11_0400	hypothetical protein	-0.386
chr11	1535297	1539853	PF11_0401	hypothetical protein	0.043
chr11	1548183	1553558	PF11_0402	hypothetical protein	-0.163
chr11	1554151	1555502	PF11_0403	hypothetical protein	0.280
chr11	1556741	1565042	PF11_0404	malaria antigen	-0.154
chr11	1576129	1578975	PF11_0405	hypothetical protein conserved	-0.240
chr11	1580591	1582480	PF11_0406	hypothetical protein	-0.020
chr11	1582992	1584509	PF11_0407	adrenodoxin reductase putative	0.254
chr11	1585390	1592031	PF11_0408	hypothetical protein	0.191
chr11	1593963	1594948	PF11_0409	hypothetical protein conserved	-0.311
chr11	1595845	1597101	PF11_0410	hypothetical protein	-0.114
chr11	1597287	1597715	PF11_0411	hypothetical protein	-0.160
chr11	1598547	1599629	PF11_0412	Vacuolar ATP synthase subunit F putative	0.217
chr11	1601254	1603305	PF11_0413	hypothetical protein	0.487
chr11	1605318	1606403	PF11_0414	hypothetical protein	0.253
chr11	1608003	1608606	PF11_0415	hypothetical protein	0.181

chr11	1610097	1615579	PF11_0416	myosin heavy chain subunit putative	0.006
chr11	1616413	1621887	PF11_0417	hypothetical protein	0.309
chr11	1623444	1630271	PF11_0418	hypothetical protein	0.083
chr11	1633917	1636855	PF11_0419	hypothetical protein	-0.022
chr11	1637284	1643389	PF11_0420	hypothetical protein	0.103
chr11	1644908	1647379	PF11_0421	hypothetical protein	-0.062
chr11	1649318	1657009	PF11_0422	hypothetical protein	1.135
chr11	1657504	1657975	PF11_0423	hypothetical protein	-0.697
chr11	1659067	1662356	PF11_0424	hypothetical protein	0.043
chr11	1663618	1665247	PF11_0425	hypothetical protein	0.131
chr11	1665758	1666765	PF11_0426	hypothetical protein	-0.325
chr11	1668299	1669078	PF11_0427	dolichyl-phosphate b-D-mannosyltransferase putative	-0.436
chr11	1669948	1671509	PF11_0428	hypothetical protein conserved	-0.193
chr11	1672516	1674554	PF11_0429	hypothetical protein	-0.046
chr11	1676252	1677766	PF11_0431	membrane skeletal protein putative	-0.235
chr11	1679075	1681153	PF11_0432	hypothetical protein	0.100
chr11	1681865	1690081	PF11_0433	hypothetical protein	0.108
chr11	1692894	1693538	PF11_0434	hypothetical protein	0.329
chr11	1695504	1701255	PF11_0435	hypothetical protein	-0.215
chr11	1702086	1703639	PF11_0436	coproporphyrinogen oxidase	-0.114
chr11	1705693	1706570	PF11_0437	hypothetical protein	0.096
chr11	1708063	1708676	PF11_0438	Ribosomal protein putative	0.389
chr11	1710322	1712169	PF11_0439	hypothetical protein	-0.169
chr11	1713078	1713971	PF11_0440	hypothetical protein	-0.132
chr11	1715280	1718691	PF11_0441	hypothetical protein	0.117
chr11	1720044	1724858	PF11_0442	hypothetical protein	-0.268
chr11	1729790	1730896	PF11_0443	hypothetical protein conserved	0.132
chr11	1731896	1732897	PF11_0445	DNA-directed RNA polymerase I putative	-0.393
chr11	1735123	1736613	PF11_0447	translation initiation factor eIF-1A putative	0.038
chr11	1737456	1740026	PF11_0448	hypothetical protein	0.156
chr11	1740932	1741825	PF11_0449	hypothetical protein	0.169
chr11	1742586	1743032	PF11_0450	hypothetical protein	-0.048
chr11	1743656	1745345	PF11_0451	hypothetical protein	0.211
chr11	1748436	1751135	PF11_0452	hypothetical protein	-0.450
chr11	1751574	1753266	PF11_0453	hypothetical protein conserved	-0.225

chr11	1755435	1755932	PF11_0454	Ribosomal protein 40S subunit putative	-0.604
chr11	1756554	1757179	PF11_0560	hypothetical protein conserved	-0.181
chr11	1757485	1758634	PF11_0455	hypothetical protein	0.404
chr11	1762253	1766647	PF11_0456	hypothetical protein	0.026
chr11	1768325	1769341	PF11_0457	hypothetical protein	0.022
chr11	1770946	1771899	PF11_0458	hypothetical protein	-0.346
chr11	1772347	1773816	PF11_0459	hypothetical protein	-0.063
chr11	1773946	1776723	PF11_0460	hypothetical protein	0.192
chr11	1777522	1778925	PF11_0461	PfRab6 GTPase	0.252
chr11	1780081	1782395	PF11_0462	hypothetical protein	0.077
chr11	1783354	1786560	PF11_0463	coat protein gamma subunit putative	-0.142
chr11	1789320	1795637	PF11_0464	serine%2Fthreonine protein kinase	0.032
chr11	1800541	1803547	PF11_0465	dynamin-like protein	-0.384
chr11	1807356	1810123	PF11_0466	transport protein putative	0.014
chr11	1810470	1811968	PF11_0467	hypothetical protein	-0.168
chr11	1813416	1814570	PF11_0468	hypothetical protein	0.360
chr11	1814954	1817701	PF11_0469	hypothetical protein	-0.105
chr11	1819741	1820740	PF11_0470	hypothetical protein	0.406
chr11	1821029	1822966	PF11_0471	hypothetical protein	-0.558
chr11	1824846	1825058	PF11_0472	hypothetical protein	-0.072
chr11	1826391	1826789	PF11_0473	hypothetical protein	-0.265
chr11	1828338	1829051	PF11_0474	hypothetical protein conserved	0.355
chr11	1830461	1831414	PF11_0476	leucine-rich repeat protein 10 LRR10	0.037
chr11	1834398	1838770	PF11_0477	CCAAT-box DNA binding protein subunit B	0.047
chr11	1839420	1842875	PF11_0478	hypothetical protein	1.641
chr11	1843642	1853611	PF11_0479	hypothetical protein	1.725
chr11	1858326	1867354	PF11_0480	hypothetical protein	0.782
chr11	1873144	1881411	PF11_0481	hypothetical protein	0.513
chr11	1883772	1887857	PF11_0482	hypothetical protein	-0.441
chr11	1888634	1892594	PF11_0483	farnesyltransferase beta subunit putative	0.093
chr11	1893467	1895840	PF11_0484	hypothetical protein	0.066
chr11	1897201	1897881	PF11_0485	mitochondrial ATP-synthase delta subunit putative	-0.099
chr11	1899178	1905939	PF11_0486	MAEBL putative	0.942
chr11	1907052	1907705	PF11_0487	hypothetical protein	0.108
chr11	1909207	1913496	PF11_0488	serine%2Fthreonine protein kinase	-0.044

chr11	1925939	1933147	MAL11_rRNA	MAL11_rRNA	0.357
chr11	1934330	1935579	PF11_0503	hypothetical protein	0.291
chr11	1941677	1942585	PF11_0504	hypothetical protein	-0.521
chr11	1945775	1946044	PF11_0505	hypothetical protein	-0.137
chr11	1950207	1951919	PF11_0506	hypothetical protein	-0.361
chr11	1952200	1968723	PF11_0507	antigen 332 putative	0.087
chr11	1972397	1973871	PF11_0508	hypothetical protein	-0.276
chr11	1976404	1979801	PF11_0509	ring-infected erythrocyte surface antigen putative	0.386
chr11	1982263	1984244	PF11_0510	protein kinase FIKK family	0.248
chr11	1985867	1986433	PF11_0511	hypothetical protein	0.579
chr11	1991356	1994015	PF11_0512	ring-infected erythrocyte surface antigen 2 RESA-2 - ma	3.633
chr11	2001547	2003310	PF11_0513	hypothetical protein	3.788
chr11	2004751	2005008	PF11_0514	hypothetical protein	4.402
chr11	2006703	2007826	PF11_0515	rifin	4.812
chr11	2009872	2010899	PF11_0516	stevor putative	4.950
chr11	2013384	2014313	PF11_0517	rifin	5.036
chr11	2015649	2016506	PF11_0518	rifin putative truncated pseudogene	5.425
chr11	2018711	2019964	PF11_0519	rifin	5.220
chr11	2021851	2023031	PF11_0520	rifin	5.226
chr11	2025814	2035883	PF11_0521	erythrocyte membrane protein 1 %28PfEMP1%29	5.260
chr12	16973	24497	PFL0005w	erythrocyte membrane protein 1 %28PfEMP1%29	4.777
chr12	26321	27687	PFL0010c	rifin	3.362
chr12	30078	31261	PFL0015c	rifin	4.658
chr12	32703	41940	PFL0020w	erythrocyte membrane protein 1 %28PfEMP1%29	4.852
chr12	46788	56805	PFL0030c	erythrocyte membrane protein 1 %28PfEMP1%29	3.755
chr12	61556	64336	PFL0035c	acetyl-CoA synthesase PfACS7	3.573
chr12	68808	70867	PFL0040c	protein kinase FIKK family	0.087
chr12	73229	74522	PFL0045c	hypothetical protein conserved	0.190
chr12	76846	79067	PFL0050c	hypothetical protein	0.333
chr12	81449	84451	PFL0055c	protein with DNAJ domain %28resa-like%29 putative	0.025
chr12	89934	90749	PFL0060w	hypothetical protein conserved in P. falciparum	-0.122
chr12	92242	92562	PFL0065w	hypothetical protein conserved	0.064
chr12	93581	97146	PFL0070c	hypothetical protein conserved in P. falciparum	0.160
chr12	98978	100471	PFL0075w	XPA binding protein 1 putative	-0.113
chr12	101581	102624	PFL0080c	serine%2Fthreonine-protein kinase Pfnek-3	0.237

chr12	103861	104604	PFL0085c	hypothetical protein conserved	0.176
chr12	105683	106339	PFL0090c	hypothetical protein conserved	-0.266
chr12	107338	108202	PFL0095c	hypothetical protein conserved	-0.210
chr12	109501	112641	PFL0100c	ATP dependent RNA helicase putative	0.210
chr12	113787	115923	PFL0105w	hypothetical protein conserved	-0.029
chr12	116475	117449	PFL0110c	mitochondrial phosphate carrier protein PfmpC	-0.521
chr12	119402	141499	PFL0115w	hypothetical protein conserved	0.564
chr12	141642	143268	PFL0120c	cyclophilin putative	-0.281
chr12	144046	147284	PFL0125c	hypothetical protein conserved	0.052
chr12	148237	155476	PFL0130c	hypothetical protein conserved	-0.309
chr12	158230	160131	PFL0135w	hypothetical protein conserved	-0.027
chr12	160713	161252	PFL0140w	hypothetical protein conserved	0.105
chr12	162426	162719	PFL0145c	high mobility group protein	-0.327
chr12	167703	171272	PFL0150w	origin recognition complex 1 protein	-0.457
chr12	171504	172133	PFL0155c	hypothetical protein conserved in P. falciparum	-0.062
chr12	172933	173358	PFL0160w	signalrecognition particle subunit SRP14	-0.295
chr12	173872	176850	PFL0165c	hypothetical protein conserved	-0.123
chr12	178159	181911	PFL0170w	transporter	0.122
chr12	182295	183965	PFL0175c	hypothetical protein conserved	-0.380
chr12	184911	185765	PFL0180w	cytochrome c1 heme lyase putative	0.095
chr12	187143	188839	PFL0185c	nucleosome assembly protein 1 putative	-0.140
chr12	194141	195459	PFL0190w	ubiquitin-conjugating enzyme e2 putative	-0.044
chr12	197506	198518	PFL0195w	hypothetical protein conserved	0.031
chr12	198967	202236	PFL0200c	hypothetical protein conserved	0.298
chr12	202945	204834	PFL0205w	hypothetical protein conserved	-0.123
chr12	205488	205973	PFL0210c	eukaryotic initiation factor 5a putative	0.978
chr12	208366	208784	PFL0215c	hypothetical protein conserved in P. falciparum	-0.644
chr12	209438	210890	PFL0220c	hypothetical protein conserved	0.030
chr12	211514	212353	PFL0225c	hypothetical protein conserved	-0.182
chr12	213209	214189	PFL0230w	hypothetical protein	-0.527
chr12	214832	215693	PFL0235w	hypothetical protein conserved	-0.185
chr12	216293	216757	PFL0240w	hypothetical protein	0.301
chr12	217182	218237	PFL0245w	hypothetical protein	0.209
chr12	219578	222171	PFL0250w	hypothetical protein conserved	-0.350
chr12	222693	224989	PFL0255c	uga suppressor tRNA-associated antigenic protein putat	-0.275

chr12	226052	227068	PFL0260c	hypothetical protein conserved	-0.152
chr12	230536	231405	PFL0265w	hypothetical protein conserved	0.041
chr12	232387	235644	PFL0270c	hypothetical protein conserved	0.143
chr12	242808	248165	PFL0275w	hypothetical protein conserved	-0.405
chr12	249333	251396	PFL0280c	hypothetical protein conserved	0.007
chr12	253152	254120	PFL0285w	glyoxalase II family protein putative	0.140
chr12	256364	263728	PFL0290w	hypothetical protein conserved	-0.314
chr12	264612	267860	PFL0295c	hypothetical protein conserved	-0.302
chr12	270297	271211	PFL0300c	phosphoesterase putative	-0.309
chr12	272317	274739	PFL0305c	hypothetical protein conserved	-0.076
chr12	275741	279333	PFL0310c	eukaryotictranslation initiation factor 3 subunit 8 putative	-0.140
chr12	281017	289299	PFL0315c	hypothetical protein conserved	0.091
chr12	290833	291873	PFL0320w	hypothetical protein conserved	0.176
chr12	292421	294376	PFL0325w	hypothetical protein conserved	-0.029
chr12	294994	299346	PFL0330c	DNA-directed RNA polymerase III subunit putative	-0.334
chr12	301126	302823	PFL0335c	eukaryotictranslation initiation factor 5 putative	-0.183
chr12	308211	310394	PFL0340w	hypothetical protein conserved	0.019
chr12	310828	313236	PFL0345w	hypothetical protein conserved	-0.060
chr12	313866	321905	PFL0350c	hypothetical protein conserved	0.070
chr12	324332	326854	PFL0355c	hypothetical protein conserved	-0.093
chr12	329631	337802	PFL0360c	hypothetical protein	0.108
chr12	343746	344356	PFL0365c	hypothetical protein conserved	-0.095
chr12	345917	347234	PFL0370w	hypothetical protein conserved	0.494
chr12	348269	349095	PFL0375w	hypothetical protein	0.190
chr12	349474	351279	PFL0380c	tRNA delta%282%29-isopentenylpyrophosphate transfer	-0.254
chr12	351845	354001	PFL0385c	blood stage antigen 41-3 precursor	0.142
chr12	354535	357441	PFL0390c	hypothetical protein conserved	-0.071
chr12	357910	359351	PFL0395c	hypothetical protein conserved	0.091
chr12	359990	360494	PFL0400w	50S ribosomal protein L29 putative	-0.063
chr12	361895	375869	PFL0405w	hypothetical protein conserved	0.182
chr12	376547	387478	PFL0410w	hypothetical protein conserved	1.012
chr12	388540	389142	PFL0415w	acyl carrier protein mitochondrial precursor putative	0.122
chr12	390927	395621	PFL0420w	amino acid transporter	-0.030
chr12	395981	401242	PFL0425c	hypothetical protein conserved	0.145
chr12	402295	402522	PFL0430w	tim10 homologue putative	-0.632

chr12	403345	404809	PFL0435w	hypothetical protein conserved	-0.025
chr12	405603	409145	PFL0440c	hypothetical protein conserved	-0.151
chr12	415398	419726	PFL0445w	hypothetical protein conserved	-0.342
chr12	421365	422195	PFL0450c	hypothetical protein conserved	-0.034
chr12	423456	426005	PFL0455c	hypothetical protein conserved	0.146
chr12	427266	427972	PFL0460w	u6 snRNA-associated Sm-like protein putative	-0.269
chr12	428489	432874	PFL0465c	Zinc finger transcription factor %28krox1%29	-0.155
chr12	436086	438464	PFL0470w	hypothetical protein conserved	-0.555
chr12	439705	442852	PFL0475w	3%27 5%27-cyclic-nucleotide phosphodiesterase putativ	0.141
chr12	443674	444981	PFL0480w	porphobilinogen deaminase putative	-0.468
chr12	445619	447262	PFL0485w	hypothetical protein conserved	0.199
chr12	447375	447944	PFL0490c	hypothetical protein conserved	0.237
chr12	448766	451333	PFL0495c	ABC transporter %28TAP family%29	-0.179
chr12	452589	453822	PFL0500w	50S ribosomal protein L1 putative	0.076
chr12	455298	456230	PFL0505c	hypothetical protein conserved	0.130
chr12	458670	461672	PFL0510c	hypothetical protein conserved	0.045
chr12	465108	465737	PFL0515w	hypothetical protein conserved	-0.221
chr12	466595	469300	PFL0520c	hypothetical protein conserved	-0.174
chr12	470222	471538	PFL0525w	hypothetical protein conserved	0.161
chr12	472253	475450	PFL0530c	hypothetical protein conserved	-0.394
chr12	476570	478363	PFL0535c	hypothetical protein conserved	0.162
chr12	480586	482452	PFL0540w	mannosyltransferase putative	-0.274
chr12	484058	489895	PFL0545w	kinesin-related protein putative	-0.146
chr12	490855	491550	PFL0550w	hypothetical protein conserved	-0.548
chr12	491886	497381	PFL0555c	hypothetical protein conserved	0.248
chr12	498518	501888	PFL0560c	minichromosome maintenance protein putative	-0.183
chr12	505338	506656	PFL0565w	heat shock protein DNAJ homologue Pfj4	-0.308
chr12	507802	508965	PFL0570c	hypothetical protein conserved	0.153
chr12	510317	519431	PFL0575w	hypothetical protein conserved	-0.031
chr12	521681	524464	PFL0580w	DNA replication licensing factor mcm5 putative	-0.631
chr12	526103	527774	PFL0585w	PfpUB Plasmodium falciparum polyubiquitin	-0.523
chr12	528999	532792	PFL0590c	non-SERCA-type Ca2%2B -transporting P-ATPase	-0.708
chr12	534906	535909	PFL0595c	glutathione peroxidase	0.178
chr12	536945	538621	PFL0600w	hypothetical protein conserved	0.191
chr12	540719	542926	PFL0605c	hypothetical protein conserved	0.448

chr12	543512	548526	PFL0610w	hypothetical protein conserved	-0.002
chr12	549882	550904	PFL0615w	hypothetical protein conserved	-0.009
chr12	552096	553847	PFL0620c	glycerol-3-phosphate acytransferase	-0.400
chr12	555764	556755	PFL0623c	hypothetical protein conserved	0.086
chr12	558040	562173	PFL0625c	eukaryotictranslation initiation factor 3 subunit 10 putativ	-0.353
chr12	565959	566924	PFL0630w	iron-sulfur subunit of succinate dehydrogenase	-0.649
chr12	568562	571828	PFL0635c	bromodomain protein putative	-0.034
chr12	576390	576641	PFL0637c	hypothetical protein pseudogene	0.625
chr12	577522	578358	PFL0640w	hypothetical protein conserved	-0.005
chr12	578463	578910	PFL0645c	hypothetical protein	-0.081
chr12	579588	581318	PFL0650c	hypothetical protein conserved	0.320
chr12	583082	585316	PFL0655w	hypothetical protein conserved	0.573
chr12	586802	587083	PFL0660w	dynein light chain 1 putative	-0.404
chr12	588121	588975	PFL0665c	RNA polymerase subunit 8c putative	-0.114
chr12	590187	592427	PFL0670c	Bi-functional aminoacyl-tRNA synthetase putative	-0.201
chr12	595160	598637	PFL0675c	hypothetical protein conserved	-0.100
chr12	600947	601557	PFL0680c	hypothetical protein conserved	-0.456
chr12	602421	606757	PFL0685w	PIG-O homolog putative	-0.044
chr12	607372	607999	PFL0690c	hypothetical protein conserved	0.084
chr12	608535	610762	PFL0695c	geranylgeranyl transferase type2 beta subunit putative	-0.251
chr12	611885	613207	PFL0700w	hypothetical protein conserved	0.037
chr12	613554	614495	PFL0705c	adrenodoxin-type ferredoxin putative	0.046
chr12	615629	616093	PFL0710w	hypothetical protein conserved	-0.027
chr12	616775	617380	PFL0715w	hypothetical protein conserved	-0.156
chr12	618757	620104	PFL0720w	hypothetical protein conserved	-0.268
chr12	621118	621768	PFL0725w	thioredoxin peroxidase 2	-0.361
chr12	623119	626266	PFL0730w	hypothetical protein conserved	0.061
chr12	627354	628196	PFL0735w	cyclophilin	-0.511
chr12	629002	629409	PFL0740c	10 kd chaperonin putative	-0.590
chr12	631154	633004	PFL0745c	hypothetical protein conserved	0.121
chr12	633885	634538	PFL0746c	conserved Plasmodium protein unknown function	0.426
chr12	635028	635450	PFL0750w	hypothetical protein conserved	-0.007
chr12	635531	638872	PFL0755c	hypothetical protein conserved	0.097
chr12	639388	640488	PFL0760w	hypothetical protein conserved	-0.081
chr12	641545	643512	PFL0765w	hypothetical protein conserved	0.077

chr12	644532	646388	PFL0770w	seryl-tRNA synthetase putative	-0.145
chr12	646972	647853	PFL0775w	hypothetical protein conserved	0.015
chr12	648882	650545	PFL0780w	glycerol-3-phosphate dehydrogenase putative	-0.539
chr12	651391	651849	PFL0785c	signal recognition particle 19 kd protein putative	-0.082
chr12	653663	656428	PFL0790w	hypothetical protein conserved	-0.039
chr12	657332	657997	PFL0795c	hypothetical protein conserved	-0.008
chr12	659731	660279	PFL0800c	hypothetical protein conserved	-0.097
chr12	663054	666275	PFL0805w	hypothetical protein conserved	0.184
chr12	670440	673259	PFL0815w	DNA-binding chaperone putative	-0.264
chr12	674342	674770	PFL0820c	hypothetical protein conserved	0.275
chr12	675449	677569	PFL0825c	hypothetical protein conserved	-0.116
chr12	678595	681954	PFL0830w	hypothetical protein conserved	-0.020
chr12	683091	685715	PFL0835w	GTP-binding protein putative	-0.335
chr12	686300	688864	PFL0840c	hypothetical protein conserved	-0.100
chr12	693060	693704	PFL0845w	hypothetical protein conserved	0.073
chr12	695552	696448	PFL0850w	anaphase promoting complex subunit 10	-0.086
chr12	696888	698450	PFL0855c	hypothetical protein conserved	-0.032
chr12	698764	699006	PFL0860c	hypothetical protein conserved	0.055
chr12	700524	702204	PFL0865w	hypothetical protein conserved	-0.048
chr12	703888	704946	PFL0870w	Plasmodium thrombospondin-related apical membrane p	-0.064
chr12	706220	707824	PFL0875w	hypothetical protein conserved	-0.065
chr12	708929	715658	PFL0880c	hypothetical protein conserved	0.180
chr12	717948	719813	PFL0885w	adaptor protein subunit puative	-0.236
chr12	720879	723441	PFL0890c	triose Pi or hexose Pi%3A Pi antiporter%3B transporter	-0.103
chr12	725834	729830	PFL0895c	hypothetical protein conserved	-0.262
chr12	732167	734952	PFL0900c	arginyl-tRNA synthetase putative	-0.118
chr12	736328	737818	PFL0905c	hypothetical protein conserved	0.072
chr12	738350	739057	PFL0910c	hypothetical protein conserved	0.104
chr12	739707	740248	PFL0915c	hypothetical protein conserved	-0.578
chr12	740987	743140	PFL0920c	hypothetical protein conserved	0.058
chr12	745995	754919	PFL0925w	Formin 2 putative	-0.127
chr12	757642	763635	PFL0930w	clathrin heavy chain putative	0.448
chr12	764441	765144	PFL0933w	PFL0933w	4.175
chr12	766647	774190	PFL0935c	erythrocyte membrane protein 1 %28PfEMP1%29	4.781
chr12	776503	779926	PFL0940c	erythrocyte membrane protein 1%28PfEMP-1%29 pseuc	4.806

chr12	783963	784823	PFL0945w	erythrocyte membrane protein 1 %28PfEMP1%29 pseud	3.841
chr12	785564	790666	PFL0950c	aminophospholipid-transporting P-ATPase	0.280
chr12	792870	793442	PFL0955c	phosphatidylethanolamine-binding protein putative	-0.310
chr12	795150	795516	PFL0957w	hypothetical protein	0.053
chr12	796493	797176	PFL0960w	D-ribulose-5-phosphate 3-epimerase putative	-0.777
chr12	798297	799604	PFL0965c	hypothetical protein conserved	-0.273
chr12	802537	804393	PFL0970w	pre-mRNA splicing factor putative	-0.284
chr12	805064	805664	PFL0972c	hypothetical protein pseudogene	0.619
chr12	805971	807014	PFL0973c	hypothetical protein	0.489
chr12	807553	816197	PFL0975w	hypothetical protein conserved	0.074
chr12	816902	818461	PFL0980w	hypothetical protein conserved	0.094
chr12	818696	819652	PFL0985c	hypothetical protein conserved	0.495
chr12	820579	822191	PFL0990w	hypothetical protein conserved	0.151
chr12	822951	824197	PFL0995c	hypothetical protein conserved	0.528
chr12	825690	827705	PFL1000c	hypothetical protein conserved	0.569
chr12	831245	832045	PFL1005c	chromodomain protein	5.009
chr12	836905	843894	PFL1010c	hypothetical protein conserved	-0.087
chr12	847430	849176	PFL1015w	hypothetical protein conserved	-0.048
chr12	849833	850645	PFL1020w	hypothetical protein conserved	-0.079
chr12	851005	854732	PFL1025c	hypothetical protein conserved	0.184
chr12	857090	858664	PFL1030w	hypothetical protein conserved	-0.674
chr12	860255	862783	PFL1035w	hypothetical protein conserved	-0.035
chr12	863803	865179	PFL1040w	hypothetical protein conserved	-0.162
chr12	865719	868896	PFL1045w	hypothetical protein conserved	-0.112
chr12	869253	871361	PFL1050w	hypothetical protein conserved	-0.224
chr12	872353	874932	PFL1055c	hypothetical protein conserved	0.004
chr12	876908	878617	PFL1060c	hypothetical protein conserved	-0.298
chr12	879622	881178	PFL1065c	hypothetical protein conserved	-0.139
chr12	883559	886024	PFL1070c	endoplasmin homolog precursor putative	-0.119
chr12	890573	898249	PFL1075w	hypothetical protein conserved	-0.132
chr12	899190	901068	PFL1080c	hypothetical protein conserved	0.161
chr12	907195	914493	PFL1085w	hypothetical protein conserved	4.872
chr12	918714	919328	PFL1090w	gliding-associated protein 45 GAP45 putative	-0.052
chr12	920782	922408	PFL1095c	hypothetical protein conserved	0.238
chr12	923519	925190	PFL1100w	hypothetical protein conserved	0.161

chr12	925367	926241	PFL1105c	hypothetical protein conserved	-0.027
chr12	927817	929983	PFL1110c	cAMP-dependent protein kinase regulatory subunit puta	0.013
chr12	932909	934273	PFL1115w	hypothetical protein conserved	-0.225
chr12	934963	938631	PFL1120c	DNA GyrAse a-subunit putative	0.014
chr12	941117	946737	PFL1125w	aminophospholipid-transporting P-ATPase	0.041
chr12	947039	960961	PFL1130c	hypothetical protein conserved	0.142
chr12	962623	967483	PFL1135c	hypothetical protein conserved	0.265
chr12	968972	970065	PFL1140w	hypothetical protein conserved	-0.100
chr12	971260	972186	PFL1145w	hypothetical protein conserved	-0.472
chr12	972702	973385	PFL1150c	ribosomal protein L24 putative	-0.350
chr12	974364	975533	PFL1155w	GTP cyclohydrolase I	-0.269
chr12	976812	977881	PFL1160c	hypothetical protein conserved	-0.268
chr12	981293	984301	PFL1165w	hypothetical protein conserved	0.133
chr12	988620	991247	PFL1170w	polyadenylate-binding protein putative	-0.418
chr12	994247	996430	PFL1175w	hypothetical protein conserved	0.057
chr12	998345	999267	PFL1180w	Chromatin assembly protein %28ASF1%29 putative	-0.219
chr12	1000671	1001937	PFL1185c	cytochrome c heme lyase putative	-0.163
chr12	1003193	1007578	PFL1190c	hypothetical protein conserved	0.039
chr12	1009281	1010243	PFL1195w	hypothetical protein conserved	-0.346
chr12	1010824	1011334	PFL1200c	hypothetical protein conserved	-0.105
chr12	1012302	1017840	PFL1205c	hypothetical protein conserved	0.300
chr12	1018498	1024000	PFL1210w	hypothetical protein conserved	0.004
chr12	1024447	1028010	PFL1215c	hypothetical protein conserved	0.107
chr12	1029854	1031380	PFL1220w	hypothetical protein conserved	-0.388
chr12	1031817	1032557	PFL1225c	hypothetical protein conserved	0.080
chr12	1033543	1035104	PFL1230w	hypothetical protein conserved	0.525
chr12	1035253	1038146	PFL1235c	hypothetical protein conserved	0.006
chr12	1039401	1043906	PFL1240c	hypothetical protein conserved	0.042
chr12	1045986	1049822	PFL1245w	ubiquitin-activating enzyme e1 putative	-0.306
chr12	1050271	1052391	PFL1250c	hypothetical protein conserved	0.192
chr12	1053287	1056273	PFL1255c	hypothetical protein conserved	0.025
chr12	1058193	1059143	PFL1260w	hydrolase %2F phosphatase putative	-0.437
chr12	1059709	1061460	PFL1265c	hypothetical protein conserved	0.083
chr12	1065787	1066674	PFL1270w	cof-like hydrolase had-superfamily subfamily iib	-0.434
chr12	1067837	1071300	PFL1275c	hypothetical protein conserved	-0.089

chr12	1072450	1076967	PFL1280w	hypothetical protein conserved	-0.097
chr12	1077859	1078767	PFL1285c	proliferating cell nuclear antigen putative	-0.157
chr12	1081189	1083099	PFL1290w	U3 snoRNP associated protein putative	-0.365
chr12	1085378	1087255	PFL1295w	hypothetical protein conserved	-0.166
chr12	1088527	1090014	PFL1300c	hypothetical protein conserved	-0.406
chr12	1092151	1092975	PFL1305c	hypothetical protein conserved	-0.090
chr12	1093534	1095762	PFL1310c	ATP-dependent RNA helicase putative	-0.405
chr12	1098120	1104059	PFL1315w	K%2B channel potassium channel	-0.113
chr12	1105242	1108730	PFL1320w	hypothetical protein conserved	0.083
chr12	1109142	1112660	PFL1325c	hypothetical protein conserved	0.129
chr12	1114016	1121084	PFL1330c	hypothetical protein conserved	-0.117
chr12	1124779	1128331	PFL1335w	hypothetical protein conserved	-0.350
chr12	1128930	1131677	PFL1340c	hypothetical protein conserved	-0.103
chr12	1134361	1137639	PFL1345c	radical SAM protein putative	-0.278
chr12	1138599	1140293	PFL1350w	RNA pseudouridylate synthase putative	0.022
chr12	1140934	1141377	PFL1355w	hypothetical protein conserved	-0.513
chr12	1141574	1143185	PFL1360c	hypothetical protein	-0.032
chr12	1143651	1146107	PFL1365w	hypothetical protein conserved	-0.081
chr12	1148102	1151452	PFL1370w	NIMA-related protein kinase Pfnek-1	-0.609
chr12	1153343	1157548	PFL1375w	hypothetical protein conserved	-0.049
chr12	1158545	1160053	PFL1380w	hypothetical protein conserved	-0.022
chr12	1160720	1162951	PFL1385c	Merozoite Surface Protein 9 MSP-9	0.130
chr12	1165499	1169139	PFL1390w	hypothetical protein conserved	0.038
chr12	1169548	1179177	PFL1395c	hypothetical protein conserved	-0.073
chr12	1180660	1185473	PFL1400c	hypothetical protein conserved	0.056
chr12	1186405	1191909	PFL1405w	hypothetical protein conserved	0.171
chr12	1192878	1199204	PFL1410c	ABC transporter %28CT family%29	-0.247
chr12	1204981	1207953	PFL1415w	hypothetical protein conserved	-0.129
chr12	1208902	1209532	PFL1420w	macrophage migration inhibitory factor homolog putative	-0.280
chr12	1211778	1213553	PFL1425w	t-complex protein 1 gamma subunit putative	-0.493
chr12	1214307	1217705	PFL1430c	hypothetical protein conserved	0.141
chr12	1218171	1218756	PFL1432w	conserved Plasmodium protein unknown function	0.442
chr12	1219596	1226455	PFL1435c	myosin d	-0.187
chr12	1228053	1232995	PFL1440c	hypothetical protein conserved	0.089
chr12	1233686	1241277	PFL1445w	hypothetical protein conserved	0.038

chr12	1242015	1243589	PFL1450c	hypothetical protein conserved	-0.276	
chr12	1245525	1249160	PFL1455w	hypothetical protein conserved	0.078	
chr12	1249758	1250647	PFL1460c	hypothetical protein conserved	-0.324	
chr12	1251904	1252815	PFL1465c	heat shock protein hslv putative	-0.335	
chr12	1253963	1255471	PFL1470c	hypothetical protein conserved	-0.284	
chr12	1256680	1258094	PFL1475w	sun-family protein putative	-0.447	
chr12	1259610	1262078	PFL1480w	cop-II coated vesicle component sec13p	-0.541	
chr12	1263651	1265583	PFL1485w	hypothetical protein conserved	-0.079	
chr12	1267358	1270156	PFL1490w	serine%2Fthreonine protein kinase putative	-0.125	
chr12	1271130	1274845	PFL1495w	hypothetical protein conserved	0.123	
chr12	1276247	1277724	PFL1500w	Rab2 GTPase	0.038	
chr12	1278450	1279601	PFL1505c	hypothetical protein conserved	0.842	12cenL
chr12	1286488	1289346	PFL1510c	hypothetical protein conserved	0.764	12cenR
chr12	1290675	1297059	PFL1515c	amino acid transporter	0.405	
chr12	1299599	1300018	PFL1520w	dim1 protein homolog putative	-0.187	
chr12	1301089	1304595	PFL1525c	pre-mRNA splicing factor RNA helicase putative	-0.248	
chr12	1306515	1306959	PFL1526c	PFL1526c	0.167	
chr12	1314687	1320161	PFL1530w	asparagine-rich protein putative	-0.104	
chr12	1321971	1325231	PFL1535w	hypothetical protein conserved	0.318	
chr12	1325741	1327330	PFL1540c	phenylalanyl-tRNA synthetase alpha chain putative	0.173	
chr12	1328302	1331362	PFL1545c	chaperonin cpn60	-0.168	
chr12	1333391	1334887	PFL1550w	lipoamide dehydrogenase	-0.621	
chr12	1336262	1337203	PFL1555w	cytochrome b5 putative	0.085	
chr12	1337700	1338806	PFL1560c	hypothetical protein conserved	-0.018	
chr12	1340170	1343303	PFL1565c	hypothetical protein conserved	-0.144	
chr12	1344252	1345340	PFL1570w	hypothetical protein conserved	0.069	
chr12	1346041	1347128	PFL1575w	hypothetical protein conserved	-0.214	
chr12	1347637	1350502	PFL1580w	hypothetical protein conserved	-0.041	
chr12	1350899	1351273	PFL1583c	hypothetical protein conserved	0.347	
chr12	1351779	1354094	PFL1585c	hypothetical protein conserved	0.127	
chr12	1355307	1357718	PFL1590c	elongation factor g putative	-0.058	
chr12	1359159	1359566	PFL1595w	hypothetical protein conserved	-0.384	
chr12	1360621	1370626	PFL1600c	hypothetical protein conserved	1.027	
chr12	1375335	1379576	PFL1605w	hypothetical protein conserved	-0.107	
chr12	1380521	1382367	PFL1610c	hypothetical protein conserved	-0.025	

chr12	1385357	1385497	PFL1615c	hypothetical protein	0.232
chr12	1385695	1402206	PFL1620w	asparagine%2Faspartate rich protein putative	-0.136
chr12	1403127	1404872	PFL1625w	hypothetical protein conserved	0.014
chr12	1405170	1405956	PFL1630c	hypothetical protein conserved	-0.246
chr12	1407662	1410873	PFL1635w	SUMO-deconjugating enzyme putative	-0.035
chr12	1411595	1413092	PFL1640c	hypothetical protein conserved	0.089
chr12	1414402	1426092	PFL1645w	hypothetical protein conserved	0.026
chr12	1427386	1433588	PFL1650w	hypothetical protein conserved	0.049
chr12	1433808	1435983	PFL1655c	epsilon DNA polymerase putative	-0.143
chr12	1436969	1439337	PFL1660c	hypothetical protein conserved	-0.171
chr12	1439884	1441038	PFL1665c	hypothetical protein conserved	0.406
chr12	1441724	1445116	PFL1670c	hypothetical protein conserved	0.230
chr12	1446363	1450323	PFL1675c	hypothetical protein conserved	-0.266
chr12	1452594	1457215	PFL1680w	splicing factor 3b subunit 3 130kD putative	-0.574
chr12	1458221	1459170	PFL1685w	hypothetical protein conserved	-0.681
chr12	1459821	1461473	PFL1690w	hypothetical protein conserved	-0.613
chr12	1461854	1462589	PFL1695c	hypothetical protein conserved	0.131
chr12	1464425	1467559	PFL1700c	V-type K%2B - independent h%2B -translocating inorgar	-0.463
chr12	1470700	1475688	PFL1705w	hypothetical protein conserved	-0.161
chr12	1476516	1480001	PFL1710c	tetQ family GTPase putative	0.127
chr12	1480989	1482929	PFL1715w	hypothetical protein conserved	-0.495
chr12	1484294	1485899	PFL1720w	Serine hydroxymethyltransferase	-0.792
chr12	1487072	1488679	PFL1725w	ATP synthase beta chain mitochondrial precursor putati	-0.992
chr12	1489307	1497053	PFL1730c	hypothetical protein conserved	0.138
chr12	1497675	1501399	PFL1735c	hypothetical protein conserved	0.158
chr12	1502824	1504056	PFL1740w	hypothetical protein conserved	-0.257
chr12	1506920	1508549	PFL1745c	clustered-asparagine-rich protein	-0.147
chr12	1512189	1516505	PFL1750c	hypothetical protein conserved	-0.268
chr12	1520316	1521593	PFL1755w	hypothetical protein conserved	0.078
chr12	1522510	1524621	PFL1760w	hypothetical protein conserved	0.245
chr12	1525396	1526205	PFL1765c	hypothetical protein conserved	-0.158
chr12	1527444	1528023	PFL1770c	hypothetical protein conserved	-0.860
chr12	1529810	1531336	PFL1775c	hypothetical protein conserved	-0.203
chr12	1532237	1534140	PFL1780w	protein-S-isoprenylcysteine O-methyltransferase putative	0.178
chr12	1534508	1535868	PFL1785c	hypothetical protein conserved	-0.006

chr12	1538127	1540397	PFL1790w	ubiquitin activating enzyme putative	-0.180
chr12	1540755	1549133	PFL1795c	hypothetical protein conserved	0.081
chr12	1550313	1556060	PFL1800w	hypothetical protein conserved	0.061
chr12	1556288	1557606	PFL1805c	hypothetical protein conserved	0.016
chr12	1558898	1560612	PFL1810w	hypothetical protein conserved	-0.234
chr12	1561523	1566568	PFL1815c	hypothetical protein conserved	0.052
chr12	1568595	1570558	PFL1820w	hypothetical protein conserved	-0.132
chr12	1572360	1573546	PFL1825w	hypothetical protein conserved	-0.048
chr12	1575186	1575507	PFL1830w	ubiquitin-like protein putative	-0.009
chr12	1577170	1580736	PFL1835w	hypothetical protein	-0.369
chr12	1581973	1582729	PFL1840w	hypothetical protein conserved	-0.037
chr12	1583612	1584584	PFL1845c	calcyclin binding protein putative	-0.412
chr12	1586250	1587455	PFL1850c	hypothetical protein conserved	0.275
chr12	1589158	1592061	PFL1855w	cell cycle control protein putative	-0.227
chr12	1592407	1593621	PFL1860c	hypothetical protein conserved	0.256
chr12	1594815	1600515	PFL1865w	hypothetical protein conserved	-0.141
chr12	1601504	1602685	PFL1870c	sphingomyelin phosphodiesterase	-0.465
chr12	1605096	1607767	PFL1875w	hypothetical protein conserved	0.166
chr12	1610352	1612730	PFL1880w	acetyl-CoA synthetase PfACS11	-0.285
chr12	1615312	1616841	PFL1885c	calcium%2Fcalmodulin-dependent protein kinase 2 puta	-0.293
chr12	1620877	1623694	PFL1890c	hypothetical protein conserved	-0.297
chr12	1626762	1627917	PFL1895w	ribosomal protein L23 putative	-0.400
chr12	1634684	1642417	PFL1900w	hypothetical protein conserved	-0.015
chr12	1645208	1645825	PFL1905w	hypothetical protein conserved	0.281
chr12	1646601	1647140	PFL1910c	hypothetical protein conserved	0.317
chr12	1648656	1651676	PFL1915w	dna gyrase subunit b putative	-0.268
chr12	1652925	1653833	PFL1920c	hydroxyethylthiazole kinase putative	-0.497
chr12	1655476	1658118	PFL1925w	cell division protein FtsH putative	-0.524
chr12	1660440	1677743	PFL1930w	hypothetical protein conserved	0.227
chr12	1678427	1681525	PFL1935c	hypothetical protein conserved	0.156
chr12	1682824	1684449	PFL1940w	3-hydroxyisobutyryl-coenzyme A hydrolase putative	0.100
chr12	1685083	1685400	PFL1945c	early transcribed membrane protein 12 ETRAMP12	0.083
chr12	1688595	1691151	PFL1947c	VAR pseudogene erythrocyte membrane protein 1 %28I	4.039
chr12	1694139	1703076	PFL1950w	erythrocyte membrane protein 1 %28PfEMP1%29	4.742
chr12	1704501	1712479	PFL1955w	erythrocyte membrane protein 1 %28PfEMP1%29	4.850

chr12	1719563	1727445	PFL1960w	erythrocyte membrane protein 1 %28PfEMP1%29	4.753
chr12	1728763	1729755	PFL1965w	rifin pseudogene	5.145
chr12	1735532	1743396	PFL1970w	erythrocyte membrane protein 1 %28PfEMP1%29	4.772
chr12	1746432	1747981	PFL1975c	hypothetical protein conserved	0.148
chr12	1748861	1756225	PFL1980c	hypothetical protein conserved	0.352
chr12	1757505	1759187	PFL1985c	hypothetical protein conserved	-0.008
chr12	1760404	1766379	PFL1990c	hypothetical protein conserved	0.066
chr12	1771984	1773246	PFL1995c	hypothetical protein conserved	0.206
chr12	1774889	1776570	PFL2000w	mitochondrial carrier protein putative	0.327
chr12	1778142	1779399	PFL2005w	replication factor c subunit 4	-0.427
chr12	1779912	1782636	PFL2010c	DEAD%2FDEAH box helicase putative	-0.092
chr12	1784368	1786398	PFL2015w	hypothetical protein conserved	0.104
chr12	1787224	1788819	PFL2020c	hypothetical protein conserved	0.094
chr12	1790545	1790751	PFL2025w	hypothetical protein conserved	0.197
chr12	1791261	1793063	PFL2030w	queuine tRNA ribosyltransferase putative	-0.118
chr12	1793520	1794823	PFL2035c	hypothetical protein conserved	-0.148
chr12	1795833	1797848	PFL2040w	hypothetical protein conserved	0.218
chr12	1798631	1799776	PFL2045w	hypothetical protein conserved	0.057
chr12	1800550	1802043	PFL2050w	protein geranylgeranyltransferase type II alpha subunit	0.006
chr12	1803641	1804471	PFL2055w	40S ribosomal protein S17 putative	-0.083
chr12	1805368	1807344	PFL2060c	rabGDI protein	-0.556
chr12	1808866	1809610	PFL2065c	mitochondrial import inner membrane translocase subun	-0.112
chr12	1811778	1812686	PFL2070w	t-SNARE putative	0.044
chr12	1813495	1814223	PFL2075c	hypothetical protein conserved	-0.545
chr12	1815299	1815550	PFL2078w	conserved Plasmodium protein unknown function	0.194
chr12	1816057	1816436	PFL2080c	hypothetical protein conserved	0.019
chr12	1818726	1820057	PFL2085w	hypothetical protein	0.117
chr12	1820869	1822583	PFL2090c	P. falciparum homologue of yeast snf7	0.111
chr12	1825947	1826872	PFL2095w	Translation initiation factor SUI1 putative	0.041
chr12	1829649	1831046	PFL2100w	hypothetical protein conserved	-0.242
chr12	1831473	1832938	PFL2105c	hypothetical protein conserved	-0.470
chr12	1834053	1840725	PFL2110c	hypothetical protein conserved	0.012
chr12	1842388	1845640	PFL2115c	glucose inhibited division protein A homologue putative	-0.068
chr12	1847666	1852145	PFL2120w	hypothetical protein conserved	-0.005
chr12	1852833	1855670	PFL2125c	hypothetical protein conserved	-0.100

chr12	1859645	1863835	PFL2130w	hypothetical protein conserved	0.042
chr12	1864218	1866650	PFL2135c	hypothetical protein conserved	-0.131
chr12	1868522	1869520	PFL2140c	ADP-ribosylation factor GTPase-activating protein	-0.203
chr12	1871908	1872800	PFL2145w	hypothetical protein conserved	0.075
chr12	1873183	1875276	PFL2150c	hypothetical protein conserved	-0.063
chr12	1877028	1879834	PFL2155w	hypothetical protein conserved	0.391
chr12	1880025	1881395	PFL2160c	hypothetical protein conserved	0.087
chr12	1883458	1887670	PFL2165w	kinesin-like protein putative	-0.197
chr12	1888239	1892687	PFL2170c	hypothetical protein conserved	0.193
chr12	1893058	1893892	PFL2175w	ubiquitin-conjugating enzyme e2 putative	-0.307
chr12	1894931	1895956	PFL2180w	50S ribosomal protein L3 putative	-0.210
chr12	1897255	1899827	PFL2185w	hypothetical protein conserved	0.026
chr12	1900239	1905056	PFL2190c	hypothetical protein conserved	0.186
chr12	1908524	1909753	PFL2195w	clathrin coat assembly protein AP180 putative	-0.125
chr12	1910952	1911887	PFL2200w	hypothetical protein conserved	0.000
chr12	1912357	1914582	PFL2205w	hypothetical protein conserved	-0.019
chr12	1915736	1917785	PFL2210w	delta-aminolevulinic acid synthetase	-0.191
chr12	1920771	1921901	PFL2215w	actin	-0.619
chr12	1925629	1929425	PFL2220w	hypothetical protein conserved	-0.264
chr12	1931957	1932571	PFL2225w	myosin A tail domain interacting protein MTIP putative	-0.544
chr12	1933748	1935454	PFL2230c	hypothetical protein conserved	0.018
chr12	1936509	1937681	PFL2235w	hypothetical protein conserved	-0.201
chr12	1938630	1939952	PFL2240w	hypothetical protein conserved	-0.118
chr12	1941352	1942478	PFL2245w	signal recognition particle beta subunit putative	-0.015
chr12	1943345	1945790	PFL2250c	rac-beta serine%2Fthreonine protein kinase PfPKB	-0.149
chr12	1946901	1948720	PFL2255w	hypothetical protein conserved	0.002
chr12	1949581	1950480	PFL2260w	hypothetical protein conserved	0.033
chr12	1950976	1951500	PFL2265c	hypothetical protein conserved	0.364
chr12	1952555	1954066	PFL2270w	mannosyl transferase putative	-0.157
chr12	1955111	1956025	PFL2275c	FK506-binding protein %28FKBP%29-type peptidyl-prop	-0.488
chr12	1959599	1962328	PFL2280w	cyclin g-associated kinase putative	-0.069
chr12	1962761	1966012	PFL2285c	hypothetical protein conserved	-0.019
chr12	1968494	1971061	PFL2290w	preprocathepsin c precursor putative	-0.304
chr12	1972425	1973063	PFL2295w	hypothetical protein conserved	-0.225
chr12	1973529	1974233	PFL2300w	hypothetical protein conserved	-0.066

chr12	1975159	1976424	PFL2305w	hypothetical protein conserved	-0.369
chr12	1977852	1978925	PFL2310w	hypothetical protein conserved	-0.488
chr12	1979773	1980186	PFL2315c	hypothetical protein conserved	-0.261
chr12	1981893	1982750	PFL2320w	hypothetical protein conserved	0.081
chr12	1983467	1984336	PFL2325c	P. falciparum homologue of Drosophila nmda1 protein p	-0.717
chr12	1988224	1990732	PFL2330w	hypothetical protein conserved	-0.139
chr12	1994762	2000731	PFL2335w	hypothetical protein conserved	0.000
chr12	2001974	2002258	PFL2340w	hypothetical protein conserved	-0.083
chr12	2002710	2004017	PFL2345c	tat-binding protein homolog	-0.776
chr12	2005162	2007180	PFL2350c	hypothetical protein conserved	0.329
chr12	2008181	2009695	PFL2355w	hypothetical protein conserved	-0.521
chr12	2010415	2010876	PFL2360w	hypothetical protein	0.257
chr12	2012630	2015713	PFL2365w	hypothetical protein conserved	-0.246
chr12	2016312	2016743	PFL2370c	hypothetical protein conserved	-0.202
chr12	2017351	2018456	PFL2375c	cutA putative	0.236
chr12	2019241	2021994	PFL2380c	leucine-rich repeat protein 12 LRR12	0.176
chr12	2023442	2026167	PFL2385c	hypothetical protein conserved	0.322
chr12	2029990	2039838	PFL2390c	hypothetical protein conserved	0.085
chr12	2041279	2043198	PFL2395c	dimethyladenosine transferase putative	-0.056
chr12	2044106	2044612	PFL2400w	hypothetical protein conserved	-0.861
chr12	2045056	2054415	PFL2405c	PFG377 protein	1.307
chr12	2059093	2063212	PFL2410w	hypothetical protein conserved	-0.317
chr12	2064972	2066025	PFL2415w	Hbeta58%2FVps26 protein homolog putative	-0.620
chr12	2067385	2069355	PFL2420w	hypothetical protein conserved	0.151
chr12	2070079	2071058	PFL2425w	adaptor-related protein complex 3 sigma 2 subunit puta	0.249
chr12	2071587	2073626	PFL2430c	eukaryotictranslation initiation factor 2b subunit 2 putati	-0.240
chr12	2075777	2076502	PFL2435w	hypothetical protein conserved	-0.087
chr12	2077703	2082872	PFL2440w	DNA repair protein rhp16 putative	-0.336
chr12	2083338	2084303	PFL2445c	hypothetical protein conserved	0.396
chr12	2085355	2086676	PFL2450c	hypothetical protein conserved	0.302
chr12	2089470	2090206	PFL2455w	hypothetical protein conserved	0.065
chr12	2092073	2094217	PFL2460w	coronin	-0.426
chr12	2095048	2096494	PFL2465c	thymidylate kinase putative	-0.285
chr12	2097422	2098831	PFL2470c	hypothetical protein conserved	-0.016
chr12	2099838	2102083	PFL2475w	DEAD%2FDEAH box helicase putative	-0.256

chr12	2103103	2105241	PFL2480w	hypothetical protein conserved	-0.003
chr12	2106330	2108279	PFL2485c	tryptophanyl-tRNA synthetase putative	0.183
chr12	2108946	2110913	PFL2490c	hypothetical protein conserved	0.258
chr12	2114614	2122473	PFL2505c	hypothetical protein conserved	0.042
chr12	2125421	2126557	PFL2510w	chitinase	-0.407
chr12	2128386	2128772	PFL2515c	hypothetical protein conserved	-0.348
chr12	2136038	2144859	PFL2520w	Plasmodium falciparum reticulocyte binding-like protein	2.753
chr12	2145251	2146601	PFL2525c	hypothetical protein	0.415
chr12	2150539	2151900	PFL2530w	hypothetical protein conserved	-0.093
chr12	2154603	2156109	PFL2535w	RESA-like protein putative	-0.112
chr12	2159558	2161600	PFL2540w	hypothetical protein conserved in P. falciparum	0.031
chr12	2162692	2162919	PFL2545c	hypothetical protein	-0.153
chr12	2168231	2169744	PFL2550w	hypothetical protein conserved in P. falciparum	0.358
chr12	2171326	2172333	PFL2555w	hypothetical protein conserved in P. falciparum	0.386
chr12	2173783	2174109	PFL2560c	hypothetical protein conserved in P. falciparum	0.455
chr12	2175313	2176071	PFL2565w	hypothetical protein consevred in P. falciparum	0.052
chr12	2188744	2189594	PFL2580w	rifin pseudogene	5.338
chr12	2190467	2191754	PFL2585c	rifin	5.155
chr12	2196141	2197106	PFL2590w	hypothetical protein onserved in P. falciparum	2.623
chr12	2198910	2199541	PFL2595w	hypothetical protein conserved in P. falciparum	4.684
chr12	2201479	2202670	PFL2605w	rifin	4.919
chr12	2204708	2205698	PFL2610w	stevor	5.288
chr12	2207814	2209099	PFL2615w	rifin	5.382
chr12	2211548	2212538	PFL2620w	stevor	5.187
chr12	2214655	2216003	PFL2625w	rifin	4.953
chr12	2218436	2219517	PFL2630w	rifin	5.172
chr12	2221840	2222832	PFL2635w	stevor	5.026
chr12	2224981	2226185	PFL2640c	rifin	5.028
chr12	2228616	2229724	PFL2645c	rifin	5.183
chr12	2234584	2235776	PFL2655w	rifin	4.929
chr12	2238194	2239401	PFL2660w	rifin	4.406
chr12	2241255	2248946	PFL2665c	erythrocyte membrane protein 1 %28PfEMP1%29	4.688
chr13	21467	28890	MAL13P1.1	erythrocyte membrane protein 1 %28PfEMP1%29	3.532
chr13	30708	31984	MAL13P1.2	rifin	4.327
chr13	34062	44845	PF13_0003	erythrocyte membrane protein 1 %28PfEMP1%29	5.259

chr13	47686	48872	PF13_0004	rifin	5.295
chr13	50765	51960	PF13_0005	rifin	4.420
chr13	54170	55362	PF13_0006	rifin	4.142
chr13	57212	58525	MAL13P1.4	rifin	5.002
chr13	60200	61275	MAL13P1.6	erythrocyte membrane protein 1-like	5.286
chr13	62618	63650	MAL13P1.7	stevor	5.418
chr13	65651	66919	MAL13P1.8	RIF pseudogene	4.970
chr13	73202	74516	PF13_0010	Gbph2	4.580
chr13	78891	79681	MAL13P1.58	hypothetical protein conserved in P. falciparum	3.551
chr13	82821	84121	PF13_0073	hypothetical protein conserved in P. falciparum	0.619
chr13	85666	86727	MAL13P1.59	hypothetical protein conserved in P. falciparum	0.466
chr13	89422	93455	MAL13P1.60	erythrocyte binding antigen 140	0.183
chr13	99651	100624	MAL13P1.61	hypothetical protein conserved in P. falciparum	0.051
chr13	102945	108482	PF13_0075	surface-associated interspersed gene 13.1 %28SURFIN	1.288
chr13	109798	110371	MAL13P1.62	hypothetical protein	0.180
chr13	112895	113918	PF13_0076	hypothetical protein conserved	0.443
chr13	121840	122493	PF13_0011	plasmodium falciparum gamete antigen 27%2F25	-0.315
chr13	123930	124619	PF13_0012	early transcribed membrane protein 13 ETRAMP13	-0.231
chr13	129891	131288	MAL13P1.12	hypothetical protein conserved	0.117
chr13	132051	140796	MAL13P1.13	hypothetical protein conserved	-0.041
chr13	143062	144297	PF13_0013	PBS lyase HEAT-like repeat domain protein	-0.272
chr13	145376	150400	MAL13P1.14	ATP-dependent DEAD box helicase putative	-0.175
chr13	150985	151837	PF13_0014	40S ribosomal protein S7 homologue putative	0.070
chr13	155194	157596	PF13_0015	hypothetical protein conserved	-0.130
chr13	158346	158980	MAL13P1.15	hypothetical protein conserved	-0.738
chr13	159226	160117	PF13_0016	methyl transferase-like protein putative	0.179
chr13	160690	161862	MAL13P1.16	synaptobrevin-like protein putative	-0.001
chr13	163119	164081	PF13_0017	hypothetical protein conserved	0.235
chr13	165215	168283	PF13_0018	hypothetical protein conserved	-0.201
chr13	170076	175991	PF13_0019	sodium%2Fhydrogen exchanger Na%2B H%2B antipor	-0.319
chr13	178985	179555	MAL13P1.17	hypothetical protein conserved	0.399
chr13	180751	184719	MAL13P1.18	hypothetical protein conserved	-0.016
chr13	185047	212862	MAL13P1.19	peptidase putative	0.009
chr13	215493	216533	MAL13P1.20	hypothetical protein conserved	-0.480
chr13	216854	220231	MAL13P1.21	hypothetical protein conserved	-0.381

chr13	222018	224922	MAL13P1.22	DNA ligase 1	-0.423
chr13	226887	228492	MAL13P1.23	hypothetical protein conserved	-0.033
chr13	231285	231926	MAL13P1.24	hypothetical protein conserved	-0.026
chr13	232695	234649	PF13_0020	hypothetical protein conserved	-0.107
chr13	236002	236637	PF13_0021	small heat shock protein putative	-0.453
chr13	237563	241435	MAL13P1.25	hypothetical protein conserved	0.174
chr13	241859	243164	PF13_0022	cyclin 4	0.046
chr13	245401	246377	PF13_0023	DNA-directed RNA polymerase 2 putative	0.163
chr13	247514	251953	PF13_0024	hypothetical protein conserved	-0.192
chr13	252728	253990	PF13_0025	hypothetical protein conserved	0.052
chr13	255332	256318	PF13_0026	hypothetical protein	-0.054
chr13	256632	267384	MAL13P1.26	hypothetical protein conserved	0.148
chr13	268987	270939	MAL13P1.28	hypothetical protein conserved	-0.098
chr13	271613	273928	PF13_0027	hypothetical protein conserved	0.261
chr13	274275	275402	PF13_0028	membrane integral peptidase M50 family putative	-0.101
chr13	277341	277931	PF13_0029	hypothetical protein conserved	-0.791
chr13	278496	279335	PF13_0030	hypothetical protein conserved	0.062
chr13	280192	281903	PF13_0031	tRNA intron exonumclease putative	-0.381
chr13	283677	287702	MAL13P1.29	hypothetical protein conserved	0.060
chr13	288365	288821	MAL13P1.30	hypothetical protein	0.098
chr13	289766	291202	PF13_0032	hydrolase putative	0.150
chr13	293644	296140	MAL13P1.31	hypothetical protein conserved	-0.193
chr13	297380	298561	PF13_0033	26S proteasome regulatory subunit putative	-0.681
chr13	301190	314131	MAL13P1.32	hypothetical protein conserved	0.331
chr13	315244	316701	PF13_0034	vacuolar ATP synthase subunit h putative	-0.057
chr13	318534	319937	MAL13P1.33	hypothetical protein conserved	0.222
chr13	321017	322219	MAL13P1.34	hypothetical protein conserved	0.130
chr13	323675	325438	MAL13P1.35	u1 small nuclear ribonucleoprotein a putative	-0.502
chr13	325418	326574	MAL13P1.36	hypothetical protein conserved	0.276
chr13	328096	331131	PF13_0035	hypothetical protein conserved	0.210
chr13	331761	332729	PF13_0036	DNAJ protein putative	0.075
chr13	333800	335692	PF13_0037	DEAD box helicase putative	-0.304
chr13	336055	337466	PF13_0038	hypothetical protein conserved	-0.059
chr13	338392	338658	PF13_0039	hypothetical protein conserved	0.174
chr13	340130	342091	PF13_0040	DNA-directed RNA polymerase alpha chain putative	-0.476

chr13	344728	348435	PF13_0041	hypothetical protein conserved	-0.240
chr13	348946	350991	PF13_0042	fork head domain protein putative	-0.176
chr13	352043	355021	MAL13P1.37	hypothetical protein conserved	0.148
chr13	356912	359404	MAL13P1.38	hypothetical protein conserved	-0.036
chr13	360372	360830	PF13_0043	CCAAT-binding transcription factor putative	-0.486
chr13	361369	368698	PF13_0044	carbamoyl phosphate synthetase putative	-0.245
chr13	372463	372849	PF13_0045	40S ribosomal protein S27 putative	0.232
chr13	373690	392275	MAL13P1.39	hypothetical protein conserved	0.168
chr13	394060	395139	MAL13P1.40	Proteinase inhibitor protein putative	0.113
chr13	396937	401154	MAL13P1.41	hypothetical protein conserved	0.269
chr13	401780	403603	PF13_0047	hypothetical protein conserved	-0.097
chr13	403991	407046	MAL13P1.42	recombinase putative	0.040
chr13	408783	412304	PF13_0048	NUDIX hydrolase putative	-0.545
chr13	416120	417202	MAL13P1.43	hypothetical protein conserved	-0.184
chr13	419024	420325	PF13_0049	60S ribosomal protein L24 putative	-0.257
chr13	424433	427237	MAL13P1.44	protein phosphatase 2c-like protein putative	-0.184
chr13	427658	429844	MAL13P1.45	U4%2FU6 small nuclear ribonuclear protein putative	-0.181
chr13	432094	436077	PF13_0050	hypothetical protein conserved	-0.410
chr13	436878	437507	PF13_0051	snornp protein gar1 homologue putative	0.374
chr13	438712	439578	PF13_0052	ribosomal RNA methyltransferase putative	-0.154
chr13	440467	445485	PF13_0053	hypothetical protein conserved	-0.177
chr13	445813	446860	PF13_0054	transcription factor putative	-0.527
chr13	447626	448303	MAL13P1.46	hypothetical protein conserved	0.096
chr13	449109	449870	MAL13P1.47	mitochondrial ATP synthase delta subunit putative	0.260
chr13	450618	453617	MAL13P1.48	hypothetical protein conserved	0.053
chr13	454263	454879	MAL13P1.49	hypothetical protein conserved	-0.179
chr13	455994	456737	PF13_0055	hypothetical protein conserved	-0.122
chr13	459163	462570	PF13_0056	hypothetical protein conserved	0.033
chr13	463587	464204	MAL13P1.50	hypothetical protein conserved	-0.187
chr13	465293	466754	MAL13P1.51	Rab5b GTPase	-0.310
chr13	468434	468865	PF13_0058	RNA-binding protein putative	-0.626
chr13	470797	475204	MAL13P1.52	hypothetical protein conserved	-0.216
chr13	476425	477336	PF13_0059	ribosomal protein S15 mitochondrial precursor putative	-0.782
chr13	477626	478054	MAL13P1.53	hypothetical protein conserved	0.378
chr13	479555	481042	PF13_0060	hypothetical protein conserved	0.086

chr13	481701	483284	MAL13P1.54	hypothetical protein conserved	-0.142
chr13	484407	485665	PF13_0061	ATP synthase gamma chain mitochondrial precursor pu	-0.266
chr13	487713	489026	PF13_0062	clathrin-adaptor medium chain putative	-0.231
chr13	490129	491811	PF13_0063	26S proteasome regulatory subunit 7 putative	-0.772
chr13	494655	496496	PF13_0064	hypothetical protein conserved	-0.208
chr13	497736	498215	MAL13P1.55	cytochrome c2 precursor putative	-0.696
chr13	501992	505249	MAL13P1.56	m1-family aminopeptidase	-0.363
chr13	508102	509937	PF13_0065	vacuolar ATP synthase catalytic subunit a	-0.967
chr13	511598	512809	PF13_0066	malonyl coa-acyl carrier protein transacylase precursor	-0.253
chr13	513270	517528	PF13_0067	hypothetical protein conserved	0.116
chr13	518309	519569	MAL13P1.57	hypothetical protein conserved	0.426
chr13	519801	520643	PF13_0068	hypothetical protein conserved	-0.289
chr13	521252	524638	PF13_0069	translation initiation factor if-2 putative	-0.186
chr13	530178	531467	PF13_0070	branched-chain alpha keto-acid dehydrogenase putative	-0.625
chr13	532472	534160	PF13_0071	hypothetical protein	0.211
chr13	536904	544725	PF13_0072	hypothetical protein conserved	-0.060
chr13	549465	553043	MAL13P1.63	Plasmodium falciparum asparagine-rich protein	-0.006
chr13	553954	554453	MAL13P1.64	ubiquitin-like protein nedd8 homologue putative	-0.255
chr13	555769	556044	MAL13P1.65	hypothetical protein	-0.189
chr13	556487	565189	MAL13P1.66	hypothetical protein conserved	0.172
chr13	566438	568435	MAL13P1.67	methionyl-tRNA formyltransferase putative	0.293
chr13	568866	569737	MAL13P1.68	peptidyl-prolyl cis-trans isomerase putative	-0.031
chr13	570042	572534	PF13_0077	DEAD box helicase putative	0.142
chr13	573371	578681	PF13_0078	hypothetical protein conserved	0.186
chr13	579728	585817	PF13_0079	hypothetical protein conserved	-0.031
chr13	586792	588011	MAL13P1.69	septum formation protein MAF homologue putative	0.114
chr13	589002	599400	MAL13P1.70	hypothetical protein conserved	0.162
chr13	600886	602070	MAL13P1.71	hypothetical protein conserved	0.085
chr13	603185	606802	MAL13P1.72	hypothetical protein conserved	0.114
chr13	607996	610254	MAL13P1.73	hypothetical protein conserved	0.058
chr13	611534	619090	PF13_0080	hypothetical protein conserved	0.151
chr13	619333	620727	MAL13P1.74	hypothetical protein conserved	-0.450
chr13	621589	622078	PF13_0081	hypothetical protein conserved	-0.040
chr13	622893	623917	PF13_0082	cop-coated vesicle membrane protein p24 precursor put	0.055
chr13	624987	626213	PF13_0083	lipoate-protein ligase a putative	0.224

chr13	626683	628877	MAL13P1.75	hypothetical protein conserved	0.087
chr13	629968	630646	PF13_0084	ubiquitin-like protein putative	0.202
chr13	632538	634712	MAL13P1.76	TFIIH basal transcription factor subunit	-0.145
chr13	637014	637814	MAL13P1.77	hypothetical protein conserved	0.371
chr13	638488	639591	PF13_0085	serine%2Fthreonine protein kinase putative	-0.310
chr13	639886	643092	MAL13P1.78	hypothetical protein conserved	-0.008
chr13	644916	646594	PF13_0086	hypothetical protein conserved	-0.054
chr13	648188	649553	MAL13P1.79	hypothetical protein conserved	-0.494
chr13	650422	650713	MAL13P1.80	hypothetical protein conserved	-0.644
chr13	652996	654040	MAL13P1.81	hypothetical protein conserved	0.061
chr13	655607	657263	MAL13P1.82	phosphatidylinositol synthase putative	-0.060
chr13	658797	659747	PF13_0087	hypothetical protein conserved	-0.465
chr13	660220	661464	PF13_0088	Myb1 protein	0.035
chr13	663943	668131	MAL13P1.83	karyopherin	0.042
chr13	668562	670947	MAL13P1.84	protein kinase putative	-0.040
chr13	673919	678724	PF13_0089	hypothetical protein conserved	-0.003
chr13	680241	681057	PF13_0090	ADP-ribosylation factor putative	-0.126
chr13	681565	682351	PF13_0090a	conserved Plasmodium protein unknown function	-0.250
chr13	683286	684678	MAL13P1.85	hypothetical protein conserved	0.049
chr13	686153	688873	PF13_0091	hypothetical protein conserved	-0.571
chr13	691216	694327	MAL13P1.86	cholinephosphate cytidylyltransferase	-0.283
chr13	695934	698187	PF13_0093	hypothetical protein conserved	0.014
chr13	698420	700124	MAL13P1.87	sec20 homolog putative	-0.088
chr13	701249	704125	MAL13P1.88	hypothetical protein conserved	-0.043
chr13	708548	710470	PF13_0096	Ubiquitin Carboxyl-terminal Hydrolase-like zinc finger prc	-0.293
chr13	712146	715163	PF13_0095	DNA replication licensing factor mcm4-related	-0.669
chr13	722162	730180	PF13_0097	hypothetical protein conserved	-0.200
chr13	731060	732494	PF13_0098	hypothetical protein conserved	0.217
chr13	733446	734063	PF13_0099	hypothetical protein conserved	0.026
chr13	734805	735074	MAL13P1.89	hypothetical protein conserved	-0.279
chr13	735739	738269	MAL13P1.90	hypothetical protein conserved	0.072
chr13	738681	739154	MAL13P1.91	hypothetical protein conserved	-0.072
chr13	740905	741655	MAL13P1.92	40S ribosomal protein S15 putative	-0.512
chr13	742127	745051	MAL13P1.93	hypothetical protein conserved	0.140
chr13	746148	746726	MAL13P1.94	hypothetical protein conserved	-0.620

chr13	748588	749172	MAL13P1.95	ferredoxin	-0.113
chr13	750517	751719	PF13_0100	glycerol-3-phosphate acyltransferase putative	-0.103
chr13	754111	759633	PF13_0101	hypothetical protein conserved	-0.286
chr13	760527	764340	MAL13P1.96	chromosome segregation protein putative	-0.155
chr13	766636	767971	MAL13P1.97	hypothetical protein	0.379
chr13	768138	769851	MAL13P1.100	hypothetical protein conserved	0.144
chr13	770847	773096	MAL13P1.102	hypothetical protein conserved	-0.014
chr13	774991	776946	PF13_0102	DNAJ-like Sec63 homologue	-0.583
chr13	780135	781880	PF13_0103	hypothetical protein conserved	-0.285
chr13	784311	787193	PF13_0104	hypothetical protein conserved	-0.483
chr13	789638	792049	PF13_0106	hypothetical protein conserved	0.140
chr13	792582	795188	PF13_0107	hypothetical protein conserved	-0.182
chr13	796140	798554	PF13_0109	N2 N2-dimethylguanosine tRNA methyltransferase putat	-0.061
chr13	801913	806230	MAL13P1.103	hypothetical protein conserved	-0.221
chr13	807420	807644	PF13_0112	hypothetical protein conserved	0.066
chr13	810388	815289	PF13_0114	hypothetical protein conserved	0.052
chr13	816023	818599	MAL13P1.105	hypothetical protein conserved	0.040
chr13	820942	821379	MAL13P1.106	hypothetical protein	-0.656
chr13	822279	826739	MAL13P1.107	hypothetical protein conserved	-0.006
chr13	829735	833511	PF13_0116	hypothetical protein conserved	-0.037
chr13	834381	835509	MAL13P1.111	hypothetical protein conserved	0.013
chr13	836764	838647	PF13_0117	hypothetical protein conserved	-0.318
chr13	839049	841250	MAL13P1.112	hypothetical protein conserved	0.359
chr13	841872	842951	PF13_0118	microsomal signal peptidase putative	0.086
chr13	843840	845312	MAL13P1.113	synaptosomal-associated protein of 25 kDa %28SNAP 2	0.002
chr13	847291	848584	PF13_0119	Rab11a GTPase	-0.393
chr13	849863	854191	PF13_0120	dihydrolipoamide suucinyl transferase putative	-0.062
chr13	855133	856664	PF13_0121	dihydrolipoamide succinyltransferase putative	-0.350
chr13	857482	858145	PF13_0122	cyclophilin putative	-0.431
chr13	859500	861548	PF13_0123	hypothetical protein conserved	0.055
chr13	861643	872665	MAL13P1.114	hypothetical protein conserved	-0.016
chr13	875466	876048	MAL13P1.115	hypothetical protein conserved	-0.050
chr13	876422	886465	MAL13P1.116	hypothetical protein conserved	0.249
chr13	887472	889900	MAL13P1.117	hypothetical protein conserved	0.073
chr13	890307	894983	MAL13P1.118	cAMP-specific 3%27 5%27-cyclic phosphodiesterase 4D	0.012

chr13	897917	901245	MAL13P1.119	cAMP-specific 3%27 5%27-cyclic phosphodiesterase 4B	-0.098
chr13	902584	905367	MAL13P1.120	splicing factor putative	-0.279
chr13	908704	909840	PF13_0124	hypothetical protein conserved	-0.204
chr13	911868	912746	PF13_0125	hypothetical protein conserved	-0.150
chr13	916561	918795	MAL13P1.121	hypothetical protein conserved	-0.295
chr13	921172	929650	MAL13P1.122	hypothetical protein conserved	-0.135
chr13	931515	938005	MAL13P1.123	hypothetical protein conserved	-0.017
chr13	938868	943771	MAL13P1.124	hypothetical protein conserved	-0.149
chr13	944476	949486	PF13_0126	hypothetical protein conserved	0.069
chr13	951356	952680	MAL13P1.125	hypothetical protein conserved	-0.082
chr13	953355	955990	MAL13P1.126	hypothetical protein conserved	-0.436
chr13	956158	957696	PF13_0127	hypothetical protein conserved	0.069
chr13	958247	959376	MAL13P1.127	hypothetical protein conserved	-0.167
chr13	960367	960847	MAL13P1.128	hypothetical protein conserved	0.225
chr13	962219	963589	MAL13P1.129	hypothetical protein conserved	0.030
chr13	963862	964891	PF13_0128	beta-hydroxyacyl-acp dehydratase precursor	-0.547
chr13	965687	966481	PF13_0129	ribosomal protein L6 homologue putative	-0.499
chr13	968311	968999	PF13_0130	vacuolar ATP synthase subunit g putative	0.095
chr13	970479	971537	PF13_0131	hypothetical protein conserved	-0.376
chr13	972048	973237	PF13_0132	60S ribosomal protein L23a putative	-0.089
chr13	975604	977376	PF13_0133	aspartyl %28acid%29 protease putative	-0.194
chr13	979166	980422	PF13_0134	hypothetical protein conserved	-0.332
chr13	982812	984002	MAL13P1.130	hypothetical protein conserved	-0.664
chr13	984797	988858	PF13_0135	hypothetical protein conserved	0.144
chr13	989669	990799	PF13_0136	hypothetical protein conserved	-0.146
chr13	991100	995479	PF13_0137	hypothetical protein conserved	-0.088
chr13	997922	999463	MAL13P1.131	hypothetical protein conserved	0.006
chr13	1000301	1002083	MAL13P1.132	microfibril-associated protein homologue putative	0.220
chr13	1002949	1019504	MAL13P1.133	hypothetical protein conserved	0.233
chr13	1021710	1022738	PF13_0138	hypothetical protein conserved	-0.029
chr13	1023461	1027944	MAL13P1.134	helicase putative	-0.004
chr13	1028693	1033830	PF13_0139	hypothetical protein conserved	-0.027
chr13	1035101	1035730	MAL13P1.135	snare protein homologue putative	-0.857
chr13	1036744	1038732	PF13_0140	dihydrofolate synthase%2Ffolylpolyglutamate synthase	-0.518
chr13	1041358	1042308	PF13_0141	L-lactate dehydrogenase	-0.941

chr13	1043056	1043289	PF13_0142	u6 snRNA-associated sm-like protein putative	-0.382	
chr13	1044753	1046066	PF13_0143	phosphoribosylpyrophosphate synthetase	-0.764	
chr13	1048049	1049560	PF13_0144	oxidoreductase putative	-0.788	
chr13	1052952	1063511	MAL13P1.137	hypothetical malaria antigen	-0.057	
chr13	1064145	1066612	MAL13P1.138	hypothetical protein conserved	-0.073	
chr13	1067298	1067886	MAL13P1.139	hypothetical protein conserved	0.081	
chr13	1071347	1075948	PF13_0146	hypothetical protein conserved	0.065	
chr13	1076362	1084788	MAL13P1.140	hypothetical protein conserved	0.175	
chr13	1085987	1087062	MAL13P1.141	hypothetical protein conserved	-0.054	
chr13	1087785	1089300	MAL13P1.142	hypothetical protein conserved	-0.280	
chr13	1090722	1091528	MAL13P1.550	hypothetical protein conserved	0.066	
chr13	1092595	1094388	PF13_0147	hypothetical protein conserved	-0.229	
chr13	1095754	1098665	MAL13P1.144	hypothetical protein conserved	-0.188	
chr13	1099690	1106037	MAL13P1.145	hypothetical protein conserved	0.105	
chr13	1108076	1122741	PF13_0148	hypothetical protein conserved	0.044	
chr13	1134783	1138241	MAL13P1.155	hypothetical protein conserved	0.230	
chr13	1139155	1141762	MAL13P1.154	hypothetical protein conserved	0.221	
chr13	1143358	1144116	MAL13P1.153	hypothetical protein conserved	0.108	
chr13	1145920	1151307	PF13_0161	hypothetical protein conserved	-0.104	
chr13	1152804	1153712	PF13_0160	hypothetical protein conserved	0.228	
chr13	1155223	1158106	MAL13P1.152	hypothetical protein conserved	0.255	
chr13	1159567	1160181	PF13_0159	nucleotidyltransferase putative	-0.179	
chr13	1160662	1161477	PF13_0158	up-frameshift suppressor3 upf3 family putative	0.152	
chr13	1162821	1164503	PF13_0157	ribose-phosphate pyrophosphokinase putative	0.391	13cenL
chr13	1174392	1181033	MAL13P1.151	hypothetical protein conserved	0.400	13cenR
chr13	1184409	1185221	PF13_0156	proteasome subunit beta type 7 precursor putative	-0.476	
chr13	1186820	1195402	PF13_0155	hypothetical protein conserved	0.064	
chr13	1196430	1197710	PF13_0154	hypothetical protein conserved	-0.234	
chr13	1201041	1202543	MAL13P1.150	hypothetical protein conerved	-0.058	
chr13	1204387	1207401	PF13_0153	hypothetical protein conserved	-0.245	
chr13	1209945	1211973	MAL13P1.149	hypothetical protein conserved	0.283	
chr13	1215108	1215929	PF13_0152	transcriptional regulatory protein sir2 homologue putativ	-0.540	
chr13	1216126	1217319	PF13_0151	hypothetical protein conserved	0.347	
chr13	1218118	1225736	PF13_0150	DNA-directed RNA polymerase 3 largest subunit	-0.170	
chr13	1227573	1234619	MAL13P1.148	P. falciparum myosin	-0.545	

chr13	1237401	1238630	MAL13P1.147	hypothetical protein conserved	-0.160
chr13	1239687	1241435	PF13_0149	chromatin assembly factor 1 subunit putative	-0.697
chr13	1243373	1246345	MAL13P1.146	AMP deaminase putative	-0.619
chr13	1252257	1258171	PF13_0162	hypothetical protein conserved	-0.445
chr13	1259154	1260059	PF13_0163	hypothetical protein conserved	0.163
chr13	1261543	1263141	MAL13P1.156	hypothetical protein conserved	-0.179
chr13	1265335	1268961	MAL13P1.157	hypothetical protein conserved	0.000
chr13	1269509	1271632	MAL13P1.158	hypothetical protein conserved	0.172
chr13	1272411	1274057	MAL13P1.159	hypothetical protein conserved	-0.170
chr13	1274646	1275206	MAL13P1.160	hypothetical protein conserved	-0.206
chr13	1275302	1276097	MAL13P1.161	hypothetical protein conserved	-0.164
chr13	1276961	1278571	MAL13P1.162	DNAJ-like protein putative	-0.106
chr13	1279998	1281483	MAL13P1.163	er lumen protein retaining receptor 1 putative	-0.292
chr13	1285186	1287363	PF13_0164	hypothetical protein conserved	-0.368
chr13	1288089	1289606	MAL13P1.164	elongation factor tu putative	-0.478
chr13	1290316	1293152	MAL13P1.165	hypothetical protein conserved	0.028
chr13	1294813	1297164	PF13_0165	hypothetical protein conserved	-0.086
chr13	1297798	1299471	PF13_0166	protein kinase putative	-0.228
chr13	1300896	1305492	MAL13P1.166	hypothetical protein conserved	-0.100
chr13	1306852	1308197	PF13_0167	hypothetical protein conserved	-0.088
chr13	1309375	1310204	MAL13P1.167	signal peptidase putative	-0.304
chr13	1310969	1312414	PF13_0168	hypothetical protein conserved	-0.538
chr13	1314122	1315984	PF13_0169	hypothetical protein conserved	-0.106
chr13	1316694	1318853	MAL13P1.168	hypothetical protein conserved	-0.345
chr13	1319287	1322043	PF13_0170	glutaminyl-tRNA synthetase putative	-0.595
chr13	1324210	1325000	PF13_0171	60S ribosomal protein L23 putative	-0.515
chr13	1326111	1327730	MAL13P1.169	syntaxin 5 putative	-0.126
chr13	1329689	1331257	PF13_0172	hypothetical protein conserved	-0.052
chr13	1332497	1335790	PF13_0173	hypothetical protein conserved	-0.794
chr13	1336599	1337090	PF13_0174	P. falciparum homologue of human mbp-1 interacting prc	-0.560
chr13	1337315	1339891	MAL13P1.170	hypothetical protein conserved	-0.128
chr13	1340984	1341640	PF13_0175	hypothetical protein conserved	-0.549
chr13	1342210	1344003	PF13_0176	apurinic%2Fapyrimidinic endonuclease Apn1	-0.187
chr13	1345004	1346476	PF13_0177	ATP-dependent RNA helicase putative	-0.419
chr13	1348977	1349720	PF13_0178	translation initiation factor 6 putative	-0.556

chr13	1350240	1354058	PF13_0179	isoleucine--tRNA ligase putative	-0.405
chr13	1355483	1357392	PF13_0180	chaperonin putative	-0.113
chr13	1357902	1360517	PF13_0181	hypothetical protein conserved	0.249
chr13	1362291	1367807	PF13_0182	hypothetical protein conserved	-0.034
chr13	1368279	1368914	MAL13P1.171	transmembrane protein Tmp21 homologue putative	-0.424
chr13	1369943	1370997	MAL13P1.172	hypothetical protein conserved	-0.377
chr13	1371869	1373254	PF13_0183	hypothetical protein conserved	-0.037
chr13	1373588	1377595	PF13_0184	hypothetical protein conserved	0.112
chr13	1379307	1379819	PF13_0185	histone h3 putative	-0.899
chr13	1382829	1383533	PF13_0186	hypothetical protein conserved	-0.109
chr13	1385256	1389341	PF13_0187	hypothetical protein conserved	-0.048
chr13	1390214	1391551	PF13_0188	hypothetical protein conserved	0.119
chr13	1392812	1395763	PF13_0189	hypothetical protein conserved	0.067
chr13	1398469	1400043	PF13_0190	hypothetical protein conserved	0.159
chr13	1401095	1402474	PF13_0191	hypothetical protein conserved	0.294
chr13	1403720	1404649	MAL13P1.173	MSP7-like protein	0.014
chr13	1405929	1408127	PF13_0192	hypothetical protein conserved	0.031
chr13	1408956	1410143	PF13_0193	MSP7-like protein	-0.137
chr13	1411298	1411906	PF13_0194	hypothetical protein	-0.252
chr13	1413300	1414145	MAL13P1.174	MSP7-like protein	0.003
chr13	1415076	1415216	MAL13P1.175	msp7-like protein fragment	0.179
chr13	1416514	1417656	PF13_0196	MSP7-like protein	-0.106
chr13	1419284	1420339	PF13_0197	Merozoite Surface Protein 7 precursor MSP7	0.105
chr13	1429072	1438611	MAL13P1.176	Plasmodium falciparum reticulocyte binding protein 2 ho	-0.450
chr13	1440977	1450583	PF13_0198	reticulocyte binding protein 2 homolog a	-0.154
chr13	1451687	1453641	MAL13P1.177	hypothetical protein conserved	-0.006
chr13	1454705	1456073	MAL13P1.178	hypothetical protein conserved	0.083
chr13	1456308	1461524	PF13_0199	hypothetical protein conserved	0.126
chr13	1462378	1464189	PF13_0200	hypothetical protein conserved	0.199
chr13	1465093	1466817	PF13_0201	Thrombospondin-related anonymous protein TRAP	-0.791
chr13	1470042	1471651	MAL13P1.179	hypothetical protein conserved	-0.064
chr13	1472381	1472879	PF13_0202	hypothetical protein conserved	-0.333
chr13	1474471	1475858	MAL13P1.180	hypothetical protein conserved	-0.018
chr13	1476530	1477504	MAL13P1.181	hypothetical protein conserved	-0.001
chr13	1478158	1480221	MAL13P1.182	hypothetical protein conserved	0.084

chr13	1481151	1481899	MAL13P1.183	hypothetical protein conserved	-0.226
chr13	1482558	1483034	PF13_0203	hypothetical protein	0.444
chr13	1486652	1487812	PF13_0204	nuclear movement protein putative	-0.350
chr13	1488257	1490458	PF13_0205	tryptophan--tRNA ligase putative	-0.131
chr13	1491124	1494504	MAL13P1.184	endopeptidase putative	-0.047
chr13	1495455	1497436	MAL13P1.185	Pf protein kinase 6	-0.161
chr13	1498073	1501690	MAL13P1.186	1-deoxy-D-xylulose 5-phosphate synthase	-0.219
chr13	1503211	1506218	PF13_0208	exonuclease putative	-0.019
chr13	1507161	1509173	PF13_0209	hypothetical protein conserved	0.128
chr13	1509788	1519789	PF13_0210	hypothetical protein conserved	-0.140
chr13	1525757	1528009	MAL13P1.188	hypothetical protein conserved	-0.207
chr13	1528697	1530403	PF13_0211	calcium-dependent protein kinase	-0.265
chr13	1534702	1535361	PF13_0212	hypothetical protein conserved	-0.208
chr13	1536658	1540548	MAL13P1.189	conserved hypothetical protein	-0.127
chr13	1542275	1544176	MAL13P1.190	proteasome regulatory component putative	-0.541
chr13	1545265	1545930	PF13_0213	60S ribosomal subunit protein L6e putative	-0.023
chr13	1549043	1550591	PF13_0214	elongation factor 1-gamma putative	-0.391
chr13	1551144	1553298	MAL13P1.191	hypothetical protein conserved	-0.005
chr13	1554394	1556559	MAL13P1.192	hypothetical protein conserved	0.165
chr13	1556854	1559929	MAL13P1.193	hypothetical protein conserved	-0.317
chr13	1561450	1563318	MAL13P1.194	hypothetical protein conserved	-0.163
chr13	1565127	1568275	MAL13P1.195	hypothetical protein conserved	-0.194
chr13	1568569	1571364	MAL13P1.196	protein kinase putative	-0.043
chr13	1572826	1573918	MAL13P1.197	hypothetical protein	-0.214
chr13	1583421	1584248	PF13_0215	hypothetical protein conserved	-0.571
chr13	1586001	1586594	PF13_0216	hypothetical protein conserved	-0.180
chr13	1587223	1588069	MAL13P1.200	hypothetical protein conserved	0.076
chr13	1588599	1590485	MAL13P1.201	hypothetical protein conserved	0.129
chr13	1590705	1596515	MAL13P1.202	hypothetical protein conserved	-0.140
chr13	1597595	1597813	PF13_0217	hypothetical protein conserved	0.540
chr13	1598596	1601373	PF13_0218	ABC transporter %28MDR family%29	-0.172
chr13	1601893	1603749	MAL13P1.203	hypothetical protein conserved	-0.403
chr13	1604571	1605407	MAL13P1.204	exoribonuclease PH putative	-0.552
chr13	1607518	1610385	PF13_0219	hypothetical protein conserved	0.143
chr13	1610910	1612184	PF13_0220	hypothetical protein conserved	-0.076

chr13	1613392	1617000	PF13_0221	hypothetical protein conserved	0.169
chr13	1618196	1619923	PF13_0222	RNA lariat debranching enzyme putative	-0.762
chr13	1621344	1623525	MAL13P1.205	Rab11b GTPase	0.121
chr13	1624085	1624521	PF13_0223	hypothetical protein conserved	-0.637
chr13	1627342	1629511	MAL13P1.206	Na%2B -dependent Pi transporter sodium-dependent ph	-0.628
chr13	1633891	1634561	PF13_0224	60S ribosomal subunit protein L18 putative	-0.625
chr13	1636877	1637864	MAL13P1.209	60S ribosomal subunit porotein L18 putative	-0.494
chr13	1639648	1641174	PF13_0225	hypothetical protein conserved	-0.316
chr13	1642150	1644510	MAL13P1.210	dolichyl-phosphate-mannose-glygolipidalpha-mannosyltr	-0.010
chr13	1645097	1645697	MAL13P1.211	hypothetical protein conserved	0.177
chr13	1646361	1647749	PF13_0226	hypothetical protein conserved	-0.177
chr13	1649144	1650680	PF13_0227	vacuolar ATP synthase subunit D putative	-0.514
chr13	1653242	1654162	PF13_0228	40S ribosomal subunit protein S6 putative	0.029
chr13	1657473	1660202	PF13_0229	IRP-like protein	-0.868
chr13	1660843	1663761	PF13_0230	hypothetical protein conserved	0.239
chr13	1664885	1665676	PF13_0231	hypothetical protein conserved	-0.018
chr13	1666863	1668184	PF13_0232	Casein kinase II regulatory subunit putative	-0.128
chr13	1669725	1670987	MAL13P1.212	hypothetical protein conserved	-0.059
chr13	1673730	1676570	PF13_0233	myosin a	-0.680
chr13	1677571	1677948	MAL13P1.213	transcription activator putative	-0.002
chr13	1679724	1681475	PF13_0234	phosphoenolpyruvate carboxykinase	-0.853
chr13	1682424	1694000	PF13_0235	hypothetical protein conserved	0.072
chr13	1696925	1698144	MAL13P1.214	phosphoethanolamine N-methyltransferase putative	-0.044
chr13	1699714	1700661	MAL13P1.215	hypothetical protein conserved	0.149
chr13	1704601	1706154	PF13_0236	hypothetical protein conserved	-0.305
chr13	1707762	1710710	PF13_0237	hypothetical protein conserved	-0.132
chr13	1714474	1719286	MAL13P1.216	DNA helicase putative	-0.208
chr13	1720350	1721703	MAL13P1.217	hypothetical protein conserved	-0.117
chr13	1721935	1723844	MAL13P1.218	UDP-N-acetylglucosamine pyrophosphorylase putative	-0.100
chr13	1724848	1727028	PF13_0238	conserved protein putative	-0.504
chr13	1784158	1785207	MAL13P1.219	hypothetical protein conserved	0.131
chr13	1785763	1787010	MAL13P1.220	lipoate synthase putative	-0.662
chr13	1787370	1791104	PF13_0239	hypothetical protein conserved	0.042
chr13	1792612	1794047	MAL13P1.221	aspartate carbamoyltransferase	-0.404
chr13	1797609	1802075	MAL13P1.222	hypothetical protein conserved	0.044

chr13	1803497	1804151	MAL13P1.223	hypothetical protein	-0.200
chr13	1804358	1805955	MAL13P1.224	hypothetical protein conserved	0.044
chr13	1806074	1806859	MAL13P1.225	thioredoxin putative	-0.038
chr13	1808558	1810267	PF13_0241	peptidase putative	-0.129
chr13	1812682	1817638	MAL13P1.226	hypothetical protein conserved	-0.169
chr13	1819528	1820364	MAL13P1.227	ubiquitin-conjugating enzyme putative	0.067
chr13	1822749	1823987	MAL13P1.228	hypothetical protein conserved	-0.450
chr13	1824621	1826027	PF13_0242	isocitrate dehydrogenase %28NADP%29 mitochondrial	-0.498
chr13	1828180	1831329	PF13_0243	hypothetical protein conserved	0.118
chr13	1831849	1833137	MAL13P1.229	hypothetical protein conserved	-0.013
chr13	1834090	1836443	MAL13P1.230	hypothetical protein conserved	0.207
chr13	1839850	1841232	MAL13P1.231	Pfsec61	-0.883
chr13	1842216	1842722	MAL13P1.232	mog1 homolog putative	0.261
chr13	1845859	1846848	MAL13P1.233	hypothetical protein conserved	0.392
chr13	1849229	1867590	MAL13P1.234	hypothetical protein conserved	-0.355
chr13	1868253	1870040	PF13_0245	hypothetical protein conserved	-0.223
chr13	1870391	1871056	PF13_0246	hypothetical protein conserved	-0.182
chr13	1872347	1873693	PF13_0247	transmission blocking target antigen precursor	-0.128
chr13	1875206	1876525	PF13_0248	pf47	-0.177
chr13	1878233	1878988	PF13_0249	hypothetical protein conserved	-0.244
chr13	1879295	1881277	PF13_0250	G-beta repeat protein putative	-0.271
chr13	1882675	1884807	PF13_0251	DNA topoisomerase III putative	-0.303
chr13	1886184	1887452	PF13_0252	nucleoside transporter 1	-0.354
chr13	1890126	1891354	MAL13P1.235	hypothetical protein conserved	0.114
chr13	1892314	1892733	MAL13P1.236	hypothetical protein conserved	-0.084
chr13	1894314	1895693	MAL13P1.237	hypothetical protein conserved	-0.373
chr13	1905291	1907216	PF13_0253	ethanolamine-phosphate cytidylyltransferase putative	-0.609
chr13	1907838	1909163	MAL13P1.238	hypothetical protein conserved	0.223
chr13	1909279	1915235	MAL13P1.239	hypothetical protein conserved	0.305
chr13	1916165	1920330	MAL13P1.240	hypothetical protein conserved	0.116
chr13	1921380	1923111	MAL13P1.241	GTPase putative	0.182
chr13	1923780	1924591	MAL13P1.242	hypothetical protein conserved	0.091
chr13	1925422	1930052	MAL13P1.243	elongation factor Tu putative	-0.077
chr13	1931927	1943711	PF13_0254	hypothetical protein conserved	0.142
chr13	1946731	1948178	MAL13P1.244	TBC domain protein putative	-0.131

chr13	1949635	1951183	PF13_0255	hypothetical protein conserved	-0.018
chr13	1951773	1954689	MAL13P1.245	hypothetical protein conserved	0.057
chr13	1955988	1961899	MAL13P1.246	cation-transporting ATPase	0.326
chr13	1962294	1962653	PF13_0256	hypothetical protein conserved	-0.212
chr13	1963221	1965758	MAL13P1.247	hypothetical protein conserved	-0.030
chr13	1966100	1967083	MAL13P1.248	nucleoside diphospahte hydrolase	0.019
chr13	1968221	1970812	PF13_0257	glutamate--tRNA ligase	-0.335
chr13	1973600	1979023	PF13_0258	sexual stage-specific protein kinase	-0.243
chr13	1980154	1981342	PF13_0259	cytidine and deoxycytidylate deaminase family putative	0.220
chr13	1981668	1991945	MAL13P1.249	hypothetical protein conserved	0.086
chr13	1993354	1994613	MAL13P1.250	hypothetical protein conserved	-0.338
chr13	1995146	1996102	PF13_0260	hypothetical protein conserved	-0.251
chr13	1997012	1998331	PF13_0261	ATP binding protein putative	-0.024
chr13	1998760	1999320	MAL13P1.251	hypothetical protein conserved	-0.008
chr13	1999663	2001465	MAL13P1.252	hypothetical protein conserved	0.369
chr13	2002293	2004281	PF13_0262	lysine--tRNA ligase	-0.699
chr13	2005808	2006612	MAL13P1.253	small nuclear ribonucleoprotein putative	0.023
chr13	2007499	2008416	PF13_0263	hypothetical protein conserved	-0.108
chr13	2009130	2011392	PF13_0264	hypothetical protein conserved	0.025
chr13	2011698	2015132	PF13_0265	hypothetical protein conserved	-0.119
chr13	2017681	2018638	MAL13P1.254	hypothetical protein conserved	-0.115
chr13	2019590	2021272	MAL13P1.255	hypothetical protein conserved	-0.423
chr13	2021697	2022437	PF13_0266	hypothetical protein conserved	0.382
chr13	2023404	2024969	PF13_0267	hypothetical protein conserved	-0.239
chr13	2030169	2036091	MAL13P1.256	phosphatidylinositol transfer protein putative	-0.075
chr13	2036736	2037886	MAL13P1.257	hypothetical protein conserved	-0.048
chr13	2039437	2044089	MAL13P1.258	hypothetical protein conserved	0.178
chr13	2044712	2045987	MAL13P1.259	hypothetical protein conserved	0.002
chr13	2048439	2049715	PF13_0268	ribosomal protein L17 putative	-0.305
chr13	2051547	2053052	PF13_0269	glycerol kinase putative	-0.355
chr13	2055947	2060255	MAL13P1.260	hypothetical protein conserved	-0.195
chr13	2062084	2062740	PF13_0270	hypothetical protein conserved	-0.132
chr13	2063645	2065098	MAL13P1.261	hypothetical protein conserved	-0.179
chr13	2065648	2071668	MAL13P1.262	hypothetical protein conserved	0.078
chr13	2072409	2075558	PF13_0271	ABC transporter %28heavy metal transporter family%29	0.167

chr13	2077095	2079483	MAL13P1.264	hypothetical protein conserved	0.045
chr13	2080020	2081861	MAL13P1.265	hypothetical protein conserved	0.213
chr13	2083724	2088940	MAL13P1.266	hypothetical protein conserved	0.089
chr13	2089696	2090668	PF13_0272	thioredoxin-related protein putative	0.148
chr13	2093010	2095915	MAL13P1.267	hypothetical protein conserved	0.381
chr13	2097818	2105419	PF13_0273	hypothetical protein conserved	-0.033
chr13	2106685	2109689	PF13_0274	hypothetical protein conserved	0.008
chr13	2110490	2112544	MAL13P1.268	hypothetical protein	-0.180
chr13	2114694	2117246	MAL13P1.269	hypothetical protein	-0.184
chr13	2120605	2121603	PF13_0275	hypothetical protein	-0.305
chr13	2126864	2127398	PF13_0276	membrane-associated histidine rich protein 2 %28MARH	-0.589
chr13	2128401	2134916	PF13_0277	hypothetical protein conserved	0.204
chr13	2136061	2139555	PF13_0278	Ran-binding protein putative	-0.061
chr13	2140458	2141438	PF13_0279	hypothetical protein conserved	0.221
chr13	2142514	2143480	PF13_0280	ER lumen protein retaining receptor	0.011
chr13	2146401	2146907	PF13_0281	hypothetical protein conserved	-0.722
chr13	2147735	2148706	PF13_0282	proteasome subunit putative	-0.187
chr13	2150495	2151550	MAL13P1.270	proteasome subunit putative	-0.387
chr13	2152088	2153875	PF13_0283	hypothetical protein conserved	0.269
chr13	2154462	2155532	PF13_0284	hypothetical protein conserved	0.162
chr13	2156651	2159062	PF13_0285	inositol-polyphosphate 5-phosphatase	-0.320
chr13	2160572	2163749	PF13_0286	methyltransferase putative	-0.006
chr13	2165647	2166749	MAL13P1.271	V-type ATPase putative	0.018
chr13	2170679	2172130	PF13_0287	adenylosuccinate synthetase	-0.578
chr13	2172641	2173605	MAL13P1.272	hypothetical protein conserved	0.579
chr13	2174972	2176195	PF13_0288	hypothetical protein conserved	0.210
chr13	2177291	2179132	PF13_0289	protease caspase family	-0.461
chr13	2180370	2182283	PF13_0290	hypothetical protein conserved	-0.013
chr13	2183155	2183451	MAL13P1.273	hypothetical protein	0.434
chr13	2183768	2186557	PF13_0291	replication licensing factor putative	-0.754
chr13	2187391	2190444	PF13_0292	hypothetical protein	0.420
chr13	2191782	2193311	PF13_0293	hypothetical protein conserved	0.135
chr13	2194239	2194311	MAL13_tRNA_T	tRNA threonine	1.875
chr13	2195927	2197903	MAL13P1.274	serine%2Fthreonine protein phosphatase pfPp5	-0.105
chr13	2198944	2200814	PF13_0295	hypothetical protein conserved	-0.118

chr13	2202925	2206791	MAL13P1.275	NLI interacting factor-like phosphatase putative	0.007
chr13	2210778	2211426	PF13_0296	hypothetical protein conserved	-0.208
chr13	2212679	2213896	PF13_0297	hypothetical protein conserved	-0.549
chr13	2215076	2219272	PF13_0298	hypothetical protein conserved	0.028
chr13	2220479	2223373	PF13_0299	hypothetical protein conserved	0.162
chr13	2224853	2225624	PF13_0300	mitochondrial inner membrane translocase putative	-0.299
chr13	2228216	2229581	PF13_0301	ubiquitin-conjugating enzyme putative	-0.102
chr13	2231422	2234442	PF13_0302	hypothetical protein conserved	-0.072
chr13	2235594	2236337	MAL13P1.276	hypothetical protein conserved	-0.244
chr13	2237303	2238745	PF13_0303	regulator of chromosome condensation putative	-0.894
chr13	2241522	2242845	MAL13P1.277	DnaJ-like protein putative	0.064
chr13	2245920	2258054	MAL13P1.278	Ser%2FThr protein kinase	-0.169
chr13	2259532	2261088	MAL13P1.279	P. falciparum Protein Kinase 5	-0.255
chr13	2262150	2263481	PF13_0304	elongation factor 1 alpha	-0.462
chr13	2265234	2266565	PF13_0305	elongation factor 1 alpha	-0.630
chr13	2267870	2270416	MAL13P1.281	glutamate--tRNA ligase putative	-0.091
chr13	2271046	2271411	PF13_0306	dynein light chain	-0.296
chr13	2272854	2276393	PF13_0307	hypothetical protein conserved	-0.006
chr13	2277825	2280545	PF13_0308	DNA helicase	-0.282
chr13	2281146	2282036	MAL13P1.282	hypothetical protein conserved	-0.025
chr13	2282553	2286543	PF13_0309	hypothetical protein conserved	-0.027
chr13	2288507	2290467	MAL13P1.283	TCP-1%2Fcpn60 chaperonin family putative	-0.354
chr13	2291970	2293489	MAL13P1.284	pyrroline carboxylate reductase	-0.341
chr13	2294233	2300527	MAL13P1.285	Patatin-like phospholipase putative	0.073
chr13	2301769	2303973	PF13_0310	hypothetical protein conserved	0.101
chr13	2305368	2310558	MAL13P1.286	hypothetical protein conserved	0.174
chr13	2310790	2311812	PF13_0312	peptidase putative	0.256
chr13	2313335	2314177	MAL13P1.287	hypothetical protein conserved	0.288
chr13	2315019	2316860	PF13_0313	zinc finger protein	-0.171
chr13	2318393	2321410	PF13_0314	hypothetical protein conserved	-0.251
chr13	2322727	2323188	MAL13P1.288	hypothetical protein conserved	-0.212
chr13	2327407	2327862	PF13_0316	40S ribosomal protein S13	-0.249
chr13	2328529	2331248	MAL13P1.294	GTP-binding protein putative	0.060
chr13	2332338	2342183	MAL13P1.293	hypothetical protein conserved	0.214
chr13	2344196	2346319	MAL13P1.292	riboflavin kinase %2F FAD synthase family protein putat	0.159

chr13	2346770	2347544	MAL13P1.291	hypothetical protein conserved	0.058
chr13	2347583	2349153	MAL13P1.290	high mobility group protein 4 putative	-0.064
chr13	2350014	2353235	MAL13P1.289	mitotic control protein dis3 homologue putative	-0.004
chr13	2355584	2357531	PF13_0315	RNA binding protein putative	-0.445
chr13	2368215	2374289	MAL13P1.295	hypothetical protein conserved	-0.071
chr13	2377498	2388594	MAL13P1.296	hypothetical protein conserved	0.113
chr13	2389673	2390688	MAL13P1.297	ADP-ribosylation factor putative	0.108
chr13	2392307	2398687	MAL13P1.298	hypothetical protein conserved	-0.100
chr13	2399844	2400377	PF13_0317	hypothetical protein conserved	-0.493
chr13	2401582	2402550	PF13_0318	RNA-binding protein putative	-0.190
chr13	2403171	2404718	MAL13P1.299	hypothetical protein conserved	-0.256
chr13	2405681	2406799	MAL13P1.300	hypothetical protein conserved	0.387
chr13	2407690	2408631	PF13_0319	hypothetical protein conserved	0.164
chr13	2409657	2420822	MAL13P1.301	protein with aminophospholipid-transporting P-ATPase a	0.537
chr13	2422466	2424109	PF13_0321	hypothetical protein	0.097
chr13	2425217	2427516	MAL13P1.302	SUMO ligase putative	-0.224
chr13	2431676	2435257	PF13_0322	falcilysin	-0.339
chr13	2436771	2439030	MAL13P1.303	polyadenylate binding protein putative	-0.038
chr13	2441423	2443597	PF13_0323	binding protein putative	-0.208
chr13	2446794	2450093	PF13_0324	Sec24 subunit putative	-0.599
chr13	2451594	2457757	MAL13P1.304	malaria antigen	-0.255
chr13	2458402	2459994	MAL13P1.305	hypothetical protein conserved	0.055
chr13	2460468	2461051	PF13_0326	actin-depolymerizing factor putative	-0.105
chr13	2461824	2464315	MAL13P1.306	hypothetical protein conserved	-0.065
chr13	2465460	2465873	MAL13P1.307	hypothetical protein conserved	0.138
chr13	2467305	2468126	PF13_0327	cytochrome c oxidase subunit 2 putative	-0.023
chr13	2469863	2477959	MAL13P1.308	hypothetical protein conserved	-0.696
chr13	2478805	2479629	PF13_0328	proliferating cell nuclear antigen	-0.383
chr13	2483087	2483686	PF13_0329	hypothetical protein	-1.021
chr13	2484190	2485808	MAL13P1.309	14-3-3 protein putative	0.009
chr13	2486719	2488170	PF13_0330	ATP-dependent DNA helicase putative	-0.673
chr13	2489476	2490045	PF13_0331	hypothetical protein conserved	-0.161
chr13	2492257	2498403	MAL13P1.310	cysteine protease calpain family	-0.255
chr13	2499518	2501791	MAL13P1.311	exonnuclease putative	0.031
chr13	2502481	2503547	MAL13P1.312	hypothetical protein conserved	-0.445

chr13	2506689	2512808	MAL13P1.313	hypothetical protein conserved	-0.101
chr13	2514821	2516947	MAL13P1.314	hypothetical protein conserved	0.126
chr13	2517673	2521827	MAL13P1.315	hypothetical protein conserved	-0.247
chr13	2522694	2527817	MAL13P1.316	hypothetical protein conserved	0.019
chr13	2528535	2532056	MAL13P1.317	hypothetical protein conserved	0.100
chr13	2532649	2535976	PF13_0333	hypothetical protein conserved	0.247
chr13	2536282	2537874	PF13_0334	polynucleotide kinase putative	0.177
chr13	2538618	2539484	MAL13P1.318	hypothetical protein conserved	0.063
chr13	2539978	2542817	PF13_0335	hypothetical protein conserved	0.179
chr13	2544098	2546811	PF13_0336	hypothetical protein conserved	0.174
chr13	2547577	2548260	PF13_0337	hypothetical protein conserved	-0.229
chr13	2548888	2551260	MAL13P1.319	hypothetical protein conserved	-0.233
chr13	2552359	2555881	MAL13P1.320	hypothetical protein conserved	0.153
chr13	2560960	2563443	MAL13P1.321	hypothetical protein conserved	-0.121
chr13	2564891	2567281	PF13_0338	cysteine-rich surface protein	-0.212
chr13	2568180	2573621	PF13_0339	hypothetical protein conserved	0.038
chr13	2575494	2578949	MAL13P1.322	splicing factor putative	-0.255
chr13	2581367	2591164	MAL13P1.323	hypothetical protein conserved	0.145
chr13	2592724	2594157	PF13_0340	exosome complex exonuclease putative	-0.141
chr13	2595092	2597734	MAL13P1.324	aldo-keto reductase putative	-0.080
chr13	2598136	2599230	MAL13P1.325	hypothetical protein conserved	0.041
chr13	2599798	2601307	PF13_0341	DNA-directed RNA polymerase 2 putative	-0.360
chr13	2602732	2604385	MAL13P1.326	ferrochelatase putative	0.048
chr13	2605033	2606079	PF13_0342	hypothetical protein conserved	-0.003
chr13	2606268	2606916	MAL13P1.327	Ribosomal protein S17 homologue putative	-0.285
chr13	2607818	2607973	MAL13P1.580	hypothetical protein conserved	-0.277
chr13	2608757	2610979	PF13_0343	hypothetical protein conserved	0.049
chr13	2612483	2614237	PF13_0344	UBA%2FTHIF-type NAD%2FFAD binding protein putati	-0.067
chr13	2615363	2616902	PF13_0345	aminomethyltransferase mitochondrial precursor	-0.168
chr13	2617596	2620335	MAL13P1.328	hypothetical protein conserved	0.094
chr13	2620888	2622433	MAL13P1.329	hypothetical protein conserved	-0.381
chr13	2626196	2627777	MAL13P1.330	hypothetical protein conserved	-0.075
chr13	2628869	2629595	PF13_0346	ubiquitin%2Fribosomal fusion protein uba52 homologue	-0.033
chr13	2631548	2631823	PF13_0347	hypothetical protein conserved	-0.568
chr13	2633067	2633575	MAL13P1.331	hypothetical protein conserved	-0.590

chr13	2634293	2634878	MAL13P1.332	hypothetical protein conserved	-0.056
chr13	2635290	2645809	MAL13P1.333	hypothetical protein conserved	0.101
chr13	2646929	2649032	MAL13P1.334	hypothetical protein conserved	-0.175
chr13	2651612	2655400	PF13_0348	PfRhop148 Rhoptry protein	0.043
chr13	2657908	2658357	PF13_0349	nucleoside diphosphate kinase b%3B putative	-0.412
chr13	2659757	2661487	PF13_0350	signal recognition particle receptor alpha subunit putativ	-0.310
chr13	2663001	2664116	PF13_0351	hypothetical protein	0.274
chr13	2664733	2666431	MAL13P1.335	phosphatidylserine synthase i%3B putative	-0.304
chr13	2671924	2674300	MAL13P1.336	hypothetical protein conserved	-0.246
chr13	2675749	2676759	MAL13P1.337	Skp1 family protein putative	-0.360
chr13	2677655	2678926	MAL13P1.338	u1 small nuclear ribonucleoprotein putative	-0.536
chr13	2679915	2681301	MAL13P1.339	hypothetical protein conserved	0.291
chr13	2681393	2682665	MAL13P1.340	hypothetical protein conserved	0.194
chr13	2683895	2687035	PF13_0352	nucleoside transporter	-0.173
chr13	2688410	2689616	PF13_0353	NADH-cytochrome b5 reductase putative	-0.218
chr13	2690255	2691193	MAL13P1.341	60S ribosomal protein	0.207
chr13	2691989	2696215	PF13_0354	alanine--tRNA ligase putative	0.293
chr13	2697562	2701110	PF13_0355	hypothetical protein conserved	3.407
chr13	2702833	2704776	MAL13P1.342	hypothetical protein conserved	1.799
chr13	2706740	2708052	MAL13P1.343	proteasome regulatory subunit putative	-0.309
chr13	2709797	2711770	MAL13P1.344	RNAse L inhibitor protein putative	-0.565
chr13	2714187	2715711	MAL13P1.345	hypothetical protein conserved	0.195
chr13	2715824	2717754	PF13_0356	hypothetical protein conserved	0.241
chr13	2719024	2721516	PF13_0357	hypothetical protein conserved	0.147
chr13	2722003	2722281	PF13_0358	mitochondrial import inner membrane translocase putati	0.046
chr13	2723759	2725489	PF13_0359	mitochondrial carrier protein putative	-0.300
chr13	2727375	2732477	MAL13P1.346	DNA repair endonuclease	-0.019
chr13	2735684	2737332	MAL13P1.347	hypothetical protein conserved	-0.606
chr13	2738919	2742196	MAL13P1.348	hypothetical protein conserved	0.037
chr13	2742993	2744558	PF13_0360	hypothetical protein conserved	0.269
chr13	2745929	2752441	MAL13P1.349	hypothetical protein	0.111
chr13	2752863	2754331	MAL13P1.350	hypothetical protein conserved	0.267
chr13	2756090	2764812	MAL13P1.351	hypothetical protein conserved	0.255
chr13	2766250	2769814	MAL13P1.352	hypothetical protein conserved	0.261
chr13	2770525	2773218	PF13_0361	hypothetical protein conserved	0.403

chr13	2856996	2864550	MAL13P1.356	erythrocyte membrane protein 1 %28PfEMP1%29	4.551
chr14	1394	5344	PF14_0001	erythrocyte membrane protein 1 %28PfEMP1%29 trunca	2.821
chr14	7209	8141	PF14_0002	rifin	3.864
chr14	14127	15364	PF14_0004	rifin	4.881
chr14	17470	18809	PF14_0005	rifin	4.946
chr14	20898	22233	PF14_0006	rifin	5.172
chr14	24338	25321	PF14_0007	stevor putative	5.198
chr14	27322	28429	PF14_0008	rifin	5.195
chr14	30084	31009	PF14_0009	hypothetical protein	4.917
chr14	34694	35774	PF14_0010	glycophorin binding protein-related antigen	4.217
chr14	41290	43151	PF14_0013	hypothetical protein	2.772
chr14	46834	47745	PF14_0014	hypothetical protein	0.108
chr14	48913	50416	PF14_0015	aminopeptidase putative	-0.066
chr14	53412	53735	PF14_0016	early transcribed membrane protein 14.1 etramp14.1	-0.538
chr14	57173	58294	PF14_0017	lysophospholipase putative	0.194
chr14	61365	63224	PF14_0018	hypothetical protein	0.077
chr14	66269	67081	PF14_0019	hypothetical protein	0.280
chr14	71368	72690	PF14_0020	choline kinase putative	-0.357
chr14	74188	77804	PF14_0021	hypothetical protein	0.174
chr14	77977	80423	PF14_0022	exopolyphosphatase putative	-0.100
chr14	81121	83760	PF14_0023	hypothetical protein conserved	-0.077
chr14	84735	85301	PF14_0024	hypothetical protein	-0.319
chr14	85883	87883	PF14_0025	proteosome subunit putative	0.135
chr14	89565	91037	PF14_0026	hypothetical protein	-0.109
chr14	92264	93537	PF14_0027	ribosomal S27a putative	0.099
chr14	94980	98404	PF14_0028	hypothetical protein conserved	0.159
chr14	100847	109234	PF14_0029	hypothetical protein	-0.052
chr14	110114	112475	PF14_0030	hypothetical protein	-0.056
chr14	113144	125340	PF14_0031	hypothetical protein	-0.085
chr14	125986	129877	PF14_0032	hypothetical protein	0.244
chr14	130636	131828	PF14_0810	hypothetical protein conserved	0.066
chr14	132554	133239	PF14_0033	hypothetical protein	0.371
chr14	134081	135073	PF14_0034	hypothetical protein conserved	-0.438
chr14	136530	140885	PF14_0035	hypothetical protein	0.170
chr14	141410	143138	PF14_0036	acid phosphatase putative	-0.216

chr14	143984	144661	PF14_0037	hypothetical protein	0.103
chr14	145134	145649	PF14_0038	cytochrome c putative	-0.537
chr14	147961	148415	PF14_0039	hypothetical protein	-0.209
chr14	150000	150608	PF14_0040	hypothetical protein	-0.443
chr14	151765	152310	PF14_0041	ribosomal protein L16 putative	-0.994
chr14	153139	154545	PF14_0042	U3 small nucleolar ribonucleoprotein U3 snoRNP putati	-0.237
chr14	154855	156918	PF14_0043	hypothetical protein	0.030
chr14	157176	160191	PF14_0788	hypothetical protein conserved	0.261
chr14	161227	162099	PF14_0044	hypothetical protein	-0.006
chr14	163990	166884	PF14_0045	hypothetical protein	-0.065
chr14	170000	170893	PF14_0046	hypothetical conserved in Plasmodium species	-0.252
chr14	172237	174911	PF14_0047	hypothetical protein	-0.091
chr14	176418	179705	PF14_0048	hypothetical protein	0.085
chr14	180897	181649	PF14_0049	hypothetical protein	-0.290
chr14	181955	185032	PF14_0050	hypothetical protein	0.091
chr14	185437	190368	PF14_0051	hypothetical protein conserved	0.174
chr14	190997	192724	PF14_0052	hypothetical protein conserved	-0.004
chr14	193216	194265	PF14_0053	ribonucleotide reductase small subunit	-0.331
chr14	196327	198486	PF14_0054	hypothetical protein conserved	-0.236
chr14	200493	204143	PF14_0055	hypothetical protein conserved	-0.005
chr14	205559	208876	PF14_0056	hypothetical protein	0.060
chr14	210300	210960	PF14_0057	RNA binding protein putative	-0.176
chr14	211566	215651	PF14_0058	hypothetical protein	0.031
chr14	217845	226307	PF14_0059	hypothetical protein	0.178
chr14	228410	229837	PF14_0060	glycerophodiester phosphodiesterase	-0.181
chr14	230864	232690	PF14_0061	hypothetical protein	0.012
chr14	233001	237744	PF14_0062	hypothetical protein	0.206
chr14	239747	243772	PF14_0063	ATP-dependent Clp protease putative	-0.023
chr14	244908	245492	PF14_0064	vacuolar protein sorting 29 putative	-0.330
chr14	246380	247721	PF14_0065	hypothetical protein	-0.426
chr14	251059	252826	PF14_0066	radical SAM protein putative	0.009
chr14	253542	257360	PF14_0067	hypothetical protein	-0.265
chr14	258746	259988	PF14_0068	fibrillarin putative	-0.316
chr14	261536	262099	PF14_0069	hypothetical protein	0.297
chr14	264403	267074	PF14_0070	hypothetical protein	-0.423

chr14	268108	268692	PF14_0071	hypothetical protein	0.107
chr14	269881	270927	PF14_0072	hypothetical protein conserved	0.247
chr14	271014	277115	PF14_0073	hypothetical protein	0.602
chr14	279176	280673	PF14_0074	hypothetical protein	-0.185
chr14	283086	284435	PF14_0075	plasmepsin putative	-0.570
chr14	288297	289655	PF14_0076	plasmepsin 1 precursor	-0.555
chr14	293471	294832	PF14_0077	plasmepsin 2	-0.380
chr14	297468	298823	PF14_0078	HAP protein	-0.311
chr14	300725	305833	PF14_0079	hypothetical protein	0.100
chr14	312569	312868	PF14_0080	hypothetical protein	0.373
chr14	313975	317457	PF14_0081	DNA repair helicase putative	0.017
chr14	320274	320861	PF14_0082	hypothetical protein	0.065
chr14	323317	324172	PF14_0083	ribosomal protein S8e putative	-0.676
chr14	324754	346468	PF14_0084	hypothetical protein	-0.019
chr14	349359	350219	PF14_0085	hypothetical protein	-0.157
chr14	351025	352047	PF14_0086	hypothetical protein	-0.400
chr14	353263	355141	PF14_0087	hypothetical protein	-0.237
chr14	356718	360188	PF14_0088	hypothetical protein	-0.438
chr14	360656	361539	PF14_0089	hypothetical protein	0.511
chr14	363092	364240	PF14_0090	hypothetical protein	-0.141
chr14	365509	365907	PF14_0091	hypothetical protein	-0.904
chr14	366484	367027	PF14_0092	hypothetical protein	-0.200
chr14	370146	373006	PF14_0093	hypothetical protein	-0.211
chr14	374800	377106	PF14_0094	hypothetical protein	0.185
chr14	377705	380182	PF14_0095	hypothetical protein	0.226
chr14	383188	384732	PF14_0096	hypothetical protein	-0.584
chr14	389592	391595	PF14_0097	cytidine diphosphate-diacylglycerol synthase	-0.053
chr14	392972	393850	PF14_0098	hypothetical protein	-0.072
chr14	396010	397967	PF14_0099	hypothetical protein conserved	0.076
chr14	400418	402994	PF14_0100	cytidine triphosphate synthetase	-0.530
chr14	403652	417062	PF14_0101	hypothetical protein	0.087
chr14	420442	422790	PF14_0102	rhoptry-associated protein 1 RAP1	-0.172
chr14	423751	424250	PF14_0103	hypothetical protein	0.181
chr14	427191	428588	PF14_0104	eukaryotictranslation initiation factor 2 gamma subunit p	-1.213
chr14	429690	430861	PF14_0105	hypothetical protein	0.209

chr14	433951	435318	PF14_0106	hypothetical protein	0.104
chr14	437224	439466	PF14_0107	hypothetical protein conserved	-0.196
chr14	440541	447010	PF14_0108	hypothetical protein	0.118
chr14	447923	448560	PF14_0109	hypothetical protein	-0.316
chr14	448629	449968	PF14_0778	hypothetical protein conserved	0.136
chr14	452458	454879	PF14_0110	rhomboid protease putative	0.291
chr14	458796	460451	PF14_0111	hypothetical protein	-0.375
chr14	461401	467451	PF14_0112	POM1 putative	0.050
chr14	468988	472016	PF14_0113	hypothetical protein	0.388
chr14	472943	474826	PF14_0114	GTP-binding protein putative	-0.057
chr14	475249	477297	PF14_0115	hypothetical protein	0.023
chr14	477469	478620	PF14_0116	hypothetical protein	0.346
chr14	480431	481414	PF14_0117	hypothetical protein conserved	-0.308
chr14	481591	481883	PF14_0118	hypothetical protein	0.102
chr14	482437	483399	PF14_0119	hypothetical protein conserved	-0.775
chr14	484438	487308	PF14_0120	hypothetical protein	-0.225
chr14	487776	490380	PF14_0121	hypothetical protein	0.060
chr14	491478	492221	PF14_0122	nuclear transport factor 2 putative	-0.265
chr14	493971	504345	PF14_0123	hypothetical protein	0.071
chr14	507360	508868	PF14_0124	actin II	-1.041
chr14	509686	511176	PF14_0125	deoxyhypusine synthase	-0.568
chr14	512363	516022	PF14_0126	hypothetical protein conserved	-0.275
chr14	516574	518193	PF14_0127	N-myristoyltransferase	-0.513
chr14	521209	522898	PF14_0128	ubiquitin conjugating enzyme putative	-0.163
chr14	523890	525305	PF14_0129	hypothetical protein	-0.065
chr14	527132	529234	PF14_0130	hypothetical protein	0.039
chr14	530135	531739	PF14_0131	hypothetical protein	0.202
chr14	534057	535727	PF14_0132	ribosomal protein S9 putative	-0.115
chr14	536796	537839	PF14_0133	ATP-dependent transporter putative	-0.758
chr14	538896	539810	PF14_0134	hypothetical protein	-0.160
chr14	541015	544199	PF14_0135	hypothetical protein	-0.020
chr14	547954	549417	PF14_0136	hypothetical protein conserved	0.016
chr14	551862	553235	PF14_0137	hypothetical protein	-0.175
chr14	553639	555772	PF14_0138	hypothetical protein	-0.006
chr14	560093	562324	PF14_0139	hypothetical protein	-0.262

chr14	564024	565247	PF14_0140	hypothetical protein	-0.018
chr14	570802	571461	PF14_0141	ribosomal protein L10 putative	-0.814
chr14	575495	577054	PF14_0142	serine%2Fthreonine protein phosphatase putative	-0.362
chr14	578387	587528	PF14_0143	protein kinase putative	-0.092
chr14	590625	592187	PF14_0144	mRNA capping enzyme putative	-0.353
chr14	592991	597145	PF14_0145	deubiquitinising protease putative	0.199
chr14	599762	600679	PF14_0146	ribonucleoprotein putative	-0.311
chr14	601980	605558	PF14_0147	ATP-dependent protease putative	0.011
chr14	606379	607347	PF14_0148	uracil-DNA glycosylase putative	-0.164
chr14	609204	611286	PF14_0149	hypothetical protein	0.048
chr14	611864	612735	PF14_0150	RNA polymerase small subunit putative	-0.357
chr14	615166	616179	PF14_0151	hypothetical protein	-0.642
chr14	618896	623320	PF14_0152	hypothetical protein	-0.039
chr14	625486	628249	PF14_0153	hypothetical protein	0.458
chr14	628785	632894	PF14_0154	hypothetical protein	0.111
chr14	634814	636532	PF14_0155	serine C-palmitoyltransferase putative	-0.755
chr14	640373	641518	PF14_0156	dimethyladenosine transferase putative	-0.297
chr14	642070	642734	PF14_0157	hypothetical protein	-0.268
chr14	643913	644988	PF14_0158	hypothetical protein	0.017
chr14	645499	648453	PF14_0159	hypothetical protein conserved	0.094
chr14	650502	657344	PF14_0160	hypothetical protein	-0.259
chr14	659538	660902	PF14_0161	hypothetical protein conserved	-0.191
chr14	661604	662378	PF14_0162	hypothetical protein	0.135
chr14	663529	665097	PF14_0163	hypothetical protein	0.053
chr14	667411	668823	PF14_0164	NADP-specific glutamate dehydrogenase	-0.535
chr14	669691	678771	PF14_0165	hypothetical protein	0.278
chr14	681176	681726	PF14_0805	hypothetical protein conserved	-0.498
chr14	682777	684801	PF14_0166	lysine -- tRNA ligase putative	-0.524
chr14	685436	686367	PF14_0167	hypothetical protein	-0.277
chr14	688591	689739	PF14_0168	hypothetical protein	-0.383
chr14	690450	691271	PF14_0169	hypothetical protein	-0.440
chr14	694584	708176	PF14_0170	hypothetical protein	-0.067
chr14	709669	713308	PF14_0171	hypothetical protein	-0.090
chr14	714707	723367	PF14_0172	hypothetical protein	0.063
chr14	723481	725997	PF14_0173	hypothetical protein	-0.142

chr14	726978	728378	PF14_0174	small nucleolar ribonucleoprotein snoRNP putative	-0.650
chr14	730693	744681	PF14_0175	conserved Plasmodium protein unknown function	-0.038
chr14	750329	751683	PF14_0176	hypothetical protein	-0.345
chr14	753388	756439	PF14_0177	DNA replication licensing factor MCM2	-0.702
chr14	759412	761324	PF14_0178	hypothetical protein	-0.115
chr14	762613	773406	PF14_0179	hypothetical protein	0.153
chr14	775459	775833	PF14_0180	hypothetical protein	-0.230
chr14	776671	777081	PF14_0181	calmodulin putative	-0.361
chr14	782104	785818	PF14_0183	RNA helicase putative	0.247
chr14	786957	790457	PF14_0184	hypothetical protein	0.238
chr14	793751	796876	PF14_0185	ATP-dependent RNA helicase putative	-0.219
chr14	797708	799465	PF14_0186	hypothetical protein	0.419
chr14	802716	803480	PF14_0187	glutathione S-transferase	-0.090
chr14	804564	811368	PF14_0188	hypothetical protein	0.223
chr14	813774	814409	PF14_0189	hypothetical protein	0.274
chr14	815320	818241	PF14_0190	hypothetical protein	0.238
chr14	820063	821592	PF14_0191	hypothetical protein	-0.101
chr14	822418	824197	PF14_0192	glutathione reductase	-0.685
chr14	825607	828109	PF14_0193	hypothetical protein	-0.077
chr14	828899	830768	PF14_0194	spliceosome-associated protein putative	-0.486
chr14	832330	836679	PF14_0195	hypothetical protein	-0.394
chr14	838922	842659	PF14_0196	hypothetical protein	0.104
chr14	843706	844614	PF14_0197	hypothetical protein	0.000
chr14	846533	849172	PF14_0198	glycine -- tRNA ligase putative	-0.510
chr14	849814	853295	PF14_0199	hypothetical protein	0.269
chr14	854069	855909	PF14_0200	hypothetical protein	-0.207
chr14	859957	863026	PF14_0201	surface protein putative Pf113	-0.196
chr14	863569	864766	PF14_0202	dynein-associated protein putative	0.276
chr14	865292	866751	PF14_0774	mannose-1-phosphate guanyltransferase putative	-0.422
chr14	867748	868866	PF14_0775	hypothetical protein	-0.233
chr14	870945	874706	PF14_0204	hypothetical protein	0.127
chr14	875632	876189	PF14_0205	ribosomal protein S25 putative	-0.544
chr14	877386	880247	PF14_0206	hypothetical protein	0.120
chr14	881974	883545	PF14_0207	RNA polymerase subunit putative	-0.109
chr14	884343	885019	PF14_0208	hypothetical protein conserved	-0.016

chr14	885156	887469	PF14_0209	hypothetical protein	0.137
chr14	888783	889898	PF14_0210	hypothetical protein	-0.110
chr14	891085	891567	PF14_0211	hypothetical protein	-0.171
chr14	891936	892994	PF14_0787	hypothetical protein	0.485
chr14	893665	894807	PF14_0212	hypothetical protein	-0.342
chr14	895949	898989	PF14_0213	hypothetical protein	0.291
chr14	900331	905223	PF14_0214	hypothetical protein	0.031
chr14	906565	908837	PF14_0215	hypothetical protein	-0.240
chr14	910172	912097	PF14_0216	hypothetical protein	-0.177
chr14	913764	917336	PF14_0217	hypothetical protein	-0.210
chr14	918374	919942	PF14_0218	actin-related protein homolog arp4 homolog	-0.137
chr14	922669	922866	PF14_0219	hypothetical protein	0.392
chr14	922876	924153	PF14_0220	hypothetical protein	-0.076
chr14	924909	926372	PF14_0221	Ran protein putative	-0.384
chr14	929448	931226	PF14_0222	hypothetical protein	-0.795
chr14	933004	935066	PF14_0223	cyclophilin putative	-0.181
chr14	936113	941583	PF14_0224	PP1-like protein serine%2Fthreonine phosphatase	-0.161
chr14	942137	946935	PF14_0225	hypothetical protein	0.576
chr14	947332	952293	PF14_0226	hypothetical protein	0.133
chr14	955810	958011	PF14_0227	calcium-dependent protein kinase putative	-0.101
chr14	959019	963695	PF14_0228	hypothetical protein	-0.191
chr14	967505	972253	PF14_0229	hypothetical protein	-0.076
chr14	973795	975485	PF14_0230	Ribosomal protein family L5 putative	-0.383
chr14	977850	979108	PF14_0231	ribosomal protein L7a putative	-0.262
chr14	983283	984503	PF14_0232	hypothetical protein conserved	0.198
chr14	985307	986817	PF14_0233	hypothetical protein	0.366
chr14	989162	992872	PF14_0234	DNA-directed DNA polymerase putative	-0.154
chr14	993594	994242	PF14_0235	hypothetical protein	0.326
chr14	995103	1000448	PF14_0236	hypothetical protein	0.843
chr14	1006585	1008011	PF14_0237	hypothetical protein	0.128
chr14	1008952	1011852	PF14_0238	hypothetical protein	0.058
chr14	1012638	1014207	PF14_0239	hypothetical protein	-0.263
chr14	1016075	1016560	PF14_0240	ribosomal protein L21e putative	-0.226
chr14	1018687	1019202	PF14_0241	basictranscription factor 3b putative	-0.264
chr14	1021594	1022799	PF14_0242	arginine n-methyltransferase putative	-0.180

chr14	1023890	1027086	PF14_0243	dynein-associated protein putative	0.005	
chr14	1027908	1029890	PF14_0244	ABC transporter %28EPP family%29	-0.298	
chr14	1031007	1035302	PF14_0245	hypothetical protein	0.332	
chr14	1036079	1039525	PF14_0246	phosphoenolpyruvate carboxylase putative	-0.007	
chr14	1042856	1049532	PF14_0247	hypothetical protein	0.004	
chr14	1049992	1050258	PF14_0248	ubiquinol-cytochrome c reductase hinge protein putative	-0.518	
chr14	1051027	1054536	PF14_0249	hypothetical protein	0.329	
chr14	1056104	1060066	PF14_0250	hypothetical protein	0.056	
chr14	1061747	1062386	PF14_0251	hypothetical protein	-0.219	
chr14	1064084	1069328	PF14_0252	hypothetical protein	0.387	14cenL
chr14	1077299	1078747	PF14_0253	hypothetical protein	0.565	14cenR
chr14	1081715	1084150	PF14_0254	DNA mismatch repair protein Msh2p putative	-0.019	
chr14	1085545	1086981	PF14_0255	hypothetical protein	0.002	
chr14	1088304	1089044	PF14_0256	exosome complex exonuclease rrp41 putative	-0.137	
chr14	1092878	1093723	PF14_0257	hypothetical protein conserved	0.463	
chr14	1095628	1097509	PF14_0258	hypothetical protein	0.177	
chr14	1097980	1099893	PF14_0259	hypothetical protein	0.243	
chr14	1101900	1104329	PF14_0260	metabolite%2Fdrug transporter	0.060	
chr14	1105142	1106456	PF14_0261	proliferation-associated protein 2g4 putative	-0.345	
chr14	1108487	1108947	PF14_0262	hypothetical protein	-0.126	
chr14	1109087	1115412	PF14_0263	hypothetical protein	0.342	
chr14	1118533	1126234	PF14_0264	protein kinase putative	0.057	
chr14	1126984	1128973	PF14_0265	peptide chain release factor 1 putative	0.040	
chr14	1129412	1130713	PF14_0266	hypothetical protein	0.061	
chr14	1133009	1134342	PF14_0267	transcription initiation TFIID-like putative	-0.193	
chr14	1134796	1139133	PF14_0268	hypothetical protein	-0.047	
chr14	1140521	1141234	PF14_0269	hypothetical protein conserved	-0.503	
chr14	1142096	1143733	PF14_0270	ribosomal protein L15 putative	-0.313	
chr14	1144460	1148020	PF14_0271	hypothetical protein	0.454	
chr14	1150589	1152360	PF14_0272	hypothetical protein	0.217	
chr14	1155297	1163636	PF14_0273	hypothetical protein	0.085	
chr14	1164274	1166508	PF14_0274	hypothetical protein conserved	-0.158	
chr14	1167086	1167745	PF14_0275	hypothetical protein	-0.084	
chr14	1168178	1169278	PF14_0276	ribosomal protein L15 putative	-0.306	
chr14	1170771	1175235	PF14_0277	coatamer protein beta subunit putative	-0.150	

chr14	1176712	1181034	PF14_0278	ATP-dependent DNA helicase putative	-0.229
chr14	1181725	1182924	PF14_0279	hypothetical protein	-0.041
chr14	1184852	1186984	PF14_0280	phosphotyrosyl phosphatase activator putative	-0.042
chr14	1188291	1191408	PF14_0281	aspartyl protease putative	-0.354
chr14	1191888	1199861	PF14_0282	hypothetical protein conserved	-0.378
chr14	1201690	1204281	PF14_0283	hypothetical protein	-0.361
chr14	1205030	1206358	PF14_0284	hypothetical protein	-0.152
chr14	1207340	1209970	PF14_0285	exodeoxyribonuclease III putative	0.063
chr14	1210954	1212718	PF14_0286	glutamate dehydrogenase putative	-0.319
chr14	1214877	1215674	PF14_0287	hypothetical protein	-0.617
chr14	1216595	1217113	PF14_0288	cytochrome c oxidase subunit II precursor putative	-0.571
chr14	1218047	1218941	PF14_0289	ribosomal protein L17 putative	-0.739
chr14	1221717	1222760	PF14_0290	hypothetical protein	-0.586
chr14	1224282	1227959	PF14_0291	hypothetical protein	0.271
chr14	1230978	1233783	PF14_0292	hypothetical protein conserved	-0.186
chr14	1233919	1237067	PF14_0293	hypothetical protein	-0.045
chr14	1238793	1241537	PF14_0294	mitogen-activated protein kinase 1 PfMAP1	-0.391
chr14	1243960	1245348	PF14_0295	ATP-specific succinyl-CoA synthetase beta subunit puta	-0.690
chr14	1246972	1247892	PF14_0296	ribosomal protein L14 putative	-0.114
chr14	1249270	1252109	PF14_0297	ecto-nucleoside triphosphate diphosphohydrolase 1 puta	-0.101
chr14	1253482	1254828	PF14_0298	hypothetical protein	-0.030
chr14	1256054	1256820	PF14_0299	hypothetical protein	-0.326
chr14	1257585	1258913	PF14_0300	syntaxin putative	0.422
chr14	1260850	1262768	PF14_0301	hypothetical protein	-0.068
chr14	1263768	1266665	PF14_0302	hypothetical protein	-0.220
chr14	1266953	1268953	PF14_0303	hypothetical protein	0.218
chr14	1269677	1274609	PF14_0304	hypothetical protein	0.126
chr14	1275841	1280733	PF14_0305	nuclear mRNA export factor TAP homolog putative	0.179
chr14	1280830	1281245	PF14_0306	hypothetical protein	-0.127
chr14	1281809	1282543	PF14_0785	hypothetical protein	0.582
chr14	1283512	1285005	PF14_0307	hypothetical protein conserved	-0.173
chr14	1285609	1289196	PF14_0308	XPC homolog	0.087
chr14	1290410	1291449	PF14_0309	protein-L-isoaspartate O-methyltransferase beta-asparta	-0.502
chr14	1292827	1295028	PF14_0310	hypothetical protein	-0.216
chr14	1296115	1297153	PF14_0311	hypothetical protein	-0.079

chr14	1298581	1298919	PF14_0784	Ribosome biogenesis protein NOP10-like	-0.527
chr14	1299836	1303288	PF14_0312	hypothetical protein	-0.033
chr14	1303970	1307773	PF14_0313	hypothetical protein	0.205
chr14	1309983	1311776	PF14_0314	chromatin assembly factor 1 p55 subunit putative	-0.431
chr14	1312246	1329519	PF14_0315	hypothetical protein	0.062
chr14	1332305	1337243	PF14_0316	DNA topoisomerase II putative	-0.253
chr14	1341632	1342805	PF14_0317	hypothetical protein conserved	0.059
chr14	1342956	1348937	PF14_0318	hypothetical protein	0.316
chr14	1349184	1358322	PF14_0319	hypothetical protein	0.094
chr14	1359945	1364501	PF14_0320	protein kinase putative	0.295
chr14	1365285	1366056	PF14_0321	ABC transporter putative	-0.284
chr14	1366922	1368145	PF14_0322	hypothetical protein	0.205
chr14	1368758	1369739	PF14_0323	calmodulin	-0.117
chr14	1372846	1374540	PF14_0324	hypothetical protein conserved	-0.483
chr14	1375567	1377707	PF14_0325	hypothetical protein	-0.072
chr14	1378291	1402608	PF14_0326	hypothetical protein	0.107
chr14	1404255	1406141	PF14_0327	methionine aminopeptidase type II putative	-0.745
chr14	1407201	1407689	PF14_0328	mitochondrial import inner membrane translocase subun	-0.839
chr14	1410082	1410840	PF14_0329	hypothetical protein conserved	-0.285
chr14	1411680	1413039	PF14_0330	hypothetical protein	-0.160
chr14	1413966	1415258	PF14_0331	cytochrome c oxidase assembly protein putative	-0.620
chr14	1415884	1416963	PF14_0332	hypothetical protein	0.060
chr14	1419326	1420877	PF14_0333	hypothetical protein	-0.542
chr14	1421844	1431164	PF14_0334	NAD%28P%29H-dependent glutamate synthase putativ	-0.629
chr14	1433681	1434253	PF14_0335	hypothetical protein conserved	0.011
chr14	1436223	1436987	PF14_0336	hypothetical protein	0.064
chr14	1437454	1442820	PF14_0337	hypothetical protein	0.024
chr14	1445028	1449434	PF14_0338	hypothetical protein	-0.385
chr14	1452547	1454061	PF14_0339	hypothetical protein	-0.273
chr14	1454498	1455352	PF14_0340	hypothetical protein	0.008
chr14	1458074	1459813	PF14_0341	glucose-6-phosphate isomerase	-0.326
chr14	1461412	1467533	PF14_0342	ion channel putative	-0.056
chr14	1467572	1474381	PF14_0343	hypothetical protein	-0.043
chr14	1479720	1482701	PF14_0344	hypothetical protein	-0.525
chr14	1484109	1489944	PF14_0345	hypothetical protein	0.200

chr14	1490597	1494157	PF14_0346	cGMP-dependent protein kinase 1 beta isozyme putativ	-0.365
chr14	1495742	1498476	PF14_0347	hypothetical protein	0.281
chr14	1498701	1499435	PF14_0348	ATP-dependent Clp protease proteolytic subunit putative	-0.006
chr14	1500071	1500908	PF14_0349	histidine triad protein putative	-0.058
chr14	1502591	1504427	PF14_0350	hypothetical protein	-0.031
chr14	1505849	1506546	PF14_0351	hypothetical protein	-0.037
chr14	1507302	1510032	PF14_0352	ribonucleoside-diphosphate reductase large subunit	-0.538
chr14	1513881	1514904	PF14_0353	hypothetical protein	0.310
chr14	1517013	1519313	PF14_0354	hypothetical protein	-0.205
chr14	1521048	1521863	PF14_0355	hypothetical protein	-0.094
chr14	1523229	1524989	PF14_0356	hypothetical protein	-0.013
chr14	1525507	1528428	PF14_0357	acetyl CoA synthetase putative	-0.337
chr14	1530219	1530773	PF14_0358	41-2 protein antigen precursor	-0.419
chr14	1533272	1534890	PF14_0359	hypothetical protein conserved	-0.206
chr14	1536427	1538412	PF14_0360	hypothetical protein	-0.310
chr14	1541570	1542577	PF14_0361	translocation protein sec62 putative	-0.532
chr14	1548861	1549130	PF14_0362	hypothetical protein	0.055
chr14	1550032	1556473	PF14_0363	metacaspase-like protein	-0.089
chr14	1557470	1560100	PF14_0364	cleavage and polyadenylation specifity factor protein put	-0.299
chr14	1562175	1563008	PF14_0365	hypothetical protein	-0.456
chr14	1564518	1567782	PF14_0366	small subunit DNA primase	-0.081
chr14	1570982	1574104	PF14_0367	hypothetical protein	-0.458
chr14	1575927	1576514	PF14_0368	2-Cys peroxiredoxin	-0.790
chr14	1578136	1579118	PF14_0369	hypothetical protein	-0.137
chr14	1580693	1588800	PF14_0370	RNA helicase putative	0.171
chr14	1588895	1593074	PF14_0371	hypothetical protein	0.327
chr14	1594448	1600450	PF14_0372	hypothetical protein	0.163
chr14	1600876	1601943	PF14_0373	iron-sulphur protein subunit of the cytochrome bc1 comp	-0.799
chr14	1608462	1612052	PF14_0374	hypothetical protein	-0.226
chr14	1612464	1613006	PF14_0375	hypothetical protein	-0.064
chr14	1613796	1615485	PF14_0376	hypothetical protein	0.268
chr14	1618762	1619737	PF14_0377	vesicle-associated membrane protein putative	-0.309
chr14	1622519	1623555	PF14_0378	triose-phosphate isomerase	-0.361
chr14	1624130	1631310	PF14_0379	hypothetical protein	0.048
chr14	1633154	1636198	PF14_0380	hypothetical protein	-0.193

chr14	1639420	1641703	PF14_0381	delta-aminolevulinic acid dehydratase	-0.011
chr14	1641976	1647110	PF14_0382	metalloendopeptidase putative	-0.076
chr14	1647616	1651637	PF14_0383	hypothetical protein	0.034
chr14	1653990	1655651	PF14_0384	hypothetical protein	-0.245
chr14	1656377	1659469	PF14_0385	hypothetical protein	-0.016
chr14	1660038	1662239	PF14_0386	hypothetical protein	-0.021
chr14	1665406	1667486	PF14_0387	metabolite%2Fdrug transporter	0.080
chr14	1668534	1669906	PF14_0388	hypothetical protein	0.083
chr14	1669923	1671593	PF14_0389	hypothetical protein	0.135
chr14	1673329	1674012	PF14_0390	hypothetical protein	0.592
chr14	1678605	1679429	PF14_0391	ribosomal protein L1 putative	-0.303
chr14	1681734	1689100	PF14_0392	Ser%2FThr protein kinase putative	-0.264
chr14	1690936	1692456	PF14_0393	structure specific recognition protein putative	-0.316
chr14	1693259	1694846	PF14_0394	hypothetical protein	0.337
chr14	1696247	1697956	PF14_0395	hypothetical protein conserved	0.064
chr14	1700617	1701993	PF14_0396	hypothetical protein	0.098
chr14	1702552	1703220	PF14_0397	hypothetical protein conserved	-0.385
chr14	1704537	1704740	PF14_0398	hypothetical protein	-0.152
chr14	1705069	1705726	PF14_0399	ADP-ribosylation-like factor putative	-0.182
chr14	1706735	1707455	PF14_0783	hypothetical protein	0.227
chr14	1708471	1711473	PF14_0400	hypothetical protein conserved	0.044
chr14	1712569	1714008	PF14_0401	methionine -- tRNA ligase putative	-0.511
chr14	1717114	1723455	PF14_0402	hypothetical protein	0.039
chr14	1724342	1727356	PF14_0403	protein prenyltransferase alpha subunit putative	0.130
chr14	1730345	1740859	PF14_0404	hypothetical protein	-0.239
chr14	1742056	1749733	PF14_0405	hypothetical protein	-0.012
chr14	1750152	1751360	PF14_0406	hypothetical protein	0.210
chr14	1753453	1763607	PF14_0407	hypothetical protein	-0.026
chr14	1764259	1768065	PF14_0408	Ser%2FThr protein kinase putative	0.116
chr14	1769569	1771293	PF14_0409	hypothetical protein	-0.124
chr14	1773219	1775140	PF14_0410	hypothetical protein	-0.019
chr14	1775953	1776605	PF14_0411	small nuclear ribonuclear protein putative	0.189
chr14	1776901	1779549	PF14_0412	hypothetical protein	-0.107
chr14	1780935	1783523	PF14_0413	hypothetical protein	-0.074
chr14	1784876	1790572	PF14_0414	hypothetical protein conserved	0.135

chr14	1791854	1792947	PF14_0415	dephospho-CoA kinase putative	0.090
chr14	1794651	1795877	PF14_0416	hypothetical protein	-0.079
chr14	1797231	1800014	PF14_0417	heat shock protein putative	-0.390
chr14	1800774	1801830	PF14_0418	hypothetical protein	0.293
chr14	1801904	1825269	PF14_0419	hypothetical protein	0.274
chr14	1826070	1827106	PF14_0420	hypothetical protein	-0.024
chr14	1828603	1829905	PF14_0421	1-acylGlycerol-3-phosphate O-acyltransferase	-0.446
chr14	1830952	1832746	PF14_0422	hypothetical protein	0.208
chr14	1833725	1838541	PF14_0423	Ser%2FThr protein kinase putative	0.130
chr14	1841022	1841531	PF14_0781	hypothetical protein	0.365
chr14	1841903	1842659	PF14_0424	hypothetical protein	0.124
chr14	1843984	1845560	PF14_0425	fructose-bisphosphate aldolase	-0.972
chr14	1848258	1848776	PF14_0426	hypothetical protein	0.326
chr14	1849115	1850335	PF14_0427	hypothetical protein	0.056
chr14	1851599	1854997	PF14_0428	histidine -- tRNA ligase putative	-0.041
chr14	1856643	1859251	PF14_0429	RNA helicase putative	0.244
chr14	1860091	1862038	PF14_0430	hypothetical protein	-0.333
chr14	1862718	1865363	PF14_0431	serine%2Fthreonine kinase-1 PfLammer	-0.394
chr14	1867559	1868008	PF14_0432	hypothetical protein	-0.013
chr14	1871721	1874324	PF14_0433	hypothetical protein	-0.264
chr14	1875364	1876817	PF14_0434	hypothetical protein	-0.058
chr14	1878601	1882520	PF14_0435	hypothetical protein	0.067
chr14	1885386	1886900	PF14_0436	helicase truncated putative	0.428
chr14	1887473	1888266	PF14_0437	helicase truncated putative	-0.133
chr14	1889356	1889472	U5RNA	U5RNA	0.708
chr14	1889921	1892051	PF14_0438	hypothetical protein	0.319
chr14	1893143	1894960	PF14_0439	leucine aminopeptidase putative	-0.702
chr14	1898294	1901869	PF14_0440	hypothetical protein	-0.048
chr14	1902859	1904034	PF14_0441	pyruvate dehydrogenase E1 beta subunit putative	-0.588
chr14	1907140	1916145	PF14_0442	hypothetical protein	-0.100
chr14	1917175	1917953	PF14_0443	centrin putative	-0.258
chr14	1919643	1920758	PF14_0444	hypothetical protein	0.124
chr14	1920913	1921588	PF14_0445	hypothetical protein	-0.051
chr14	1922902	1923725	PF14_0446	hypothetical protein	-0.682
chr14	1924368	1926593	PF14_0447	glutaminyl-peptide cyclotransferase	-0.056

chr14	1929874	1931322	PF14_0448	ribosomal protein S2 putative	-0.178
chr14	1932364	1933143	PF14_0449	hypothetical protein	0.021
chr14	1933928	1935718	PF14_0450	hypothetical protein	-0.144
chr14	1936831	1937317	PF14_0451	mitochondrial ribosomal protein S14 precursor putative	-0.344
chr14	1937649	1939060	PF14_0790	hypothetical protein conserved	0.148
chr14	1941163	1941629	PF14_0452	hypothetical protein	-0.276
chr14	1943879	1944907	PF14_0453	hypothetical protein	0.212
chr14	1947189	1953149	PF14_0454	hypothetical protein	0.095
chr14	1954544	1957618	PF14_0455	multidrug resistance protein 2 %28heavy metal transport	-0.290
chr14	1959976	1963905	PF14_0456	hypothetical protein conserved	-0.007
chr14	1965118	1965705	PF14_0457	hypothetical protein	-0.120
chr14	1966188	1968732	PF14_0458	hypothetical protein	0.117
chr14	1969077	1971346	PF14_0460	hypothetical protein conserved	-0.154
chr14	1972441	1977366	PF14_0461	hypothetical protein	0.097
chr14	1980262	1983050	PF14_0462	SEL-1 protein putative	-0.119
chr14	1984132	1995517	PF14_0463	chloroquine resistance marker protein	0.075
chr14	1997485	1998246	PF14_0464	hypothetical protein	-0.183
chr14	1999591	2000592	PF14_0465	hypothetical protein	-0.427
chr14	2001026	2002049	PF14_0466	hypothetical protein	0.098
chr14	2002864	2003187	PF14_0815	hypothetical protein	0.065
chr14	2004349	2005399	PF14_0467	hypothetical protein	-0.349
chr14	2008374	2012437	PF14_0468	hypothetical protein	-0.186
chr14	2016901	2019503	PF14_0469	transcription factor IIIb subunit putative	-0.696
chr14	2020152	2024648	PF14_0470	hypothetical protein	-0.132
chr14	2031024	2033334	PF14_0471	hypothetical protein	-0.086
chr14	2035053	2036567	PF14_0472	hypothetical protein	-0.057
chr14	2038069	2041479	PF14_0473	3%27-5%27 exonuclease putative	0.132
chr14	2042946	2043479	PF14_0474	hypothetical protein	-0.481
chr14	2043944	2044966	PF14_0475	hypothetical protein	-0.006
chr14	2047730	2050020	PF14_0476	serine%2Fthreonine protein kinase putative	0.178
chr14	2051558	2053060	PF14_0477	signal recognition particle 54 kDa protein putative	-0.582
chr14	2056578	2059175	PF14_0478	hypothetical protein	-0.011
chr14	2059873	2065707	PF14_0479	hypothetical protein	0.119
chr14	2067676	2073593	PF14_0480	hypothetical protein	0.083
chr14	2075523	2077286	PF14_0481	hypothetical protein conserved	-0.008

chr14	2078425	2080287	PF14_0482	hypothetical protein conserved	-0.220
chr14	2080835	2082184	PF14_0483	hypothetical protein	-0.167
chr14	2083168	2084602	PF14_0484	acetyl-CoA acetyltransferase putative	-0.428
chr14	2085042	2087456	PF14_0485	hypothetical protein	0.265
chr14	2090839	2093337	PF14_0486	elongation factor 2	-0.991
chr14	2094277	2099673	PF14_0487	hypothetical protein	-0.009
chr14	2104883	2106817	PF14_0488	hypothetical protein	0.156
chr14	2108810	2112724	PF14_0489	histone deacetylase putative	-0.127
chr14	2113497	2113940	PF14_0490	hypothetical protein	0.132
chr14	2115372	2118037	PF14_0491	hypothetical protein	-0.431
chr14	2118883	2120588	PF14_0492	protein phosphatase 2b regulatory subunit putative	0.154
chr14	2123397	2126535	PF14_0493	sortilin putative	-0.477
chr14	2128104	2131193	PF14_0494	hypothetical protein conserved	-0.174
chr14	2134234	2140803	PF14_0495	hypothetical protein	-0.503
chr14	2141598	2142614	PF14_0496	leucine-rich repeat protein 2 LRR2	0.162
chr14	2143238	2144812	PF14_0497	hypothetical protein	0.216
chr14	2145102	2146166	PF14_0498	hypothetical protein	0.011
chr14	2148357	2151059	PF14_0499	hypothetical protein	-0.233
chr14	2152070	2153550	PF14_0500	hypothetical protein	0.158
chr14	2155672	2166403	PF14_0501	hypothetical protein	0.251
chr14	2167353	2169476	PF14_0502	hypothetical protein	-0.230
chr14	2170397	2171379	PF14_0503	hypothetical protein	0.218
chr14	2171805	2176648	PF14_0504	hypothetical protein	0.166
chr14	2176773	2178665	PF14_0505	hypothetical protein	-0.214
chr14	2180351	2186567	PF14_0506	hypothetical protein	0.043
chr14	2188114	2193885	PF14_0507	hypothetical protein	0.169
chr14	2194646	2198416	PF14_0508	pyridine nucleotide transhydrogenase putative	-0.746
chr14	2199307	2204644	PF14_0509	hypothetical protein	0.251
chr14	2206963	2208256	PF14_0510	hypothetical protein	-0.517
chr14	2209728	2212460	PF14_0511	glucose-6-phosphatedehydrogenase-6-phosphogluco no	-0.209
chr14	2214846	2217731	PF14_0512	hypothetical protein	-0.240
chr14	2219016	2220551	PF14_0513	RNA binding protein putative	0.060
chr14	2221287	2221820	PF14_0514	tRNA intron endonuclease putative	0.219
chr14	2222426	2226640	PF14_0515	hypothetical protein	0.160
chr14	2229220	2231517	PF14_0516	serine%2Fthreonine-protein kinase	-0.228

chr14	2234118	2236412	PF14_0517	peptidase putative	-0.368
chr14	2238330	2238818	PF14_0518	nifU protein putative	-0.281
chr14	2239474	2240163	PF14_0519	ribosomal protein S11 putative	-0.587
chr14	2241369	2242775	PF14_0520	6-phosphogluconate dehydrogenase decarboxylating pu	-0.583
chr14	2243809	2244303	PF14_0521	hypothetical protein	0.157
chr14	2245036	2245500	PF14_0522	hypothetical protein	-0.308
chr14	2247181	2248502	PF14_0523	protein phosphatase 2C putative	0.138
chr14	2251124	2251562	PF14_0524	protein phosphatase 7 homolog putative	0.127
chr14	2251601	2251870	PF14_0525	hypothetical protein	-0.582
chr14	2255135	2256677	PF14_0526	hypothetical protein conserved	-0.152
chr14	2259401	2261233	PF14_0527	hypothetical protein	-0.554
chr14	2262675	2263737	PF14_0528	hemolysin putative	-0.290
chr14	2267137	2270787	PF14_0529	gamma-adaptin putative	-0.179
chr14	2271465	2277179	PF14_0530	ferlin putative	-0.097
chr14	2278637	2282098	PF14_0531	hypothetical protein	0.169
chr14	2285049	2290163	PF14_0532	hypothetical protein	-0.227
chr14	2290823	2292627	PF14_0791	dfg10 like protein putative	0.277
chr14	2296427	2300804	PF14_0533	hypothetical protein	0.025
chr14	2301392	2302780	PF14_0534	serine hydroxymethyltransferase	0.023
chr14	2303501	2306404	PF14_0535	hypothetical protein	0.065
chr14	2306891	2308138	PF14_0536	hypothetical protein	0.108
chr14	2309117	2312408	PF14_0537	hypothetical protein	0.041
chr14	2314316	2321491	PF14_0538	hypothetical protein	-0.050
chr14	2321889	2322734	PF14_0539	hypothetical protein	-0.109
chr14	2323708	2325762	PF14_0540	hypothetical protein	-0.073
chr14	2329869	2332022	PF14_0541	V-type H%28%2B%29-translocating pyrophosphatase p	-1.047
chr14	2335064	2335898	PF14_0542	hypothetical protein conserved	0.020
chr14	2336585	2339089	PF14_0543	signal peptide peptidase mSPP	0.047
chr14	2340720	2342963	PF14_0544	hypothetical protein	-0.362
chr14	2343576	2344154	PF14_0545	thioredoxin	-0.129
chr14	2346114	2348033	PF14_0546	hypothetical protein conserved	-0.176
chr14	2350921	2360088	PF14_0547	hypothetical protein	0.225
chr14	2360810	2362069	PF14_0548	ATPase putative	-0.370
chr14	2363514	2365306	PF14_0549	hypothetical protein	-0.160
chr14	2367223	2371659	PF14_0550	hypothetical protein	0.158

chr14	2372169	2373680	PF14_0551	hypothetical protein	0.130
chr14	2375515	2385882	PF14_0552	hypothetical protein	0.121
chr14	2387722	2389431	PF14_0553	cysteine proteinase falcipain-1	-0.103
chr14	2390662	2390838	PF14_0554	hypothetical protein	0.174
chr14	2391054	2391537	PF14_0555	hypothetical protein	0.162
chr14	2391719	2396018	PF14_0556	hypothetical protein	0.022
chr14	2397855	2398805	PF14_0557	hypothetical protein	-0.338
chr14	2400451	2405347	PF14_0558	hypothetical protein	0.043
chr14	2406604	2410095	PF14_0559	hypothetical protein	0.001
chr14	2410890	2411951	PF14_0560	hypothetical protein	-0.253
chr14	2414806	2417403	PF14_0561	hypothetical protein	0.214
chr14	2418358	2419234	PF14_0562	hypothetical protein	-0.039
chr14	2420700	2422925	PF14_0563	DEAD-box RNA helicase putative	-0.653
chr14	2423843	2426236	PF14_0564	hypothetical protein conserved	-0.045
chr14	2427160	2430027	PF14_0565	hypothetical protein	0.310
chr14	2432113	2432682	PF14_0566	hypothetical protein	0.345
chr14	2433297	2434319	PF14_0567	hypothetical protein	-0.047
chr14	2436631	2439552	PF14_0568	hypothetical protein	0.166
chr14	2441281	2443832	PF14_0569	hypothetical protein	0.222
chr14	2444674	2445710	PF14_0570	pyridoxamine-phosphate oxidase	-0.551
chr14	2446919	2449225	PF14_0571	hypothetical protein	-0.079
chr14	2450143	2450724	PF14_0572	hypothetical protein	-0.406
chr14	2452406	2453293	PF14_0573	hypothetical protein	-0.140
chr14	2454838	2455815	PF14_0574	hypothetical protein	-0.265
chr14	2456815	2457738	PF14_0575	hypothetical protein	0.517
chr14	2461257	2463040	PF14_0576	ubiquitin carboxyl-terminal hydrolase putative	-0.118
chr14	2466027	2470925	PF14_0577	hypothetical protein	-0.136
chr14	2473694	2474140	PF14_0578	hypothetical protein	-0.224
chr14	2475603	2476409	PF14_0579	ribosomal protein L27 putative	-0.192
chr14	2477764	2479671	PF14_0580	hypothetical protein	0.064
chr14	2480376	2481852	PF14_0581	ribosomal protein S10 putative	-0.024
chr14	2482711	2485022	PF14_0582	hypothetical protein	0.328
chr14	2485800	2488556	PF14_0583	hypothetical protein	0.250
chr14	2489634	2490184	PF14_0584	ribosomal protein S4 putative	-0.277
chr14	2492101	2492304	PF14_0585	ribosomal protein S28e putative	-0.338

chr14	2493426	2494697	PF14_0586	hypothetical protein	-0.256
chr14	2497982	2500319	PF14_0587	hypothetical protein	-0.370
chr14	2501513	2502450	PF14_0792	hypothetical protein conserved	0.305
chr14	2504084	2512440	PF14_0588	hypothetical protein	-0.085
chr14	2514608	2517880	PF14_0589	valine - tRNA ligase putative	-0.443
chr14	2518327	2518692	PF14_0590	hypothetical protein	-0.175
chr14	2519140	2523144	PF14_0591	hypothetical protein	0.207
chr14	2523854	2524075	PF14_0592	hypothetical protein	-0.494
chr14	2524856	2528929	PF14_0593	hypothetical protein	0.006
chr14	2531367	2541335	PF14_0594	hypothetical protein	0.051
chr14	2548371	2550329	PF14_0595	hypothetical protein conserved	0.973
chr14	2550627	2552367	PF14_0596	hypothetical protein	0.561
chr14	2553649	2555039	PF14_0597	cytochrome c1 precursor putative	-0.872
chr14	2559033	2560282	PF14_0598	glyceraldehyde-3-phosphate dehydrogenase	-0.797
chr14	2561004	2563427	PF14_0599	hypothetical protein	0.156
chr14	2565292	2566218	PF14_0600	hypothetical protein	0.344
chr14	2570369	2571654	PF14_0601	replication factor C3	-0.280
chr14	2572923	2574542	PF14_0602	DNA polymerase alpha subunit putative	0.021
chr14	2575261	2576271	PF14_0603	hypothetical protein	-0.172
chr14	2577909	2579314	PF14_0604	hypothetical protein	-0.062
chr14	2581478	2582458	PF14_0605	cyclin putative	0.169
chr14	2583122	2583484	PF14_0606	hypothetical protein conserved	0.401
chr14	2584735	2589717	PF14_0607	hypothetical protein	-0.002
chr14	2591937	2594054	PF14_0608	hypothetical protein	0.192
chr14	2595804	2598506	PF14_0609	hypothetical protein	0.190
chr14	2598777	2599889	PF14_0610	hypothetical protein	-0.007
chr14	2601376	2604240	PF14_0611	hypothetical protein	0.160
chr14	2604439	2604918	PF14_0612	hypothetical protein	0.141
chr14	2607227	2617556	PF14_0613	hypothetical protein	0.060
chr14	2619614	2624122	PF14_0614	hypothetical protein	-0.212
chr14	2625387	2626535	PF14_0615	ATP synthase %28C%2FAC39%29 subunit putative	0.330
chr14	2630493	2632613	PF14_0616	i-AAA protease putative	-0.538
chr14	2635776	2636612	PF14_0617	hypothetical protein	-0.150
chr14	2637501	2640104	PF14_0618	hypothetical protein	0.173
chr14	2640807	2641937	PF14_0619	hypothetical protein	0.021

chr14	2642939	2646127	PF14_0620	hypothetical protein	-0.206
chr14	2646538	2646738	PF14_0621	hypothetical protein	-0.241
chr14	2650455	2655075	PF14_0622	hypothetical protein	0.321
chr14	2655426	2656674	PF14_0623	hypothetical protein	0.067
chr14	2657418	2657900	PF14_0624	hypothetical protein	-0.172
chr14	2659818	2660846	PF14_0625	hypothetical protein	0.255
chr14	2661956	2682641	PF14_0626	dynein beta chain putative	0.104
chr14	2684077	2685470	PF14_0627	ribosomal protein S3 putative	-0.027
chr14	2686330	2686805	PF14_0628	hypothetical protein	0.314
chr14	2686879	2688476	PF14_0629	hypothetical protein	0.219
chr14	2690440	2693262	PF14_0630	protein serine%2Fthreonine phosphatase	-0.812
chr14	2694749	2703985	PF14_0631	hypothetical protein	-0.291
chr14	2709354	2713128	PF14_0632	26S proteasome subunit putative	-0.460
chr14	2714780	2717221	PF14_0633	hypothetical protein	0.017
chr14	2726736	2727578	PF14_0634	hypothetical protein	0.055
chr14	2729397	2730747	PF14_0635	RNA binding protein putative	0.068
chr14	2731922	2734630	PF14_0636	hypothetical protein	-0.365
chr14	2736844	2742133	PF14_0637	rhoptry protein putative	0.088
chr14	2742323	2744905	PF14_0638	hypothetical protein	0.149
chr14	2745751	2747256	PF14_0639	DNA-3-methyladenine glycosylase putative	-0.270
chr14	2748328	2750067	PF14_0640	hypothetical protein	-0.025
chr14	2750894	2752360	PF14_0641	1-deoxy-D-xylulose 5-phosphate reductoisomerase	-0.327
chr14	2753072	2753956	PF14_0642	hypothetical protein	0.031
chr14	2755161	2756735	PF14_0643	hypothetical protein conserved	-0.132
chr14	2757601	2764086	PF14_0644	hypothetical protein	0.060
chr14	2766044	2766552	PF14_0645	hypothetical protein	-0.133
chr14	2767498	2767871	PF14_0646	hypothetical protein	-0.061
chr14	2771037	2776244	PF14_0647	hypothetical protein	-0.096
chr14	2777568	2785050	PF14_0648	hypothetical protein	0.212
chr14	2786244	2794259	PF14_0649	hypothetical protein	-0.228
chr14	2802444	2803124	PF14_0650	hypothetical protein	-0.131
chr14	2803529	2804549	PF14_0651	leucine-rich repeat protein 14.2	0.166
chr14	2809414	2812002	PF14_0652	hypothetical protein	-0.078
chr14	2812982	2814321	PF14_0653	hypothetical protein	-0.224
chr14	2816036	2821807	PF14_0654	aminophospholipid transporting P_ATPase putative	0.478

chr14	2825306	2826854	PF14_0655	RNA helicase-1 putative	-0.567
chr14	2827878	2830379	PF14_0656	U2 snRNP auxiliary factor putative	-0.159
chr14	2831864	2834305	PF14_0657	hypothetical protein	0.094
chr14	2835539	2836087	PF14_0658	translation initiation factor EF-1 putative	0.386
chr14	2837255	2838037	PF14_0659	hypothetical protein	0.222
chr14	2838849	2839925	PF14_0660	hypothetical protein	-0.100
chr14	2841058	2841846	PF14_0661	hypothetical protein conserved	0.222
chr14	2845139	2846452	PF14_0662	hypothetical protein	-0.314
chr14	2847171	2849234	PF14_0663	hypothetical protein	0.376
chr14	2852179	2862504	PF14_0664	biotin carboxylase subunit of acetyl CoA carboxylase pu	0.897
chr14	2863850	2864467	PF14_0665	hypothetical protein	0.108
chr14	2864960	2866181	PF14_0666	hypothetical protein	-0.253
chr14	2866294	2868119	PF14_0667	hypothetical protein	0.003
chr14	2870005	2877150	PF14_0668	hypothetical protein	0.135
chr14	2877963	2878684	PF14_0669	hypothetical protein	-0.001
chr14	2879394	2879840	PF14_0670	hypothetical protein	-0.309
chr14	2880467	2880948	PF14_0671	hypothetical protein	-0.083
chr14	2882702	2886018	PF14_0672	cyclic nucleotide phosphodiesterase putative	-0.128
chr14	2886824	2889143	PF14_0673	hypothetical protein	0.079
chr14	2889992	2891734	PF14_0674	hypothetical protein	0.203
chr14	2892159	2895937	PF14_0675	reticulocyte binding protein 2 homolog B putative	0.232
chr14	2897854	2898595	PF14_0676	20S proteasome beta 4 subunit putative	-0.502
chr14	2902072	2903475	PF14_0677	RNA 3%27-Terminal Phosphate Cyclase-like protein pu	-0.269
chr14	2905101	2906503	PF14_0678	exported protein 2	-0.283
chr14	2912323	2915291	PF14_0679	inorganic anion exchanger inorganic anion antiporter	-0.277
chr14	2915684	2917943	PF14_0680	hypothetical protein	0.188
chr14	2919127	2920952	PF14_0681	diacylglycerol kinase putative	-0.366
chr14	2922288	2924015	PF14_0682	hypothetical protein	-0.494
chr14	2926071	2929610	PF14_0683	hypothetical protein	0.563
chr14	2929760	2931484	PF14_0684	hypothetical protein	0.190
chr14	2931960	2932724	PF14_0685	hypothetical protein	-0.216
chr14	2933099	2937040	PF14_0686	hypothetical protein	0.183
chr14	2937554	2939716	PF14_0687	hypothetical protein	0.083
chr14	2940415	2941540	PF14_0688	hypothetical protein conserved	-0.195
chr14	2942270	2943196	PF14_0689	hypothetical protein	0.104

chr14	2945599	2952488	PF14_0690	histone deactylase putative	-0.139
chr14	2954045	2956284	PF14_0691	hypothetical protein	0.182
chr14	2957411	2961555	PF14_0692	hypothetical protein	0.032
chr14	2961659	2962582	PF14_0693	hypothetical protein	-0.006
chr14	2962923	2965023	PF14_0694	protein disulfide isomerase putative	0.175
chr14	2966794	2969271	PF14_0695	DNA-directed RNA polymerase alpha subunit truncated	-0.300
chr14	2969849	2971729	PF14_0696	hypothetical protein	-0.054
chr14	2972915	2973991	PF14_0697	dihydroorotase putative	-0.307
chr14	2975052	2975429	PF14_0698	hypothetical protein	-0.258
chr14	2976148	2978054	PF14_0699	hypothetical protein conserved	0.066
chr14	2981537	2982901	PF14_0700	hypothetical protein conserved	-0.154
chr14	2983999	2985608	PF14_0701	hypothetical protein	0.188
chr14	2985864	2988181	PF14_0702	hypothetical protein	0.114
chr14	2989514	2992159	PF14_0703	hypothetical protein	-0.251
chr14	2993189	2995216	PF14_0704	hypothetical protein	-0.097
chr14	2997535	2997879	PF14_0705	hypothetical protein	-0.491
chr14	2998529	3002290	PF14_0706	hypothetical protein	0.395
chr14	3007536	3010094	PF14_0707	hypothetical protein	0.043
chr14	3011520	3017834	PF14_0708	hypothetical protein	0.586
chr14	3023163	3023661	PF14_0709	ribosomal protein L20 putative	0.836
chr14	3023787	3037703	PF14_0710	hypothetical protein	1.108
chr14	3040703	3043495	PF14_0711	hypothetical protein conserved	-0.010
chr14	3044064	3054994	PF14_0712	hypothetical protein	0.226
chr14	3055650	3057755	PF14_0713	hypothetical protein	0.118
chr14	3060799	3062569	PF14_0714	hypothetical protein conserved	0.029
chr14	3063614	3065698	PF14_0715	hypothetical protein	0.224
chr14	3067497	3068627	PF14_0716	Proteosome subunit alpha type 1 putative	-0.201
chr14	3072196	3073182	PF14_0717	hypothetical protein	-0.238
chr14	3075723	3076121	PF14_0718	hypothetical protein conserved	0.670
chr14	3076377	3080957	PF14_0719	hypothetical protein	0.247
chr14	3081685	3084570	PF14_0720	hypothetical protein	0.279
chr14	3085431	3086117	PF14_0721	cytochrome c oxidase assembly protein putative	0.090
chr14	3086996	3105006	PF14_0722	hypothetical protein	4.221
chr14	3106884	3111746	PF14_0723	hypothetical protein	3.945
chr14	3112444	3114633	PF14_0724	hypothetical protein	0.476

chr14	3117327	3118886	PF14_0725	tubulin putative	0.439
chr14	3120789	3121784	PF14_0726	hypothetical protein	0.968
chr14	3124272	3126644	PF14_0727	hypothetical protein	0.550
chr14	3127612	3128054	PF14_0728	hypothetical protein	0.102
chr14	3131039	3131572	PF14_0729	early transcribed membrane protein 14.2 etramp14.2	0.275
chr14	3133886	3134149	PF14_0730	hypothetical protein	0.119
chr14	3134459	3135586	PF14_0731	hypothetical protein	0.106
chr14	3139075	3140917	PF14_0732	hypothetical protein	0.170
chr14	3142010	3143256	PF14_0733	protein kinase FIKK family	0.062
chr14	3143425	3144276	PF14_0734	protein kianse FIKK family	0.306
chr14	3147425	3148519	PF14_0735	hypothetical protein	2.874
chr14	3151938	3154858	PF14_0736	hypothetical protein	3.932
chr14	3156911	3157972	PF14_0737	lysophospholipase putative	0.431
chr14	3159422	3160537	PF14_0738	lysophospholipase putative	0.482
chr14	3161783	3162064	PF14_0739	hypothetical protein	0.002
chr14	3168498	3170028	PF14_0740	hypothetical protein	4.224
chr14	3170158	3171627	PF14_0741	hypothetical protein	4.360
chr14	3172907	3173579	PF14_0742	hypothetical protein	4.189
chr14	3175803	3176393	PF14_0743	hypothetical protein	4.624
chr14	3179559	3180545	PF14_0744	hypothetical protein	4.928
chr14	3183574	3184287	PF14_0745	hypothetical protein	4.639
chr14	3188823	3190483	PF14_0746	hypothetical protein	0.434
chr14	3193220	3199413	PF14_0747	hypothetical protein	3.387
chr14	3202516	3203952	PF14_0748	hypothetical protein	3.016
chr14	3207562	3207834	PF14_0749	acyl CoA binding protein	1.147
chr14	3209064	3211509	PF14_0751	fatty acyl coenzyme A synthetase-1 putative	2.708
chr14	3213516	3214552	PF14_0752	hypothetical protein	3.185
chr14	3216334	3217231	PF14_0753	hypothetical protein	1.570
chr14	3219854	3220204	PF14_0754	hypothetical protein	3.018
chr14	3220861	3221511	PF14_0755	hypothetical protein	5.074
chr14	3223223	3224567	PF14_0756	hypothetical protein	4.003
chr14	3228607	3229524	PF14_0757	hypothetical protein	3.218
chr14	3232574	3236470	PF14_0758	hypothetical protein	2.174
chr14	3239080	3239535	PF14_0759	hypothetical protein	4.825
chr14	3242844	3243564	PF14_0760	hypothetical protein	2.018

chr14	3254027	3256489	PF14_0761	fatty acyl CoA synthetase 1	3.634
chr14	3260680	3261662	PF14_0763	hypothetical protein	1.918
chr14	3266071	3267312	PF14_0766	rifin	4.830
chr14	3269429	3270431	PF14_0767	stevor putative	5.216
chr14	3272836	3273783	PF14_0768	rifin	5.057
chr14	3276165	3277436	PF14_0769	rifin	4.876
chr14	3279435	3280597	PF14_0770	rifin	4.978
chr14	3282685	3283687	PF14_0771	stevor putative	5.119
chr14	3285835	3286938	PF14_0772	rifin	5.013
chr14	3290888	3291436	PF14_0773	erythrocyte membrane protein 1 %28PfEMP1%29 trunc	4.543
chr2	25232	31168	PFB0010w	erythrocyte membrane protein 1 %28PfEMP1%29	4.622
chr2	33030	34259	PFB0015c	rifin	3.785
chr2	35927	37249	PFB0020c	erythrocyte membrane protein 1 %28PfEMP1%29 trunc	5.151
chr2	38287	39303	PFB0025c	stevor putative	5.294
chr2	41515	42858	PFB0030c	rifin	4.769
chr2	45286	46800	PFB0035c	rifin	4.484
chr2	48923	50147	PFB0040c	rifin	5.259
chr2	51842	53124	PFB0045c	erythrocyte membrane protein 1 %28PfEMP1%29 trunc	5.380
chr2	54418	54936	PFB0050c	stevor isoform gam beta	5.595
chr2	57344	58421	PFB0055c	rifin	5.155
chr2	60107	60319	PFB0056c	hypothetical protein	4.208
chr2	63157	64376	PFB0060w	rifin	5.193
chr2	66550	67545	PFB0065w	stevor putative	5.260
chr2	69088	69771	PFB0070w	hypothetical protein	4.608
chr2	73441	74396	PFB0075c	hypothetical protein	0.934
chr2	77251	78808	PFB0080c	hypothetical protein	0.252
chr2	81291	84165	PFB0085c	DnaJ protein putative	0.213
chr2	86832	88633	PFB0090c	DnaJ protein putative	-0.069
chr2	91318	98838	PFB0095c	erythrocyte membrane protein 3	0.112
chr2	103385	105796	PFB0100c	knob associated histidine-rich protein	-0.271
chr2	109564	110580	PFB0105c	hypothetical protein	0.111
chr2	112547	113682	PFB0106c	hypothetical protein	0.045
chr2	117225	118235	PFB0110w	hypothetical protein	0.360
chr2	120524	124102	PFB0115w	hypothetical protein	0.185
chr2	127994	128314	PFB0120w	early transcribed membrane protein 2 ETRAMP2	-1.045

chr2	129342	133563	PFB0125c	hypothetical protein	0.095
chr2	135523	137139	PFB0130w	polyprenyl synthetase putative	-0.072
chr2	137557	138389	PFB0135w	conserved Plasmodium protein unknown function	-0.096
chr2	139955	141192	PFB0140w	hypothetical protein	0.338
chr2	141625	147564	PFB0145c	hypothetical protein	0.279
chr2	149524	159660	PFB0150c	protein kinase putative	0.058
chr2	158137	159660	PFB0151c	conserved Plasmodium protein unknown function	0.084
chr2	160514	161242	PFB0160w	ERCC1 nucleotide excision repair protein putative	0.101
chr2	162193	163253	PFB0161c	hypothetical protein conserved	0.130
chr2	166144	168051	PFB0170w	hypothetical protein conserved	-0.014
chr2	168682	170136	PFB0175c	Mak16 protein putative	-0.150
chr2	171563	174900	PFB0177c	hypothetical protein	-0.213
chr2	176567	178300	PFB0180w	5%27-3%27 exonuclease N-terminal resolvase-like dom	-0.121
chr2	178955	181526	PFB0185w	hypothetical protein conserved	-0.081
chr2	182228	189115	PFB0190c	hypothetical protein	0.139
chr2	192818	194199	PFB0194w	hypothetical protein conserved	-0.237
chr2	194826	196916	PFB0195c	hypothetical protein	0.027
chr2	197219	198043	PFB0196c	dynein light chain putative	0.203
chr2	198353	199570	PFB0200c	aspartate aminotransferase putative	-0.224
chr2	200482	204027	PFB0205c	5%27-3%27 exonuclease putative	-0.139
chr2	205889	207403	PFB0210c	hexose transporter PfHT1	-0.821
chr2	209583	210833	PFB0215c	3%27-5%27 exonuclease putative	-0.144
chr2	211348	212627	PFB0220w	UbiE-like methlytransferase putative	-0.208
chr2	212982	214508	PFB0225c	hypothetical protein	0.055
chr2	214838	218472	PFB0230c	hypothetical protein	0.035
chr2	214838	217348	PFB0227c	conserved Plasmodium protein unknown function	-0.034
chr2	219690	222256	PFB0235w	hypothetical protein	-0.078
chr2	223478	224851	PFB0240w	hypothetical protein conserved	0.098
chr2	225562	226245	PFB0245c	DNA-directed RNA polymerase II 16 kDa subunit putativ	-0.381
chr2	228025	229932	PFB0250w	hypothetical protein	-0.081
chr2	231141	232529	PFB0255w	hypothetical protein conserved	0.513
chr2	232949	233476	PFB0257c	hypothetical protein conserved	0.030
chr2	235237	238816	PFB0260w	proteasome 26S regulatory subunit putative	-0.173
chr2	239246	243796	PFB0265c	DNA repair endonuclease putative	0.044
chr2	245292	246699	PFB0270w	hypothetical protein conserved	0.008

chr2	249030	250727	PFB0275w	metabolite%2Fdrug transporter putative	-0.771
chr2	252394	254818	PFB0279w	hypothetical protein	-0.106
chr2	254824	262443	PFB0280w	hypothetical protein	-0.064
chr2	263037	267347	PFB0285c	hypothetical protein	-0.141
chr2	270078	270619	PFB0290c	transcription factor putative	0.379
chr2	271594	273009	PFB0295w	adenylosuccinate lyase putative	-0.148
chr2	273689	274507	PFB0300c	merozoite surface protein 2 precursor	-0.152
chr2	275540	276494	PFB0305c	merozoite surface protein 5	-0.048
chr2	277494	278456	PFB0310c	merozoite surface protein 4	-0.468
chr2	281907	287045	PFB0315w	41 kDa antigen	-0.162
chr2	288183	288665	PFB0320c	hypothetical protein conserved	0.508
chr2	290168	292703	PFB0325c	cysteine protease putative	-0.367
chr2	294273	297616	PFB0330c	cysteine protease putative	-0.194
chr2	298897	301793	PFB0335c	cysteine protease putative	-0.394
chr2	303593	307027	PFB0340c	cysteine protease putative	-0.121
chr2	308847	312155	PFB0345c	cysteine protease putative	-0.259
chr2	313449	316741	PFB0350c	cysteine protease putative	-0.152
chr2	317596	321163	PFB0355c	cysteine protease putative	-0.274
chr2	322338	325723	PFB0360c	cysteine protease putative	-0.179
chr2	327932	335203	PFB0365w	hypothetical protein conserved	-0.018
chr2	335679	336581	PFB0370c	RNA-binding protein putative	-0.183
chr2	337558	342966	PFB0375w	hypothetical protein	0.278
chr2	343308	349340	PFB0380c	hypothetical protein	0.188
chr2	352836	353628	PFB0385w	acyl carrier protein putative	-0.241
chr2	354621	356117	PFB0390w	ribosome releasing factor putative	-0.145
chr2	356501	358014	PFB0391c	hypothetical protein	0.220
chr2	359142	359849	PFB0395w	hypothetical protein	0.323
chr2	360828	368354	PFB0400w	Pfs45-48 related protein putative	0.572
chr2	370438	379845	PFB0405w	transmission-blocking target antigen s230 precursor	1.811
chr2	381130	383445	PFB0410c	phospholipase putative	-0.599
chr2	384865	386580	PFB0415c	hypothetical protein	-0.225
chr2	387273	388151	PFB0420w	2C-methyl-D-erythritol 2 4-cyclodiphosphate synthase	0.039
chr2	388424	390611	PFB0423c	conserved protein unknown function	-0.083
chr2	388712	392789	PFB0425c	hypothetical protein	0.012
chr2	395439	398745	PFB0435c	amino acid transporter putative	-0.241

chr2	400153	402005	PFB0440c	hypothetical protein	-0.059	
chr2	404281	406355	PFB0445c	helicase putative	-0.445	
chr2	409983	410228	PFB0450w	Sec61-gamma subunit of protein translocation complex	-0.209	
chr2	412275	412859	PFB0455w	ribosomal L37ae protein putative	-0.121	
chr2	413416	421137	PFB0460c	hypothetical protein	0.012	
chr2	423063	424654	PFB0465c	monocarboxylate transporter putative	-0.469	
chr2	425689	426303	PFB0467w	hypothetical protein conserved	-0.087	
chr2	427490	431530	PFB0470w	hypothetical protein	0.389	
chr2	432041	434086	PFB0475c	hypothetical protein conserved	-0.085	
chr2	436050	436994	PFB0480w	syntaxin putative	0.181	
chr2	437755	439002	PFB0485c	hypothetical protein	0.383	
chr2	442080	442934	PFB0490c	hypothetical protein	0.074	2cenL
chr2	451751	455116	PFB0495w	hypothetical protein	0.615	2cenR
chr2	455579	456286	PFB0500c	Rab5a GTPase	0.290	
chr2	457185	458585	PFB0501c	hypothetical protein	-0.055	
chr2	459072	461518	PFB0505c	beta-ketoacyl-acyl carrier protein synthase III precursor	-0.054	
chr2	463163	466900	PFB0510w	hypothetical protein	0.114	
chr2	467847	468476	PFB0515w	hypothetical protein	0.039	
chr2	469790	473491	PFB0520w	protein kinase putative	0.090	
chr2	475242	477074	PFB0525w	asparagine -- tRNA ligase putative	-0.356	
chr2	477432	479108	PFB0530c	hypothetical protein	0.258	
chr2	480187	481432	PFB0535w	GDP-fructose%3AGMP antiporter putative	-0.151	
chr2	483436	488970	PFB0540w	hypothetical protein	-0.226	
chr2	489904	490818	PFB0545c	ribosomal protein L7%2FL12 putative	0.134	
chr2	492616	494306	PFB0550w	peptide chain release factor subunit 1 putative	-0.300	
chr2	495166	507854	PFB0555c	hypothetical protein	0.196	
chr2	509525	521746	PFB0560w	hypothetical protein	0.087	
chr2	522931	523999	PFB0570w	SPATR-like protein putative	-0.341	
chr2	524700	526043	PFB0575c	hypothetical protein	-0.060	
chr2	528249	531923	PFB0580w	MATE antiporter putative	-0.187	
chr2	533253	534350	PFB0585w	Leu%2FPhe-tRNA protein transferase putative	-0.314	
chr2	535646	536146	PFB0590w	hypothetical protein	-0.108	
chr2	537789	539420	PFB0595w	heat shock 40 kDa protein putative	-0.245	
chr2	540409	540969	PFB0600c	hypothetical protein	0.268	
chr2	541811	543807	PFB0605w	Ser%2FThr protein kinase putative	0.182	

chr2	544008	545408	PFB0610c	hypothetical protein	0.111
chr2	546482	555294	PFB0615c	hypothetical protein	0.193
chr2	557048	557696	PFB0620w	hypothetical protein	-0.279
chr2	558585	560594	PFB0625w	hypothetical protein	0.121
chr2	561122	564622	PFB0630c	hypothetical protein	0.118
chr2	566320	567894	PFB0635w	T-complex protein 1 putative	-0.320
chr2	568695	573315	PFB0640c	COPII-coated vesicle component putative	-0.480
chr2	575155	576054	PFB0645c	Ribosomal protein L13 putative	-0.536
chr2	578662	586164	PFB0650w	hypothetical protein	0.599
chr2	586765	589051	PFB0655c	hypothetical protein	-0.034
chr2	590501	591421	PFB0660w	hypothetical protein	0.205
chr2	594196	599340	PFB0665w	Ser%2FThr protein kinase putative	-0.266
chr2	599829	601712	PFB0670c	hypothetical protein	-0.082
chr2	605045	609325	PFB0675w	hypothetical protein	-0.035
chr2	610087	615372	PFB0680w	hypothetical protein	0.238
chr2	616772	619429	PFB0685c	acyl-CoA synthetase PfACS9	-0.077
chr2	622378	624306	PFB0687c	RING zinc finger protein putative	-0.046
chr2	626976	627710	PFB0690w	hypothetical protein	0.000
chr2	628642	631308	PFB0695c	acyl-CoA synthetase	0.194
chr2	634589	637507	PFB0700c	hypothetical protein	-0.154
chr2	640400	642694	PFB0705w	hypothetical protein	-0.044
chr2	643106	644317	PFB0710c	hypothetical protein	0.132
chr2	645929	650761	PFB0715w	DNA-directed RNA polymerase II second largest subunit	-0.634
chr2	650663	653362	PFB0720c	hypothetical protein conserved	-0.182
chr2	655997	656323	PFB0725c	hypothetical protein	-0.140
chr2	658636	664629	PFB0730w	DNA helicase putative	-0.011
chr2	664984	669841	PFB0735c	hypothetical protein	0.262
chr2	664984	668532	PFB0732c	conserved Plasmodium protein unknown function	0.293
chr2	670413	674557	PFB0745w	hypothetical protein	0.129
chr2	675430	677598	PFB0750w	vacuolar protein-sorting protein VPS45 putative	0.041
chr2	678486	683702	PFB0755w	hypothetical protein	-0.075
chr2	686484	688544	PFB0760w	MtN3-like protein putative	-0.311
chr2	690310	695782	PFB0765w	hypothetical protein	0.037
chr2	696192	699314	PFB0770c	hypothetical protein	-0.165
chr2	701244	702893	PFB0775w	hypothetical protein	-0.058

chr2	705177	706340	PFB0790c	hypothetical protein	0.039
chr2	707737	709392	PFB0795w	ATP synthase F1 alpha subunit putative	-0.516
chr2	709798	715251	PFB0800c	hypothetical protein	0.131
chr2	715797	716548	PFB0805c	clathrin coat assembly protein putative	0.135
chr2	717748	718280	PFB0810w	hypothetical protein	-0.159
chr2	720436	722660	PFB0815w	Pf Calcium-dependent protein kinase 1	-0.345
chr2	723746	729931	PFB0820c	hypothetical protein	0.110
chr2	731119	732311	PFB0826c	hypothetical protein	0.296
chr2	734271	734594	PFB0830w	Ribosomal protein S26e putative	-0.295
chr2	735437	736996	PFB0835c	hypothetical protein	-0.136
chr2	738859	739851	PFB0840w	replication factor C subunit 2	-0.377
chr2	741013	742458	PFB0845w	hypothetical protein	0.074
chr2	743288	749364	PFB0855c	hypothetical protein	0.141
chr2	743288	745711	PFB0850c	conserved Plasmodium protein unknown function	-0.054
chr2	750048	751736	PFB0860c	RNA helicase putative	-0.540
chr2	752965	753438	PFB0865w	small nuclear ribonucleoprotein putative	-0.438
chr2	755417	762559	PFB0870w	hypothetical protein	0.074
chr2	763490	764950	PFB0875c	Chromatin-binding protein putative	-0.095
chr2	767149	767940	PFB0877c	hypothetical protein	0.772
chr2	769760	771040	PFB0880w	hypothetical protein conserved	-0.055
chr2	772314	772688	PFB0885w	40S ribosomal protein S30 putative	-0.195
chr2	773333	773752	PFB0886c	hypothetical protein	0.062
chr2	775637	776034	PFB0888w	hypothetical protein	0.174
chr2	776826	778063	PFB0890c	pseudouridine synthetase putative	0.340
chr2	779416	782130	PFB0895c	replication factor C subunit 1 putative	-0.064
chr2	783721	784826	PFB0900c	hypothetical protein	0.000
chr2	786868	787725	PFB0905c	hypothetical protein	0.002
chr2	791462	792379	PFB0910w	hypothetical protein	0.176
chr2	796750	801584	PFB0915w	liver stage antigen 3	-0.033
chr2	804518	807739	PFB0920w	DnaJ protein putative	-0.137
chr2	809509	810296	PFB0921c	hypothetical protein	0.242
chr2	813825	814652	PFB0923c	hypothetical protein	0.149
chr2	820427	822539	PFB0925w	DnaJ protein putative	0.195
chr2	823823	824862	PFB0926c	hypothetical protein	0.502
chr2	831187	832003	PFB0930w	hypothetical protein	-0.128

chr2	833803	834686	PFB0932w	hypothetical protein	-0.001
chr2	838841	844114	PFB0935w	cytoadherence linked asexual protein 2	2.174
chr2	844860	845837	PFB0946c	hypothetical protein	3.602
chr2	847487	849117	PFB0950w	hypothetical protein	3.731
chr2	851422	851960	PFB0951w	hypothetical protein pseudogene	1.452
chr2	855043	855736	PFB0953w	hypothetical protein	0.767
chr2	857433	858043	PFB0954c	hypothetical protein conserved in P falciparum	2.642
chr2	858455	859069	PFB1070w	hypothetical protein	2.724
chr2	860353	861502	PFB0955w	stevor degenerate putative	4.997
chr2	863384	863840	PFB0960c	Plasmdoium falciparum Maurer%27s Cleft 2 transmembr	4.425
chr2	865914	866324	PFB0965c	hypothetical protein	3.583
chr2	869506	871122	PFB0970c	hypothetical protein	1.242
chr2	873129	873317	PFB0972w	hypothetical protein	1.653
chr2	873915	874109	PFB0973c	hypothetical protein	3.188
chr2	874129	874323	PFB0974c	erythrocyte membrane protein 1 %28PfEMP1%29 trunca	4.623
chr2	878466	879249	PFB0985c	Plasmodium falciparum Maurer%27s Cleft 2 transmembr	4.814
chr2	886915	887802	PFB1000w	rifin	5.239
chr2	889660	890745	PFB1005w	rifin	5.373
chr2	892856	894206	PFB1010w	rifin	4.848
chr2	896226	897420	PFB1015w	rifin	4.937
chr2	899451	900445	PFB1020w	stevor putative	4.968
chr2	902500	902853	PFB1030w	hypothetical protein	5.255
chr2	904346	905775	PFB1035w	rifin	4.807
chr2	907678	908861	PFB1040w	rifin	5.051
chr2	909350	911054	PFB1045w	erythrocyte membrane protein 1 %28PfEMP1%29 trunca	4.814
chr2	913244	914457	PFB1050w	rifin	3.924
chr2	916352	923648	PFB1055c	erythrocyte membrane protein 1 %28PfEMP1%29	4.743
chr3	8394	10745	PFC0002c	hypothetical protein conserved in P. falciparum	4.563
chr3	33641	41158	PFC0005w	PfEMP1	4.901
chr3	43045	44255	PFC0010c	rifin	3.753
chr3	48956	49949	PFC0025c	stevor	5.275
chr3	52066	53260	PFC0030c	rifin	5.184
chr3	58103	59390	PFC0035w	rifin	5.035
chr3	61248	62459	PFC0040w	rifin	5.376
chr3	64604	65508	PFC0045w	rifin-like protein	4.746

chr3	66136	68595	PFC0050c	acetyl-CoA synethetase PfACS2	5.061
chr3	73172	74152	PFC0055w	hypothetical protein	4.126
chr3	74873	77071	PFC0060c	Serine%2Fthreonine protein kinase putative	2.462
chr3	78689	80193	PFC0065c	alpha%2Fbeta hydrolase protein putative	0.488
chr3	81847	82809	PFC0070c	hypothetical protein	0.267
chr3	84928	85909	PFC0075c	hypothetical protein conserved	-0.077
chr3	87793	90714	PFC0080c	hypothetical protein	0.176
chr3	93248	94587	PFC0085c	hypothetical protein conserved	0.079
chr3	101005	101961	PFC0090w	hypothetical protein conserved	-0.127
chr3	102593	103789	PFC0095c	hypothetical protein conserved	0.259
chr3	104641	106560	PFC0100c	regulatory protein putative	0.169
chr3	110750	114766	PFC0105w	serine%2Fthreonine protein kinase putative	3.226
chr3	116138	121415	PFC0110w	Cytoadherence linked asexual protein 3.1	4.766
chr3	122672	126915	PFC0115c	erythrocyte membrane protein 1 %28PfEMP1%29 pseud	5.466
chr3	132097	137339	PFC0120w	Cytoadherence linked asexual protein 3.2	2.991
chr3	138235	142332	PFC0125w	ABC transporter %28TAP family%29	0.141
chr3	143051	143735	PFC0126c	hypothetical protein conserved	0.157
chr3	144725	145984	PFC0130c	hypothetical protein conserved	0.094
chr3	148844	152822	PFC0135c	nuclear export receptor crm1 homolog	-0.043
chr3	156029	158380	PFC0140c	N-ethylmaleimide-sensitive fusion protein putative	-0.406
chr3	159454	163779	PFC0145c	hypothetical protein conserved	0.118
chr3	166194	170976	PFC0150w	hypothetical protein conserved	-0.291
chr3	171698	172333	PFC0155c	DNA-directed RNA polymerase subunit I putative	-0.442
chr3	175096	177803	PFC0160w	binding protein putative	-0.121
chr3	179407	186087	PFC0165w	hypothetical protein conserved	-0.056
chr3	186856	187395	PFC0166w	thioredoxin-like redox-active protein putative	-0.404
chr3	187921	189267	PFC0170c	dihydrolipoamide acyltransferase putative	-0.057
chr3	191106	192887	PFC0175w	IBR domain protein putative	0.127
chr3	193246	194114	PFC0176c	hypothetical protein conserved	-0.455
chr3	195218	198194	PFC0180c	membrane skeletal protein putative	-0.282
chr3	201218	202795	PFC0185w	hypothetical protein conserved	-0.299
chr3	204018	205619	PFC0190c	EH %28Eps15 homology%29 protein	-0.252
chr3	207807	208477	PFC0191c	hypothetical protein	0.087
chr3	208956	213246	PFC0195w	hypothetical protein conserved	0.105
chr3	214615	215163	PFC0200w	60S Ribosomal protein L44 putative	0.186

chr3	215791	216546	PFC0205c	PfGLP-1 1-cys-glutaredoxin-like protein-1	-0.099
chr3	217997	219190	PFC0210c	circumsporozoite %28CS%29 protein	-0.121
chr3	220611	222467	PFC0215c	hypothetical protein	0.000
chr3	223731	228071	PFC0220w	hypothetical protein conserved	0.194
chr3	228467	228979	PFC0221c	hypothetical protein conserved	0.126
chr3	230732	232008	PFC0225c	elongation factor %28EF-TS%29 putative	-0.172
chr3	233008	244944	PFC0230c	hypothetical protein conserved	0.010
chr3	247917	251336	PFC0235w	hypothetical protein conserved	-0.015
chr3	251951	255091	PFC0240c	hypothetical protein conserved	0.107
chr3	257007	257760	PFC0241w	hypothetical protein conserved	0.124
chr3	258160	269961	PFC0245c	hypothetical protein conserved	0.122
chr3	272539	274392	PFC0250c	AP endonuclease %28DNA-%28apurinic or apyrimidinic	-0.385
chr3	275631	276503	PFC0255c	ubiquitin-conjugating enzyme E2 putative	-0.276
chr3	278591	281935	PFC0260w	hypothetical protein conserved	-0.202
chr3	282254	282787	PFC0261c	hypothetical protein conserved	0.291
chr3	283581	283994	PFC0262c	hypothetical protein conserved	0.006
chr3	285312	287422	PFC0265c	hypothetical protein conserved	-0.003
chr3	289618	291223	PFC0270w	hypothetical protein conserved	0.170
chr3	291782	292769	PFC0271c	glutaredoxin putative	-0.226
chr3	295191	297152	PFC0275w	FAD-dependent glycerol-3-phosphate dehydrogenase p	-0.576
chr3	297681	298580	PFC0280c	hypothetical protein conserved	-0.621
chr3	299500	299757	PFC0281w	hypothetical protein conserved	-0.681
chr3	300991	301673	PFC0282w	hypothetical protein conserved	0.238
chr3	302782	304380	PFC0285c	T-complex protein beta subunit putative	-0.829
chr3	307326	308479	PFC0290w	40S ribosomal protein S23 putative	-0.179
chr3	309367	310016	PFC0295c	40S ribosomal protein S12 putative	-0.674
chr3	311650	312910	PFC0300c	60S ribosomal protein L7 putative	-0.292
chr3	315479	317011	PFC0305w	EB1 homolog putative	-0.210
chr3	317725	318837	PFC0310c	ATP-dependent CLP protease putative	-0.042
chr3	319608	321511	PFC0315c	hypothetical protein conserved	-0.077
chr3	322597	325058	PFC0320w	hypothetical protein conserved	-0.136
chr3	325462	331944	PFC0325c	hypothetical protein conserved	-0.221
chr3	333854	335953	PFC0330w	hypothetical protein conserved	-0.078
chr3	336703	348327	PFC0335c	hypothetical protein conserved	0.083
chr3	350224	351720	PFC0340w	DNA polymerase delta small subunit putative	-0.139

chr3	352994	358129	PFC0345w	hypothetical protein	-0.128
chr3	359017	360908	PFC0350c	T-complex protein eta subunit putative	-0.403
chr3	362334	363347	PFC0355c	hypothetical protein	-0.311
chr3	365247	365365	PFC0358w	PFC0358w	0.515
chr3	366600	367464	PFC0360w	hypothetical protein conserved	-0.070
chr3	369307	370905	PFC0365w	conserved protein putative	-0.413
chr3	372481	374112	PFC0370w	hypothetical protein conserved	-0.428
chr3	374720	375192	PFC0371w	hypothetical protein conserved	-0.382
chr3	375805	379965	PFC0375c	splicing factor putative	-0.655
chr3	381050	383554	PFC0380w	dual-specificity protein phosphatase putative	-0.231
chr3	383894	385148	PFC0381c	hypothetical protein conserved	-0.198
chr3	385812	391279	PFC0385c	serine%2Fthreonine protein kinase putative	-0.183
chr3	394323	397190	PFC0390w	hypothetical protein conserved	-0.574
chr3	399607	402114	PFC0395w	asparagine synthetase putative	-0.548
chr3	404654	404992	PFC0400w	60S Acidic ribosomal protein P2	0.257
chr3	405776	407602	PFC0405c	hypothetical protein conserved	0.146
chr3	408763	410436	PFC0410w	rat BRAIN protein-like	-0.098
chr3	410908	416347	PFC0415c	hypothetical protein conserved	0.057
chr3	417371	418189	PFC0416w	hypothetical protein conserved	-0.258
chr3	419053	421354	PFC0420w	calcium-dependent protein kinase 3	-0.457
chr3	423964	437616	PFC0425w	hypothetical protein conserved	-0.091
chr3	439394	442834	PFC0430w	hypothetical protein conserved	-0.038
chr3	444174	448058	PFC0435w	hypothetical protein conserved	-0.004
chr3	448971	455780	PFC0440c	helicase putative	-0.088
chr3	457849	458701	PFC0441c	eukaryotic translation initiation factor putative	0.018
chr3	460002	460950	PFC0445w	hypothetical protein conserved	0.141
chr3	461338	461667	PFC0450w	hypothetical protein conserved	-0.056
chr3	462761	465121	PFC0455w	synbindin-like protein putative	-0.163
chr3	465963	466794	PFC0460w	hypothetical protein conserved	-0.185
chr3	467536	469837	PFC0465c	hypothetical protein conserved	-0.042
chr3	471464	475762	PFC0470w	valine -- tRNA ligase putative	-0.017
chr3	476798	480129	PFC0475c	hypothetical protein conserved	-0.137
chr3	485480	493186	PFC0485w	protein kinase putative	-0.321
chr3	493794	494039	PFC0486c	hypothetical protein conserved	-0.787
chr3	496277	499028	PFC0490w	dolichyl-diphospho-oligosaccharide protein	-0.113

chr3	499373	502523	PFC0495w	aspartyl protease putative	0.058	
chr3	503330	505521	PFC0500w	hypothetical protein conserved	-0.324	
chr3	505966	508272	PFC0505c	hypothetical protein conserved	-0.100	
chr3	508805	509626	PFC0506w	hypothetical protein conserved	-0.268	
chr3	510427	512937	PFC0510w	zinc finger protein putative	-0.289	
chr3	513468	517178	PFC0515c	hypothetical protein conserved	-0.010	
chr3	518644	519558	PFC0520w	26S proteasome regulatory subunit S14 putative	-0.285	
chr3	520829	523114	PFC0525c	glycogen synthase kinase 3	-0.337	
chr3	525713	527452	PFC0530w	transporter	-0.368	
chr3	530916	531915	PFC0535w	60S ribosomal protein L26 putative	-0.322	
chr3	532720	533951	PFC0540w	hypothetical protein	-0.243	
chr3	534298	535481	PFC0545c	hypothetical protein conserved	0.086	
chr3	536420	539487	PFC0550w	hypothetical protein conserved	0.064	
chr3	539918	540619	PFC0555c	hypothetical protein conserved	0.232	
chr3	540732	541171	PFC0556c	hypothetical protein conserved	0.152	
chr3	542505	544859	PFC0560c	hypothetical protein conserved	0.076	
chr3	545395	548130	PFC0565w	hypothetical protein conserved	0.085	
chr3	548678	551298	PFC0570c	hypothetical protein conserved	0.076	
chr3	551712	555449	PFC0571c	hypothetical protein conserved	0.117	
chr3	556011	558631	PFC0575w	hypothetical protein conserved	0.022	
chr3	559043	562333	PFC0580c	hypothetical protein conserved	0.407	
chr3	563358	565527	PFC0581w	hypothetical protein conserved	-0.051	
chr3	565713	567729	PFC0582c	hypothetical protein conserved	0.273	
chr3	569342	575042	PFC0590c	der1-like protein putative	0.355	
chr3	576215	577141	PFC0595c	serine%2Fthreonine protein phosphatase putative	-0.748	
chr3	580902	581844	PFC0600w	hypothetical protein conserved	0.007	
chr3	583234	583977	PFC0605w	hypothetical protein conserved	0.005	
chr3	585679	589224	PFC0610c	hypothetical protein	0.552	3cenL
chr3	601273	604606	PFC0615w	hypothetical protein conserved	0.470	3cenR
chr3	605558	607393	PFC0625w	hypothetical protein conserved	0.417	
chr3	608009	608689	PFC0630w	hypothetical protein conserved	0.491	
chr3	609414	610097	PFC0635c	translation initiation factor E4 putative	-0.132	
chr3	613146	619490	PFC0640w	CSP and TRAP-related protein %28CTRP%29	0.963	
chr3	620120	621948	PFC0645w	hypothetical protein conserved	0.689	
chr3	622829	626852	PFC0650w	hypothetical protein conserved	0.417	

chr3	627285	628055	PFC0670c	hypothetical protein conserved	0.191
chr3	629520	630218	PFC0675c	hypothetical protein conserved	-0.037
chr3	632260	634136	PFC0680w	hypothetical protein conserved	-0.504
chr3	634957	637980	PFC0685w	hypothetical protein conserved	0.214
chr3	638156	639253	PFC0690c	hypothetical protein conserved	0.045
chr3	640860	641942	PFC0695w	hypothetical protein conserved	-0.024
chr3	642595	643518	PFC0700c	hypothetical protein conserved	-0.143
chr3	645309	646205	PFC0701w	hypothetical protein conserved	-0.287
chr3	646523	653921	PFC0705c	hypothetical protein conserved	0.348
chr3	656694	658317	PFC0710w	inorganic pyrophosphatase putative	-0.180
chr3	658800	661826	PFC0715c	hypothetical protein conserved	-0.027
chr3	663680	665065	PFC0720w	hypothetical protein conserved	-0.291
chr3	665976	667547	PFC0725c	formate-nitrate transporter putative	-0.251
chr3	671131	672309	PFC0730w	conserved protein putative	-0.259
chr3	674515	675147	PFC0735w	40S ribosomal protein S15A putative	-0.483
chr3	675907	677950	PFC0740c	binding protein putative	-0.133
chr3	679468	680358	PFC0745c	proteasome component C8 putative	-0.581
chr3	682955	686155	PFC0750w	hypothetical protein conserved	-0.132
chr3	686827	691488	PFC0755c	protein kinase predicted	-0.104
chr3	696215	706399	PFC0760c	hypothetical protein conserved	0.204
chr3	708925	710754	PFC0765c	hypothetical protein conserved	-0.077
chr3	713195	718503	PFC0770c	kinesin-related protein putative	0.084
chr3	721686	722801	PFC0775w	40S ribosomal protein S11 putative	0.490
chr3	724930	734747	PFC0780w	hypothetical protein conserved	-0.121
chr3	734876	735553	PFC0785c	proteasome regulatory protein putative	-0.402
chr3	735893	738404	PFC0790w	hypothetical protein conserved	-0.055
chr3	738862	739470	PFC0795w	hypothetical protein conserved	-0.123
chr3	740600	741724	PFC0800w	band 7-related protein	-0.392
chr3	745136	752509	PFC0805w	DNA-directed RNA polymerase II putative	-0.456
chr3	753162	756963	PFC0810c	hypothetical protein conserved	0.069
chr3	758364	762110	PFC0815c	hypothetical protein conserved	0.125
chr3	764486	779431	PFC0820w	hypothetical protein conserved	-0.017
chr3	779783	782836	PFC0825c	cleavage and polyadenylation specificity factor protein p	-0.282
chr3	785124	785483	PFC0830w	trophozoite stage antigen	-0.417
chr3	786529	787979	PFC0831w	triosephophate isomerase putative	-0.099

chr3	788511	790152 PFC0835c	hypothetical protein conserved	0.039
chr3	792248	797842 PFC0840w	P-type ATPase putative	-0.487
chr3	798931	799254 PFC0845c	ubiquitin--protein ligase putative	-0.575
chr3	800263	802983 PFC0850c	hypothetical protein conserved	0.210
chr3	803948	804530 PFC0855w	ubiquitin-conjugating enzyme putative	-0.434
chr3	807240	810842 PFC0860w	kinesin putative	-0.238
chr3	812239	813525 PFC0865w	RNA-binding protein putative	-0.298
chr3	814583	815365 PFC0870w	elongation factor 1 %28EF-1%29 putative	-0.175
chr3	817378	827472 PFC0875w	ABC transporter putative	-0.019
chr3	827746	829179 PFC0880c	hypothetical protein conserved	0.237
chr3	830503	831447 PFC0885c	hypothetical protein conserved	-0.281
chr3	834171	835251 PFC0886w	hypothetical protein conserved	0.158
chr3	837032	838428 PFC0890w	Sec22 subunit putative	0.047
chr3	838983	840960 PFC0895w	hypothetical protein conserved	0.066
chr3	842383	843990 PFC0900w	T-complex protein 1 epsilon subunit putative	-0.268
chr3	844917	854174 PFC0905c	hypothetical protein conserved	2.313
chr3	857483	858772 PFC0910w	hypothetical protein conserved	-0.104
chr3	859411	860502 PFC0911c	hypothetical protein conserved	0.342
chr3	861684	863090 PFC0912w	hypothetical protein conserved	-0.115
chr3	867487	869012 PFC0915w	ATP-dependent RNA helicase putative	-0.442
chr3	871883	872965 PFC0920w	histone H2A variant putative	-0.019
chr3	874405	876181 PFC0925w	hypothetical protein conserved	0.183
chr3	876629	882163 PFC0930c	hypothetical protein conserved	0.158
chr3	883632	885646 PFC0935c	N-acetylglucosamine-1-phosphate transferase putative	-0.150
chr3	886684	889104 PFC0940c	hypothetical protein conserved	0.203
chr3	891430	892781 PFC0945w	protein kinase putative	-0.271
chr3	893215	896439 PFC0950c	peptidase putative	-0.137
chr3	897526	900038 PFC0955w	ATP-dependent RNA helicase	-0.052
chr3	900433	906787 PFC0960c	hypothetical protein conserved	0.160
chr3	909587	917707 PFC0965w	hypothetical protein conserved	-0.078
chr3	919645	924613 PFC0970w	hypothetical protein conserved	-0.034
chr3	925315	925830 PFC0975c	PFCYP19 cyclophilin peptidyl-prolyl cis-trans isomerase	-0.556
chr3	927311	929450 PFC0980c	mRNA capping enzyme	-0.220
chr3	929859	933677 PFC0990c	hypothetical protein conserved	0.225
chr3	934883	936844 PFC0995c	diacylglycerol O-acyltransferase putative	-0.335

chr3	939216	942070	PFC1000w	hypothetical protein conserved	-0.055
chr3	942209	943423	PFC1005c	HesB-like domain protein	0.185
chr3	945046	949365	PFC1010w	hypothetical protein conserved	0.117
chr3	949636	951938	PFC1011c	hypothetical protein conserved	0.134
chr3	952113	959039	PFC1015c	hypothetical protein conserved	0.197
chr3	960773	961875	PFC1016w	hypothetical protein conserved	-0.072
chr3	962225	963174	PFC1020c	40S ribosomal protein S3A putative	-0.100
chr3	965347	965874	PFC1025w	F49C12.11-like protein	0.103
chr3	967838	972466	PFC1030w	hypothetical protein conserved	0.061
chr3	973540	974865	PFC1035w	hypothetical protein conserved	0.031
chr3	975640	976941	PFC1040w	hypothetical protein conserved	0.296
chr3	977376	980636	PFC1045c	hypothetical protein conserved	-0.020
chr3	982935	984280	PFC1050w	hypothetical protein conserved	-0.050
chr3	985301	987552	PFC1055w	hypothetical protein conserved	0.239
chr3	988214	990295	PFC1060c	hypothetical protein conserved	0.121
chr3	995319	998810	PFC1065w	hypothetical protein conserved	0.421
chr3	999860	1000734	PFC1070c	VARC pseudogene	4.112
chr3	1004083	1004872	PFC1080c	Plasmodium falciparum Maurer%27s Cleft 2 transmembr	4.749
chr3	1012465	1013612	PFC1095w	rifin %283D7-rifT3-5%29	5.078
chr3	1015544	1016643	PFC1100w	rifin	5.195
chr3	1018668	1019678	PFC1105w	stevor %283D7-stevorT3-2%29	5.495
chr3	1024241	1025599	PFC1115w	rifin %283D7-rifT3-7%29	3.951
chr3	1027492	1034924	PFC1120c	var %283D7-varT3-2%29	4.801
chr3	1046861	1050978	PFC1125w	hypothetical protein conserved in P. falciparum	3.925
chr4	35153	44124	PFD0005w	erythrocyte membrane protein 1 %28PfEMP1%29	4.881
chr4	52002	63307	PFD0020c	erythrocyte membrane protein 1 %28PfEMP1%29	5.029
chr4	66083	67386	PFD0025w	rifin	4.495
chr4	69551	70923	PFD0030c	rifin	4.725
chr4	73048	74020	PFD0035c	stevor	5.194
chr4	76408	77560	PFD0040c	rifin	5.022
chr4	79715	80787	PFD0045c	rifin	5.000
chr4	85640	86898	PFD0050w	rifin	5.231
chr4	88694	90022	PFD0055w	rifin	5.009
chr4	91844	93055	PFD0060w	rifin	5.268
chr4	95600	96631	PFD0065w	stevor pseudogene	4.308

chr4	99023	100256	PFD0070c	rifin	1.080
chr4	107134	108560	PFD0075w	hypothetical protein conserved in P. falciparum	1.982
chr4	110192	112053	PFD0080c	hypothetical protein conserved in P.falciparum	0.605
chr4	114830	117745	PFD0085c	acyl-Coa synthetase PfACS6	3.087
chr4	121052	122509	PFD0090c	hypothetical protein conserved in P.falciparum	1.596
chr4	130044	131920	PFD0095c	hypothetical protein conserved in P.falciparum	2.211
chr4	134169	140925	PFD0100c	surface-associated interspersed gene 4.1 %28SURFIN4	2.756
chr4	144098	153112	PFD0110w	normocyte-binding protein 1 pseudogene	1.606
chr4	153950	154840	PFD0115c	hypothetical protein	0.449
chr4	162335	163442	PFD0120w	RIF pseudogene RIFIN pseudogene	5.181
chr4	165737	166772	PFD0125c	stevor	5.283
chr4	168969	169472	PFD0134c	RIF pseudogene RIFIN pseudogene	5.116
chr4	169474	170188	PFD0135c	rifin truncated	4.794
chr4	173451	178412	PFD0135w	erythrocyte membrane protein 1 %28PfEMP1%29 pseud	4.666
chr4	178964	179372	PFD0144c	hypothetical protein	0.656
chr4	181831	183711	PFD0145c	hypothetical protein conserved	0.264
chr4	184977	186659	PFD0150w	hypothetical protein	0.277
chr4	186889	188010	PFD0155c	hypothetical protein	0.623
chr4	189329	201171	PFD0160w	hypothetical protein conserved	0.194
chr4	202002	203204	PFD0165w	ubiquitin-specific protease putative	-0.191
chr4	204305	206218	PFD0170c	hypothetical protein conserved	-0.059
chr4	207242	207667	PFD0175c	hypothetical protein	0.120
chr4	212692	215858	PFD0180c	CGI-201 protein short form	-0.128
chr4	218114	220318	PFD0185c	peptidase	-0.137
chr4	225550	229301	PFD0190w	SET domain putative	-0.174
chr4	230174	230594	PFD0195c	hypothetical protein conserved	0.517
chr4	231242	238197	PFD0200c	hypothetical protein conserved	0.917
chr4	239059	239713	PFD0205c	hypothetical protein conserved	-0.128
chr4	240478	243386	PFD0207c	hypothetical protein conserved	0.468
chr4	244820	245959	PFD0210c	pbs36 homologue	-0.306
chr4	247371	248807	PFD0215c	pf52 protein	-0.019
chr4	254195	266611	PFD0225w	hypothetical protein conserved	0.051
chr4	266919	269883	PFD0230c	protease putative	-0.044
chr4	270770	272635	PFD0235c	hypothetical protein conserved	-0.018
chr4	273709	274845	PFD0240c	pfs45-48 related protein putative	-0.363

chr4	275425	277599	PFD0245c	ATP-dependent RNA helicae-like protein putative	-0.082
chr4	278720	283289	PFD0250c	Sec24-like protein putative	-0.556
chr4	285541	288452	PFD0255w	ag-1 blood stage membrane protein homologue	0.007
chr4	288872	294766	PFD0260c	sequestrin	-0.106
chr4	297721	307446	PFD0265w	pre-mRNA splicing factor putative	-0.703
chr4	307729	308737	PFD0270c	hypothetical protein conserved	-0.028
chr4	309799	311274	PFD0275w	secy-independent transporter protein putative	0.160
chr4	311319	311815	PFD0280w	hypothetical protein	-0.326
chr4	312218	319465	PFD0285c	lysine decarboxylase putative	-0.283
chr4	322034	322342	PFD0290w	hypothetical protein conserved	-0.057
chr4	323012	325650	PFD0295c	apical sushi protein ASP	-0.058
chr4	327491	329017	PFD0300w	hypothetical protein conserved	-0.078
chr4	329739	331698	PFD0305c	vacuolar ATP synthase subunit b	-0.327
chr4	335693	336166	PFD0310w	sexual stage-specific protein precursor	-0.789
chr4	336415	336857	PFD0315c	hypothetical protein	-0.979
chr4	338602	340333	PFD0311w	hydroxyacyl glutathione hydrolase putative	-0.229
chr4	340759	350972	PFD0320c	hypothetical protein conserved	0.118
chr4	353226	353645	PFD0325w	hypothetical protein conserved	0.032
chr4	354376	358348	PFD0330w	hypothetical protein conserved	-0.060
chr4	358450	359906	PFD0335c	hypothetical protein conserved	-0.390
chr4	360578	367091	PFD0340c	hypothetical protein conserved	-0.031
chr4	367471	368688	PFD0345c	hypothetical protein conserved	0.256
chr4	369311	370366	PFD0350w	hypothetical protein conserved	0.153
chr4	370523	371857	PFD0355c	Peptidyl-tRNA hydrolase PTH2 putative	0.043
chr4	373303	374265	PFD0360w	hypothetical protein conserved	-0.009
chr4	374769	375077	PFD0365c	hypothetical protein	-0.281
chr4	376809	378194	PFD0367w	hypothetical protein conserved	0.011
chr4	379964	383602	PFD0375w	hypothetical protein conserved	-0.206
chr4	384406	390014	PFD0380c	hypothetical protein conserved	-0.045
chr4	391851	397733	PFD0385w	hypothetical protein conserved	-0.136
chr4	398274	400277	PFD0385c	AAA family ATPase putative	-0.126
chr4	400991	401506	PFD0395c	hypothetical protein conserved	0.339
chr4	404254	407589	PFD0400w	hypothetical protein conserved	-0.001
chr4	408361	410229	PFD0403w	hypothetical protein conserved	0.051
chr4	410521	412261	PFD0405c	hypothetical protein conserved	-0.285

chr4	413132	414806	PFD0415c	hypothetical protein conserved	0.002
chr4	415576	417594	PFD0420c	flap exonuclease putative	-0.209
chr4	420300	423254	PFD0425w	hypothetical protein conserved	-0.306
chr4	424269	427721	PFD0430c	MAC%2FPerforin putative	-0.071
chr4	428908	429855	PFD0435c	hypothetical protein conserved	-0.046
chr4	431197	434091	PFD0440w	peptidase M22 family putative	-0.064
chr4	434561	442264	PFD0445c	hypothetical protein conserved	0.223
chr4	443114	444878	PFD0450c	pre-mrna splicing factor putative	-0.031
chr4	445784	447232	PFD0455w	ribosomal processing protein putative	-0.680
chr4	447865	451139	PFD0460c	hypothetical protein conserved	0.137
chr4	452897	454915	PFD0462w	DNAJ protein	-0.346
chr4	456086	457099	PFD0465c	hypothetical protein conserved	-0.245
chr4	457297	457530	PFD0466c	hypothetical protein	-0.036
chr4	459031	462468	PFD0470c	replication factor a protein putative	-0.227
chr4	463819	464917	PFD0480w	hypothetical protein conserved	0.128
chr4	466607	468334	PFD0485w	hypothetical protein conserved	-0.177
chr4	468888	470854	PFD0487c	actin-like protein putative	-0.190
chr4	471629	474236	PFD0495c	hypothetical protein conserved	0.207
chr4	477425	480493	PFD0505c	protein phosphatase 2C	-0.252
chr4	481439	482455	PFD0515w	exosome complex exonuclease rrp4 putative	-0.486
chr4	482841	483482	PFD0520c	hypothetical protein conserved	-0.338
chr4	485925	487365	PFD0525w	hypothetical protein conserved	-0.248
chr4	488074	490788	PFD0530c	Hypothetical protein conserved	-0.178
chr4	492729	496625	PFD0535w	hypothetical protein conserved	-0.100
chr4	497339	500272	PFD0540c	hypothetical protein conserved	0.216
chr4	506065	510777	PFD0545w	hypothetical protein conserved	-0.249
chr4	511375	511992	PFD0550c	hypothetical protein conserved	-0.121
chr4	512830	516567	PFD0555c	hypothetical protein conserved	0.261
chr4	517277	519445	PFD0560w	hypothetical protein conserved	-0.068
chr4	520051	523965	PFD0565c	RNA helicase putative	0.129
chr4	525582	525998	PFD0580c	hypothetical protein conserved	0.476
chr4	526729	532515	PFD0585c	hypothetical protein conserved	0.180
chr4	535089	541382	PFD0590c	DNA polymerase alpha	0.185
chr4	543671	546007	PFD0595w	hypothetical protein conserved	-0.010
chr4	547085	548095	PFD0600c	ribosomal protein putative	0.082

chr4	548708	549706	PFD0605c	hypothetical protein conserved	0.217	
chr4	550522	552144	PFD0610w	hypothetical protein conserved	0.572	
chr4	552884	560707	PFD0615c	erythrocyte membrane protein 1 %28PfEMP1%29	4.703	
chr4	566284	567535	PFD0620c	PFD0620c	4.903	
chr4	566507	567535	PFD0595c	rifin truncated	4.903	
chr4	568564	576239	PFD0625c	erythrocyte membrane protein 1 %28PfEMP1%29	4.827	
chr4	583793	591651	PFD0630c	erythrocyte membrane protein 1 %28PfEMP1%29	5.266	
chr4	598932	606832	PFD0635c	erythrocyte membrane protein 1 %28PfEMP1%29	5.173	
chr4	610149	611439	PFD0640c	rifin	5.278	
chr4	616138	617416	PFD0645w	rifin	4.793	
chr4	618860	619174	PFD0650w	hypothetical protein	5.658	
chr4	619781	621052	PFD0655w	erythrocyte membrane protein 1 %28PfEMP1%29 pseud	3.827	
chr4	622339	623226	PFD0660w	phosphoglycerate mutase putative	0.488	
chr4	623897	625333	PFD0665c	26s proteasome aaa-ATPase subunit Rpt3 putative	0.053	
chr4	626769	627785	PFD0670c	lysine decarboxylase-like protein putative	0.085	
chr4	629077	629919	PFD0675w	ribosomal protein l10 putative	0.269	
chr4	630151	633209	PFD0655c	ubiquitin carboxyl-terminal hydrolase a putative	0.294	
chr4	633874	640107	PFD0685c	chromosome associated protein putative	0.412	
chr4	641010	645886	PFD0690c	hypothetical protein conserved	0.544	4cenL
chr4	654372	655371	PFD0692c	hypothetical protein conserved	0.532	4cenR
chr4	656876	657714	PFD0669c	hypothetical protein conserved	0.063	
chr4	659333	660537	PFD0695w	hypothetical protein conserved	0.173	
chr4	662008	664297	PFD0700c	RNA binding protein putative	-0.096	
chr4	665603	666521	PFD0705c	hypothetical protein conserved	-0.174	
chr4	667607	669502	PFD0710w	GTP-binding protein putative	-0.072	
chr4	670399	670995	PFD0715c	hypothetical protein conserved	-0.198	
chr4	672956	675130	PFD0720w	hypothetical protein conserved	0.140	
chr4	675561	676700	PFD0725c	arsenical pump-driving ATPase putative	-0.104	
chr4	677866	679107	PFD0730w	hypothetical protein conserved	0.378	
chr4	679483	686121	PFD0735c	hypothetical protein conserved	0.174	
chr4	686279	686643	PFD0711c	hypothetical protein	1.352	
chr4	688793	692951	PFD0740w	Plasmodium falciparum protein kinase putative	-0.131	
chr4	693397	694023	PFD0745c	nonclathrin coat protein zeta2-cop-related protein putativ	-0.228	
chr4	695175	696036	PFD0750w	nuclear cap-binding protein putative	-0.341	
chr4	696623	697312	PFD0755c	adenylate kinase 1	0.173	

chr4	698114	699499	PFD0760c	hypothetical protein conserved	0.114
chr4	700889	703516	PFD0765w	RING finger protein putative	0.060
chr4	704901	705751	PFD0770c	ribosomal protein l15 putative	-0.507
chr4	708509	711145	PFD0775c	hypothetical RNA binding protein	-0.153
chr4	713512	715992	PFD0780w	glutamyl-tRNA%28Gln%29 amidotransferase subunit A	-0.114
chr4	716443	717066	PFD0785c	hypothetical protein conserved	-0.093
chr4	718075	722472	PFD0790c	DNA replication licensing factor putative	-0.343
chr4	725185	729657	PFD0795w	hypothetical protein conserved	-0.298
chr4	730056	731225	PFD0800c	hypothetical protein conserved	-0.008
chr4	733248	734111	PFD0805w	prohibitin-like protein putative	0.272
chr4	734693	734962	PFD0807c	PfMNL-2 CISD1-like iron-sulfur protein putative	0.132
chr4	736944	738205	PFD0810w	small GTP-binding protein sar1	-0.057
chr4	739598	746854	PFD0815c	hypothetical protein conserved	-0.018
chr4	749130	750072	PFD0820w	hypothetical protein conserved	-0.053
chr4	750592	752618	PFD0825c	RNA-binding protein of pumilio%2Fmpt5 family putative	-0.139
chr4	755069	756895	PFD0830w	bifunctional dihydrofolate reductase-thymidylate synthas(	-0.188
chr4	757841	760693	PFD0835c	LETM1-like protein putative	0.082
chr4	763275	783584	PFD0840w	hypothetical protein conserved	0.154
chr4	784181	786405	PFD0850c	hypothetical protein conserved	-0.005
chr4	787231	790227	PFD0855c	hypothetical protein conserved	0.029
chr4	790602	793411	PFD0860w	hypothetical protein conserved	0.257
chr4	794224	796323	PFD0865c	cdc2-related protein kinase 1	-0.200
chr4	797705	799966	PFD0870w	hypothetical protein conserved	0.042
chr4	800808	816685	PFD0872w	hypothetical protein conserved	-0.028
chr4	817976	821623	PFD0875c	hypothetical protein conserved	0.142
chr4	824381	825670	PFD0880w	hypothetical protein conserved	0.007
chr4	826343	829321	PFD0885c	hypothetical protein conserved	-0.401
chr4	836943	837574	PFD0890w	hypothetical protein conserved	0.244
chr4	837969	839408	PFD0895c	Bet3 transport protein putative	-0.178
chr4	841374	847409	PFD0900w	hypothetical protein conserved	-0.023
chr4	848176	849885	PFD0905w	hypothetical protein conserved	-0.261
chr4	850542	851497	PFD0910w	hypothetical protein conserved	-0.157
chr4	854211	856212	PFD0915w	hypothetical protein conserved	-0.169
chr4	856773	859222	PFD0920w	hypothetical protein conserved	-0.219
chr4	859490	860546	PFD0925w	hypothetical protein conserved	0.033

chr4	861859	862269	PFD0930w	CGI-141 protein homolog putative	-0.070
chr4	862878	864029	PFD0935c	hypothetical protein conserved	-0.208
chr4	864978	872937	PFD0940w	hypothetical protein conserved	-0.012
chr4	873481	875091	PFD0945c	hypothetical membrane protein conserved	0.117
chr4	877717	878853	PFD0950w	ran binding protein 1	-0.169
chr4	880118	881095	PFD0955w	hypothetical protein conserved	0.011
chr4	881791	882501	PFD0960c	ribosomal protein L7Ae-related protein putative	-0.309
chr4	885004	900847	PFD0965W	putative phosphatidylinositol 4-kinase frameshifted	0.289
chr4	901593	911705	PFD0970c	Zinc finger putative	0.172
chr4	914037	915782	PFD0975w	ROI kinase-like protein	-0.147
chr4	917168	918952	PFD0980w	holo-%28acyl-carrier protein%29 synthase putative	0.130
chr4	924970	935391	PFD0985w	hypothetical protein conserved	0.011
chr4	936320	937120	PFD0990w	ribosome recycling factor putative	0.288
chr4	937692	938588	PFD0993c	hypothetical protein conserved	0.351
chr4	939470	946314	PFD0995c	erythrocyte membrane protein 1 %28PfEMP1%29	5.111
chr4	962506	970050	PFD1005c	erythrocyte membrane protein 1 %28PfEMP1%29	5.093
chr4	971526	972712	PFD1010w	rifin	5.111
chr4	973470	981030	PFD1015c	erythrocyte membrane protein 1 %28PfEMP1%29	4.829
chr4	986508	987434	PFD1020c	rifin	5.289
chr4	990641	991887	PFD1025w	erythrocyte membrane protein 1 %28PfEMP1%29-like ps	4.701
chr4	992282	993323	PFD1004c	hypothetical protein conserved	2.339
chr4	993740	998800	PFD1030c	hypothetical protein conserved	1.108
chr4	1000438	1000692	PFD1006w	hypothetical protein	0.127
chr4	1003087	1004625	PFD1035w	steroid dehydrogenase kik-i putative	0.065
chr4	1006053	1007210	PFD1037w	conserved Plasmodium protein unknown function	0.039
chr4	1008902	1021687	PFD1045c	erythrocyte membrane-associated antigen putative	0.192
chr4	1026927	1028606	PFD1050w	alpha-tubulin ii	-0.399
chr4	1031088	1031839	PFD1055w	ribosomal protein S19s putative	-0.143
chr4	1033769	1042393	PFD1060w	u5 small nuclear ribonucleoprotein-specific protein putat	-0.086
chr4	1043137	1043559	PFD1065c	hypothetical protein conserved	0.067
chr4	1044910	1046082	PFD1070w	eukaryotic initiation factor putative	-0.511
chr4	1047004	1049745	PFD1045w	hypothetical membrane protein conserved	0.248
chr4	1047004	1049336	PFD1075w	conserved Plasmodium membrane protein unknown fun	0.216
chr4	1049821	1051136	PFD1080w	hypothetical protein conserved	0.059
chr4	1052136	1052918	PFD1085w	hypothetical protein conserved	0.174

chr4	1053710	1054334	PFD1090c	clathrin assembly protein putative	0.018
chr4	1054847	1055414	PFD1095w	BSD-domain protein putative	0.034
chr4	1056016	1057446	PFD1100c	hypothetical protein conserved	0.205
chr4	1060105	1060758	PFD1105w	hypothetical protein	0.469
chr4	1063436	1064990	PFD1110w	hypothetical membrane protein conserved	-0.786
chr4	1066229	1071067	PFD1115c	hypothetical protein conserved	0.054
chr4	1075873	1076283	PFD1120c	early transcribed membrane protein 4 ETRAMP4	-0.147
chr4	1080786	1081973	PFD1130w	hypothetical protein conserved	-0.146
chr4	1082365	1082927	PFD1135c	hypothetical protein	0.306
chr4	1085398	1086628	PFD1140w	hypothetical protein conserved in P.falciparum	0.101
chr4	1086802	1088589	PFD1145c	reticulocyte binding protein homolog 5 Rh5	0.334
chr4	1090419	1095717	PFD1150c	reticulocyte binding protein homolog 4 Rh4	0.488
chr4	1097578	1102275	PFD1155w	erythrocyte binding antigen-165	1.809
chr4	1104251	1111551	PFD1160w	surface-associated interspersed gene 4.2 %28SURFIN4	1.758
chr4	1113689	1115922	PFD1165w	FIKK protein kinase	0.154
chr4	1116974	1118115	PFD1170c	RESA-like protein truncated	0.284
chr4	1122059	1126043	PFD1175w	Plasmodium falciparum trophozoite antigen r45-like prote	0.603
chr4	1127614	1128632	PFD1180w	trophozoite antigen r45-like protein truncated	1.378
chr4	1131406	1132429	PFD1185w	hypothetical protein conserved in P.falciparum	2.675
chr4	1133287	1133655	PFD1190c	hypothetical protein	4.765
chr4	1134195	1134520	PFD1195c	hypothetical protein	4.231
chr4	1138042	1138802	PFD1200c	hypothetical protein conserved in P.falciparum	2.948
chr4	1140213	1141012	PFD1205w	hypothetical integral membrane protein conserved in P. f	3.789
chr4	1145209	1145718	PFD1210w	hypothetical protein conserved in P.falciparum	4.590
chr4	1147614	1148446	PFD1215w	hypothetical protein conserved in P.falciparum	4.349
chr4	1150294	1151319	PFD1220c	stevor	4.838
chr4	1152896	1153810	PFD1200w	RIF pseudogene RIFIN pseudogene	5.148
chr4	1156126	1157255	PFD1230c	rifin	5.167
chr4	1176675	1183848	PFD1245c	erythrocyte membrane protein 1 %28PfEMP1%29	4.765
chr4	1192754	1195747	PFD1250w	hypothetical protein conserved in P falciparum	4.055
chr5	20929	28456	PFE0005w	erythrocyte membrane protein 1 %28PfEMP1%29	3.961
chr5	29923	30963	PFE0015c	RIF pseudogene RIFIN pseudogene	3.568
chr5	33056	34378	PFE0020c	rifin	4.906
chr5	36464	37576	PFE0025c	rifin	4.866
chr5	39443	40488	PFE0030c	stevor pseudogene	4.287

chr5	41129	41403	PFE0035c	RIF pseudogene RIFIN pseudogene	3.109
chr5	43327	47761	PFE0040c	Mature parasite-infected erythrocyte surface antigen %28	0.799
chr5	50422	52510	PFE0045c	kinase putative	0.012
chr5	55841	56872	PFE0050w	hypothetical protein	0.127
chr5	57954	59726	PFE0055c	heat shock protein putative	0.085
chr5	64065	65489	PFE0060w	hypothetical protein	-0.016
chr5	68930	70113	PFE0065w	skeleton binding protein1 PfSBP1	0.502
chr5	74509	79842	PFE0070w	interspersed repeat antigen putative	1.710
chr5	81134	82336	PFE0075c	rhoptry-associated protein 3 RAP3	0.305
chr5	84041	85237	PFE0080c	rhoptry-associated protein 2 RAP2	-0.006
chr5	86820	89201	PFE0085c	hypothetical protein conserved	0.245
chr5	91361	94591	PFE0090w	hypothetical protein conserved	-0.058
chr5	95161	96633	PFE0095c	hypothetical protein conserved	0.051
chr5	97657	101895	PFE0100w	hypothetical protein conserved	0.435
chr5	102193	102897	PFE0105c	hypothetical protein conserved	0.520
chr5	104256	105737	PFE0110w	hypothetical protein conserved	-0.294
chr5	107075	107938	PFE0115c	hypothetical protein conserved	-0.055
chr5	109898	111691	PFE0120c	Merozoite Surface Protein 8 MSP8	0.026
chr5	114149	115933	PFE0125w	hypothetical protein conserved	0.104
chr5	117163	121455	PFE0130c	hypothetical protein conserved	0.011
chr5	122449	122862	PFE0135w	hypothetical protein conserved	-0.330
chr5	123332	123904	PFE0140c	hypothetical protein conserved	-0.039
chr5	125116	126514	PFE0145w	50S ribosomal subunit protein L28 putative	-0.053
chr5	127064	128677	PFE0150c	4-diphosphocytidyl-2c-methyl-D-erythritol kinase %28CM	-0.076
chr5	130453	133392	PFE0155w	hypothetical protein conserved	0.053
chr5	135004	135930	PFE0160c	Ser%2FArg-rich splicing factor putative	-0.331
chr5	140710	141471	PFE0165w	actin depolymerizing factor putative	0.010
chr5	142871	145423	PFE0170c	protein kinase putative	-0.025
chr5	146639	150131	PFE0175c	unconventional myosin pfm-b	-0.144
chr5	150354	150912	PFE0180w	hypothetical protein	0.055
chr5	151422	152384	PFE0185c	60S ribosomal subunit protein L31 putative	0.081
chr5	153889	156057	PFE0190c	hypothetical protein conserved	0.419
chr5	157625	164968	PFE0195w	cation transporting P-ATPase	-0.224
chr5	165441	166642	PFE0200c	hypothetical protein conserved	0.098
chr5	167649	169502	PFE0205w	ATP-dependent helicase putative	0.004

chr5	169549	170590	PFE0210c	hypothetical protein conserved	0.237
chr5	172150	174417	PFE0215w	ATP-dependent helicase putative	-0.544
chr5	174989	176433	PFE0220w	hypothetical protein conserved	-0.102
chr5	177410	178555	PFE0225w	3-methyl-2-oxobutanoate dehydrogenase %28lipoamide	-0.857
chr5	180421	187470	PFE0230w	hypothetical protein conserved	0.184
chr5	187813	204732	PFE0240c	hypothetical protein conserved	0.023
chr5	206450	207822	PFE0240w	hypothetical protein conserved	0.007
chr5	209062	218189	PFE0245c	hypothetical protein conserved	-0.018
chr5	220839	229253	PFE0250w	hypothetical protein conserved	-0.105
chr5	230695	232392	PFE0255w	actin-related protein putative	-0.083
chr5	233815	235650	PFE0260w	UDP-N-acetyl glucosamine%3AUMP antiporter	0.385
chr5	236143	238365	PFE0265c	hypothetical protein conserved	0.093
chr5	239456	243880	PFE0270c	DNA repair protein putative	-0.444
chr5	245933	246934	PFE0275w	hypothetical protein conserved	-0.281
chr5	247801	250575	PFE0280c	hypothetical protein conserved	-0.063
chr5	252047	252606	PFE0285c	ubiquitin-like protein putative	-0.481
chr5	254263	255517	PFE0290c	hypothetical protein conserved	-0.062
chr5	256225	257378	PFE0295w	hypothetical protein conserved	0.266
chr5	257748	258831	PFE0300c	60S ribosomal subunit protein L24 putative	-0.203
chr5	259764	260747	PFE0305w	transcription initiation factor TFiid TATA-binding protein	-0.081
chr5	260961	262184	PFE0310c	hypothetical protein conserved	0.206
chr5	262672	263922	PFE0315c	hypothetical protein	-0.254
chr5	265595	268567	PFE0320w	hypothetical protein	-0.110
chr5	269319	278345	PFE0325w	hypothetical protein conserved	-0.277
chr5	278781	279492	PFE0328w	hypothetical protein conserved pseudogene	-0.069
chr5	280886	283345	PFE0330w	hypothetical protein conserved	-0.357
chr5	284691	288023	PFE0335w	hypothetical protein conserved	-0.167
chr5	288776	291055	PFE0340c	rhomboid protease putative	-0.417
chr5	292696	293474	PFE0345c	hypothetical protein conserved	-0.006
chr5	294668	295903	PFE0350c	60S ribosomal subunit protein L4%2FL1 putative	-0.498
chr5	298427	300736	PFE0355c	serine protease belonging to subtilisin family putative	-0.399
chr5	301930	304611	PFE0360c	hypothetical protein conserved	0.046
chr5	305416	306658	PFE0365c	hypothetical protein conserved	0.170
chr5	307490	309556	PFE0370c	subtilisin-like protease precursor putative	-0.705
chr5	313199	315157	PFE0375w	cell differentiation protein rcd1 putative	-0.587

chr5	316766	318361	PFE0380c	hypothetical protein conserved	-0.102	
chr5	322239	326822	PFE0385w	hypothetical protein conserved	-0.322	
chr5	327656	328530	PFE0390w	hypothetical protein conserved	0.063	
chr5	328666	329715	PFE0395c	hypothetical protein conserved	-0.520	
chr5	331680	336704	PFE0400w	hypothetical protein conserved	-0.431	
chr5	337426	339025	PFE0405c	hypothetical protein conserved	-0.344	
chr5	342205	343233	PFE0410w	triose or hexose phosphate %2F phosphate translocator	-0.441	
chr5	344931	346744	PFE0415w	transcription factor iib putative	-0.154	
chr5	347219	355039	PFE0420c	guanidine nucleotide exchange factor putative	-0.155	
chr5	356222	357010	PFE0425w	hypothetical protein conserved	-0.406	
chr5	358261	362733	PFE0430w	ATP-dependent RNA helicase putative	-0.215	
chr5	363588	364442	PFE0435c	single-strand binding protein putative	-0.263	
chr5	367022	376426	PFE0440w	hypothetical protein conserved	0.038	
chr5	376855	378452	PFE0445c	SNAP protein %28soluble N-ethylmaleimide-sensitive fa	-0.486	
chr5	381077	386203	PFE0450w	chromosome condensation protein putative	-0.039	
chr5	386956	388505	PFE0455w	phosphatase 1 regulatory subunit putative	0.122	
chr5	388696	389191	PFE0460c	hypothetical protein conserved	-0.237	
chr5	389914	398997	PFE0465c	RNA polymerase I	-0.131	
chr5	401437	402771	PFE0470w	hypothetical protein conserved	-0.213	
chr5	403342	405642	PFE0475w	asparagine -- t RNA ligase putative	-0.393	
chr5	405961	407897	PFE0480c	hypothetical protein conserved	0.086	
chr5	410268	415308	PFE0485w	phosphatidylinositol 4-kinase putative	-0.096	
chr5	416365	417309	PFE0490w	hypothetical protein conserved	0.249	
chr5	418136	421425	PFE0495w	hypothetical protein conserved	0.251	
chr5	422746	430353	PFE0500c	hypothetical protein conserved	0.104	
chr5	437493	439736	PFE0505w	cyclophilin putative	0.006	
chr5	440777	441877	PFE0510c	hypothetical protein conserved	0.051	
chr5	444258	445577	PFE0515w	hypothetical protein conserved	0.531	
chr5	445981	448500	PFE0520c	topoisomerase I	0.222	5cenL
chr5	459299	460342	PFE0530w	hypothetical protein conserved	0.771	5cenR
chr5	461015	461368	PFE0535w	hypothetical protein conserved	0.395	
chr5	461579	464240	PFE0540w	WD-repeat potein putative	0.378	
chr5	465637	466933	PFE0543c	hypothetical protein	0.453	
chr5	467583	468098	PFE0545c	histamine-releasing factor putative	-0.511	
chr5	469274	469840	PFE0550w	hypothetical protein conserved	0.126	

chr5	471053	474056	PFE0555w	stearoyl-CoA desaturase %28acyl-CoA desaturase faty	-0.079
chr5	474485	478744	PFE0560c	hypothetical protein conserved	0.309
chr5	481421	482566	PFE0565w	hypothetical protein conserved	0.167
chr5	486600	516785	PFE0570w	hypothetical protein conserved	0.119
chr5	517257	517883	PFE0575c	hypothetical protein conserved	0.302
chr5	519127	520076	PFE0580w	hypothetical protein conserved	0.258
chr5	520718	522691	PFE0585c	myo-inositol 1-phosphate synthase putative	-0.172
chr5	525024	527216	PFE0590w	hypothetical protein conserved	0.291
chr5	527637	528737	PFE0595w	hypothetical protein conserved	-0.006
chr5	528844	529407	PFE0600c	hypothetical protein conserved	-0.074
chr5	529932	531899	PFE0605c	glutathione synthetase	-0.100
chr5	533554	534336	PFE0610c	CDK-activating kinase assembly factor putative	-0.231
chr5	536271	536975	PFE0615w	hypothetical protein conserved	-0.354
chr5	539729	542591	PFE0620c	hypothetical protein conserved	0.171
chr5	544538	545991	PFE0625w	Rab1b GTPase	-0.462
chr5	546732	547577	PFE0630c	orotate phosphoribosyltransferase putative	0.156
chr5	548404	553338	PFE0635c	hypothetical protein conserved	-0.053
chr5	553988	554785	PFE0640w	hypothetical protein conserved	0.114
chr5	555262	556071	PFE0645w	hypothetical protein conserved	-0.345
chr5	557037	558010	PFE0650c	hypothetical protein conserved	-0.346
chr5	559511	568382	PFE0655w	hypothetical protein conserved	0.089
chr5	569180	569917	PFE0660c	purine nucleotide phosphorylase putative	-0.745
chr5	571056	572659	PFE0665c	GTP-binding protein putative	0.065
chr5	573586	574246	PFE0670w	hypothetical protein conserved	-0.046
chr5	574939	578503	PFE0675c	deoxyribodipyrimidine photolyase %28photoreactivating	-0.266
chr5	580848	581836	PFE0680w	hypothetical protein conserved	0.044
chr5	581882	584122	PFE0685w	hypothetical protein conserved	0.017
chr5	585355	585978	PFE0690c	PfRab1a	-0.346
chr5	587750	588675	PFE0695w	hypothetical protein conserved	0.077
chr5	588919	590046	PFE0700c	hypothetical protein conserved	-0.198
chr5	591336	595661	PFE0705c	helicase belonging to UvrD family putative	-0.112
chr5	596816	599419	PFE0710w	hypothetical protein conserved	0.148
chr5	600045	603431	PFE0715w	asparagine -- tRNA ligase putative	-0.025
chr5	605025	606328	PFE0725c	hypothetical protein conserved	-0.252
chr5	607103	608449	PFE0730c	ribose 5-phosphate epimerase putative	-0.318

chr5	610087	614769	PFE0735w	hypothetical protein conserved	0.009
chr5	615280	617991	PFE0740c	hypothetical protein conserved	-0.048
chr5	618627	619943	PFE0745w	hypothetical protein conserved	0.118
chr5	620808	623354	PFE0750c	hypothetical protein conserved	-0.536
chr5	624717	626479	PFE0755c	rhomboid protease putative	0.156
chr5	627296	627959	PFE0760w	hypothetical protein conserved	-0.294
chr5	628979	635380	PFE0765w	phosphatidylinositol 3-kinase putative	-0.090
chr5	637426	640379	PFE0770w	hypothetical protein conserved	-0.024
chr5	641135	644683	PFE0775c	amino acid transporter	-0.064
chr5	646657	652104	PFE0780w	hypothetical protein conserved	-0.178
chr5	653964	655334	PFE0785c	drug%2Fmetabolite transporter	-0.677
chr5	658421	658675	PFE0790c	BolA-like protein conserved	-0.440
chr5	660148	661134	PFE0795c	nif-like protein putative	-0.128
chr5	663549	666915	PFE0800w	hypothetical protein conserved	-0.011
chr5	667484	674859	PFE0805w	cation-transporting ATPase 1	-0.099
chr5	675807	676612	PFE0810c	40S ribosomal subunit protein S14 putative	-0.459
chr5	678908	680953	PFE0815w	hypothetical protein conserved	-0.670
chr5	681428	682435	PFE0820c	hypothetical protein conserved	-0.202
chr5	683706	685857	PFE0825w	metabolite drug transporter	-0.544
chr5	686808	691640	PFE0830c	hypothetical protein conserved	-0.158
chr5	694490	698776	PFE0835w	ubiquitin carboxyl-terminal hydrolase 2 putative	-0.141
chr5	700118	707254	PFE0840c	hypothetical protein conserved	-0.062
chr5	712760	713770	PFE0845c	60S ribosomal subunit protein L8 putative	-0.807
chr5	715763	716673	PFE0850c	60S ribosomal protein L12 putative	0.075
chr5	718139	719853	PFE0855c	hypothetical protein conserved	0.314
chr5	720741	722639	PFE0860c	hypothetical protein conserved	-0.026
chr5	724679	725749	PFE0865c	splicing factor putative	-0.279
chr5	731405	735160	PFE0870w	transcriptional regulator putative	-0.256
chr5	735885	738572	PFE0875c	hypothetical protein conserved	-0.246
chr5	740154	741032	PFE0880c	f-actin capping protein beta subunit putative	-0.078
chr5	743177	745327	PFE0885w	eukaryotictranslation initiation factor 3 subunit putative	-0.613
chr5	745796	746632	PFE0890c	hypothetical protein conserved	0.086
chr5	747793	749675	PFE0895c	Zinc finger protein putative	-0.567
chr5	750659	752433	PFE0900w	hypothetical protein conserved	-0.347
chr5	753296	757542	PFE0905w	hypothetical protein conserved	-0.322

chr5	758083	758816	PFE0910w	hypothetical protein conserved	-0.216
chr5	759829	760996	PFE0915c	proteasome subunit beta type 1	-0.409
chr5	762101	762790	PFE0920c	cyclin2 related protein putative	-0.308
chr5	763530	766901	PFE0925c	snrnp protein putative	0.077
chr5	769308	773138	PFE0930w	hypothetical protein conserved	-0.243
chr5	774073	780436	PFE0935c	RNA-binding protein putative	0.031
chr5	783653	784088	PFE0940c	hypothetical protein	0.196
chr5	784312	784989	PFE0945c	hypothetical protein conserved	-0.581
chr5	785662	786563	PFE0950c	hypothetical protein conserved	0.154
chr5	788597	789478	PFE0955w	hypothetical protein conserved	-0.167
chr5	790442	790771	PFE0960w	50S ribosomal subunit protein L14 putative	-0.316
chr5	791717	792934	PFE0965c	vacuolar ATP synthetase putative	-0.335
chr5	795525	797471	PFE0970w	cytochrome c oxidase assembly protein %28heme A%3A	-0.312
chr5	798034	798773	PFE0975c	40S ribosomal subunit protein S24 putative	-0.163
chr5	801447	809124	PFE0980c	hypothetical protein conserved	-0.146
chr5	812645	814912	PFE0985w	hypothetical protein conserved	-0.041
chr5	818628	819136	PFE0990w	hypothetical protein conserved	-0.303
chr5	820716	822905	PFE0995c	hypothetical protein conserved	-0.275
chr5	825832	827409	PFE1000c	hypothetical protein conserved	0.214
chr5	828950	829880	PFE1005w	40S ribosomal subunit protein S9 putative	-0.354
chr5	831614	834184	PFE1010w	protein phosphatase 2c putative	-0.121
chr5	835311	837404	PFE1015c	hypothetical protein conserved	-0.100
chr5	838425	839080	PFE1020w	u6 snRNA-associated sm-like protein Lsm2 putative	-0.341
chr5	839652	840557	PFE1025c	hypothetical protein conserved	0.057
chr5	841720	842652	PFE1030c	phosphomethylpyrimidine kinase putative	-0.157
chr5	843583	844574	PFE1035c	BIS%285%27-nucleosyl%29-tetraphosphatase %28diad	-0.079
chr5	845840	848764	PFE1040c	hypothetical protein conserved	0.096
chr5	849859	856023	PFE1045c	hypothetical protein conserved	0.131
chr5	857035	858474	PFE1050w	adenosylhomocysteinase%28S-adenosyl-L-homocystein	-0.757
chr5	859370	861060	PFE1055c	hypothetical protein conserved	-0.262
chr5	861927	867580	PFE1060c	hypothetical protein conserved	0.417
chr5	868910	869875	PFE1065w	hypothetical protein conserved	0.215
chr5	870181	874567	PFE1070c	hypothetical protein conserved	0.249
chr5	875870	877558	PFE1075c	hypothetical protein conserved	0.313
chr5	878065	879759	PFE1080w	ribosomal large subunit pseudouridylate synthase putati	-0.148

chr5	880062	880352	PFE1082c	conserved Plasmodium protein unknown function	0.135
chr5	882373	884898	PFE1085w	DEAD-box subfamily ATP-dependent helicase putative	-0.126
chr5	885711	888229	PFE1090w	nuceotide binding protein putative	-0.049
chr5	888654	894724	PFE1095w	hypothetical protein conserved	0.045
chr5	896013	896935	PFE1100w	hypothetical protein conserved	0.078
chr5	897460	899751	PFE1105c	hypothetical protein conserved	-0.063
chr5	901409	902168	PFE1110w	hypothetical protein conserved	-0.020
chr5	902608	904839	PFE1115c	s-adenosylmethionine-dependent methyltransferase put	-0.144
chr5	907832	936310	PFE1120w	hypothetical protein conserved	0.077
chr5	937310	938032	PFE1125w	50S ribosomal subunit protein L17 putative	-0.196
chr5	940244	941695	PFE1130w	hypothetical protein conserved	-0.544
chr5	942762	943868	PFE1135w	hypothetical protein conserved	0.082
chr5	944179	944926	PFE1140c	G10 protein putative	0.048
chr5	948946	953604	PFE1145w	hypothetical protein conserved	0.121
chr5	957885	962144	PFE1150w	multidrug resistance protein	-0.344
chr5	963222	965039	PFE1155c	mitochondrial processing peptidase alpha subunit putati	-0.485
chr5	966118	969732	PFE1160w	hypothetical protein conserved	0.150
chr5	970261	970957	PFE1165c	hypothetical protein conserved	-0.421
chr5	973513	975659	PFE1170w	hypothetical protein	-0.129
chr5	976685	977810	PFE1173c	outer arm dynein lc3 putative	0.172
chr5	978660	979865	PFE1175w	hypothetical protein conserved	-0.219
chr5	980749	985349	PFE1180c	hypothetical protein conserved	0.373
chr5	990000	992054	PFE1185w	transporter putative	-0.163
chr5	993428	993865	PFE1190c	hypothetical protein conserved	-0.132
chr5	998748	1002119	PFE1195w	karyopherin beta	-0.558
chr5	1004232	1008103	PFE1200w	hypothetical protein conserved	-0.093
chr5	1008631	1009399	PFE1205c	hypothetical protein conserved	-0.325
chr5	1010946	1012166	PFE1210c	hypothetical protein conserved	0.011
chr5	1013448	1014545	PFE1215c	developmentally regulated GTP-binding protein 1 putati	-0.398
chr5	1016193	1016564	PFE1220w	hypothetical protein conserved	0.153
chr5	1017804	1018577	PFE1225w	50S ribosomal subunit protein L12 putative	0.021
chr5	1019580	1019897	PFE1230c	hypothetical protein conserved	-0.538
chr5	1021403	1024302	PFE1235c	hypothetical protein	-0.036
chr5	1027391	1030357	PFE1240w	hypothetical protein conserved	-0.198
chr5	1036224	1038338	PFE1245w	hypothetical protein conserved	-0.587

chr5	1041741	1043762	PFE1250w	acetyl-CoA synthetase PfACS10	-0.707
chr5	1045458	1051249	PFE1255w	hypothetical protein conserved	0.058
chr5	1052464	1053909	PFE1260c	hypothetical protein conserved	-0.001
chr5	1057647	1059050	PFE1265w	G-protein coupled receptor putative	-0.104
chr5	1059820	1065505	PFE1270c	hypothetical protein conserved	-0.104
chr5	1066213	1067708	PFE1275c	RNA methyltransferase putative	0.243
chr5	1068512	1069281	PFE1280w	hypothetical protein conserved	0.203
chr5	1071243	1072145	PFE1285w	hypothetical protein conserved	-0.674
chr5	1073642	1075640	PFE1290w	serine%2Fthreonine-protein kinase. Pfnek-2	0.023
chr5	1075909	1078329	PFE1295c	hypothetical protein conserved	0.311
chr5	1079774	1081003	PFE1300w	hypothetical protein conserved	0.382
chr5	1081553	1084026	PFE1305c	hypothetical protein conserved	0.026
chr5	1085618	1086589	PFE1310c	hypothetical protein conserved	-0.177
chr5	1088418	1089503	PFE1315w	hypothetical protein conserved	-0.103
chr5	1090925	1095079	PFE1320w	hypothetical protein conserved	-0.043
chr5	1101198	1114772	PFE1325w	hypothetical protein conserved	-0.487
chr5	1117256	1119646	PFE1330c	hypothetical protein conserved	0.099
chr5	1120699	1121805	PFE1335c	hypothetical protein conserved	-0.037
chr5	1123605	1124590	PFE1340w	transmembrane protein putative	-0.228
chr5	1125094	1128141	PFE1345c	minichromosome maintenance protein 3 putative	-0.340
chr5	1129954	1130749	PFE1350c	ubiquitin-conjugating enzyme putative	-0.400
chr5	1132932	1134849	PFE1355c	ubiquitin carboxyl-terminal hydrolase putative	-0.142
chr5	1135971	1136858	PFE1360c	methionine aminopeptidase putative	-0.296
chr5	1137710	1137865	PFE1363c	hypothetical protein	0.069
chr5	1140621	1141148	PFE1365w	hypothetical protein conserved	-0.110
chr5	1143612	1145353	PFE1370w	hsp70 interacting protein putative	-0.081
chr5	1145999	1148804	PFE1375c	hypothetical protein conserved	-0.051
chr5	1153670	1155667	PFE1390w	RNA helicase-1	-0.631
chr5	1156199	1156573	PFE1395c	hypothetical protein conserved	0.071
chr5	1157834	1161547	PFE1400c	beta adaptin protein putative	-0.222
chr5	1163402	1165066	PFE1405c	eukaryotictranslation initiation factor 3 subunit 6 putativ	0.023
chr5	1166514	1168004	PFE1410c	hypothetical protein conserved	-0.331
chr5	1171653	1173884	PFE1415w	cell cycle regulator with zn-finger domain putative	-0.114
chr5	1174900	1176834	PFE1420w	F-actin capping protein alpha subunit putative	0.199
chr5	1176962	1177279	PFE1425c	hypothetical protein conserved	-0.153

chr5	1178269	1178883	PFE1430c	cyclophilin putative	-0.679
chr5	1180418	1182031	PFE1435c	hypothetical protein conserved	0.177
chr5	1183775	1185632	PFE1440c	hypothetical protein conserved	0.072
chr5	1186780	1188939	PFE1445c	hypothetical protein conserved	-0.340
chr5	1190993	1191675	PFE1450c	hypothetical protein conserved	-0.113
chr5	1192966	1195325	PFE1455w	sugar transporter putative	-0.225
chr5	1196308	1197382	PFE1460w	hypothetical protein conserved	-0.071
chr5	1199158	1205180	PFE1465w	hypothetical protein conserved	-0.253
chr5	1206466	1207672	PFE1470w	cell cycle regulator protein putative	0.259
chr5	1208999	1212095	PFE1480c	hypothetical protein conserved	-0.061
chr5	1213526	1219369	PFE1485w	hypothetical protein conserved	-0.187
chr5	1220770	1221985	PFE1490c	hypothetical protein conserved	-0.412
chr5	1222419	1222748	PFE1495w	hypothetical protein	1.703
chr5	1224591	1224884	PFE1500c	hypothetical protein conserved	0.289
chr5	1226876	1227750	PFE1505w	hypothetical protein conserved	-0.508
chr5	1229070	1230644	PFE1510c	phosphoenolpyruvate%3Aphosphate antiporter putative	-0.313
chr5	1233394	1238079	PFE1515w	hypothetical protein conserved	0.144
chr5	1238626	1243980	PFE1520c	hypothetical protein conserved	0.197
chr5	1244744	1248352	PFE1525w	hypothetical protein conserved	0.421
chr5	1248361	1249954	PFE1530c	hypothetical protein conserved	-0.186
chr5	1250730	1254183	PFE1535w	hypothetical protein conserved	0.048
chr5	1255486	1257131	PFE1540w	hypothetical protein conserved	-0.008
chr5	1257716	1265743	PFE1545c	diaphanous homolog putative	-0.140
chr5	1267403	1269532	PFE1550w	hypothetical protein conserved	-0.068
chr5	1270381	1278477	PFE1555c	hypothetical protein conserved	0.253
chr5	1279951	1280592	PFE1560c	hypothetical protein conserved	0.193
chr5	1281282	1282247	PFE1565w	hypothetical protein conserved	-0.270
chr5	1282584	1286099	PFE1570c	hypothetical protein conserved	0.200
chr5	1289594	1291685	MAL5_18S	MAL5_18S	0.781
chr5	1291686	1292044	MAL5_ITS1	MAL5_ITS1	0.568
chr5	1292403	1296192	MAL5_28S	MAL5_28S	0.806
chr5	1301219	1301764	PFE1590w	early transcribed membrane protein 5 ETRAMP5	-0.142
chr5	1303657	1304521	PFE1595c	hypothetical protein conserved	-0.257
chr5	1308273	1309935	PFE1600w	hypothetical protein conserved in P. falciparum	-0.035
chr5	1312466	1314149	PFE1605w	DNAJ protein	-0.013

chr5	1316337	1316841	PFE1610w	hypothetical protein	0.335
chr5	1318526	1318984	PFE1615c	hypothetical protein	0.484
chr5	1322395	1322718	PFE1620c	erythrocyte membrane protein 1 %28PfEMP1%29	2.501
chr5	1322795	1323082	PFE1625c	erythrocyte membrane protein 1 %28PfEMP1%29 pseud	2.981
chr5	1326240	1327424	PFE1630w	rifin	4.659
chr5	1329844	1330623	PFE1635w	RIF pseudogene RIFIN pseudogene	4.161
chr5	1333465	1342959	PFE1640w	erythrocyte membrane protein 1 %28PfEMP1%29 trunc	3.835
chr6	653	1432	PFF0005c	erythrocyte membrane protein 1 %28PfEMP1%29 pseud	5.019
chr6	3503	12835	PFF0010w	erythrocyte membrane protein 1 %28PfEMP1%29	4.932
chr6	15065	16410	PFF0015c	rifin	3.421
chr6	18586	22721	PFF0020c	erythrocyte membrane protein 1 %28PfEMP1%29-like pr	5.602
chr6	26557	27830	PFF0025w	rifin	4.051
chr6	29618	31484	PFF0030c	erythrocyte membrane protein 1 %28PfEMP1%29 pseud	4.426
chr6	31965	33244	PFF0035c	rifin	5.159
chr6	35355	36613	PFF0040c	rifin pseudogene	4.526
chr6	65785	67392	PFF0075c	hypothetical protein conserved in P.falciparum	2.587
chr6	68830	69690	PFF0080c	hypothetical protein conserved	2.598
chr6	70945	71932	PFF0085w	hypothetical protein conserved in P.falciparum	2.603
chr6	78187	78781	PFF0090w	hypothetical protein	-0.317
chr6	82606	86919	PFF0095c	hypothetical protein	-0.162
chr6	92503	96665	PFF0100w	putative ATP dependent RNA helicase	-0.270
chr6	97887	98948	PFF0105w	MYND finger domain protein	-0.387
chr6	99919	100722	PFF0110w	hypothetical protein	0.191
chr6	101382	104558	PFF0115c	elongation factor G putative	-0.424
chr6	105500	106639	PFF0120w	putative geranylgeranyltransferase	-0.268
chr6	107034	111661	PFF0125c	hypothetical protein	0.172
chr6	113821	115221	PFF0130c	hypothetical protein conserved	0.018
chr6	116081	118021	PFF0135w	hypothetical protein conserved	0.069
chr6	118635	120200	PFF0140c	hypothetical protein conserved	0.091
chr6	122631	124409	PFF0145w	hypothetical protein	0.193
chr6	125455	126813	PFF0150c	hypothetical protein	-0.169
chr6	128618	130033	PFF0155w	bcs1-like protein putative	-0.497
chr6	130326	132035	PFF0160c	dihydroorotate dehydrogenase mitochondrial precursor	-0.320
chr6	133147	136458	PFF0165c	hypothetical protein conserved	0.281
chr6	139190	140515	PFF0170w	calcium antiporter putative	-0.347

chr6	141415	148940	PFF0175c	hypothetical protein conserved	0.852
chr6	151052	152383	PFF0180w	phenylalanyl-tRNA synthetase putative	-0.060
chr6	152538	160452	PFF0185c	hypothetical protein	-0.119
chr6	161251	162378	PFF0190c	hypothetical protein	0.405
chr6	163825	169857	PFF0195c	hypothetical protein conserved	0.108
chr6	171298	177237	PFF0200c	hypothetical protein conserved	-0.198
chr6	180933	181478	PFF0205w	hypothetical protein conserved	-0.198
chr6	182213	184534	PFF0210w	hypothetical protein conserved	-0.153
chr6	184889	185840	PFF0215w	hypothetical protein conserved	-0.060
chr6	189009	198075	PFF0220w	hypothetical protein expressed	-0.169
chr6	198859	204352	PFF0225w	DNA helicase putative	0.026
chr6	204637	206204	PFF0230c	glyoxalase I putative	-0.629
chr6	206852	211156	PFF0235c	RAP protein putative	0.245
chr6	212412	213029	PFF0240c	hypothetical protein	-0.001
chr6	213555	214139	PFF0245w	50S ribosomal subunit L24 putative	-0.252
chr6	217237	219504	PFF0250w	RNA binding protein putative	-0.240
chr6	220404	221810	PFF0255c	hypothetical protein conserved	-0.548
chr6	222736	224284	PFF0260w	serine%2Fthreonine protein kinase Pfnek-5	-0.485
chr6	224610	225092	PFF0265c	calcium-binding protein putative	0.262
chr6	225343	227443	PFF0270c	cyclin dependent kinase binding protein putative	-0.095
chr6	228129	234832	PFF0275c	nucleoside diphosphate kinase putative	-0.053
chr6	236394	237248	PFF0280w	hypothetical protein conserved	-0.507
chr6	237808	244518	PFF0285c	DNA repair protein RAD50 putative	0.006
chr6	247637	249163	PFF0290w	long chain polyunsaturated fatty acid elongation enzyme	-0.339
chr6	250609	253800	PFF0295c	hypothetical protein conserved	-0.255
chr6	257600	259970	PFF0300w	RNA-binding protein putative	-0.369
chr6	261027	261941	PFF0305c	ubiquitin-conjugating enzyme E2 putative	-0.571
chr6	262822	263190	PFF0310w	hypothetical protein conserved	0.449
chr6	263932	264823	PFF0315c	hypothetical protein conserved	0.358
chr6	265835	268395	PFF0320c	polypyrimidine tract binding protein putative	-0.168
chr6	270550	277758	PFF0325c	hypothetical protein expressed conserved	-0.284
chr6	282045	287013	PFF0330w	coatomer alpha subunit putative	-0.433
chr6	288373	289272	PFF0335c	hypothetical protein	-0.663
chr6	290422	291081	PFF0340c	glutaredoxin-like protein putative	-0.302
chr6	293860	296793	PFF0345w	translation initiation factor IF-2 putative	-0.470

chr6	297937	301193	PFF0350w	MYND finger protein putative	0.004
chr6	301870	303286	PFF0355c	hypothetical protein conserved	-0.183
chr6	304634	306038	PFF0360w	uroporphyrinogen decarboxylase putative	-0.591
chr6	306380	309957	PFF0365c	G-protein associated signal transduction protein putative	0.055
chr6	311546	313111	PFF0370w	para-hydroxybenzoate--polyprenyltransferase %284- hyc	0.028
chr6	314017	316878	PFF0375c	hypothetical protein conserved	0.111
chr6	320736	328994	PFF0380w	hypothetical protein conserved	-0.225
chr6	329533	331721	PFF0385c	hypothetical protein conserved	-0.227
chr6	332339	334930	PFF0390w	hypothetical protein conserved	-0.034
chr6	335103	337364	PFF0395c	WD repeat protein putative	-0.168
chr6	337788	341756	PFF0400w	hypothetical protein conserved	-0.185
chr6	341870	342817	PFF0405c	hypothetical protein conserved	0.295
chr6	343208	349367	PFF0410w	hypothetical protein conserved	0.162
chr6	350215	350511	PFF0415c	hypothetical protein conserved	-0.217
chr6	351776	352771	PFF0420c	proteasome subunit alpha type 2 putative	-0.453
chr6	355225	356272	PFF0425w	hypothetical protein conserved	0.103
chr6	358387	360199	PFF0430w	chaperone putative	-0.553
chr6	362631	363875	PFF0435w	ornithine aminotransferase	-0.605
chr6	365283	368455	PFF0440w	hypothetical protein conserved	0.041
chr6	370257	388490	PFF0445w	hypothetical protein conserved	0.074
chr6	388911	390744	PFF0450c	Zn2%2B or Fe2%2B permease	-0.357
chr6	392987	394607	PFF0455w	citrate synthase-like protein putative	0.325
chr6	396238	399285	PFF0460w	hypothetical protein conserved	-0.003
chr6	399809	401620	PFF0465c	cardiolipin synthetase putative	-0.021
chr6	402742	407238	PFF0470w	hypothetical protein conserved	0.030
chr6	408037	411998	PFF0475w	hypothetical protein	0.051
chr6	413648	419776	PFF0480w	hypothetical protein conserved	-0.201
chr6	421152	422617	PFF0485c	zinc finger putative	-0.307
chr6	425800	431853	PFF0490w	hypothetical protein conserved	0.009
chr6	432936	434173	PFF0495w	ribosomal protein L19-like protein putative	-0.836
chr6	434802	436460	PFF0500c	step II splicing factor putative	-0.174
chr6	437658	440540	PFF0505c	hypothetical protein conserved	0.183
chr6	444742	445152	PFF0510w	histone H3 putative	0.123
chr6	445756	446358	PFF0515c	hypothetical protein conserved	0.388
chr6	449286	451193	PFF0520w	calcium-dependent protein kinase	-0.162

chr6	452662	453441	PFF0525w	hypothetical protein conserved	0.158	
chr6	453666	455684	PFF0530w	transketolase putative	-0.496	
chr6	456742	460584	PFF0535c	trancription factor putative	0.090	
chr6	463756	464671	PFF0540c	hypothetical membrane protein conserved	0.001	
chr6	466193	466444	PFF0545c	hypothetical protein conserved	-0.323	
chr6	467476	468637	PFF0550w	hypothetical protein conserved	-0.037	
chr6	470044	471219	PFF0555w	hypothetical protein conserved	0.372	
chr6	471653	474145	PFF0560c	hypothetical protein conserved	0.335	6cenL
chr6	482820	483902	PFF0565c	hypothetical protein conserved	1.216	6cenR
chr6	485109	487061	PFF0570c	hypothetical protein conserved	0.491	
chr6	488574	489151	PFF0573c	60S ribosomal protein L39 putative	0.474	
chr6	490853	501135	PFF0575c	hypothetical protein conserved	0.069	
chr6	506375	507193	PFF0580w	hypothetical protein conserved	-0.193	
chr6	507673	508281	PFF0585c	hypothetical protein conserved	0.056	
chr6	509155	511125	PFF0590c	homologue of human HSPC025	-0.241	
chr6	512967	519543	PFF0595c	leucine-rich repeat protein 5 LRR5	0.119	
chr6	520746	521933	PFF0600w	hypothetical protein conserved	0.056	
chr6	522244	522660	PFF0605c	hypothetical protein conserved	0.379	
chr6	523652	525100	PFF0610c	PP-loop family protein putative	-0.279	
chr6	525982	527025	PFF0615c	Plasmodium falciparum membrane protein pf12 precursc	-0.345	
chr6	528055	529170	PFF0620c	Pfs45-48 related protein putative	-0.174	
chr6	531121	533181	PFF0625w	nucleolar GTP-binding protein 1 putative	-0.652	
chr6	533919	535359	PFF0630c	hypothetical protein conserved	-0.178	
chr6	537891	538744	PFF0635w	hypothetical protein conserved	0.239	
chr6	539299	539669	PFF0640w	hypothetical protein conserved	-0.093	
chr6	540072	544793	PFF0645c	integral membrane protein	0.052	
chr6	546959	547736	PFF0650w	ribosomal protein L18 putative	-0.502	
chr6	548275	552366	PFF0655c	adapter-related protein putative	0.061	
chr6	554452	558223	PFF0660w	hypothetical protein conserved	-0.144	
chr6	559344	561455	PFF0665c	syntaxin binding protein putative	-0.169	
chr6	566134	578988	PFF0670w	hypothetical protein conserved	-0.289	
chr6	579430	585891	PFF0675c	myosin-like protein putative	0.074	
chr6	586448	588630	PFF0680c	thiamin-phosphate pyrophosphorylase putative	0.054	
chr6	589452	595899	PFF0685c	Hypothetical protein conserved	0.072	
chr6	589452	592259	PFF0683c	conserved Plasmodium protein unknown function	0.223	

chr6	597479	600512	PFF0690c	organic anion transporter	-0.328
chr6	604568	605335	PFF0695w	hypothetical protein conserved	-0.050
chr6	606137	607165	PFF0700c	60S ribosomal protein L19 putative	-0.298
chr6	608662	611097	PFF0705c	hypothetical protein conserved	0.315
chr6	612466	614764	PFF0710w	hypothetical protein conserved	0.040
chr6	615292	616548	PFF0715c	endonuclease iii homologue putative	-0.262
chr6	617757	621531	PFF0720w	hypothetical membrane protein conserved	-0.160
chr6	622267	622707	PFF0725w	hypothetical protein conserved	-0.538
chr6	623018	624316	PFF0730c	enoyl-acyl carrier reductase	-0.537
chr6	625360	627512	PFF0735w	hypothetical protein conserved	0.124
chr6	627688	630312	PFF0740c	Phosphatidylinositol glycan W putative	0.181
chr6	630965	640169	PFF0745c	ribonuclease putative	-0.033
chr6	644048	646165	PFF0750w	cyclin-dependent protein kinase predicted	-0.270
chr6	646633	655592	PFF0755c	hypothetical protein conserved	0.164
chr6	656372	657632	PFF0760w	RNA and export factor binding protein putative	0.114
chr6	657935	661785	PFF0765c	hypothetical protein conserved	-0.119
chr6	662431	667851	PFF0770c	hypothetical protein conserved	0.216
chr6	669856	671628	PFF0775w	pyridoxal kinase-like protein putative	-0.139
chr6	672115	672750	PFF0780w	hypothetical protein conserved	0.177
chr6	674114	676372	PFF0785w	hypothetical protein conserved	-0.239
chr6	676885	677568	PFF0790c	hypothetical protein conserved	0.366
chr6	678384	683426	PFF0795w	hypothetical protein conserved	0.312
chr6	684739	688854	PFF0800w	hypothetical protein conserved	-0.186
chr6	689251	691128	PFF0805c	hypothetical protein conserved	-0.230
chr6	691766	693316	PFF0810c	hypothetical protein conserved	-0.065
chr6	695926	697491	PFF0815w	malate%3Aquinone oxidoreductase putative	-0.591
chr6	699249	703214	PFF0820w	hypothetical protein conserved	0.264
chr6	704032	705165	PFF0825c	mitochondrial import receptor subunit tom40	-0.797
chr6	710056	714573	PFF0830w	alpha adaptin-like protein putative	0.124
chr6	717047	718914	PFF0835w	hypothetical protein conserved	0.019
chr6	720532	721309	PFF0840w	hypothetical protein conserved	0.113
chr6	723114	731487	PFF0845c	erythrocyte membrane protein 1 %28PfEMP1%29	4.823
chr6	735365	736322	PFF0847w	Stevor pseudogene	4.932
chr6	738256	739262	PFF0850c	stevor	4.783
chr6	741266	742403	PFF0855c	rifin	4.955

chr6	748413	748811	PFF0860c	histone h2a	0.949
chr6	754392	754802	PFF0865w	histone h3	-0.445
chr6	758606	760993	PFF0870w	hypothetical protein conserved	-0.059
chr6	761936	764167	PFF0875w	hypothetical protein conserved	0.074
chr6	764664	765489	PFF0880c	hypothetical protein conserved	-0.028
chr6	768789	769453	PFF0885w	60S ribosomal protein L27a putative	-0.478
chr6	770144	772503	PFF0890c	hypothetical membrane protein conserved	0.040
chr6	774526	775467	PFF0895w	malate dehydrogenase putative	-0.644
chr6	775977	777832	PFF0900c	rhomboid protease putative	0.006
chr6	778689	779633	PFF0905w	hypothetical protein conserved	0.130
chr6	779846	783293	PFF0910c	hypothetical protein conserved	0.257
chr6	785031	788024	PFF0915w	N-acetylglucosamine transferase	0.251
chr6	788220	795909	PFF0920c	hypothetical protein conserved	0.144
chr6	796700	798515	PFF0925w	hypothetical protein conserved	0.357
chr6	799394	802786	PFF0930w	hypothetical protein conserved	0.167
chr6	803371	814549	PFF0935c	hypothetical protein conserved	0.051
chr6	815619	818244	PFF0940c	cell division cycle protein 48 homologue putative	-0.723
chr6	820066	824244	PFF0945c	bi-functional enzyme PfACS10%3A long-chain fatty- acic	-0.068
chr6	825154	827769	PFF0950w	hypothetical protein conserved	0.123
chr6	828196	828991	PFF0955c	hypothetical protein conserved	-0.312
chr6	829136	830386	PFF0960c	hypothetical protein conserved	0.894
chr6	831283	839030	PFF0965c	hypothetical membrane protein conserved	0.195
chr6	840520	841221	PFF0970w	splicing factor 3a subunit putative	-0.457
chr6	841843	843360	PFF0975c	hypothetical protein conserved	-0.189
chr6	845657	846154	PFF0980w	hypothetical protein conserved	-0.138
chr6	846520	847491	PFF0985c	hypothetical protein conserved	0.277
chr6	848402	850861	PFF0990c	hypothetical protein conserved	0.619
chr6	851375	852952	PFF0995c	Merozoite surface protein 10 MSP10	-0.127
chr6	855157	857109	PFF1000w	cleavage stimulation factor subunit 1- like protein putativ	-0.163
chr6	857723	858607	PFF1005w	hypothetical protein conserved	0.352
chr6	859023	860111	PFF1010c	hypothetical protein conserved	0.163
chr6	862879	865413	PFF1015w	hypothetical protein conserved	0.086
chr6	865744	869906	PFF1020c	hypothetical protein conserved	-0.310
chr6	871294	872199	PFF1025c	pyridoxine biosynthetic enzyme pdx1 homologue putativ	-0.543
chr6	873807	876188	PFF1030w	hypothetical protein conserved	0.100

chr6	878788	880782	PFF1035w	Pfs77 protein	0.082
chr6	881961	882992	PFF1040w	hypothetical protein conserved	0.265
chr6	884298	889612	PFF1045w	hypothetical protein conserved	0.205
chr6	891995	892775	PFF1050w	nascent polypeptide associated complex alpha chain pu	-0.150
chr6	893448	895778	PFF1055c	hypothetical protein conserved	0.020
chr6	896987	897966	PFF1060w	hypothetical protein conserved	0.118
chr6	898339	902361	PFF1065c	hypothetical protein conserved	0.188
chr6	903738	906320	PFF1070c	hypothetical protein conserved	-0.346
chr6	908015	911094	PFF1075w	hypothetical protein conserved	-0.142
chr6	911501	911887	PFF1080w	hypothetical protein conserved	-0.004
chr6	912404	913708	PFF1085c	conserved hypothetical protein expressed transcript	-0.035
chr6	914663	915427	PFF1085a	conserved Plasmodium protein unknown function	0.299
chr6	915998	917297	PFF1090c	hypothetical membrane protein conserved	0.107
chr6	919683	924026	PFF1095w	leucyl-tRNA synthetase cytoplasmic putative	-0.316
chr6	925103	931277	PFF1100c	hypothetical protein conserved	-0.001
chr6	937066	938649	PFF1105c	chorismate synthase	-0.447
chr6	939658	943720	PFF1110c	hypothetical protein conserved	-0.064
chr6	946130	947245	PFF1115w	ferredoxin--NADP reductase putative	-0.424
chr6	947597	948645	PFF1120c	hypothetical protein conserved	-0.282
chr6	950224	951507	PFF1125c	RNA-binding protein mei2 homologue putative	-0.019
chr6	952907	954849	PFF1130c	superoxide dismutase PfSOD2	0.163
chr6	956766	958757	PFF1135w	transcription or splicing factor-like protein putative	-0.417
chr6	959133	962546	PFF1140c	ATP-dependent DEAD box helicase putative	-0.262
chr6	963742	970956	PFF1145c	protein kinase putative	-0.049
chr6	977184	978050	PFF1150w	ribonuclease H1 large subunit putative	-0.423
chr6	981067	982548	PFF1155w	hexokinase	-0.630
chr6	983947	984714	PFF1160w	hypothetical protein conserved	-0.326
chr6	985239	986871	PFF1165c	hypothetical protein conserved	-0.224
chr6	987809	989648	PFF1170w	hypothetical protein conserved	-0.029
chr6	990376	992199	PFF1175c	hypothetical protein conserved	0.288
chr6	994285	994554	PFF1180w	anaphase-promoting complex subunit putative	-0.441
chr6	999525	1007684	PFF1185w	iswi protein homologue	-0.128
chr6	1009727	1010975	PFF1190c	N-acetylglucosaminyl- phosphatidylinositol de-n-acetylas	-0.328
chr6	1011430	1014570	PFF1195c	hypothetical protein conserved	0.127
chr6	1015869	1017031	PFF1200w	hypothetical protein conserved	-0.217

chr6	1017669	1018445	PFF1205w	hypothetical protein conserved	0.266
chr6	1020025	1021876	PFF1210w	hypothetical membrane protein conserved	-0.109
chr6	1022423	1024215	PFF1215w	hypothetical membrane protein conserved	0.034
chr6	1025180	1026337	PFF1220w	hypothetical protein conserved	0.128
chr6	1026824	1031158	PFF1225c	DNA polymerase 1 putative	0.004
chr6	1032537	1033775	PFF1230c	hypothetical protein conserved	-0.087
chr6	1035359	1036372	PFF1235w	hypothetical protein conserved	0.177
chr6	1039656	1041551	PFF1240w	poly%28A%29 polymerase PAP putative	-0.174
chr6	1042653	1042949	PFF1245c	hypothetical protein conserved	-0.379
chr6	1043547	1045475	PFF1250w	hypothetical protein conserved	0.125
chr6	1046987	1048053	PFF1255w	hypothetical protein conserved	-0.084
chr6	1048363	1053966	PFF1260c	hypothetical protein conserved	0.162
chr6	1056053	1058530	PFF1265w	oxidoreductase short-chain dehydrogenase family putat	-0.034
chr6	1058667	1059923	PFF1270c	hypothetical protein conserved	0.240
chr6	1061116	1062755	PFF1275c	3-oxoacyl-%28acyl-carrier-protein%29 synthase i%2Fii	-0.668
chr6	1065148	1068627	PFF1280w	hypothetical protein conserved	-0.229
chr6	1069963	1072806	PFF1285w	hypothetical protein conserved	0.313
chr6	1073459	1073896	PFF1290c	hypothetical protein conserved	-0.257
chr6	1075019	1076137	PFF1295w	hypothetical protein conserved	-0.289
chr6	1078996	1081321	PFF1300w	pyruvate kinase putative	-0.465
chr6	1082911	1084029	PFF1305w	hypothetical protein conserved	0.027
chr6	1084509	1084841	PFF1310c	hypothetical protein conserved	0.319
chr6	1089191	1094329	PFF1315w	hypothetical protein with ankyrin repeats	-0.360
chr6	1095126	1096374	PFF1320c	troponin c-like protein putative	-0.054
chr6	1097301	1098650	PFF1325c	c3h4-type ring finger protein putative	-0.271
chr6	1100278	1100859	PFF1330c	mitochondrial import inner membrane translocase subun	-0.082
chr6	1101735	1102853	PFF1335c	4-methyl-5%28B-hydroxyethyl%29-thiazol monophospha	0.142
chr6	1104016	1105200	PFF1340w	hypothetical protein conserved	-0.131
chr6	1108198	1111641	PFF1345w	transportin	-0.257
chr6	1114545	1117538	PFF1350c	acetyl-coenzyme a synthetase	-0.399
chr6	1119588	1120859	PFF1355w	hypothetical protein conserved	-0.071
chr6	1121691	1122212	PFF1360w	6-pyruvoyl tetrahydropterin synthase putative	-0.364
chr6	1122808	1153665	PFF1365c	HECT-domain %28ubiquitin-transferase%29 putative	-0.240
chr6	1159863	1169081	PFF1370w	P. falciparum PK4 protein kinase	-0.082
chr6	1170473	1173697	PFF1375c	ethanolaminephosphotransferase putative	0.089

chr6	1176266	1178585	PFF1377w	hypothetical membrane protein conserved	0.101
chr6	1179054	1180004	PFF1380c	hypothetical protein conserved	0.253
chr6	1180574	1182354	PFF1385c	hypothetical protein conserved	0.075
chr6	1183897	1185618	PFF1390w	hypothetical protein conserved	0.186
chr6	1185997	1188579	PFF1395c	glutamyl-tRNA%28Gln%29 amidotransferase subunit B	0.064
chr6	1189620	1193979	PFF1400w	RAP protein putative	0.009
chr6	1194305	1195180	PFF1405c	hypothetical protein conserved	0.511
chr6	1196042	1198039	PFF1410c	hypothetical protein conserved	-0.333
chr6	1199854	1200996	PFF1415c	DNAJ domain protein putative	-0.085
chr6	1205191	1207782	PFF1420w	phosphatidylcholine-sterol acyltransferase precursor put	-0.479
chr6	1210422	1212764	PFF1425w	RNA binding protein putative	-0.344
chr6	1213950	1216007	PFF1430c	amino acid transporter	-0.124
chr6	1218309	1219253	PFF1435w	hypothetical protein conserved	0.131
chr6	1221943	1242925	PFF1440w	SET-domain protein putative	-0.060
chr6	1243583	1247222	PFF1445c	cullin-like protein putative	0.137
chr6	1250415	1251654	PFF1450w	sec14-like cytosolic factor or phosphatidylinositol%2Fphc	-0.274
chr6	1252055	1253988	PFF1455c	hypothetical protein conserved	-0.014
chr6	1254910	1256943	PFF1460c	hypothetical protein conserved	-0.036
chr6	1258883	1260017	PFF1465w	hypothetical protein conserved	-0.012
chr6	1260401	1269599	PFF1470c	DNA polymerase epsilon catalytic subunit a putative	0.026
chr6	1272366	1273535	PFF1475c	hypothetical protein conserved	0.044
chr6	1276332	1277828	PFF1480w	microtubule-associated protein ytm1 homologue putative	0.065
chr6	1279823	1283343	PFF1485w	hypothetical protein conserved	-0.011
chr6	1285061	1287034	PFF1490w	hypothetical protein conserved	0.232
chr6	1288240	1290808	PFF1495w	hypothetical protein conserved	0.315
chr6	1291038	1292975	PFF1500c	DEAD%2FDEAH box ATP-dependent RNA helicase put	-0.085
chr6	1295477	1296334	PFF1505w	hypothetical protein conserved	1.986
chr6	1313711	1315256	PFF1515c	erythrocyte membrane protein 1%28PfEMP1%29 pseud	4.073
chr6	1318330	1319119	PFF1525c	hypothetical protein conserved in P. falciparum	4.960
chr6	1329498	1330966	PFF1545w	rifin	4.717
chr6	1333037	1334038	PFF1550w	stevor	5.048
chr6	1336149	1337476	PFF1555w	rifin	4.970
chr6	1338221	1339420	PFF1560c	rifin	5.430
chr6	1341310	1342598	PFF1565c	rifin	5.319
chr6	1347344	1348574	PFF1570w	rifin	4.672

chr6	1350727	1352090	PFF1575w	rifin	4.072
chr6	1353948	1366431	PFF1580c	erythrocyte membrane protein 1 %28PfEMP1%29	4.899
chr6	1367913	1369105	PFF1585w	rifin pseudogene	4.933
chr6	1371502	1372862	PFF1590w	rifin	4.325
chr6	1374798	1382628	PFF1595c	erythrocyte membrane protein 1 %28PfEMP1%29	4.826
chr7	126425	127236	MAL7P1.5	Plasmodium falciparum Maurer%27s Cleft 2 transmembr	4.397
chr7	131294	132431	PF07_0003	rifin	5.194
chr7	135266	138319	PF07_0004	hypothetical protein	4.437
chr7	140158	141050	MAL7P1.6	hypothetical protein conserved in P.falciparum	1.296
chr7	144051	146252	MAL7P1.7	RESA-like protein	0.958
chr7	147233	148507	PF07_0005	lysophospholipases-like protein putative	0.389
chr7	152127	154096	PF07_0006	starp antigen	0.049
chr7	157060	157651	PF07_0007	hypothetical protein conserved	0.322
chr7	160323	161355	PF07_0008	hypothetical protein	-0.340
chr7	165282	165545	PF07_0009	chitinase precursor fragment truncated	-0.534
chr7	167416	167499	MAL7_tRNA_Ty	tRNA Tyrosine	1.930
chr7	168665	169315	MAL7P1.10	hypothetical protein conserved	0.259
chr7	170606	176137	PF07_0010	hypothetical protein conserved	0.156
chr7	176777	177319	PF07_0011	hypothetical protein conserved	0.147
chr7	177876	182498	PF07_0012	hypothetical protein conserved	-0.025
chr7	183912	184352	MAL7P1.11	hypothetical protein conserved	-0.560
chr7	184742	187204	PF07_0013	hypothetical protein conserved	-0.236
chr7	190978	197882	MAL7P1.12	erythrocyte membrane-associated antigen	-0.046
chr7	199071	201329	MAL7P1.13	hypothetical protein conserved	0.173
chr7	202021	202770	MAL7P1.14	hypothetical protein conserved	0.066
chr7	205180	208356	PF07_0014	hypothetical protein conserved	-0.146
chr7	209079	221771	MAL7P1.15	hypothetical membrane protein conserved	0.015
chr7	222864	232670	MAL7P1.16	hypothetical membrane protein conserved	0.101
chr7	235411	246090	MAL7P1.17	hypothetical membrane protein conserved	-0.020
chr7	246684	250265	PF07_0015	hypothetical protein conserved	-0.122
chr7	253048	258627	PF07_0016	hypothetical protein conserved	-0.218
chr7	260097	263129	PF07_0017	hypothetical protein conserved	-0.294
chr7	264069	268880	MAL7P1.18	serine%2Fthreonine protein kinase putative	0.173
chr7	271348	284452	MAL7P1.19	ubiquitin-transferase putative	0.037
chr7	288781	292794	PF07_0018	hypothetical membrane protein conserved	0.313

chr7	293539	297432	PF07_0019	hypothetical protein conserved	-0.017
chr7	299278	300336	MAL7P1.20	peptide chain release factor putative	-0.058
chr7	301639	305532	PF07_0020	hypothetical protein conserved	0.005
chr7	307662	310631	PF07_0021	hypothetical protein conserved	-0.161
chr7	311466	316064	PF07_0022	hypothetical protein conserved	0.097
chr7	317358	319835	MAL7P1.21	origin recognition complex subunit putative	-0.102
chr7	320354	322819	PF07_0023	DNA replication licensing factor mcm7 homologue putati	-0.555
chr7	325403	334087	PF07_0024	inositol phosphatase putative	-0.112
chr7	403656	408461	MAL7P1.22	hypothetical protein conserved	0.232
chr7	409365	410099	PF07_0025	hypothetical protein conserved	0.060
chr7	413079	415964	PF07_0026	ubiquitin-protein ligase E3 putative	-0.296
chr7	417383	421157	MAL7P1.23	RAP protein putative	0.023
chr7	422307	423086	MAL7P1.24	hypothetical protein conserved	-0.067
chr7	424085	426809	MAL7P1.25	cytoskeleton associated protein putative	-0.132
chr7	428275	428484	PF07_0027	DNA-directed RNA polymerase 2 8.2 kDa polypeptide pu	-0.673
chr7	429621	432771	PF07_0028	hypothetical protein conserved	0.135
chr7	433409	434683	MAL7P1.26	O-sialoglycoprotein endopeptidase putative	-0.275
chr7	436970	439992	PF07_0029	heat shock protein 86	-0.277
chr7	440961	443699	PF07_0030	heat shock protein 86 family protein	0.167
chr7	444851	445743	PF07_0031	heat shock protein 86 family protein	-0.058
chr7	446880	447566	PF07_0032	Cg8 protein	-0.474
chr7	451089	453710	PF07_0033	Cg4 protein	-0.313
chr7	454668	455621	PF07_0034	Cg3 protein	-0.061
chr7	458600	461695	MAL7P1.27	chloroquine resistance transporter	-0.080
chr7	463593	467339	PF07_0035	cg1 protein	-0.187
chr7	467721	468539	PF07_0036	Cg6 protein	0.087
chr7	468938	477127	PF07_0037	Cg2 protein	0.032
chr7	478614	482612	PF07_0038	Cg7 protein	-0.009
chr7	484101	484724	PF07_0039	hypothetical protein conserved	-0.119
chr7	485382	489136	MAL7P1.28	ribonucleases p%2Fmrp protein subunit putative	0.046
chr7	490467	491573	PF07_0040	lysophospholipase-like protein putative	-0.436
chr7	493935	501259	MAL7P1.29	hypothetical protein conserved	-0.126
chr7	502893	513874	MAL7P1.30	hypothetical protein conserved	0.485
chr7	515435	516814	PF07_0041	hypothetical protein conserved	-0.004
chr7	518483	527215	PF07_0042	hypothetical protein conserved	0.181

chr7	527344	528919	MAL7P1.31	hypothetical membrane protein conserved	-0.026
chr7	529908	531098	MAL7P1.32	hypothetical protein conserved	-0.015
chr7	531494	532930	MAL7P1.33	hypothetical protein conserved	0.039
chr7	533846	534516	PF07_0043	60S ribosomal protein L34-a putative	-0.082
chr7	536747	537352	PF07_0044	hypothetical protein conserved	0.851
chr7	538079	539209	PF07_0045	hypothetical protein conserved	0.056
chr7	540216	541415	PF07_0046	50S ribosomal protein L1 putative	-0.159
chr7	542659	546348	PF07_0047	cell division cycle ATPase putative	-0.176
chr7	547897	548610	MAL7P1.34	hypothetical protein conserved	-0.001
chr7	550616	553618	MAL7P1.35	hypothetical protein conserved	0.404
chr7	554714	555073	MAL7P1.36	hypothetical protein conserved	0.071
chr7	555786	557972	MAL7P1.37	sin3 associated polypeptide p18-like protein	0.253
chr7	560623	562689	MAL7P1.38	regulator of chromosome condensation protein putative	1.248
chr7	567328	574803	PF07_0048	erythrocyte membrane protein 1 %28PfEMP1%29	4.915
chr7	580490	581698	MAL7P1.43	RIF pseudogene RIFIN pseudogene	4.961
chr7	582716	590509	PF07_0049	erythrocyte membrane protein 1 %28PfEMP1%29	4.761
chr7	595721	596947	MAL7P1.47	RIF pseudogene RIFIN pseudogene	4.977
chr7	598284	605898	MAL7P1.50	erythrocyte membrane protein 1 %28PfEMP1%29	4.838
chr7	607536	614456	PF07_0050	erythrocyte membrane protein 1 %28PfEMP1%29	4.969
chr7	618284	619538	MAL7P1.52	RIF pseudogene RIFIN pseudogene	5.099
chr7	622104	629686	PF07_0051	erythrocyte membrane protein 1 %28PfEMP1%29	4.925
chr7	636764	644301	MAL7P1.55	erythrocyte membrane protein 1 %28PfEMP1%29	5.066
chr7	645704	653111	MAL7P1.56	erythrocyte membrane protein 1 %28PfEMP1%29	4.777
chr7	657519	658759	MAL7P1.57	rifin	5.071
chr7	661031	661818	MAL7P1.58	Plasmodium falciparum Maurer%27s Cleft 2 transmembr	4.637
chr7	662489	663346	MAL7P1.59	hypothetical protein conserved in P. falciparum	4.478
chr7	664741	665900	MAL7P1.61	erythrocyte membrane protein 1 %28PfEMP1%29 pseud	3.467
chr7	667836	669118	MAL7P1.64	hypothetical membrane protein conserved	0.239
chr7	669763	672627	MAL7P1.65	hypothetical protein conserved	0.299
chr7	673311	676245	MAL7P1.66	hypothetical protein conserved	0.270
chr7	676841	678572	MAL7P1.67	hypothetical protein conserved	0.203
chr7	679757	680678	PF07_0052	hypothetical protein conserved	0.082
chr7	682445	693154	PF07_0053	hypothetical protein conserved	0.116
chr7	698478	698849	PF07_0054	histone h2b putative	0.099
chr7	702096	704144	PF07_0055	hypothetical protein conserved	0.325

chr7	706210	710295	PF07_0056	hypothetical protein conserved	-0.185
chr7	712247	717045	MAL7P1.68	Zinc finger putative	0.115
chr7	717821	718570	MAL7P1.69	calmodulin putative	-0.087
chr7	720332	721543	PF07_0057	transcription elongation factor s-ii putative	-0.066
chr7	723361	725088	PF07_0058	hypothetical protein conserved	0.445
chr7	728827	729795	PF07_0059	4-nitrophenylphosphatase putative	-0.038
chr7	729986	730506	PF07_0060	hypothetical protein conserved	-0.173
chr7	731437	738057	PF07_0061	hypothetical protein conserved	-0.147
chr7	739881	742067	MAL7P1.73	calcium%2Fcalmodulin-dependent protein kinase putativ	0.094
chr7	743310	744224	MAL7P1.74	hypothetical protein conserved	-0.049
chr7	744919	745433	MAL7P1.75	mitochondrial ATP synthase F1 epsilon subunit putative	-0.249
chr7	746444	748708	PF07_0062	GTP-binding translation elongation factor tu family protei	-0.568
chr7	749899	750258	PF07_0063	hypothetical protein conserved	-0.865
chr7	750976	752280	PF07_0064	drug%2Fmetabolite exporter drug%2Fmetabolite transp	-0.157
chr7	754615	756285	PF07_0065	zinc transporter putative	-0.584
chr7	758583	761960	PF07_0066	hypothetical protein conserved	-0.338
chr7	763075	766284	PF07_0067	hypothetical protein conserved	0.114
chr7	767845	770343	MAL7P1.76	hypothetical protein conserved	-0.059
chr7	772657	774344	MAL7P1.77	hypothetical protein conserved	-0.152
chr7	775386	776843	MAL7P1.78	hypothetical protein conserved	0.053
chr7	777093	780091	MAL7P1.79	hypothetical protein conserved	0.056
chr7	780672	782595	PF07_0068	cysteine desulfurase putative	-0.303
chr7	783485	786502	PF07_0069	hypothetical protein conserved	-0.148
chr7	787446	788872	MAL7P1.81	eukaryotictranslation initiation factor 3 37.28 kDa subunit	-0.575
chr7	791746	792714	PF07_0070	drug metabolite transporter	-0.077
chr7	793959	796585	MAL7P1.82	hypothetical membrane protein conserved	0.348
chr7	797386	800277	MAL7P1.83	hypothetical protein conserved	-0.027
chr7	800567	802039	MAL7P1.84	hypothetical protein expressed conserved	-0.192
chr7	803623	806075	MAL7P1.86	transcription initiation factor iie alpha subunit putative	0.000
chr7	807253	809508	PF07_0071	queuine tRNA-ribosyltransferase%3B putative	-0.359
chr7	811139	813072	PF07_0072	calcium-dependent protein kinase 4	-0.369
chr7	814096	818253	MAL7P1.87	hypothetical protein conserved	-0.130
chr7	820170	821789	PF07_0073	seryl-tRNA synthetase putative	-0.405
chr7	822733	827325	PF07_0074	hypothetical protein conserved	-0.194
chr7	828327	830640	MAL7P1.88	thioredoxin-like protein	0.109

chr7	832593	851013	MAL7P1.89	Dynein heavy chain putative	0.399	
chr7	851890	860115	MAL7P1.91	exported serine%2Fthreonine protein kinase	0.586	7cenL
chr7	867677	876011	MAL7P1.92	cysteine repeat modular protein 2 homologue	0.685	7cenR
chr7	877814	878391	MAL7P1.93	mitochondrial ribosomal protein S8 putative	-0.841	
chr7	879821	881260	MAL7P1.94	prefoldin subunit 3 putative	0.246	
chr7	882316	886681	MAL7P1.95	hypothetical protein conserved	0.139	
chr7	886925	888298	PF07_0075	hypothetical membrane protein conserved	-0.109	
chr7	889006	889810	PF07_0076	hypothetical protein conserved	0.762	
chr7	891333	892237	MAL7P1.97	hypothetical protein conserved	-0.342	
chr7	893511	893972	MAL7P1.98	hypothetical protein conserved	-0.311	
chr7	894366	895400	MAL7P1.99	hypothetical protein conserved	-0.250	
chr7	895859	898206	MAL7P1.100	serine%2Fthreonine protein kinase Pfnek-4	-0.185	
chr7	899871	902969	PF07_0077	actin-related protein	-0.149	
chr7	903774	908969	MAL7P1.102	hypothetical protein conserved	0.156	
chr7	910390	912030	PF07_0078	hypothetical protein conserved	-0.063	
chr7	914118	914868	PF07_0079	60S ribosomal protein L11a putative	-0.206	
chr7	916959	917863	PF07_0080	40S ribosomal protein S10 putative	-0.137	
chr7	918828	920189	PF07_0081	hypothetical protein conserved	0.165	
chr7	921303	931508	PF07_0082	hypothetical protein conserved	-0.093	
chr7	932171	932876	MAL7P1.104	3%27-5%27 exoribonuclease Csl4 homolog putative	-0.543	
chr7	933241	934008	PF07_0083	hypothetical protein conserved	-0.294	
chr7	934830	935322	PF07_0084	hypothetical protein conserved	-0.406	
chr7	935841	936239	MAL7P1.105	hypothetical protein conserved	-0.118	
chr7	938312	940364	PF07_0085	ferrodoxin reductase-like protein	-0.600	
chr7	941722	942863	MAL7P1.106	hypothetical protein conserved	0.085	
chr7	943192	944754	MAL7P1.107	hypothetical protein conserved	0.098	
chr7	947059	947552	MAL7P1.108	hypothetical protein conserved	0.148	
chr7	947898	954427	MAL7P1.109	hypothetical protein conserved	0.146	
chr7	955127	956513	MAL7P1.110	Ham1-like protein putative	-0.050	
chr7	956925	958163	MAL7P1.111	hypothetical protein conserved	-0.202	
chr7	959519	970047	PF07_0086	hypothetical protein conserved	0.024	
chr7	971810	972544	PF07_0087	hypothetical protein conserved	-0.202	
chr7	973334	976192	MAL7P1.112	hypothetical protein conserved	0.333	
chr7	977661	980360	MAL7P1.113	DEAD box helicase putative	-0.233	
chr7	982068	983404	MAL7P1.114	P36-like protein homologue putative	-0.608	

chr7	983890	986229	MAL7P1.115	hypothetical membrane protein conserved	0.354
chr7	986835	987540	PF07_0088	40S ribosomal protein S5 putative	-0.629
chr7	990873	992276	PF07_0089	hypothetical protein conserved	-0.225
chr7	993655	994271	MAL7P1.117	hypothetical protein conserved	-0.295
chr7	997152	997889	PF07_0090	hypothetical protein conserved	-0.371
chr7	999399	1000610	MAL7P1.118	PelOta protein homologue putative	-0.181
chr7	1001739	1003988	MAL7P1.119	hypothetical protein conserved	0.987
chr7	1004825	1008490	MAL7P1.120	hypothetical protein conserved	0.047
chr7	1010160	1011719	MAL7P1.122	conserved GTP-binding protein putative	-0.334
chr7	1012296	1013393	PF07_0091	cell cycle control protein cwf15 homologue	0.030
chr7	1014262	1016070	PF07_0092	hypothetical protein conserved	-0.289
chr7	1017407	1017823	PF07_0093	hypothetical protein conserved	-0.334
chr7	1018746	1020485	PF07_0094	hypothetical protein conserved	-0.315
chr7	1021040	1023158	MAL7P1.123	hypothetical protein conserved	0.061
chr7	1023880	1025111	PF07_0095	tRNAHis guanylyltransferase putative	-0.211
chr7	1025868	1027253	PF07_0096	hypothetical protein conserved	0.125
chr7	1028034	1029648	MAL7P1.124	hypothetical protein conserved	-0.270
chr7	1031142	1033784	MAL7P1.125	hypothetical protein conserved	-0.297
chr7	1034296	1038564	PF07_0097	hypothetical protein conserved	0.074
chr7	1040154	1040834	PF07_0098	dynactin 4 putative	-0.502
chr7	1041974	1042741	PF07_0099	hypothetical protein conserved	-0.162
chr7	1043521	1046619	PF07_0100	hypothetical protein conserved	-0.147
chr7	1048608	1055609	PF07_0101	hypothetical protein conserved	-0.097
chr7	1056077	1060122	MAL7P1.126	hypothetical protein conserved	-0.070
chr7	1061711	1067521	MAL7P1.127	hypothetical protein conserved	0.185
chr7	1068671	1071825	MAL7P1.129	hypothetical protein conserved	0.364
chr7	1074760	1076043	PF07_0102	hypothetical protein conserved	0.207
chr7	1076884	1078006	MAL7P1.130	3-demethylubiquinone-9 3-methyltransferase-like protein	-0.189
chr7	1079027	1079497	PF07_0103	chaperone putative	-0.373
chr7	1080048	1081543	MAL7P1.131	hypothetical protein conserved	0.185
chr7	1081820	1088453	MAL7P1.132	hypothetical protein conserved	0.078
chr7	1090345	1091008	MAL7P1.133	hypothetical protein conserved	0.039
chr7	1091185	1101485	MAL7P1.134	hypothetical protein conserved	0.176
chr7	1103088	1104738	MAL7P1.137	kelch protein putative	-0.290
chr7	1107841	1113534	PF07_0104	kinesin-like protein putative	-0.124

chr7	1114309	1118352	PF07_0105	exonuclease i putative	-0.056
chr7	1120628	1125616	MAL7P1.138	hypothetical membrane protein conserved	0.137
chr7	1126356	1127057	MAL7P1.139	mago nashi protein homolog putative	0.188
chr7	1127578	1130130	PF07_0106	hypothetical protein conserved	-0.186
chr7	1134391	1135431	MAL7P1.141	hypothetical protein conserved	0.321
chr7	1136018	1137719	MAL7P1.142	hypothetical protein conserved	0.534
chr7	1141224	1141588	MAL7_ITS1	MAL7_ITS1	0.647
chr7	1141946	1144480	MAL7_28S	MAL7_28S	1.006
chr7	1148942	1151498	PF07_0107	hypothetical protein	0.327
chr7	1155894	1158343	MAL7P1.144	protein kinase putative	-0.349
chr7	1159532	1163740	MAL7P1.145	mismatch repair protein pms1 homologue putative	-0.141
chr7	1164207	1179868	MAL7P1.146	hypothetical protein conserved	0.274
chr7	1181932	1191953	MAL7P1.147	ubiquitin carboxyl-terminal hydrolase putative	-0.228
chr7	1195855	1198130	PF07_0108	hypothetical protein conserved	-0.224
chr7	1198679	1199191	PF07_0109	hypothetical protein conserved	-0.083
chr7	1201024	1202583	PF07_0110	hypothetical protein conserved	-0.210
chr7	1202946	1206434	PF07_0111	hypothetical protein conserved	-0.022
chr7	1207531	1210731	MAL7P1.149	hypothetical protein conserved	-0.149
chr7	1212124	1213785	MAL7P1.150	cysteine desulfurase putative	-0.250
chr7	1215076	1217196	MAL7P1.151	modification methylase-like protein putative	-0.092
chr7	1218086	1219207	PF07_0112	proteasome subunit alpha type 5 putative	-0.884
chr7	1219831	1221954	PF07_0113	hypothetical protein conserved	0.157
chr7	1222817	1225780	MAL7P1.152	hypothetical protein conserved	0.029
chr7	1226662	1229532	PF07_0114	hypothetical protein conserved	0.108
chr7	1231320	1237076	PF07_0115	cation transporting ATPase cation transporter	-0.263
chr7	1239777	1245548	PF07_0116	hypothetical protein conserved	0.085
chr7	1246555	1247665	PF07_0117	eukaryotictranslation initiation factor 2 alpha subunit puta	-0.730
chr7	1248992	1265677	PF07_0118	hypothetical protein conserved	0.165
chr7	1267033	1268893	MAL7P1.153	hypothetical protein conserved	-0.120
chr7	1269217	1269852	PF07_0119	hypothetical protein conserved	-0.645
chr7	1270550	1271139	MAL7P1.154	hypothetical protein conserved	0.171
chr7	1271464	1272577	MAL7P1.154a	hypothetical protein conserved	0.205
chr7	1273802	1280849	MAL7P1.155	hypothetical protein conserved	-0.081
chr7	1281405	1284090	MAL7P1.156	hypothetical protein conserved	0.040
chr7	1285965	1287556	MAL7P1.157	hypothetical protein conserved	-0.160

chr7	1287648	1290048	MAL7P1.157a	hypothetical protein	-0.011
chr7	1291238	1292083	MAL7P1.158	signal recognition particle putative	0.174
chr7	1293822	1301564	PF07_0120	hypothetical protein conserved	-0.122
chr7	1302774	1303640	MAL7P1.159	antioxidant protein putative	-0.143
chr7	1304213	1306879	PF07_0121	NMD3 protein putative	0.148
chr7	1308223	1309473	PF07_0122	BRIX domain putative	-0.195
chr7	1310747	1313029	PF07_0123	mRNA %28N6-adenosine%29-methyltransferase putativ	-0.085
chr7	1314163	1315106	MAL7P1.160	hypothetical protein conserved	0.046
chr7	1315530	1318156	PF07_0124	hypothetical protein conserved	0.304
chr7	1319821	1320114	MAL7P1.161	dynein light chain putative	0.211
chr7	1321286	1336243	MAL7P1.162	dynein heavy chain putative	3.624
chr7	1337161	1337698	MAL7P1.163	hypothetical protein conserved	-0.171
chr7	1338289	1340895	PF07_0125	hypothetical protein conserved	0.225
chr7	1341433	1344600	MAL7P1.164	adapter-related protein putative	0.060
chr7	1352835	1356830	PF07_0126	hypothetical protein conserved	-0.019
chr7	1359723	1360472	PF07_0127	hypothetical protein conserved	-0.004
chr7	1361270	1369591	MAL7P1.167	hypothetical protein conserved	0.249
chr7	1374387	1374458	MAL7_tRNA_Th	tRNA Threonine	2.535
chr7	1376682	1377743	MAL7P1.170	ring stage expressed protein	0.092
chr7	1380751	1387189	MAL7P1.171	hypothetical protein	-0.255
chr7	1394335	1397166	MAL7P1.172	hypothetical protein conserved	-0.378
chr7	1398218	1399372	MAL7P1.173	hypothetical protein	0.254
chr7	1405408	1406525	MAL7P1.174	hypothetical protein conserved in P.falciparum	0.118
chr7	1409134	1410858	MAL7P1.175	protein kinase putative pseudogene	-0.068
chr7	1413431	1417944	MAL7P1.176	erythrocyte binding antigen	0.044
chr7	1423537	1425972	PF07_0129	acyl-coA synthetase PfACS5	0.529
chr7	1427439	1428136	MAL7P1.177	predicted integral membrane protein conserved in P. falc	0.251
chr7	1434304	1436145	MAL7P1.178	hypothetical protein conserved in P.falciparum	2.821
chr7	1437788	1438902	MAL7P1.179	MAL7P1.179	4.807
chr7	1441011	1442002	PF07_0130	stevor	5.322
chr7	1443129	1444413	MAL7P1.183	erythrocyte membrane protein 1 %28PfEMP1%29 pseud	5.553
chr7	1446087	1447419	PF07_0132	rifin	5.232
chr7	1451813	1453015	MAL7P1.184	rifin	5.157
chr7	1454828	1456058	PF07_0134	rifin	5.002
chr7	1457999	1459354	MAL7P1.185	rifin	5.011

chr7	1469810	1471059	PF07_0138	rifin	4.481
chr7	1472964	1481610	MAL7P1.187	erythrocyte membrane protein 1 %28PfEMP1%29	4.793
chr8	22369	29661	PF08_0142	erythrocyte membrane protein 1 %28PfEMP1%29	4.653
chr8	30708	40041	PF08_0141	erythrocyte membrane protein 1 %28PfEMP1%29	4.807
chr8	41956	51947	PF08_0140	erythrocyte membrane protein 1 %28PfEMP1%29	5.072
chr8	57476	58827	PF08_0138	rifin	5.041
chr8	62262	63259	MAL8P1.163	hypothetical protein conserved in P.falciparum	3.022
chr8	66840	72909	MAL8P1.162	surface-associated interspersed gene 8.3 %28SURFIN8.	3.272
chr8	80947	81766	MAL8P1.161	hypothetical protein conserved in P.falciparum	2.157
chr8	83961	85028	MAL8P1.160	hypothetical protein conserved in P.falciparum	2.263
chr8	85692	89546	PF08_0137	hypothetical protein conserved	0.637
chr8	92521	98695	MAL8b_28s	MAL8b_28s	1.251
chr8	100014	100175	PF08_tmp2	PF08_tmp2	1.400
chr8	103078	103950	PF08_0136b	von willebrand factor a-domain-related protein putative	1.687
chr8	105649	106012	PF08_0136	hypothetical protein	0.688
chr8	107054	107293	MAL8P1.158	hypothetical protein	-0.157
chr8	107716	109365	PF08_0135	hypothetical protein conserved	-0.036
chr8	110303	110680	PF08_0134	hypothetical protein conserved	-0.165
chr8	113422	118751	MAL8P1.157	ubiquitin-like protease 1 homolog Ulp1 homolog putativ	0.152
chr8	120155	123190	MAL8P1.156	mannose-6-phosphate isomerase putative	0.145
chr8	125998	127290	MAL8P1.155	hypothetical protein	0.441
chr8	129972	143960	MAL8P1.154	hypothetical protein conserved	0.680
chr8	147210	151403	PF08_0132	glutamate dehydrogenase putative	-0.433
chr8	152929	160789	MAL8P1.153	hypothetical protein conserved	-0.228
chr8	166536	167198	PF08_0131	1-cys peroxidoxin	-0.693
chr8	168189	171554	PF08_0130	wd repeat protein putative	-0.317
chr8	172985	175505	MAL8P1.152	hypothetical protein conserved	-0.111
chr8	175767	180026	MAL8P1.151	inositol phosphatase putative	0.042
chr8	181683	189233	MAL8P1.150	hypothetical protein conserved	-0.239
chr8	189686	191436	MAL8P1.149	hypothetical protein conserved	0.047
chr8	193096	195223	PF08_0129	protein phosphatase putative	-0.392
chr8	196268	196798	MAL8P1.148	hypothetical protein conserved	0.000
chr8	197701	199734	PF08_0128	SYF2 splicing factor putative	-0.073
chr8	200301	204038	PF08_0127	hypothetical protein conserved	-0.117
chr8	205361	207781	MAL8P1.146	filament assembling protein putative	0.226

chr8	209571	211411	MAL8P1.145	hypothetical protein conserved	-0.159	
chr8	213372	217091	PF08_0126	DNA repair protein rad54 putative	-0.411	
chr8	219008	223411	MAL8P1.144	AAA family ATPase putative	-0.072	
chr8	224882	231329	MAL8P1.143	hypothetical protein conserved	0.056	
chr8	232518	233876	PF08_0125	tubulin gamma chain	-0.599	
chr8	234587	235546	MAL8P1.142	proteasome beta-subunit	-0.600	
chr8	238973	242168	PF08_0124	hypothetical protein conserved	0.161	
chr8	243223	245895	MAL8P1.141	hypothetical protein conserved	0.253	
chr8	246831	249053	MAL8P1.140	methionine aminopeptidase putative	0.041	
chr8	251203	269556	MAL8P1.139	folate%2Fbiopterin transporter	0.105	
chr8	270223	271815	PF08_0123	pseudouridylate synthase putative	0.441	
chr8	272536	278529	PF08_0122	hypothetical protein	0.193	
chr8	279497	280150	PF08_0121	peptidyl-prolyl cis-trans isomerase precursor	-0.185	
chr8	280827	282302	PF08_0120	GTPase-activating protein putative	-0.204	
chr8	284172	285638	MAL8P1.138	hypothetical protein conserved	0.076	
chr8	288075	289736	MAL8P1.137	hypothetical protein conserved	-0.203	
chr8	292018	292299	PF08_0119	hypothetical protein conserved	-0.595	
chr8	294229	297987	PF08_0118	hypothetical protein conserved	0.763	8cenL
chr8	323495	326686	MAL8P1.136	hypothetical protein conserved	-0.014	
chr8	327521	331132	PF08_0117	hypothetical protein conserved	0.091	
chr8	331635	333806	PF08_0116	hypothetical protein conserved	0.078	
chr8	334858	338855	MAL8P1.135	hypothetical membrane protein conserved	-0.079	
chr8	339030	344382	MAL8P1.134	hypothetical protein conserved	-0.472	
chr8	345416	345949	MAL8P1.133	glycosyltransferase family 28 protein putative	-0.334	
chr8	346555	348582	PF08_0115	DnaJ protein putative	-0.471	
chr8	350439	353938	MAL8P1.132	kinesin-like protein putative	-0.175	
chr8	355593	363517	PF08_0114	hypothetical protein conserved	0.112	
chr8	364381	367542	PF08_0113	vacuolar proton-translocating ATPase subunit A putative	-0.390	
chr8	369728	370804	PF08_0112	hypothetical protein conserved	-0.048	
chr8	372043	373744	MAL8P1.131	Gas41 homologue putative	0.012	
chr8	374023	377988	PF08_0111	RNA helicase putative	-0.023	
chr8	379145	380667	MAL8P1.130	hypothetical membrane protein putative	0.089	
chr8	382685	383968	PF08_0110	PfRab18 GTPase	0.127	
chr8	385418	386917	MAL8P1.300	hypothetical protein conserved	0.158	
chr8	388339	389484	MAL8P1.128	proteasome subunit alpha putative	-0.265	

chr8	390879	395381	MAL8P1.127	hypothetical protein conserved	-0.189
chr8	396770	399909	MAL8P1.126	serine protease putative	-0.242
chr8	400713	402482	PF08_0109	hypothetical protein conserved	-0.304
chr8	403717	405263	MAL8P1.125	tyrosyl-tRNA synthetase putative	-0.633
chr8	406118	410206	MAL8P1.124	hypothetical protein conserved	0.205
chr8	411058	416290	MAL8P1.123	hypothetical protein conserved	0.154
chr8	417466	419187	PF08_0108	pepsinogen putative	-0.392
chr8	422615	423762	MAL8P1.122	hypothetical protein conserved	-0.108
chr8	424318	425658	MAL8P1.121	hypothetical protein conserved	0.321
chr8	432287	440173	PF08_0107	erythrocyte membrane protein 1 %28PfEMP1%29	4.905
chr8	441530	449184	PF08_0106	erythrocyte membrane protein 1 %28PfEMP1%29	4.933
chr8	451736	453020	PF08_0105	rifin	4.759
chr8	454924	456040	PF08_0104	rifin	4.447
chr8	460433	467688	PF08_0103	erythrocyte membrane protein 1 %28PfEMP1%29	4.610
chr8	469064	473014	PF08_0102	asparagine-rich antigen Pfa55-14	0.073
chr8	476694	479246	PF08_0101	hypothetical protein conserved	0.198
chr8	479633	479920	MAL8P1.114	hypothetical protein conserved	-0.305
chr8	481101	498503	MAL8P1.113	Peptidase family C50 putative	0.155
chr8	500584	502690	PF08_0100	ruvB-like DNA helicase putative	-0.101
chr8	503459	507819	MAL8P1.112	hypothetical protein conserved	0.413
chr8	509345	513549	MAL8P1.111	hypothetical protein conserved	-0.116
chr8	518542	518814	PF08_0099	Acyl CoA binding protein putative	-0.184
chr8	520452	520858	MAL8P1.110	plastid 50S ribosomal protein L33 putative	-0.022
chr8	522308	525130	PF08_0098	protein kinase putative	-0.062
chr8	526179	528384	MAL8P1.109	Protein phosphatase 2C putative	-0.110
chr8	533536	535152	PF08_0097	hypothetical membrane protein conserved	0.102
chr8	535590	537740	MAL8P1.108	protein phosphatase putative	0.009
chr8	543092	545917	PF08_0096	RNA helicase putative	-0.424
chr8	547069	548486	MAL8P1.107	hypothetical protein conserved	0.472
chr8	549321	551737	PF08_0095	dihydropteroate synthetase	-0.079
chr8	552320	553549	MAL8P1.106	hypothetical protein conserved	0.114
chr8	555558	557972	PF08_0094	cullin-like protein putative	0.126
chr8	559005	560627	PF08_0093	hypothetical protein conserved	0.138
chr8	561865	565397	MAL8P1.105	hypothetical protein conserved	0.280
chr8	568944	574268	MAL8P1.104	CAF1 family ribonuclease putative	-0.281

chr8	577418	579490	MAL8P1.103	hypothetical protein conserved	-0.391
chr8	580603	583341	PF08_0092	hypothetical protein conserved	0.142
chr8	584103	588073	PF08_0091	hypothetical protein conserved	-0.066
chr8	590941	594609	PF08_0090	hypothetical protein conserved	0.391
chr8	594769	596532	MAL8P1.102	hypothetical protein conserved	0.396
chr8	597603	601264	MAL8P1.101	hypothetical protein conserved	-0.019
chr8	601645	603367	MAL8P1.100	hypothetical protein conserved	0.017
chr8	606043	613998	PF08_0089	hypothetical protein conserved	-0.050
chr8	614445	615776	MAL8P1.99	GTPase putative	-0.001
chr8	616354	618842	MAL8P1.98	serine protease putative	0.060
chr8	619963	621348	PF08_0088	hypothetical protein conserved	-0.028
chr8	622925	624562	PF08_0087	importin alpha putative	-0.633
chr8	627189	630065	PF08_0086	RNA-binding protein putative	-0.161
chr8	632704	633349	PF08_0085	ubiquitin-conjugating enzyme putative	-0.223
chr8	635110	635817	PF08_0084	RNA-binding protein %28U1 snRNP-like%29 putative	-0.174
chr8	637748	642578	MAL8P1.97	hypothetical membrane protein	0.057
chr8	644206	646005	PF08_0083	hypothetical protein conserved	-0.060
chr8	648437	650425	PF08_0082	hypothetical protein conserved	-0.195
chr8	652538	653925	MAL8P1.96	CS-domain containing protein putative	-0.270
chr8	657400	658751	MAL8P1.95	hypothetical protein conserved	0.088
chr8	659614	660639	MAL8P1.94	hypothetical protein	0.372
chr8	661275	663093	PF08_0081	hypothetical protein conserved	0.009
chr8	666669	668216	PF08_0080	hypothetical protein conserved	0.353
chr8	670662	671855	PF08_0079	Translation initiation factor SUI1 putative	-0.094
chr8	672278	676537	PF08_0078	hypothetical protein conserved	0.341
chr8	677512	678585	PF08_0077	GDP-mannose 4 6-dehydratase putative	-0.471
chr8	679110	679846	PF08_0076	40S ribosomal protein S16 putative	-0.364
chr8	682078	682930	PF08_0075	60S ribosomal protein L13 putative	-0.055
chr8	683386	684693	MAL8P1.93	hypothetical protein conserved	0.213
chr8	688461	689207	PF08_0074	DNA%2FRNA-binding protein Alba putative	-0.329
chr8	691458	695264	MAL8P1.92	ATPase putative	-0.127
chr8	696336	698643	MAL8P1.91	hypothetical protein conserved	-0.029
chr8	701035	701160	MAL8P1.90	hypothetical protein	0.474
chr8	701497	703773	MAL8P1.88	hypothetical protein conserved	0.153
chr8	704623	705804	PF08_0073	hypothetical protein conserved	-0.469

chr8	707197	708117	PF08_0072	hypothetical protein conserved	-0.023
chr8	708185	708400	MAL8P1.87	hypothetical protein	0.203
chr8	709349	709945	PF08_0071	Fe-superoxide dismutase	-1.050
chr8	710666	711721	MAL8P1.86	hypothetical protein conserved	0.278
chr8	712360	715287	PF08_0070	RAP protein putative	-0.143
chr8	718234	720867	PF08_0069	importin beta putative	-0.336
chr8	723049	726597	PF08_0068	hypothetical protein conserved	0.058
chr8	726902	727623	MAL8P1.85	hypothetical protein conserved	-0.250
chr8	728783	730849	MAL8P1.84	hypothetical protein conserved	-0.109
chr8	731038	733308	MAL8P1.83	eukaryotictranslation initiation factor putative	0.007
chr8	734600	735694	PF08_0067	hypothetical protein conserved	0.467
chr8	736801	742302	MAL8P1.82	hypothetical protein conserved	-0.166
chr8	744628	746628	PF08_0066	lipoamide dehydrogenase putative	-0.385
chr8	747136	748611	PF08_0065	WD repeat protein putative	-0.551
chr8	749776	750746	MAL8P1.81	flavoprotein putative	-0.321
chr8	751205	751834	PF08_0064	hypothetical protein conserved	-0.238
chr8	753065	757296	MAL8P1.80	hypothetical protein conserved	0.064
chr8	758347	759421	MAL8P1.79	hypothetical protein conserved	-0.435
chr8	761266	762159	MAL8P1.78	small heat shock protein putative	-0.369
chr8	763246	766458	PF08_0063	ClpB protein putative	-0.655
chr8	768239	769064	MAL8P1.77	hypothetical protein conserved	0.087
chr8	769045	770921	MAL8P1.76	meiotic recombination protein dmc1-like protein	-0.704
chr8	772075	772902	PF08_0062	Adenylate kinase	0.085
chr8	773697	774866	MAL8P1.75	Ubiquitin-activating enzyme putative	-0.172
chr8	775428	777920	MAL8P1.75a	GTPase putative	-0.117
chr8	778386	780776	PF08_0061	hypothetical protein conserved	0.106
chr8	784038	790745	PF08_0060	asparagine-rich antigen	-0.459
chr8	796757	798934	MAL8P1.74	hypothetical protein conserved	0.195
chr8	800563	801331	PF08_0059	protein kinase c inhibitor-like protein putative	-0.974
chr8	802046	805160	PF08_0058	hypothetical protein conserved	-0.558
chr8	806923	814591	MAL8P1.73	hypothetical protein conserved	0.013
chr8	816276	816577	PF08_0057	hypothetical protein conserved	-0.141
chr8	817929	818228	MAL8P1.72	high mobility group protein putative	-0.181
chr8	822460	823578	MAL8P1.71	hypothetical protein conserved	-0.583
chr8	825690	827252	MAL8P1.70	hypothetical protein conserved	-0.507

chr8	831139	832346	MAL8P1.69	14-3-3 protein homologue putative	-0.204
chr8	834318	836358	MAL8P1.68	hypothetical protein conserved	-0.025
chr8	837111	837981	MAL8P1.67	hypothetical protein conserved	-0.080
chr8	843415	844313	PF08_0056	zinc finger protein putative	0.065
chr8	846351	848478	MAL8P1.66	hypothetical protein conserved	-0.023
chr8	849567	854044	MAL8P1.65	DNA helicase putative	-0.305
chr8	856751	857839	PF08_0055	u3 small nucleolar ribonucleoprotein protein	-0.248
chr8	861481	863514	PF08_0054	heat shock 70 kDa protein	-0.510
chr8	864602	869234	MAL8P1.64	hypothetical protein conserved	-0.027
chr8	870659	871570	PF08_0053	BRIX domain putative	0.080
chr8	872507	874537	PF08_0052	hypothetical protein conserved	-0.313
chr8	875464	878595	PF08_0051	hypothetical protein conserved	0.172
chr8	879592	881556	PF08_0050	MAC%2Fperforin putative	-0.648
chr8	882687	885728	MAL8P1.63	hypothetical protein conserved	-0.095
chr8	886295	887233	MAL8P1.62	hypothetical protein conserved in other Plasmodium spe	-0.396
chr8	888957	891568	MAL8P1.61	hypothetical protein conserved	0.051
chr8	892755	899599	MAL8P1.60	hypothetical protein conserved	0.075
chr8	900100	900598	PF08_0049	ribonucleoprotein putative	-0.136
chr8	901838	908810	PF08_0048	ATP-dependent helicase putative	-0.216
chr8	910303	911812	MAL8P1.59	hypothetical protein conserved	-0.134
chr8	913360	915345	MAL8P1.58	phosphatidylglycerophosphate synthase	-0.073
chr8	920151	921992	PF08_0047	hypothetical protein conserved	0.047
chr8	923304	925028	PF08_0046	hypothetical protein conserved	0.035
chr8	926209	928767	MAL8P1.57	C-13 antigen	-0.352
chr8	930241	931957	MAL8P1.56	hypothetical protein conserved	-0.072
chr8	933417	936722	PF08_0045	2-oxoglutarate dehydrogenase e1 component	-0.581
chr8	937522	939972	MAL8P1.55	hypothetical protein conserved	0.144
chr8	940679	945741	MAL8P1.54	hypothetical protein conserved	0.179
chr8	949825	951974	MAL8P1.53	hypothetical protein conserved	-0.041
chr8	954523	957388	PF08_0044	Plasmodium falciparum protein kinase 1	-0.096
chr8	958572	959147	PF08_0043	hypothetical protein conserved	-0.132
chr8	959605	962814	PF08_0042	ATP-dependent RNA helicase prh1 putative	-0.163
chr8	966895	967730	MAL8P1.52	hypothetical protein conserved	-0.278
chr8	968974	969813	PF08_0041	ribosome biogenesis protein nep1 homologue putative	-0.057
chr8	970763	972787	PF08_0040	hypothetical protein conserved	-0.337

chr8	974659	975340	PF08_0039	ribosomal protein putative	0.167
chr8	977148	978156	MAL8P1.51	protein-transport protein sec61 beta 1 subunit putative	0.005
chr8	978506	979262	MAL8P1.50	hypothetical protein conserved	-0.188
chr8	979872	981708	PF08_0038	hypothetical protein conserved	-0.396
chr8	982092	982796	PF08_0037	hypothetical protein conserved	-0.361
chr8	983644	988240	MAL8P1.49	hypothetical membrane protein conserved	0.352
chr8	989354	989836	MAL8P1.48	small nuclear ribonucleoprotein putative	-0.040
chr8	991623	993264	MAL8P1.47	hypothetical protein conserved	0.366
chr8	993727	995037	MAL8P1.46	outer arm dynein light chain 2	-0.144
chr8	998127	1000601	PF08_0036	Pfsec23	-0.789
chr8	1002053	1004827	MAL8P1.45	hypothetical protein conserved	0.215
chr8	1005095	1005760	MAL8P1.44	hypothetical protein	0.000
chr8	1006579	1007882	MAL8P1.43	u5 snrnp-specific protein putative	-0.524
chr8	1008849	1012379	PF08_0035	hypothetical protein conserved	-0.234
chr8	1013999	1018448	MAL8P1.42	hypothetical protein conserved	0.006
chr8	1019161	1020340	MAL8P1.41	RWD domain-containing protein putative	-0.389
chr8	1023701	1024918	MAL8P1.40	RNA-binding protein putative	-0.637
chr8	1029824	1034586	PF08_0034	histone acetyltransferase Gcn5 putative	-0.233
chr8	1034637	1037500	MAL8P1.38	hypothetical protein conserved	-0.052
chr8	1038172	1039680	PF08_0033	hypothetical protein conserved	-0.441
chr8	1040979	1042220	MAL8P1.37	lipoate-protein ligase putative	-0.104
chr8	1042904	1043399	MAL8P1.36	hypothetical protein conserved	0.248
chr8	1044885	1046852	PF08_0032	DnaJ protein putative	0.156
chr8	1048623	1049579	PF08_0031	oxoglutarate%2Fmalate translocator protein putative	-0.652
chr8	1050354	1052777	PF08_0030	hypothetical protein conserved	-0.320
chr8	1053843	1055063	MAL8P1.35	exonuclease putative	-0.194
chr8	1056207	1060075	MAL8P1.34	hypothetical protein conserved	0.053
chr8	1060318	1062639	MAL8P1.33	GTP-binding protein putative	-0.136
chr8	1063376	1065133	MAL8P1.32	nucleoside transporter putative	-0.113
chr8	1067226	1068544	PF08_0029	hypothetical protein conserved	-0.161
chr8	1069635	1070857	MAL8P1.31	hypothetical protein conserved	0.012
chr8	1072069	1075489	MAL8P1.30	hypothetical membrane protein conserved	0.215
chr8	1076872	1080702	PF08_0028	hypothetical protein conserved	0.180
chr8	1081127	1084063	PF08_0027	hypothetical protein conserved	0.118
chr8	1087120	1092320	MAL8P1.29	hypothetical protein conserved	-0.021

chr8	1093070	1094293	MAL8P1.28	hypothetical protein conserved	0.103
chr8	1094762	1095561	MAL8P1.27	hypothetical protein conserved	0.078
chr8	1096188	1096634	MAL8P1.26	hypothetical protein conserved	-0.370
chr8	1097495	1098435	MAL8P1.25a	hypothetical protein conserved	-0.136
chr8	1099556	1101448	PF08_0026	hypothetical protein conserved	-0.034
chr8	1101888	1102145	PF08_0025	hypothetical protein conserved	-0.167
chr8	1102959	1103876	PF08_0024	hypothetical protein conserved	-0.077
chr8	1104555	1106180	MAL8P1.25	hypothetical protein conserved	-0.313
chr8	1107120	1109033	PF08_0023	hypothetical protein conserved	0.273
chr8	1110348	1113866	MAL8P1.24	hypothetical protein conserved	0.129
chr8	1114441	1140216	MAL8P1.23	ubiquitin-protein ligase 1 putative	-0.033
chr8	1143571	1145379	PF08_0022	hypothetical protein conserved	-0.001
chr8	1147239	1148576	PF08_0021	hypothetical protein conserved	-0.392
chr8	1149082	1150902	MAL8P1.22	dehydrodolichyl diphosphate synthetase putative	0.114
chr8	1153374	1157354	PF08_0020	Ubiquitination-mediated degradation component putative	-0.262
chr8	1158393	1159223	MAL8P1.21	SNARE protein	-0.096
chr8	1161236	1162780	PF08_0019	receptor for activated C kinase homolog PfRACK	-0.508
chr8	1165727	1168277	MAL8P1.20	hypothetical protein conserved	0.163
chr8	1168651	1172520	MAL8P1.19	RNA helicase putative	-0.195
chr8	1173466	1177659	PF08_0018	translation initiation factor-like protein	-0.007
chr8	1178382	1181396	PF08_0017	hypothetical protein conserved	-0.104
chr8	1182367	1184868	PF08_0016	hypothetical protein conserved	-0.295
chr8	1185667	1186323	PF08_0015	hypothetical protein conserved	-0.231
chr8	1187298	1188197	PF08_0014	plastid 50S ribosomal protein putative	0.102
chr8	1188695	1193997	MAL8P1.18	hypothetical protein conserved	-0.206
chr8	1194919	1197750	PF08_0013	hypothetical membrane protein conserved	-0.139
chr8	1199930	1207129	PF08_0012	SET domain protein putative	-0.181
chr8	1207860	1209466	MAL8P1.17	disulfide isomerase precursor putative	-0.648
chr8	1211269	1212709	MAL8P1.16	rhomboid protease putative	-0.522
chr8	1213409	1216651	MAL8P1.15	hypothetical protein conserved	0.109
chr8	1217343	1221788	PF08_0011	leucine - tRNA ligase	-0.090
chr8	1222097	1223755	PF08_0010	hypothetical protein conserved	0.138
chr8	1225166	1226935	MAL8P1.14	hypothetical protein conserved	-0.211
chr8	1227822	1229481	PF08_0009	translation initiation factor EIF-2b alpha subunit putative	0.021
chr8	1230971	1233204	MAL8P1.13	integral membrane protein conserved	-0.434

chr8	1234444	1237545	MAL8P1.12	hypothetical protein conserved	0.312
chr8	1238357	1240573	PF08_0008	hypothetical protein conserved	-0.149
chr8	1243038	1243967	PF08_0007	hypothetical protein conserved	-0.181
chr8	1246283	1252255	MAL8P1.11	hypothetical membrane protein conserved	0.025
chr8	1253421	1254867	MAL8P1.10	hypothetical protein conserved	-0.113
chr8	1255629	1256447	PF08_0006	prohibitin putative	-0.631
chr8	1257626	1258096	MAL8P1.9	u6 snRNA-associated sm-like protein putative	-0.456
chr8	1258562	1260112	MAL8P1.8	hypothetical protein conserved	-0.198
chr8	1261119	1267769	MAL8P1.7	hypothetical protein	0.100
chr8	1270206	1270718	MAL8P1.6	early transcribed membrane protein 8 ETRAMP 8	-0.542
chr8	1281075	1281185	MAL8a_5.8s	MAL8a_5.8s	0.354
chr8	1285649	1288826	PF08_tmp1	PF08_tmp1	0.398
chr8	1291311	1292477	PF08_0005	hypothetical protein conserved	-0.068
chr8	1295093	1295793	PF08_0004	hypothetical protein conserved	0.172
chr8	1297205	1299399	PF08_0003	tryptophan%2Fthreonine-rich antigen	-0.064
chr8	1304394	1305884	MAL8P1.4	hypothetical protein conserved	-0.033
chr8	1308124	1308995	MAL8P1.3	integral membrane protein conserved in P. falciparum	-0.110
chr8	1311708	1318026	PF08_0002	surface-associated interspersed gene 8.2 %28SURFIN8.	0.245
chr8	1318671	1319412	PF08_0001	hypothetical protein	-0.055
chr8	1322488	1323896	MAL8P1.2	hypothetical protein conserved in P.falciparum	0.126
chr8	1326215	1332258	MAL8P1.1	surface-associated interspersed gene 8.1 %28SURFIN8	1.617
chr8	1407868	1410774	MAL8P1.335	hypothetical protein conserved in P. falciparum	4.223
chr8	1412780	1413400	MAL8P1.330	hypothetical protein conserved in P.falciparum	4.391
chr9	4171	4620	PFI0002w	hypothetical proein conserved in P. falciparum	3.875
chr9	20080	27885	PFI0005w	erythrocyte membrane protein 1 %28PfEMP1%29	4.701
chr9	29797	31157	PFI0010c	rifin	3.763
chr9	33553	34795	PFI0015c	rifin	4.029
chr9	39554	40740	PFI0020w	rifin	3.859
chr9	42909	44045	PFI0025c	rifin	3.566
chr9	45902	47236	PFI0030c	rifin	4.698
chr9	49612	50971	PFI0035c	rifin	5.019
chr9	52648	54024	PFI0040c	VARC-like pseudogene	5.178
chr9	55074	56060	PFI0045c	stevor	5.446
chr9	58162	59240	PFI0050c	rifin	4.938
chr9	61342	62553	PFI0055c	rifin	5.119

chr9	64216	64428	PFI0060c	hypothetical protein conserved in P.falciparum	4.315
chr9	67334	68625	PFI0065w	rifin	5.265
chr9	70521	71738	PFI0070w	rifin	5.039
chr9	74409	75746	PFI0075w	rifin	5.125
chr9	77861	78874	PFI0080w	stevor	4.068
chr9	82002	82594	PFI0085c	hypothetical protein conserved in P.falciparum	0.180
chr9	84624	85574	PFI0086w	Plasmodium exported protein unknown function	0.341
chr9	86773	88074	PFI0090c	hypothetical protein	0.169
chr9	89360	91294	PFI0095c	protein kinase putative	-0.030
chr9	93829	95612	PFI0100c	protein kinase putative	-0.207
chr9	98401	100338	PFI0105c	protein kinase FIKK family	-0.170
chr9	102491	104592	PFI0110c	protein kinase putative	-0.135
chr9	106655	108405	PFI0115c	protein kinase putative	0.074
chr9	109333	111328	PFI0120c	protein kinase FIKK family	-0.114
chr9	113408	115548	PFI0125c	protein kinase FIKK family	0.079
chr9	119260	120748	PFI0130c	hypothetical protein conserved in P.falciparum	-0.048
chr9	121620	125005	PFI0135c	papain family cysteine protease putative	-0.262
chr9	127415	127723	PFI0140w	hypothetical protein conserved	-0.447
chr9	129223	129699	PFI0145w	hypothetical protein conserved	0.094
chr9	130139	130936	PFI0150c	hypothetical protein	0.110
chr9	133761	135586	PFI0155c	PfRab7 GTPase	0.143
chr9	140976	150587	PFI0160w	hypothetical protein conserved	-0.004
chr9	151699	159540	PFI0165c	DEAD%2FDEAH box helicase putative	-0.035
chr9	162768	166922	PFI0170w	hypothetical protein conserved	-0.219
chr9	168491	172885	PFI0175w	hypothetical protein conserved	-0.092
chr9	175540	177440	PFI0180w	alpha tubulin	-0.628
chr9	179185	183989	PFI0185w	hypothetical protein conserved	-0.033
chr9	185819	186381	PFI0190w	ribosomal protein L32 putative	-0.504
chr9	187340	188620	PFI0195c	hypothetical protein conserved	-0.163
chr9	191016	195825	PFI0200c	adapter-related protein putative	-0.202
chr9	197865	198814	PFI0205w	hypothetical protein conserved	0.129
chr9	199049	207456	PFI0210c	hypothetical membrane protein conserved	0.736
chr9	208166	208723	PFI0215c	signal peptidase putative	-0.329
chr9	210075	210607	PFI0220w	prefoldin subunit putative	-0.026
chr9	212513	217981	PFI0225w	Ubiquitin carboxyl-terminal hydrolases family 2 putative	0.023

chr9	218673	219460	PFI0230c	bacterial histone-like protein putative	0.164
chr9	222269	224154	PFI0235w	replication factor A-related protein putative	-0.145
chr9	225017	232829	PFI0240c	Cu2%2B -transporting ATPase Cu2%2B transporter	-0.116
chr9	234499	235344	PFI0245c	hypothetical protein conserved	0.139
chr9	236357	242967	PFI0250c	hypothetical protein conserved	-0.236
chr9	245259	248858	PFI0255c	mitochondrial carrier protein putative	0.086
chr9	251351	269707	PFI0260c	dynein heavy chain putative	1.884
chr9	270738	274787	PFI0265c	RhopH3	-0.060
chr9	278366	279085	PFI0270w	hypothetical protein conserved	-0.323
chr9	280204	284099	PFI0275w	hypothetical protein conserved	0.581
chr9	284197	286637	PFI0280c	autophagocytosis associated protein putative	0.274
chr9	289047	292894	PFI0285w	hypothetical protein conserved	-0.147
chr9	293582	296614	PFI0290c	beta subunit of coatomer complex putative	-0.116
chr9	298662	304176	PFI0295c	RNB-like protein putative	0.113
chr9	305067	306623	PFI0300w	developmental protein putative	-0.184
chr9	307300	309036	PFI0305c	hypothetical protein conserved	0.535
chr9	310688	311992	PFI0310w	Maf-like protein putative	-0.235
chr9	312471	314984	PFI0315c	hypothetical protein conserved	-0.029
chr9	318308	319543	PFI0320w	arginase putative	-0.499
chr9	320808	323339	PFI0325c	hypothetical protein conserved	-0.379
chr9	330496	334594	PFI0330c	hypothetical protein conserved	0.296
chr9	330496	331962	PFI0328c	conserved Plasmodium protein unknown function	0.061
chr9	335068	336763	PFI0335w	tubulin-specific chaperone putative	-0.028
chr9	337557	337913	PFI0336w	conserved Plasmodium protein unknown function	0.909
chr9	338133	342401	PFI0340c	hypothetical protein conserved	0.047
chr9	345885	349286	PFI0345w	GTPase activator putative	-0.193
chr9	350179	350955	PFI0350c	hypothetical protein conserved	0.089
chr9	351948	354716	PFI0355c	ATP-dependent heat shock protein putative	-0.252
chr9	356658	357676	PFI0360c	hypothetical protein conserved	-0.206
chr9	360528	363208	PFI0365w	hypothetical protein conserved	-0.031
chr9	364062	364901	PFI0370c	subunit of proteaseome activator complex putative	-0.391
chr9	366918	367493	PFI0375w	ribosomal protein L35 putative	0.141
chr9	367858	368782	PFI0380c	formylmethionine deformylase putative	-0.401
chr9	370444	371547	PFI0385c	S1%2FP1nuclease putative	-0.364
chr9	373755	375578	PFI0390c	hypothetical membrane protein conserved	0.064

chr9	378027	382988	PFI0395w	hypothetical protein conserved	-0.160
chr9	383642	385708	PFI0400c	hypothetical membrane protein conserved	0.027
chr9	387918	388728	PFI0405w	hypothetical protein conserved	0.178
chr9	389597	398007	PFI0410c	hypothetical protein conserved	0.012
chr9	399194	400717	PFI0415c	ribosomal RNA methyltransferase putative	-0.289
chr9	401487	403291	PFI0420c	tRNA pseudouridine synthase putative	0.097
chr9	404304	406277	PFI0425w	transporter putative	0.056
chr9	406514	409657	PFI0430c	hypothetical protein conserved	0.074
chr9	410318	413833	PFI0435c	hypothetical protein conserved	0.040
chr9	415197	417425	PFI0440w	hypothetical protein conserved	0.017
chr9	418200	419887	PFI0445c	hypothetical protein conserved	0.588
chr9	420477	421674	PFI0450c	apoptosis-related protein putative	-0.018
chr9	424163	428098	PFI0455w	exoribonuclease putative	-0.389
chr9	430570	432039	PFI0460w	hypothetical protein conserved	-0.955
chr9	433230	435365	PFI0465c	hypothetical protein conserved	0.148
chr9	440906	444016	PFI0470w	FHA domain protein putative	-0.338
chr9	445779	446320	PFI0475w	small nuclear ribonucleoprotein %28snRNP%29 putative	-0.570
chr9	447919	452040	PFI0480w	helicase with Zn-finger motif putative	-0.105
chr9	452390	455734	PFI0485c	hypothetical protein conserved	-0.159
chr9	456950	461309	PFI0490c	hypothetical protein conserved	-0.039
chr9	464550	473759	PFI0495w	hypothetical protein conserved	0.090
chr9	475662	476960	PFI0500w	hypothetical protein conserved	0.109
chr9	477768	481349	PFI0505c	selenide water dikinase putative	0.051
chr9	482452	487545	PFI0510c	hypothetical protein conserved	0.255
chr9	489458	490856	PFI0515w	prenylated protein putative	0.156
chr9	491266	492897	PFI0520w	actin-like protein putative	-0.171
chr9	494154	495497	PFI0525w	nucleotide binding protein putative	-0.443
chr9	496141	497718	PFI0530c	DNA primase large subunit putative	-0.224
chr9	500174	502724	PFI0535w	Phosphatidylinositol N-acetylglucosaminyltransferase pu	0.414
chr9	504403	508121	PFI0540w	hypothetical protein conserved	-0.036
chr9	509925	510518	PFI0545w	hypothetical protein conserved	0.127
chr9	511488	521520	PFI0550w	cysteine repeat modular protein homologue putative	2.107
chr9	522212	526009	PFI0555c	hypothetical protein conserved	0.058
chr9	526318	527969	PFI0560c	hypothetical protein conserved	0.312
chr9	530011	531435	PFI0565w	hypothetical protein conserved	0.022

chr9	532332	535589	PFI0570w	GTP-binding protein putative	-0.307
chr9	535842	536672	PFI0575c	hypothetical protein conserved	0.384
chr9	538212	539863	PFI0580c	Falstatin putative	-0.230
chr9	541024	545730	PFI0585c	hypothetical protein conserved	0.059
chr9	546791	548092	PFI0590c	hypothetical protein conserved	0.004
chr9	549076	550437	PFI0595c	hypothetical membrane protein conserved	-0.296
chr9	551417	553158	PFI0605c	hypothetical protein conserved	-0.399
chr9	556081	557949	PFI0610w	hypothetical protein conserved	0.081
chr9	559135	561970	PFI0615w	hypothetical protein conserved	0.138
chr9	562668	562925	PFI0620w	hypothetical protein conserved	0.032
chr9	563320	565170	PFI0625c	hypothetical protein conserved	0.204
chr9	567274	568631	PFI0630w	26S proteasome regulatory subunit putative	-0.180
chr9	569115	570365	PFI0635c	hypothetical protein conserved	0.297
chr9	571022	573829	PFI0640c	hypothetical protein conserved	0.084
chr9	575670	576500	PFI0645w	EF-1B	0.019
chr9	576951	579017	PFI0650c	hypothetical protein conserved	-0.176
chr9	580751	581459	PFI0655c	hypothetical protein conserved	-0.352
chr9	582331	583959	PFI0660c	protease putative	-0.089
chr9	584717	588412	PFI0665w	hypothetical protein conserved	0.039
chr9	589254	591233	PFI0670w	hypothetical protein conserved	-0.239
chr9	593098	593547	PFI0675w	hypothetical protein	-0.015
chr9	594263	597225	PFI0680c	hypothetical protein conserved	0.141
chr9	597685	600552	PFI0685w	pseudouridylate synthase putative	-0.301
chr9	601067	604318	PFI0690c	hypothetical protein conserved	-0.039
chr9	608352	610066	PFI0695c	phospholipid or glycerol acyltransferase putative	-0.664
chr9	613195	615483	PFI0700c	Met-10%2B like protein putative	-0.149
chr9	617954	619826	PFI0705w	hypothetical protein conserved	0.108
chr9	620779	626140	PFI0710c	hypothetical protein conserved	-0.097
chr9	627745	628310	PFI0715w	Zinc binding protein putative	-0.224
chr9	630648	632198	PFI0720w	transporter	-0.628
chr9	633565	634191	PFI0725c	GINS complex subunit Psf3 putative	0.117
chr9	636612	638741	PFI0730w	BSD domain putative	0.028
chr9	639649	641250	PFI0735c	NADH dehydrogenase putative	-0.220
chr9	643223	644034	PFI0740c	ubiquitin conjugating enzyme putative	-0.400
chr9	645623	646591	PFI0745w	hypothetical protein conserved	0.006

chr9	646942	649107	PFI0750c	hypothetical protein conserved	-0.155
chr9	650576	654832	PFI0755c	6-phosphofructokinase putative	-0.788
chr9	657409	659403	PFI0760w	hypothetical protein conserved	0.280
chr9	660167	660883	PFI0765w	hypothetical protein conserved	0.260
chr9	661472	662317	PFI0770c	hypothetical protein conserved	-0.220
chr9	663909	664562	PFI0775w	glycolipid transfer protein putative	-0.022
chr9	665969	666469	PFI0780w	hypothetical protein conserved	0.085
chr9	667246	671716	PFI0785c	sugar transporter	-0.148
chr9	674491	675148	PFI0790w	thioredoxin putative	-0.131
chr9	675913	679623	PFI0795w	hypothetical protein conserved	-0.041
chr9	680222	681928	PFI0800c	hypothetical protein conserved	-0.050
chr9	683553	691112	PFI0805w	hypothetical protein conserved	0.240
chr9	691559	693560	PFI0810c	apicoplast Ufd1 precursor	-0.053
chr9	694595	695254	PFI0815c	hypothetical protein conserved	-0.052
chr9	696853	698019	PFI0820c	RNA-binding protein putative	-0.182
chr9	703358	703507	PFI0823w	hypothetical protein conserved	-0.310
chr9	705347	706345	PFI0825w	hypothetical protein conserved	0.144
chr9	706582	712062	PFI0830c	hypothetical protein conserved	-0.129
chr9	713206	715179	PFI0835c	N-glycosylase%2FDNA lyase putative	-0.097
chr9	716315	717496	PFI0840w	hypothetical membrane protein conserved	-0.080
chr9	718511	719530	PFI0845w	hypothetical protein conserved	-0.099
chr9	720980	724855	PFI0850w	hypothetical protein conserved	0.060
chr9	726693	728738	PFI0855w	hypothetical protein conserved	-0.101
chr9	729664	732126	PFI0860c	ATP-dependent RNA helicase putative	-0.346
chr9	732941	734017	PFI0865w	ATP binding protein putative	-0.360
chr9	734619	735932	PFI0870w	hypothetical protein conserved	0.189
chr9	737975	740266	PFI0875w	Heat shock protein	-0.286
chr9	742484	743674	PFI0880c	acid phosphatase putative	-0.566
chr9	747163	748189	PFI0885w	heme binding protein putative	-0.140
chr9	748668	749876	PFI0890c	large ribosomal subunit protein L3 prokaryotic %2850S%	-0.606
chr9	751348	752412	PFI0895c	Mov34 domain-containing protein	-0.150
chr9	754287	760259	PFI0900w	hypothetical protein conserved	0.023
chr9	761306	762716	PFI0905w	hypothetical protein	0.044
chr9	764838	767024	PFI0910w	DNA helicase putative	-0.325
chr9	769442	772095	PFI0915w	hypothetical protein conserved	-0.241

chr9	772636	774369	PFI0920c	Dihydrouridine synthase putative	-0.375
chr9	777548	780739	PFI0925w	gamma-glutamylcysteine synthetase	-0.357
chr9	781358	782665	PFI0930c	Nucleosome assembly protein	-0.218
chr9	786146	788202	PFI0935w	DNAJ-like molecular chaperone protein putative	0.057
chr9	789504	790468	PFI0940c	PPPDE peptidase family putative	0.007
chr9	793207	794157	PFI0945w	hypothetical protein conserved	-0.507
chr9	795529	797460	PFI0950w	protein disulfide isomerase putative	-0.357
chr9	798488	799918	PFI0955w	sugar transporter	-0.437
chr9	800693	802126	PFI0960w	dolichyl-diphosphooligosaccharide--protein-glyc ot ransfe	0.341
chr9	803019	803747	PFI0965w	conserved protein putative	0.140
chr9	804407	807910	PFI0970c	TLD domain putative	-0.091
chr9	809649	819794	PFI0975c	hypothetical protein conserved	0.065
chr9	822655	824583	PFI0980w	hypothetical membrane protein conserved	-0.315
chr9	825323	827130	PFI0985c	chaperone protein	0.149
chr9	827720	830671	PFI0990c	CS domain protein putative	-0.310
chr9	831659	832638	PFI0995w	hypothetical protein conserved	-0.007
chr9	833744	839302	PFI1000w	hypothetical protein conserved	0.150
chr9	841526	842645	PFI1005w	ADP-ribosylation factor-like protein putative	-0.399
chr9	843531	847595	PFI1010w	hypothetical protein conserved	0.023
chr9	849505	851286	PFI1015w	hypothetical protein conserved	-0.193
chr9	852515	854047	PFI1020c	Inosine-5%27-monophosphate dehydrogenase	-0.962
chr9	855506	857276	PFI1025w	RNA-binding protein putative	0.189
chr9	857577	858819	PFI1030c	ubiquitin conjugating enzyme E2 putative	-0.041
chr9	861394	862316	PFI1035w	hypothetical protein conserved	0.338
chr9	862771	870481	PFI1040c	hypothetical membrane protein conserved	0.182
chr9	871816	873440	PFI1045w	hypothetical protein conserved	-0.150
chr9	873961	874947	PFI1050c	Nifu-like protein putative	0.168
chr9	875592	876857	PFI1055w	hypothetical protein conserved	0.172
chr9	877994	883107	PFI1060w	hypothetical protein conserved	0.065
chr9	883505	885582	PFI1065c	hypothetical protein conserved	-0.021
chr9	886055	887379	PFI1070c	BRIX domain putative	-0.066
chr9	888549	889295	PFI1075w	hypothetical protein conserved	-0.217
chr9	890047	893257	PFI1080w	hypothetical protein conserved	-0.084
chr9	895405	900294	PFI1085w	ubiquitin-like protein putative	-0.222
chr9	903352	904560	PFI1090w	s-adenosylmethionine synthetase putative	-0.418

chr9	906474	908347	PFI1095w	hypothetical protein conserved	-0.165
chr9	908714	912051	PFI1100w	para-aminobenzoic acid synthetase	-0.170
chr9	913220	914470	PFI1105w	Phosphoglycerate kinase	-0.735
chr9	917189	918475	PFI1110w	glutamate--ammonia ligase %28glutamine synthetase%2	-0.390
chr9	919099	921292	PFI1115c	pre-mRNA splicing factor protein putative	0.220
chr9	922282	935362	PFI1120c	hypothetical protein conserved	0.176
chr9	936858	938335	PFI1125c	3-oxoacyl-%28acyl-carrier protein%29 reductase putativ	-0.183
chr9	939570	940577	PFI1130c	DNA-directed RNA polymerase II putative	-0.190
chr9	941820	942488	PFI1135c	OTU-like cysteine protease putative	-0.592
chr9	944164	946458	PFI1140w	NADPH-cytochrome p450 reductase	-0.197
chr9	947023	949488	PFI1145w	MAC%2Fperforin putative	-0.540
chr9	950398	953712	PFI1150w	hypothetical protein conserved	-0.034
chr9	956595	957825	PFI1155w	hypothetical protein conserved	-0.069
chr9	958753	959907	PFI1160w	Lipoate-protein ligase A type 2	-0.197
chr9	960171	961380	PFI1165c	hypothetical protein conserved	-0.266
chr9	961923	963548	PFI1170c	Thioredoxin reductase	-0.818
chr9	966785	968059	PFI1175c	RNA-binding protein putative	-0.113
chr9	972538	976627	PFI1180w	hypothetical protein conserved	-0.206
chr9	977859	979955	PFI1185c	hypothetical protein conserved	0.041
chr9	981142	984324	PFI1190w	hypothetical protein conserved	-0.052
chr9	984805	986007	PFI1195c	Thiamine pyrophosphokinase	-0.209
chr9	988967	992905	PFI1200w	hypothetical protein conserved	-0.370
chr9	993301	997665	PFI1205c	hypothetical membrane protein conserved	0.209
chr9	999453	1005835	PFI1210w	hypothetical protein conserved	-0.045
chr9	1006763	1008532	PFI1215w	splicesome-associated protein putative	-0.414
chr9	1009053	1009966	PFI1216w	telomeric repeat binding factor 1	-0.145
chr9	1010370	1011825	PFI1220w	hypothetical protein conserved	0.206
chr9	1012108	1013853	PFI1225w	hypothetical protein conserved	-0.061
chr9	1014468	1015508	PFI1230c	hypothetical protein conserved	-0.292
chr9	1017425	1018908	PFI1235w	methyltransferase putative	-0.223
chr9	1019154	1020893	PFI1240c	prolyl-t-RNA synthase putative	-0.069
chr9	1021848	1023610	PFI1245c	Protein phosphatase-beta	-0.766
chr9	1025483	1026343	PFI1250w	thioredoxin putative	-0.150
chr9	1027084	1027694	PFI1255w	hypothetical protein conserved	-0.406
chr9	1028651	1030000	PFI1260c	Histone deacetylase	-1.317

chr9	1033305	1038569	PFI1265w	hypothetical protein conserved	-0.065
chr9	1040703	1041506	PFI1270w	hypothetical protein conserved	-0.479
chr9	1043532	1047784	PFI1275w	protein kinase putative	-0.007
chr9	1048270	1057806	PFI1280c	protein kinase putative	0.012
chr9	1063043	1063198	PFI1281w	conserved Plasmodium protein unknown function	-0.535
chr9	1064742	1067913	PFI1285w	hypothetical protein conserved	-0.065
chr9	1068551	1070307	PFI1290w	protein kinase putative	-0.307
chr9	1070955	1072945	PFI1295c	monocarboxylase transporter	-0.268
chr9	1075905	1081673	PFI1300c	hypothetical protein conserved	-0.060
chr9	1084686	1088785	PFI1305w	hypothetical membrane protein conserved	-0.011
chr9	1089901	1092420	PFI1310w	NAD synthase putative	-0.093
chr9	1092834	1096508	PFI1315c	hypothetical protein conserved	0.109
chr9	1097314	1098230	PFI1320c	hypothetical protein conserved	-0.415
chr9	1099065	1099740	PFI1325w	CS domain protein putative	-0.355
chr9	1100243	1100821	PFI1330c	hypothetical protein conserved	-0.168
chr9	1107402	1112646	PFI1335w	hypothetical protein conserved	0.325
chr9	1113948	1115993	PFI1340w	fumarate hydratase putative	-0.498
chr9	1116406	1119937	PFI1345c	hypothetical protein conserved	0.184
chr9	1120091	1121321	PFI1350c	dynein light chain putative	0.103
chr9	1123851	1125848	PFI1355w	hypothetical protein conserved	-0.163
chr9	1126980	1127918	PFI1360c	serine%2Fthreonine protein phosphatase putative	-0.751
chr9	1128861	1129930	PFI1365w	cytochrome c oxidase subunit putative	-0.732
chr9	1131064	1132125	PFI1370c	phosphatidylserine decarboxylase	-0.621
chr9	1135535	1136381	PFI1375w	cytochrome C oxidase putative	-0.178
chr9	1136956	1138173	PFI1380c	hypothetical protein conserved	0.136
chr9	1139930	1142377	PFI1385c	hypothetical protein conserved	0.343
chr9	1142961	1144469	PFI1390w	hypothetical protein conserved	0.036
chr9	1145354	1146028	PFI1395w	hypothetical protein conserved	-0.593
chr9	1146229	1147187	PFI1400c	hypothetical protein conserved	-0.173
chr9	1148151	1148492	PFI1405c	hypothetical protein conserved	-0.010
chr9	1150638	1155143	PFI1410c	hypothetical protein conserved	0.081
chr9	1156237	1158699	PFI1415w	Serine%2FThreonine protein kinase putative	-0.175
chr9	1159866	1160465	PFI1420w	guanylate kinase putative	-0.248
chr9	1161070	1162529	PFI1425w	hypothetical protein conserved	-0.089
chr9	1163560	1165465	PFI1430w	hypothetical protein conserved	0.405

chr9	1167832	1169567	PFI1435w	RNA binding function putative	0.053	
chr9	1171329	1173473	PFI1440w	hypothetical protein conserved	0.010	
chr9	1175193	1180752	PFI1445w	High molecular weight rhoptry protein-2	-0.347	
chr9	1181208	1181986	PFI1450c	hypothetical protein conserved	0.395	
chr9	1182677	1183685	PFI1455c	hypothetical protein conserved	-0.491	
chr9	1185097	1186892	PFI1460c	hypothetical protein conserved	0.259	
chr9	1187653	1188886	PFI1463w	hypothetical protein conserved	0.235	
chr9	1189636	1191312	PFI1465w	procollagen lysine 5-dioxygenase putative	0.112	
chr9	1191777	1198452	PFI1470c	leucine-rich repeat protein 8 LRR8	0.325	
chr9	1201802	1206964	PFI1475w	merozoite surface protein 1 precursor	0.090	
chr9	1207964	1211693	PFI1480w	hypothetical protein conserved	0.203	
chr9	1212444	1216490	PFI1485c	diacylglycerol kinase putative	0.214	
chr9	1218383	1220990	PFI1490c	large cyclophilin-like protein	0.303	
chr9	1221977	1223596	PFI1495w	hypothetical protein conserved	0.191	
chr9	1224981	1239336	PFI1500w	hypothetical membrane protein conserved	0.354	9cenL
chr9	1247261	1247930	PFI1835c	Nifu-like protein putative	0.770	9cenR
chr9	1248856	1251591	PFI1505c	hypothetical protein conserved	0.439	
chr9	1254805	1257282	PFI1510w	Nucleolar protein Nop52 putative	0.197	
chr9	1258539	1259557	PFI1515w	hypothetical protein conserved	0.107	
chr9	1261017	1261934	PFI1520w	hypothetical protein	0.020	
chr9	1263337	1264561	PFI1525w	hypothetical protein conserved	-0.047	
chr9	1264741	1266005	PFI1530c	hypothetical protein conserved	0.164	
chr9	1268267	1269405	PFI1535w	hypothetical protein conserved	0.142	
chr9	1270145	1272646	PFI1540w	hypothetical protein conserved	-0.140	
chr9	1273072	1273920	PFI1545c	proteasome precursor putative	-0.280	
chr9	1275258	1276285	PFI1550c	hypothetical protein conserved	0.006	
chr9	1277997	1279172	PFI1555w	hypothetical membrane protein	-0.276	
chr9	1279209	1283511	PFI1560c	hypothetical protein conserved	-0.077	
chr9	1286998	1288362	PFI1565w	conserved protein	-0.001	
chr9	1289305	1291017	PFI1570c	aminopeptidase putative	-0.381	
chr9	1292107	1293633	PFI1575c	peptide release factor putative	-0.143	
chr9	1294259	1296634	PFI1580c	DHHC-type zinc finger protein putative	-0.140	
chr9	1297275	1298124	PFI1585c	30S ribosomal protein S6-like protein putative	-0.175	
chr9	1298855	1302565	PFI1615c	hypothetical protein conserved	0.085	
chr9	1303380	1307779	PFI1590c	hypothetical protein conserved	0.256	

chr9	1309457	1309933	PFI1595c	hypothetical protein conserved	-0.065
chr9	1311430	1313715	PFI1600w	mRNA processing protein putative	-0.302
chr9	1314542	1317587	PFI1605w	hypothetical protein conserved	0.009
chr9	1317994	1319458	PFI1610c	calcylin binding protein-like putative	0.043
chr9	1320401	1324689	PFI1615w	hypothetical protein conserved	0.079
chr9	1325238	1329776	PFI1620c	hyptohetical protein	-0.044
chr9	1331339	1332793	PFI1625c	organelle processing peptidase putative	-0.745
chr9	1334105	1334784	PFI1630c	hypothetical protein conserved	0.108
chr9	1335729	1337936	PFI1635w	delta tubulin putative	-0.052
chr9	1338164	1339284	PFI1640c	hypothetical protein conserved	-0.122
chr9	1340054	1341982	PFI1645c	hypothetical protein conserved	0.176
chr9	1343374	1346544	PFI1650w	DNA excision-repair helicase putative	-0.436
chr9	1346847	1347284	PFI1655c	hypothetical protein conserved	0.087
chr9	1348011	1348448	PFI1660w	hypothetical protein conserved	-0.394
chr9	1349779	1350381	PFI1665w	uncharacterised trophozoite protein	-0.482
chr9	1350825	1351490	PFI1670c	vacuolar ATP synthase subunit E putative	-0.238
chr9	1354570	1355484	PFI1675w	hypothetical protein conserved	0.236
chr9	1357400	1359251	PFI1680w	hypothetical protein conserved	0.081
chr9	1361924	1363837	PFI1685w	cAMP-dependent protein kinase catalytic subunit	-0.316
chr9	1364245	1365988	PFI1690c	hypothetical protein conserved	0.052
chr9	1366558	1367139	PFI1695c	small nuclear ribonucleoprotein %28snRNP%29 putative	-0.206
chr9	1367385	1367603	PFI1696c	conserved protein unknown function	0.246
chr9	1368583	1372026	PFI1700c	vesicle transport protein putative	0.021
chr9	1373007	1373711	PFI1705w	hypothetical protein conserved	0.101
chr9	1377942	1379741	PFI1710w	cytoadherence-linked protein	0.043
chr9	1400789	1401893	PFI1715w	hypothetical protein conserved	-0.193
chr9	1405181	1407011	PFI1720w	Gametocyte-implicated protein %28Fragment%29	-0.414
chr9	1409188	1409997	PFI1725w	hypothetical protein conserved	-0.146
chr9	1413829	1419743	PFI1730w	cytoadherence linked asexual protein 9%28CLAG9%29	-0.035
chr9	1420472	1422796	PFI1735c	ring exported protein REX	0.097
chr9	1427463	1428011	PFI1740c	hypothetical protein	0.109
chr9	1430596	1431135	PFI1745c	hypothetical protein conserved	0.022
chr9	1433321	1434097	PFI1750c	hypothetical protein conserved	-0.387
chr9	1436676	1437970	PFI1755c	hypothetical protein conserved	0.090
chr9	1440772	1441649	PFI1760w	hypothetical protein conserved	0.359

chr9	1444256	1444835	PFI1765c	hypothetical protein	0.374
chr9	1446917	1447935	PFI1770w	hypothetical protein conserved in P.falciparum	2.252
chr9	1454410	1455708	PFI1775w	hydrolase putative	0.884
chr9	1458380	1459850	PFI1780w	hypothetical protein	2.098
chr9	1462388	1463647	PFI1785w	hypothetical protein conserved in P.falciparum	4.272
chr9	1466102	1467043	PFI1790w	hypothetical protein conserved in P.falciparum	4.349
chr9	1468534	1469405	PFI1795c	hypothetical protein	4.376
chr9	1471839	1472912	PFI1800w	hydrolase putative	5.020
chr9	1474419	1475692	PFI1805w	rifin	5.178
chr9	1477514	1478724	PFI1810w	rifin	4.084
chr9	1480935	1482199	PFI1815c	rifin	4.484
chr9	1486058	1490175	PFI1820w	erythrocyte membrane protein 1 %28PfEMP1%29	5.531
chr9	1495567	1503324	PFI1830c	erythrocyte membrane protein 1 %28PfEMP1%29	4.598
