# Supplementary material for: Plasmodium falciparum Heterochromatin Protein 1 Marks Genomic Loci Linked to Phenotypic Variation of Exported Virulence Factors
Source: PLoS Pathog. 2009 Sep 4;5(9):e1000569. doi: 10.1371/journal.ppat.1000569 (PMC2731224; doi:10.1371/journal.ppat.1000569)
Supplement: Table S2 — 425 PfHP1-enriched genes are mostly members of P. falciparum lineage-specific gene families coding for exported proteins. The table lists all 425 genes bound by PfHP1 (>1.6 (log2) recovery over input). Information contained in columns A-H was retrieved from PlasmoDB v5.5 (www.plasmodb.org). Clustering of genes into the class of predicted exported protein families (columns I and J) was done according to previously published information [3],[89],[90]. Column K lists the log2 ratios of PfHP1 ChIP over input for each gene in the list. Column L lists the log2 ratios of H3K9me3 ChIP over input for each PfHP1-bound gene reported recently by Salcedo-Amaya and co-workers using the same NimbleGen whole genome microarray (385,000 probes; 48b median spacing) [46]. Genes without H3K9me3-association are highlighted in green. Column M lists the log2 ratios of H3K9me3 ChIP over input for each PfHP1-bound gene published by Lopez-Rubio et al. [37]. Lopez-Rubio and colleagues used a NimbleGen platform based on a different probe set containing 145,000 features. Genes without H3K9me3-association are highlighted in bright yellow (not represented on the array) and pale yellow (below threshold). (0.04 MB PDF) [file ppat.1000569.s009.pdf]

Chr	Start	End	distance to previous HP1-occupied gene	Gene ID	PlasmoDB v5.5 reannotation	Pf_specific (www.plasmodb.org)	exported (Sargeant et al., 2006; Marti et al., 2004; Hiller et al., 2004)	gene family (Sargeant et al., 2006)	PfHP1-HA (log2 ChIP/input)	H3K9me3 (log2 ChIP/input) (Salcedo-Amaya et al., 2009)	H3K9me3 (log2 ChIP/input) (Lopez-Rubio et al., 2009)
chr1	61500	61718	150	PF11_0016	hypothetical protein	1		hyp	5.174	2.072	ND
chr1	29733	37349		PFA0005w	erythrocyte membrane protein 1 (PfEMP1)	1	1	var	4.648	1.393	2.435
chr1	39205	40430	1856	PFA0010c	rifin	1	1	rifin	3.763	1.267	1.567
chr1	42590	46730	2160	PFA0015c	var-like protein	1	1	var	5.042	1.795	2.558
chr1	50586	51859	3856	PFA0020w	rifin	1	1	rifin	3.721	1.282	1.815
chr1	54001	55229	2142	PFA0030c	rifin	1	1	rifin	4.398	1.356	1.825
chr1	60005	61236	4776	PFA0040w	rifin	1	1	rifin	5.190	1.723	2.498
chr1	62420	63633	1184	PFA0045c	rifin	1	1	rifin	5.355	1.569	2.417
chr1	66050	67222	2417	PFA0050c	rifin	1	1	rifin	5.214	1.733	2.705
chr1	74796	75599	7574	PFA0065w	Pfmc-2TM Maurer's cleft two transmembrane protein	1	1	Pfmc-2tm	4.980	1.538	ND
chr1	76215	77042	616	PFA0070c	conserved Plasmodium falciparum protein, unknown function (pseudogene)	1			4.500	1.973	ND
chr1	81998	83339	4956	PFA0080c	rifin	1	1	rifin	4.155	1.461	2.199
chr1	85024	86385	1685	PFA0085c	erythrocyte membrane protein 1 (PfEMP1), pseudogene, exon2	1	1	var	5.384	1.707	ND
chr1	87436	88410	1051	PFA0090c	stevor	1	1	stevor	5.430	1.678	ND
chr1	90475	91653	2065	PFA0095c	rifin	1	1	rifin	5.051	1.492	3.026
chr1	93346	94066	1693	PFA0100c	Plasmodium exported protein (PHISTa), unknown function	1	1	phistA	4.692	1.427	2.34

chr1	95257	96046	1191	PFA0105w	stevor pseudogene	1	1	stevor	4.366	1.132	1.646
chr1	477431	477551	381385	MAL1_5.8s	5.8s rRNA	1			2.330	-0.375	ND
chr1	477552	478427	1	MAL1_ITS2	ITS2 A-type	1			2.165	-0.221	0.074
chr1	478428	482531		MAL1_28s	28s rRNA (A-type)	1			1.699	-0.275	0.078
chr1	496393	501225		PFA0625w	Surface-associated interspersed gene 1.1 (SURFIN 1.1)	1	1	surfin	1.895	0.321	-0.187
chr1	502108	504017	23681	PFA0630c	Plasmodium exported protein, unknown function	1	1		3.455	0.264	0.49
chr1	513235	519004		PFA0650w	surface-associated interspersed gene pseudogene, (SURFIN) pseudogene	1	1	surfin	1.753	0.177	-0.294
chr1	528829	538073	24812	PFA0665w	DBL containing protein, unknown function	1			4.965	1.634	2.277
chr1	544202	548702	6129	PFA0675w	P. falciparum RESA-like protein with DnaJ domain	1	1	dnaj III	3.111	0.894	0.769
chr1	549319	550102	617	PFA0680c	Pfmc-2TM Maurer's cleft two transmembrane protein	1	1	Pfmc-2tm	4.525	1.967	ND
chr1	552298	553435	2196	PFA0685c	Plasmodium exported protein (hyp4), unknown function	1	1	hyp4	4.076	1.732	2.197
chr1	554362	555519	927	PFA0690w	Plasmodium exported protein, unknown function	1	1		4.228	1.632	2.181
chr1	555770	556651	251	PFA0695c	erythrocyte membrane protein 1 (PfEMP1), pseudogene, exon2	1	1	var	3.871	1.325	2.298
chr1	559923	560430	3272	PFA0700c	Plasmodium exported protein (hyp10), unknown function	1	1	hyp10	3.594	1.034	1.639
chr1	562299	563256	1869	PFA0705c	stevor pseudogene	1	1	stevor	4.923	1.568	2.117
chr1	565425	566564	2169	PFA0710c	rifin	1	1	rifin	4.787	1.411	2.327
chr1	571206	571993	4642	PFA0715c	Plasmodium exported protein (hyp7), unknown function	1	1	hyp7	3.380	0.864	1.382
chr1	573708	574404	1715	PFA0720w	hypothetical protein	1			3.381	1.512	1.58
chr1	579424	585509	5020	PFA0725w	Surface-associated interspersed protein 1.3 (SURFIN 1.3)	1	1	surfin	3.434	1.440	1.852
chr1	589111	590114	3602	PFA0735w	Plasmodium exported protein (PHISTa), unknown function	1	1	phistA	2.887	0.938	ND
chr1	593569	594907	3455	PFA0740w	rifin	1	1	rifin	4.843	1.641	2.723
chr1	600185	601192	5278	PFA0750w	stevor	1	1	stevor	5.223	1.598	2.67
chr1	602412	602844	1220	PFA0755w	erythrocyte membrane protein 1 (PfEMP1) pseudogene	1	1	var	5.127	1.635	2.465
chr1	605882	607251	3038	PFA0760w	rifin	1	1	rifin	4.148	1.162	1.68
chr1	609110	616613	1859	PFA0765c	erythrocyte membrane protein 1 (PfEMP1)	1	1	var	4.815	1.633	2.487
chr10	28491	36165		PF10_0001	erythrocyte membrane protein 1 (PfEMP1)	1	1	var	4.711	1.705	3.026
chr10	38051	39004	1886	PF10_0002	rifin	1	1	rifin	3.504	1.495	1.98
chr10	41774	42964	2770	PF10_0003	rifin	1	1	rifin	4.850	1.519	2.61
chr10	44817	46134	1853	PF10_0004	rifin	1	1	rifin	5.036	1.680	2.715
chr10	48053	50279	1919	PF10_0005	rifin	1	1	rifin	4.489	1.367	2.235
chr10	51350	52600	1071	PF10_0006	rifin	1	1	rifin	4.040	1.230	1.867
chr10	54543	54770	1943	PF10_0007	pseudogene, putative	1		hyp	3.043	0.978	1.085
chr10	55124	55508	354	PF10_0008	hypothetical protein	1	1	hyp	3.152	0.825	1.055
chr10	57969	58981	2461	PF10_0009	pseudogene, stevor, putative	1	1	stevor	5.330	1.725	2.401
chr10	60068	60777	1087	PF10_0011	erythrocyte membrane protein 1 (PfEMP1), truncated, degenerate	1	1	var	3.722	1.148	2.167
chr10	60889	61366	112	PF10_0012	erythrocyte membrane protein 1 (PfEMP1), truncated	1	1	var	2.630	0.546	0.983
chr10	1430152	1430364	1368786	PF10_0354	hypothetical protein	1			3.548	1.560	1.753
chr10	1432495	1434783	2131	PF10_0355	Erythrocyte membrane protein, putative	1	1		4.882	1.498	2.365
chr10	1436313	1437068	1530	PF10_0356	liver stage antigen-1	1			3.320	0.875	2.003
chr10	1519020	1547824	81952	PF10_0374	Pf 11-1 protein	1			2.565	-0.183	-0.224

chr10	1568708	1571814		PF10_0380	Serine/Threonine protein kinase, FIKK family	1	1	fikk	1.972	0.457	0.843
chr10	1574059	1575422	26235	PF10_0381	DnaJ protein, putative	1	1	dnaj III	3.562	1.466	ND
chr10	1581697	1582710	6275	PF10_0383	Plasmodium repeat_MYXSPDY protein	1		hyp	3.012	-0.893	ND
chr10	1591577	1592366	8867	PF10_0390	Pfmc-2TM Maurer's cleft two transmembrane protein	1	1	Pfmc-2tm	4.742	1.507	ND
chr10	1599899	1601076	7533	PF10_0393	rifin	1	1	rifin	5.406	1.725	ND
chr10	1602934	1603988	1858	PF10_0394	rifin	1	1	rifin	4.988	1.462	2.255
chr10	1605929	1606952	1941	PF10_0395	stevor, putative	1	1	stevor	5.227	1.622	2.297
chr10	1609062	1610421	2110	PF10_0396	rifin	1	1	rifin	5.022	1.543	2.408
chr10	1612529	1613734	2108	PF10_0397	rifin	1	1	rifin	4.869	1.394	2.724
chr10	1615803	1617069	2069	PF10_0398	rifin	1	1	rifin	5.155	1.644	2.513
chr10	1619085	1620243	2016	PF10_0399	rifin	1	1	rifin	5.113	1.550	2.125
chr10	1622367	1623683	2124	PF10_0400	rifin	1	1	rifin	5.212	1.647	2.3
chr10	1625771	1626823	2088	PF10_0401	rifin	1	1	rifin	5.238	1.637	2.665
chr10	1628913	1630022	2090	PF10_0402	rifin	1	1	rifin	5.222	1.573	2.593
chr10	1632397	1633664	2375	PF10_0403	rifin	1	1	rifin	5.151	1.438	2.508
chr10	1635595	1636778	1931	PF10_0404	rifin	1	1	rifin	4.754	1.331	2.053
chr10	1639170	1640517	2392	PF10_0405	rifin	1	1	rifin	4.495	1.544	1.809
chr10	1642400	1649947	1883	PF10_0406	erythrocyte membrane protein 1 (PfEMP1)	1	1	var	4.860	1.790	2.505
chr11	24160	31598		PF11_0007	erythrocyte membrane protein 1 (PfEMP1)	1	1	var	4.564	1.528	2.687
chr11	32666	42386	1068	PF11_0008	erythrocyte membrane protein 1 (PfEMP1)	1	1	var	4.960	1.677	2.4
chr11	45056	46233	2670	PF11_0009	rifin	1	1	rifin	5.139	1.616	2.489
chr11	48112	49307	1879	PF11_0010	rifin	1	1	rifin	5.120	1.749	2.509
chr11	51623	52751	2316	PF11_0011	rifin	1	1	rifin	4.922	1.681	ND
chr11	56792	57840	4041	PF11_0013	stevor, putative, degenerate	1	1	stevor	4.857	1.602	1.677
chr11	59550	60328	1710	PF11_0014	Plasmodium falciparum Maurer's Cleft 2 transmembrane domain protein 11.1	1	1	Pfmc-2tm	4.602	1.758	ND
chr11	60928	61350	600	PF11_0015	hypothetical protein	1		hyp	4.690	1.425	ND
chr11	66686	67965	4968	PF11_0529	rifin	1	1	rifin	4.365	1.414	1.949
chr11	70081	71289	2116	PF11_0020	rifin	1	1	rifin	3.551	1.543	ND
chr11	73244	74442	1955	PF11_0021	rifin	1	1	rifin	3.374	1.588	2.685
chr11	96964	99609	22522	PF11_0033	hypothetical protein	1		hyp	3.227	-0.399	ND
chr11	105357	107235	5748	PF11_0034	DnaJ protein, putative	1	1	dnaj III	4.463	1.568	ND
chr11	110028	111002	2793	PF11_0035	Plasmodium exported protein, unknown function	1	1		2.769	1.436	0.214
chr11	1839420	1842875		PF11_0478	kinesin motor domain containing protein				1.641	0.062	1.675
chr11	1843642	1853611		PF11_0479	conserved Plasmodium protein, unknown function			hyp	1.725	0.309	1.414
chr11	1991356	1994015	1880354	PF11_0512	RESA-like protein with PHIST and DnaJ domains	1	1	phistb dnaj	3.633	0.410	2.14
chr11	2001547	2003310	7532	PF11_0513	DnaJ protein, putative	1	1	dnaj III	3.788	1.227	1.103
chr11	2004751	2005008	1441	PF11_0514	Plasmodium exported protein (PHISTa), unknown function	1	1	phistA	4.402	1.373	2.407
chr11	2006703	2007826	1695	PF11_0515	rifin	1	1	rifin	4.812	1.404	2.214
chr11	2009872	2010899	2046	PF11_0516	stevor, putative	1	1	stevor	4.950	1.532	2.435
chr11	2013384	2014313	2485	PF11_0517	rifin	1	1	rifin	5.036	1.660	2.556

chr11	2015649	2016506	1336	PF11_0518	rifin, putative,truncated, pseudogene	1	1	rifin	5.425	1.588	2.616
chr11	2018711	2019964	2205	PF11_0519	rifin	1	1	rifin	5.220	1.635	2.48
chr11	2021851	2023031	1887	PF11_0520	rifin	1	1	rifin	5.226	1.706	ND
chr11	2025814	2035883	2783	PF11_0521	erythrocyte membrane protein 1 (PfEMP1)	1	1	var	5.260	1.797	2.509
chr12	16973	24497		PFL0005w	erythrocyte membrane protein 1 (PfEMP1)	1	1	var	4.777	1.709	2.521
chr12	26321	27687	1824	PFL0010c	rifin	1	1	rifin	3.362	1.764	2.125
chr12	30078	31261	2391	PFL0015c	rifin	1	1	rifin	4.658	1.567	2.788
chr12	32703	41940	1442	PFL0020w	erythrocyte membrane protein 1 (PfEMP1)	1	1	var	4.852	1.578	2.48
chr12	46788	56805	4848	PFL0030c	erythrocyte membrane protein 1 (PfEMP1)	1	1	var	3.755	0.607	2.323
chr12	61556	64336	4751	PFL0035c	acyl-CoA synthetase, PfACS7	1	1	acyl-CoA syn	3.573	0.738	1.711
chr12	764441	765144	700105	PFL0933w	null				4.175	1.705	ND
chr12	766647	774190	1503	PFL0935c	erythrocyte membrane protein 1 (PfEMP1)	1	1	var	4.781	1.725	2.912
chr12	776503	779926	2313	PFL0940c	erythrocyte membrane protein 1(PfEMP-1) pseudogene	1	1	var	4.806	1.807	2.88
chr12	783963	784823	4037	PFL0945w	erythrocyte membrane protein 1 (PfEMP1) pseudogene	1	1	var	3.841	1.292	2.112
chr12	907195	914493	75150	PFL1085w	transcription factor with AP2 domain			apiap2	4.872	1.736	2.499
chr12	1688595	1691151	774102	PFL1947c	VAR pseudogene, erythrocyte membrane protein 1 (PfEMP1) pseudogene	1	1	var	4.039	1.292	2.233
chr12	1694139	1703076	2988	PFL1950w	erythrocyte membrane protein 1 (PfEMP1)	1	1	var	4.742	1.636	2.691
chr12	1704501	1712479	1425	PFL1955w	erythrocyte membrane protein 1 (PfEMP1)	1	1	var	4.850	1.685	2.573
chr12	1719563	1727445	7084	PFL1960w	erythrocyte membrane protein 1 (PfEMP1)	1	1	var	4.753	1.621	2.737
chr12	1728763	1729755	1318	PFL1965w	rifin pseudogene	1	1	rifin	5.145	1.591	2.764
chr12	1735532	1743396	5777	PFL1970w	erythrocyte membrane protein 1 (PfEMP1)	1	1	var	4.772	1.416	2.496
chr12	2136038	2144859	392642	PFL2520w	reticulocyte-binding protein 3 homologue	1			2.753	0.079	-0.057
chr12	2188744	2189594	43885	PFL2580w	rifin, pseudogene	1	1	rifin	5.338	1.777	2.389
chr12	2190467	2191754	873	PFL2585c	rifin	1	1	rifin	5.155	1.718	2.334
chr12	2196141	2197106	4387	PFL2590w	Plasmodium exported protein (PHISTa), unknown function	1	1	phistA	2.623	1.225	ND
chr12	2198910	2199541	1804	PFL2595w	Plasmodium exported protein (PHISTa), unknown function	1	1	phistA	4.684	1.794	ND
chr12	2201479	2202670	1938	PFL2605w	rifin	1	1	rifin	4.919	1.365	2.594
chr12	2204708	2205698	2038	PFL2610w	stevor	1	1	stevor	5.288	1.637	2.506
chr12	2207814	2209099	2116	PFL2615w	rifin	1	1	rifin	5.382	1.558	2.381
chr12	2211548	2212538	2449	PFL2620w	stevor	1	1	stevor	5.187	1.574	ND
chr12	2214655	2216003	2117	PFL2625w	rifin	1	1	rifin	4.953	1.501	2.514
chr12	2218436	2219517	2433	PFL2630w	rifin	1	1	rifin	5.172	1.702	2.18
chr12	2221840	2222832	2323	PFL2635w	stevor	1	1	stevor	5.026	1.506	2.437
chr12	2224981	2226185	2149	PFL2640c	rifin	1	1	rifin	5.028	1.694	2.284
chr12	2228616	2229724	2431	PFL2645c	rifin	1	1	rifin	5.183	1.747	2.341
chr12	2234584	2235776	4860	PFL2655w	rifin	1	1	rifin	4.929	1.439	2.472
chr12	2238194	2239401	2418	PFL2660w	rifin	1	1	rifin	4.406	1.570	2.144
chr12	2241255	2248946	1854	PFL2665c	erythrocyte membrane protein 1 (PfEMP1)	1	1	var	4.688	1.661	2.394
chr13	21467	28890		MAL13P1.1	erythrocyte membrane protein 1 (PfEMP1)	1	1	var	3.532	1.185	2.183
chr13	30708	31984	1818	MAL13P1.2	rifin	1	1	rifin	4.327	1.476	1.891

chr13	34062	44845	2078	PF13_0003	erythrocyte membrane protein 1 (PfEMP1)	1	1	var	5.259	1.774	2.675
chr13	47686	48872	2841	PF13_0004	rifin	1	1	rifin	5.295	1.618	ND
chr13	50765	51960	1893	PF13_0005	rifin	1	1	rifin	4.420	1.543	2.035
chr13	54170	55362	2210	PF13_0006	rifin	1	1	rifin	4.142	1.414	2.099
chr13	57212	58525	1850	MAL13P1.4	rifin	1	1	rifin	5.002	1.727	2.366
chr13	60200	61275	1675	MAL13P1.6	erythrocyte membrane protein 1-like	1	1	var	5.286	1.877	ND
chr13	62618	63650	1343	MAL13P1.7	stevor	1	1	stevor	5.418	1.614	2.337
chr13	65651	66919	2001	MAL13P1.8	RIF pseudogene	1	1	rifin	4.970	1.598	ND
chr13	73202	74516	6283	PF13_0010	glycophorin binding protein family, Gbph	1	1	gbph	4.580	1.314	2.033
chr13	78891	79681	4375	MAL13P1.58	Plasmodium exported protein (PHISTa-like), unknown function	1	1	phistA	3.551	0.605	1.411
chr13	2194239	2194311		MAL13_tRNA_T	tRNA threonine				1.875	-0.465	ND
chr13	2697562	2701110	2617881	PF13_0355	conserved Plasmodium protein, unknown function				3.407	0.252	-0.145
chr13	2702833	2704776		MAL13P1.342	conserved Plasmodium protein, unknown function				1.799	0.112	-0.123
chr13	2856996	2864550	155886	MAL13P1.356	erythrocyte membrane protein 1 (PfEMP1)	1	1	var	4.551	1.670	2.632
chr14	1394	5344		PF14_0001	erythrocyte membrane protein 1 (PfEMP1), truncated, pseudogene	1	1	var	2.821	0.879	0.963
chr14	7209	8141	1865	PF14_0002	rifin	1	1	rifin	3.864	1.194	1
chr14	14127	15364	5986	PF14_0004	rifin	1	1	rifin	4.881	1.720	2.501
chr14	17470	18809	2106	PF14_0005	rifin	1	1	rifin	4.946	1.653	2.359
chr14	20898	22233	2089	PF14_0006	rifin	1	1	rifin	5.172	1.735	2.668
chr14	24338	25321	2105	PF14_0007	stevor	1	1	stevor	5.198	1.598	2.381
chr14	27322	28429	2001	PF14_0008	rifin	1	1	rifin	5.195	1.623	2.39
chr14	30084	31009	1655	PF14_0009	Plasmodium exported protein (PHISTa), unknown function	1	1	phistA	4.917	1.819	ND
chr14	34694	35774	3685	PF14_0010	Glycophorin binding protein family, Gbph	1	1	gbph	4.217	1.525	2.336
chr14	41290	43151	5516	PF14_0013	DnaJ protein, putative	1	1	dnaj III	2.772	0.670	1.237
chr14	3086996	3105006	3043845	PF14_0722	cysteine repeat modular protein 4			crmp	4.221	0.440	0.51
chr14	3106884	3111746	1878	PF14_0723	LCCL domain-containing protein CCP1				3.945	0.145	0.374
chr14	3147425	3148519	35679	PF14_0735	hypothetical protein	1			2.874	1.145	1.199
chr14	3151938	3154858	3419	PF14_0736	Plasmodium exported protein, unknown function	1	1		3.932	1.515	2.122
chr14	3168498	3170028	13640	PF14_0740	Plasmodium exported protein (hyp17), unknown function	1	1	hyp17	4.224	1.672	2.026
chr14	3170158	3171627	130	PF14_0741	hypothetical protein	1		hyp	4.360	1.646	2.3
chr14	3172907	3173579	1280	PF14_0742	Plasmodium exported protein (hyp6), unknown function	1	1	hyp6	4.189	1.556	2.296
chr14	3175803	3176393	2224	PF14_0743	Plasmodium exported protein (hyp15), unknown function	1	1	hyp15	4.624	1.698	1.95
chr14	3179559	3180545	3166	PF14_0744	Plasmodium exported protein, unknown function	1	1		4.928	1.516	2.292
chr14	3183574	3184287	3029	PF14_0745	hypothetical protein	1		hyp	4.639	1.529	2.276
chr14	3193220	3199413	8933	PF14_0747	surface -associated intersprsed gene 14.1 (SURFIN 14.1)	1	1	surfin	3.387	1.219	1.417
chr14	3202516	3203952	3103	PF14_0748	Plasmodium exported protein (PHISTa), unknown function	1	1	phistA	3.016	1.305	1.536
chr14	3209064	3211509	5112	PF14_0751	acyl-CoA synthetase, PfACS1b	1	1	acyl-CoA syn	2.708	1.479	2.002
chr14	3213516	3214552	2007	PF14_0752	Plasmodium exported protein (PHISTa), unknown function	1	1	phistA	3.185	1.348	1.78
chr14	3219854	3220204	5302	PF14_0754	hypothetical protein	1		hyp	3.018	0.962	1.164
chr14	3220861	3221511	657	PF14_0755	hypothetical protein	1	1	hyp	5.074	1.690	1.72

chr14	3223223	3224567	1712	PF14_0756	Conserved P. falciparum protein	1	1	rif-like	4.003	1.362	2.045
chr14	3228607	3229524	4040	PF14_0757	Plasmodium exported protein (PHISTa), unknown function	1	1	phistA	3.218	0.887	1.138
chr14	3232574	3236470	3050	PF14_0758	Plasmodium exported protein (hyp17), unknown function	1	1	hyp17	2.174	0.674	0.885
chr14	3239080	3239535	2610	PF14_0759	conserved Plasmodium protein, unknown function, pseudogene	1		PFD0075-like	4.825	1.659	1.878
chr14	3242844	3243564	3309	PF14_0760	Plasmodium exported protein, unknown function	1	1		2.018	0.482	1.203
chr14	3254027	3256489	10463	PF14_0761	acyl-CoA synthetase, PfACS1a	1	1	acyl-CoA syn	3.634	1.705	2.176
chr14	3260680	3261662		PF14_0763	Plasmodium exported protein (PHISTa), unknown function	1	1	phistA	1.918	0.974	ND
chr14	3266071	3267312	9582	PF14_0766	rifin	1	1	rifin	4.830	1.423	2.184
chr14	3269429	3270431	2117	PF14_0767	stevor, putative	1	1	stevor	5.216	1.623	ND
chr14	3272836	3273783	2405	PF14_0768	rifin	1	1	rifin	5.057	1.533	2.337
chr14	3276165	3277436	2382	PF14_0769	rifin	1	1	rifin	4.876	1.585	2.289
chr14	3279435	3280597	1999	PF14_0770	rifin	1	1	rifin	4.978	1.421	2.317
chr14	3282685	3283687	2088	PF14_0771	stevor, putative	1	1	stevor	5.119	1.684	2.458
chr14	3285835	3286938	2148	PF14_0772	rifin	1	1	rifin	5.013	1.800	2.578
chr14	3290888	3291436	3950	PF14_0773	erythrocyte membrane protein 1 (PfEMP1), truncated, pseudogene	1	1	var	4.543	1.486	2.264
chr2	25232	31168		PFB0010w	erythrocyte membrane protein 1 (PfEMP1)	1	1	var	4.622	1.608	2.491
chr2	33030	34259	1862	PFB0015c	rifin	1	1	rifin	3.785	1.278	2.218
chr2	35927	37249	1668	PFB0020c	erythrocyte membrane protein 1 (PfEMP1), exon2	1	1	var	5.151	1.764	2.689
chr2	38287	39303	1038	PFB0025c	stevor	1	1	stevor	5.294	1.560	2.64
chr2	41515	42858	2212	PFB0030c	rifin	1	1	rifin	4.769	1.563	2.92
chr2	45286	46800	2428	PFB0035c	rifin	1	1	rifin	4.484	1.423	2.542
chr2	48923	50147	2123	PFB0040c	rifin	1	1	rifin	5.259	1.689	2.433
chr2	51842	53124	1695	PFB0045c	erythrocyte membrane protein 1 (PfEMP1), truncated	1	1	var	5.380	1.823	ND
chr2	54418	54936	1294	PFB0050c	stevor pseudogene	1	1	stevor	5.595	1.648	2.702
chr2	57344	58421	2408	PFB0055c	rifin	1	1	rifin	5.155	1.562	2.788
chr2	60107	60319	1686	PFB0056c	hypothetical protein	1		hyp	4.208	1.609	ND
chr2	63157	64376	2838	PFB0060w	rifin	1	1	rifin	5.193	1.787	2.624
chr2	66550	67545	2174	PFB0065w	stevor, putative	1	1	stevor	5.260	1.520	2.628
chr2	69088	69771	1543	PFB0070w	Plasmodium exported protein (hyp10), unknown function	1	1	hyp10	4.608	1.392	1.753
chr2	370438	379845		PFB0405w	transmission-blocking target antigen s230				1.811	0.141	0.102
chr2	838841	844114	769070	PFB0935w	cytoadherence linked asexual protein 2			rhoph1/clag	2.174	0.652	0.66
chr2	844860	845837	746	PFB0946c	conserved Plasmodium falciparum protein family	1		hyp	3.602	0.929	1.98
chr2	847487	849117	1650	PFB0950w	conserved Plasmodium falciparum protein family	1		hyp	3.731	1.324	1.88
chr2	857433	858043	8316	PFB0954c	conserved Plasmodium protein, unknown function, pseudogene	1		PFD0075-like	2.642	1.069	1.647
chr2	858455	859069	412	PFB1070w	hypothetical protein	1		PFD0075-like	2.724	0.895	1.702
chr2	860353	861502	1284	PFB0955w	stevor	1	1	stevor	4.997	1.594	2.286
chr2	863384	863840	1882	PFB0960c	Pfmc-2TM family pseudogene	1	1	Pfmc-2tm	4.425	1.491	2.182
chr2	865914	866324	2074	PFB0965c	hypothetical protein, pseudogene	1		hyp	3.583	1.415	1.902
chr2	873129	873317		PFB0972w	hypothetical protein	1			1.653	0.429	1.688
chr2	873915	874109	7591	PFB0973c	hypothetical protein	1			3.188	1.020	ND

chr2	874129	874323	20	PFB0974c	erythrocyte membrane protein 1 (PfEMP1), truncated, degenerate	1	1	var	4.623	1.630	ND
chr2	878466	879249	4143	PFB0985c	Pfmc-2TM Maurer's cleft two transmembrane protein	1	1	Pfmc-2tm	4.814	1.738	ND
chr2	886915	887802	7666	PFB1000w	rifin	1	1	rifin	5.239	1.601	ND
chr2	889660	890745	1858	PFB1005w	rifin	1	1	rifin	5.373	1.514	2.615
chr2	892856	894206	2111	PFB1010w	rifin	1	1	rifin	4.848	1.569	2.655
chr2	896226	897420	2020	PFB1015w	rifin	1	1	rifin	4.937	1.461	2.655
chr2	899451	900445	2031	PFB1020w	stevor	1	1	stevor	4.968	1.649	2.218
chr2	902500	902853	2055	PFB1030w	conserved Plasmodium falciparum protein family	1		hyp	5.255	1.633	ND
chr2	904346	905775	1493	PFB1035w	rifin	1	1	rifin	4.807	1.577	2.35
chr2	907678	908861	1903	PFB1040w	rifin	1	1	rifin	5.051	1.384	2.449
chr2	909350	911054	489	PFB1045w	erythrocyte membrane protein 1 (PfEMP1), truncated	1	1	var	4.814	1.503	ND
chr2	913244	914457	2190	PFB1050w	rifin	1	1	rifin	3.924	1.274	1.125
chr2	916352	923648	1895	PFB1055c	erythrocyte membrane protein 1 (PfEMP1)	1	1	var	4.743	1.648	2.614
chr3	8394	10745		PFC0002c	hypothetical protein, conserved in P. falciparum	1		MAL8P1.335	4.563	0.865	ND
chr3	33641	41158	22896	PFC0005w	PfEMP1	1	1	var	4.901	1.581	2.515
chr3	43045	44255	1887	PFC0010c	rifin	1	1	rifin	3.753	1.243	1.261
chr3	48956	49949	4701	PFC0025c	stevor	1	1	stevor	5.275	1.776	2.757
chr3	52066	53260	2117	PFC0030c	rifin	1	1	rifin	5.184	1.646	2.448
chr3	58103	59390	4843	PFC0035w	rifin	1	1	rifin	5.035	1.620	2.62
chr3	61248	62459	1858	PFC0040w	rifin	1	1	rifin	5.376	1.970	ND
chr3	64604	65508	2145	PFC0045w	rifin	1	1	rifin	4.746	1.793	1.777
chr3	66136	68595	628	PFC0050c	acyl-CoA synthetase, PfACS2	1	1	acyl-CoA syn	5.061	1.694	2.227
chr3	73172	74152	4577	PFC0055w	Plasmodium exported protein (hyp13), unknown function	1	1	hyp13	4.126	1.121	1.953
chr3	74873	77071	721	PFC0060c	Serine/Threonine protein kinase, FIKK family	1	1	fikk	2.462	0.904	1.205
chr3	110750	114766	33679	PFC0105w	serine/threonine protein kinase, putative				3.226	1.008	1.308
chr3	116138	121415	1372	PFC0110w	Cytoadherence linked asexual protein 3.2	1		rhoph1/clag	4.766	1.621	2.342
chr3	122672	126915	1257	PFC0115c	erythrocyte membrane protein 1 (PfEMP1) pseudogene	1	1	var	5.466	2.084	2.852
chr3	132097	137339	5182	PFC0120w	Cytoadherence linked asexual protein 3.1	1		rhoph1/clag	2.991	0.795	0.964
chr3	844917	854174	707578	PFC0905c	conserved Plasmodium protein, unknown function				2.313	0.095	-0.144
chr3	999860	1000734	145686	PFC1070c	VARC pseudogene	1	1	var	4.112	1.339	ND
chr3	1004083	1004872	3349	PFC1080c	Pfmc-2TM Maurer's cleft two transmembrane protein	1	1	Pfmc-2tm	4.749	1.646	1.845
chr3	1012465	1013612	7593	PFC1095w	rifin (3D7-rifT3-5)	1	1	rifin	5.078	1.592	2.852
chr3	1015544	1016643	1932	PFC1100w	rifin	1	1	rifin	5.195	1.602	2.561
chr3	1018668	1019678	2025	PFC1105w	stevor (3D7-stevorT3-2)	1	1	stevor	5.495	1.734	2.509
chr3	1024241	1025599	4563	PFC1115w	rifin (3D7-rifT3-7)	1	1	rifin	3.951	1.023	1.581
chr3	1027492	1034924	1893	PFC1120c	var (3D7-varT3-2)	1	1	var	4.801	1.588	2.643
chr3	1046861	1050978	11937	PFC1125w	hypothetical protein, conserved in P. falciparum	1		MAL8P1.335	3.925	0.343	2.965
chr4	35153	44124		PFD0005w	erythrocyte membrane protein 1 (PfEMP1)	1	1	var	4.881	1.659	2.498
chr4	52002	63307	7878	PFD0020c	erythrocyte membrane protein 1 (PfEMP1)	1	1	var	5.029	1.785	2.646
chr4	66083	67386	2776	PFD0025w	rifin	1	1	rifin	4.495	1.603	2.274

chr4	69551	70923	2165	PFD0030c	rifin	1	1	rifin	4.725	1.683	2.436
chr4	73048	74020	2125	PFD0035c	stevor	1	1	stevor	5.194	1.579	2.422
chr4	76408	77560	2388	PFD0040c	rifin	1	1	rifin	5.022	1.565	2.55
chr4	79715	80787	2155	PFD0045c	rifin	1	1	rifin	5.000	1.551	2.365
chr4	85640	86898	4853	PFD0050w	rifin	1	1	rifin	5.231	1.571	2.562
chr4	88694	90022	1796	PFD0055w	rifin	1	1	rifin	5.009	1.644	2.576
chr4	91844	93055	1822	PFD0060w	rifin	1	1	rifin	5.268	1.822	2.556
chr4	95600	96631	2545	PFD0065w	stevor pseudogene	1	1	stevor	4.308	1.233	1.636
chr4	107134	108560		PFD0075w	conserved Plasmodium falciparum protein family	1		PFD0075-like	1.982	0.868	0.26
chr4	114830	117745	18199	PFD0085c	acyl-CoA synthetase, PfACS6	1	1	acyl-CoA syn	3.087	0.971	1.51
chr4	121052	122509		PFD0090c	Plasmodium exported protein (PHISTa), unknown function	1	1	phistA	1.596	1.051	0.752
chr4	130044	131920	12299	PFD0095c	Plasmodium exported protein (PHISTb), unknown function	1	1	phistB	2.211	0.782	1.462
chr4	134169	140925	2249	PFD0100c	surface-associated interspersed gene 4.1, (SURFIN4.1)	1	1	surfin	2.756	0.609	1.421
chr4	144098	153112		PFD0110w	reticulocyte-binding protein homologue 1	1			1.606	0.268	0.206
chr4	162335	163442	21410	PFD0120w	RIF pseudogene, RIFIN pseudogene	1	1	rifin	5.181	1.653	2.474
chr4	165737	166772	2295	PFD0125c	stevor	1	1	stevor	5.283	1.599	2.481
chr4	168969	169472	2197	PFD0134c	RIF pseudogene, RIFIN pseudogene	1	1	rifin	5.116	1.629	2.508
chr4	169474	170188	2	PFD0135c	rifin, truncated	1	1	rifin	4.794	1.268	2.381
chr4	173451	178412	3263	PFD0135w	erythrocyte membrane protein 1 (PfEMP1) pseudogene	1	1	var	4.666	1.628	2.571
chr4	552884	560707	374472	PFD0615c	erythrocyte membrane protein 1 (PfEMP1)	1	1	var	4.703	1.599	2.694
chr4	566284	567535	5577	PFD0620c	null	1			4.903	1.704	ND
chr4	566507	567535	-1028	PFD0595c	rifin, truncated	1	1	rifin	4.903	1.704	ND
chr4	568564	576239	1029	PFD0625c	erythrocyte membrane protein 1 (PfEMP1)	1	1	var	4.827	1.755	2.8
chr4	583793	591651	7554	PFD0630c	erythrocyte membrane protein 1 (PfEMP1)	1	1	var	5.266	1.749	ND
chr4	598932	606832	7281	PFD0635c	erythrocyte membrane protein 1 (PfEMP1)	1	1	var	5.173	1.828	2.601
chr4	610149	611439	3317	PFD0640c	rifin	1	1	rifin	5.278	1.796	2.667
chr4	616138	617416	4699	PFD0645w	rifin	1	1	rifin	4.793	1.774	2.415
chr4	618860	619174	1444	PFD0650w	hypothetical protein	1			5.658	2.146	2.225
chr4	619781	621052	607	PFD0655w	erythrocyte membrane protein 1 (PfEMP1) pseudogene	1	1	var	3.827	1.269	1.447
chr4	939470	946314	318418	PFD0995c	erythrocyte membrane protein 1 (PfEMP1)	1	1	var	5.111	1.509	2.656
chr4	962506	970050	16192	PFD1005c	erythrocyte membrane protein 1 (PfEMP1)	1	1	var	5.093	1.795	2.722
chr4	971526	972712	1476	PFD1010w	rifin	1	1	rifin	5.111	1.562	2.754
chr4	973470	981030	758	PFD1015c	erythrocyte membrane protein 1 (PfEMP1)	1	1	var	4.829	1.834	2.573
chr4	986508	987434	5478	PFD1020c	rifin	1	1	rifin	5.289	1.614	2.688
chr4	990641	991887	3207	PFD1025w	erythrocyte membrane protein 1 (PfEMP1)-like pseudogene	1	1	var	4.701	1.489	2.412
chr4	992282	993323	395	PFD1004c	hypothetical protein, conserved				2.339	0.700	1.073
chr4	1097578	1102275		PFD1155w	erythrocyte binding antigen-165				1.809	0.025	0.439
chr4	1104251	1111551		PFD1160w	surface-associated interspersed gene 4.2, (SURFIN4.2)	1	1	surfin	1.758	0.183	0.364
chr4	1131406	1132429	138083	PFD1185w	Plasmodium exported protein (PHISTa), unknown function	1	1	phistA	2.675	1.489	2.136
chr4	1133287	1133655	858	PFD1190c	hypothetical protein	1		PFD0075-like	4.765	1.651	2.408

chr4	1134195	1134520	540	PFD1195c	conserved Plasmodium protein, unknown function, pseudogene	1		PFD0075-like	4.231	1.505	2.153
chr4	1138042	1138802	3522	PFD1200c	Plasmodium exported protein (hyp6), unknown function	1	1	hyp6	2.948	1.155	1.611
chr4	1140213	1141012	1411	PFD1205w	Plasmodium exported protein (hyp15), unknown function	1	1	hyp15	3.789	1.561	2.017
chr4	1145209	1145718	4197	PFD1210w	Plasmodium exported protein (PHISTa), unknown function	1	1	phistA	4.590	1.444	1.926
chr4	1147614	1148446	1896	PFD1215w	Plasmodium exported protein (PHISTa), unknown function	1	1	phistA	4.349	1.657	2.085
chr4	1150294	1151319	1848	PFD1220c	stevor	1	1	stevor	4.838	1.759	2.488
chr4	1152896	1153810	1577	PFD1200w	RIF pseudogene, RIFIN pseudogene	1	1	rifin	5.148	1.501	ND
chr4	1156126	1157255	2316	PFD1230c	rifin	1	1	rifin	5.167	1.659	2.59
chr4	1176675	1183848	19420	PFD1245c	erythrocyte membrane protein 1 (PfEMP1)	1	1	var	4.765	1.712	2.588
chr4	1192754	1195747	8906	PFD1250w	hypothetical protein, conserved in P falciparum	1		MAL8P1.335	4.055	0.686	2.876
chr5	20929	28456		PFE0005w	erythrocyte membrane protein 1 (PfEMP1)	1	1	var	3.961	1.190	2.349
chr5	29923	30963	1467	PFE0015c	RIF pseudogene, RIFIN pseudogene	1	1	rifin	3.568	0.973	1.377
chr5	33056	34378	2093	PFE0020c	rifin	1	1	rifin	4.906	1.722	2.86
chr5	36464	37576	2086	PFE0025c	rifin	1	1	rifin	4.866	1.427	2.521
chr5	39443	40488	1867	PFE0030c	stevor pseudogene	1	1	stevor	4.287	1.652	2.261
chr5	41129	41403	641	PFE0035c	RIF pseudogene, RIFIN pseudogene	1	1	rifin	3.109	1.588	1.527
chr5	74509	79842		PFE0070w	interspersed repeat antigen, putative	1			1.710	0.090	0.058
chr5	1222419	1222748		PFE1495w	null				1.703	-0.229	ND
chr5	1322395	1322718	1280992	PFE1620c	erythrocyte membrane protein 1 (PfEMP1)	1	1	var	2.501	0.729	1.203
chr5	1322795	1323082	77	PFE1625c	erythrocyte membrane protein 1 (PfEMP1) pseudogene	1	1	var	2.981	1.229	1.797
chr5	1326240	1327424	3158	PFE1630w	rifin	1	1	rifin	4.659	1.402	2.653
chr5	1329844	1330623	2420	PFE1635w	RIF pseudogene, RIFIN pseudogene	1	1	rifin	4.161	1.380	2.2
chr5	1333465	1342959	2842	PFE1640w	erythrocyte membrane protein 1 (PfEMP1), truncated	1	1	var	3.835	1.481	2.475
chr6	653	1432		PFF0005c	erythrocyte membrane protein 1 (PfEMP1) pseudogene	1	1	var	5.019	1.534	2.343
chr6	3503	12835	2071	PFF0010w	erythrocyte membrane protein 1 (PfEMP1)	1	1	var	4.932	1.882	2.707
chr6	15065	16410	2230	PFF0015c	rifin	1	1	rifin	3.421	1.537	2.608
chr6	18586	22721	2176	PFF0020c	erythrocyte membrane protein 1 (PfEMP1)-like protein	1	1	var	5.602	1.853	2.591
chr6	26557	27830	3836	PFF0025w	rifin	1	1	rifin	4.051	1.526	2.549
chr6	29618	31484	1788	PFF0030c	erythrocyte membrane protein 1 (PfEMP1) pseudogene	1	1	var	4.426	1.260	ND
chr6	31965	33244	481	PFF0035c	rifin	1	1	rifin	5.159	1.519	2.663
chr6	35355	36613	2111	PFF0040c	rifin pseudogene	1	1	rifin	4.526	1.437	2.627
chr6	65785	67392	29172	PFF0075c	Plasmodium exported protein (PHISTb), unknown function	1	1	phistB	2.587	0.248	ND
chr6	68830	69690	1438	PFF0080c	TPR-like domain containing protein, putative	1			2.598	0.378	ND
chr6	70945	71932	1255	PFF0085w	Plasmodium exported protein (PHISTa), unknown function	1	1	phistA	2.603	-0.075	0.945
chr6	723114	731487	651182	PFF0845c	erythrocyte membrane protein 1 (PfEMP1)	1	1	var	4.823	1.588	2.586
chr6	735365	736322	3878	PFF0847w	Stevor, pseudogene	1	1	stevor	4.932	0.903	1.456
chr6	738256	739262	1934	PFF0850c	stevor	1	1	stevor	4.783	1.319	1.885
chr6	741266	742403	2004	PFF0855c	rifin	1	1	rifin	4.955	1.880	2.495
chr6	1295477	1296334		PFF1505w	TPR-like domain containing protein, putative	1			1.986	0.210	ND
chr6	1313711	1315256	571308	PFF1515c	erythrocyte membrane protein 1(PfEMP1) pseudogene	1	1	var	4.073	1.130	ND

chr6	1318330	1319119	3074	PFF1525c	Pfmc-2TM Maurer's cleft two transmembrane protein	1	1	Pfmc-2tm	4.960	1.674	ND
chr6	1329498	1330966	10379	PFF1545w	rifin	1	1	rifin	4.717	1.457	2.265
chr6	1333037	1334038	2071	PFF1550w	stevor	1	1	stevor	5.048	1.461	2.273
chr6	1336149	1337476	2111	PFF1555w	rifin	1	1	rifin	4.970	1.640	2.47
chr6	1338221	1339420	745	PFF1560c	rifin	1	1	rifin	5.430	1.928	ND
chr6	1341310	1342598	1890	PFF1565c	rifin	1	1	rifin	5.319	1.552	2.718
chr6	1347344	1348574	4746	PFF1570w	rifin	1	1	rifin	4.672	1.523	ND
chr6	1350727	1352090	2153	PFF1575w	rifin	1	1	rifin	4.072	1.449	2.211
chr6	1353948	1366431	1858	PFF1580c	erythrocyte membrane protein 1 (PfEMP1)	1	1	var	4.899	1.657	2.686
chr6	1367913	1369105	1482	PFF1585w	rifin pseudogene	1	1	rifin	4.933	1.377	2.527
chr6	1371502	1372862	2397	PFF1590w	rifin	1	1	rifin	4.325	1.201	1.532
chr6	1374798	1382628	1936	PFF1595c	erythrocyte membrane protein 1 (PfEMP1)	1	1	var	4.826	1.463	2.387
chr7	126425	127236		MAL7P1.5	Pfmc-2TM Maurer's cleft two transmembrane protein	1	1	Pfmc-2tm	4.397	1.643	2.02
chr7	131294	132431	4058	PF07_0003	rifin	1	1	rifin	5.194	1.734	2.548
chr7	135266	138319	2835	PF07_0004	Plasmodium exported protein, unknown function	1	1		4.437	1.587	1.552
chr7	167416	167499		MAL7_tRNA_Ty	tRNA Tyrosine	1			1.930	-0.212	ND
chr7	567328	574803	429009	PF07_0048	erythrocyte membrane protein 1 (PfEMP1)	1	1	var	4.915	1.740	2.819
chr7	580490	581698	5687	MAL7P1.43	RIF pseudogene, RIFIN pseudogene	1	1	rifin	4.961	1.703	ND
chr7	582716	590509	1018	PF07_0049	erythrocyte membrane protein 1 (PfEMP1)	1	1	var	4.761	1.696	2.655
chr7	595721	596947	5212	MAL7P1.47	RIF pseudogene, RIFIN pseudogene	1	1	rifin	4.977	1.518	2.656
chr7	598284	605898	1337	MAL7P1.50	erythrocyte membrane protein 1 (PfEMP1)	1	1	var	4.838	1.636	2.99
chr7	607536	614456	1638	PF07_0050	erythrocyte membrane protein 1 (PfEMP1)	1	1	var	4.969	1.783	2.654
chr7	618284	619538	3828	MAL7P1.52	RIF pseudogene, RIFIN pseudogene	1	1	rifin	5.099	1.772	2.737
chr7	622104	629686	2566	PF07_0051	erythrocyte membrane protein 1 (PfEMP1)	1	1	var	4.925	1.620	2.702
chr7	636764	644301	7078	MAL7P1.55	erythrocyte membrane protein 1 (PfEMP1)	1	1	var	5.066	1.747	2.773
chr7	645704	653111	1403	MAL7P1.56	erythrocyte membrane protein 1 (PfEMP1)	1	1	var	4.777	1.697	2.787
chr7	657519	658759	4408	MAL7P1.57	rifin	1	1	rifin	5.071	1.579	2.755
chr7	661031	661818	2272	MAL7P1.58	Pfmc-2TM Maurer's cleft two transmembrane protein	1	1	Pfmc-2tm	4.637	1.538	2.071
chr7	662489	663346	671	MAL7P1.59	hypothetical protein, conserved in P. falciparum	1		hyp	4.478	1.516	1.373
chr7	664741	665900	1395	MAL7P1.61	erythrocyte membrane protein 1 (PfEMP1) pseudogene	1	1	var	3.467	0.917	0.993
chr7	1321286	1336243	655386	MAL7P1.162	dynein heavy chain, putative			dynein	3.624	0.111	0.505
chr7	1374387	1374458	38144	MAL7_tRNA_Th	tRNA Threonine	1			2.535	-0.347	ND
chr7	1434304	1436145	59846	MAL7P1.178	alpha/beta hydrolase protein, putative	1	1	hydrolase	2.821	1.012	0.957
chr7	1437788	1438902	1643	MAL7P1.179	null	1			4.807	1.561	ND
chr7	1441011	1442002	2109	PF07_0130	stevor	1	1	stevor	5.322	1.608	2.603
chr7	1443129	1444413	1127	MAL7P1.183	erythrocyte membrane protein 1 (PfEMP1) pseudogene	1	1	var	5.553	1.655	ND
chr7	1446087	1447419	1674	PF07_0132	rifin	1	1	rifin	5.232	1.577	2.662
chr7	1451813	1453015	4394	MAL7P1.184	rifin	1	1	rifin	5.157	1.679	2.695
chr7	1454828	1456058	1813	PF07_0134	rifin	1	1	rifin	5.002	1.685	2.588
chr7	1457999	1459354	1941	MAL7P1.185	rifin	1	1	rifin	5.011	1.587	2.604

chr7	1469810	1471059	10456	PF07_0138	rifin	1	1	rifin	4.481	1.656	2.276
chr7	1472964	1481610	1905	MAL7P1.187	erythrocyte membrane protein 1 (PfEMP1)	1	1	var	4.793	1.711	2.655
chr8	22369	29661		PF08_0142	erythrocyte membrane protein 1 (PfEMP1)	1	1	var	4.653	1.623	2.657
chr8	30708	40041	1047	PF08_0141	erythrocyte membrane protein 1 (PfEMP1)	1	1	var	4.807	1.415	2.156
chr8	41956	51947	1915	PF08_0140	erythrocyte membrane protein 1 (PfEMP1)	1	1	var	5.072	1.720	2.704
chr8	57476	58827	5529	PF08_0138	rifin	1	1	rifin	5.041	1.616	2.758
chr8	62262	63259	3435	MAL8P1.163	Plasmodium exported protein (PHISTa), unknown function	1	1	phistA	3.022	1.353	ND
chr8	66840	72909	3581	MAL8P1.162	surface-associated interspersed gene 8.3 (SURFIN8.3)	1	1	surfin	3.272	1.333	0.965
chr8	80947	81766	8038	MAL8P1.161	Plasmodium exported protein (hyp7), unknown function	1	1	hyp7	2.157	1.278	0.25
chr8	83961	85028	2195	MAL8P1.160	Plasmodium exported protein (hyp7), unknown function	1	1	hyp7	2.263	1.048	0.727
chr8	103078	103950		PF08_0136b	von willebrand factor a-domain-related protein, putative				1.687	0.250	-0.312
chr8	432287	440173	347259	PF08_0107	erythrocyte membrane protein 1 (PfEMP1)	1	1	var	4.905	1.751	2.92
chr8	441530	449184	1357	PF08_0106	erythrocyte membrane protein 1 (PfEMP1)	1	1	var	4.933	1.679	2.84
chr8	451736	453020	2552	PF08_0105	rifin	1	1	rifin	4.759	1.732	2.725
chr8	454924	456040	1904	PF08_0104	rifin	1	1	rifin	4.447	1.755	2.619
chr8	460433	467688	4393	PF08_0103	erythrocyte membrane protein 1 (PfEMP1)	1	1	var	4.610	1.525	2.389
chr8	1326215	1332258		MAL8P1.1	surface-associated interspersed gene 8.1, (SURFIN8.1)	1	1	surfin	1.617	0.543	0.013
chr8	1407868	1410774	940180	MAL8P1.335	hypothetical protein, conserved in P. falciparum	1		MAL8P1.335	4.223	0.755	ND
chr8	1412780	1413400	2006	MAL8P1.330	hypothetical protein, conserved in P.falciparum	1			4.391	0.312	ND
chr9	4171	4620		PFI0002w	hypothetical proein conserved in P. falciparum	1			3.875	-0.261	ND
chr9	20080	27885	15460	PFI0005w	erythrocyte membrane protein 1 (PfEMP1)	1	1	var	4.701	1.703	2.782
chr9	29797	31157	1912	PFI0010c	rifin	1	1	rifin	3.763	1.622	2.094
chr9	33553	34795	2396	PFI0015c	rifin	1	1	rifin	4.029	1.533	2.292
chr9	39554	40740	4759	PFI0020w	rifin	1	1	rifin	3.859	1.443	ND
chr9	42909	44045	2169	PFI0025c	rifin	1	1	rifin	3.566	1.101	1.542
chr9	45902	47236	1857	PFI0030c	rifin	1	1	rifin	4.698	1.674	2.536
chr9	49612	50971	2376	PFI0035c	rifin	1	1	rifin	5.019	1.524	2.573
chr9	52648	54024	1677	PFI0040c	VARC-like pseudogene	1	1	var	5.178	1.610	ND
chr9	55074	56060	1050	PFI0045c	stevor	1	1	stevor	5.446	1.648	2.67
chr9	58162	59240	2102	PFI0050c	rifin	1	1	rifin	4.938	1.392	2.516
chr9	61342	62553	2102	PFI0055c	rifin	1	1	rifin	5.119	1.792	ND
chr9	64216	64428	1663	PFI0060c	hypothetical protein, pseudogene	1		hyp	4.315	1.604	ND
chr9	67334	68625	2906	PFI0065w	rifin	1	1	rifin	5.265	1.657	2.898
chr9	70521	71738	1896	PFI0070w	rifin	1	1	rifin	5.039	1.667	2.578
chr9	74409	75746	2671	PFI0075w	rifin	1	1	rifin	5.125	1.748	2.606
chr9	77861	78874	2115	PFI0080w	stevor	1	1	stevor	4.068	1.138	1.565
chr9	251351	269707		PFI0260c	dynein heavy chain, putative			dynein	1.884	0.041	0.509
chr9	511488	521520	432614	PFI0550w	cysteine repeat modular protein 1			crmp	2.107	0.083	0.053
chr9	1446917	1447935	925397	PFI1770w	Plasmodium exported protein (PHISTb), unknown function	1	1	phistB	2.252	0.569	0.894
chr9	1458380	1459850	10445	PFI1780w	Plasmodium exported protein (PHISTc), unknown function	1	1	phistC	2.098	0.712	0.474

chr9	1462388	1463647	2538	PFI1785w	Plasmodium exported protein (PHISTb), unknown function	1	1	phistB	4.272	1.583	2.233
chr9	1466102	1467043	2455	PFI1790w	Plasmodium exported protein (PHISTb), unknown function	1	1	phistB	4.349	1.436	2.167
chr9	1468534	1469405	1491	PFI1795c	Plasmodium exported protein, unknown function	1	1		4.376	1.192	1.798
chr9	1471839	1472912	2434	PFI1800w	lysophospholipase, putative	1		lipase	5.020	1.817	2.496
chr9	1474419	1475692	1507	PFI1805w	rifin	1	1	rifin	5.178	1.580	2.694
chr9	1477514	1478724	1822	PFI1810w	rifin	1	1	rifin	4.084	1.112	1.883
chr9	1480935	1482199	2211	PFI1815c	rifin	1	1	rifin	4.484	1.391	2.022
chr9	1486058	1490175	3859	PFI1820w	erythrocyte membrane protein 1 (PfEMP1)	1	1	var	5.531	1.846	2.443
chr9	1495567	1503324	5392	PFI1830c	erythrocyte membrane protein 1 (PfEMP1)	1	1	var	4.598	1.657	2.701
