# Supplementary material for: Plasmodium falciparum Heterochromatin Protein 1 Marks Genomic Loci Linked to Phenotypic Variation of Exported Virulence Factors
Source: PLoS Pathog. 2009 Sep 4;5(9):e1000569. doi: 10.1371/journal.ppat.1000569 (PMC2731224; doi:10.1371/journal.ppat.1000569)
Supplement: Protocol S1 — Generation of transfection constructs. (0.13 MB PDF) [file ppat.1000569.s012.pdf]

Protocol S1 Generation of transfection constructs. The h*dhfr* gene in pHBcam^R [1] was deleted and replaced with a *BamH*I-*Not*I-*Avr*II multi-cloning site (pBcam-MCS). A triple HA-tag was amplified by PCR from HA-pENTR1/2 [2] (HAtagF: atc**ggatcc**aag**gcggccgc**attgctcgagcta**ccatgg**tat**gctagc***gtggtgcttaggtacccgtac*; HAtagR: gatc**tctaga**tga**gtcgac***ataaagttgtta**gagctc**ggcataatc*), digested with BamHI/XbaI and ligated into BamHI/AvrII digested pBcam-MCS to obtain the parental tagging vector pBcam-3xHA. pBcamHP1-3xHA was generated by cloning the full-length PFL1005c sequence amplified from 3D7 gDNA using primers HP1-BamHIF (gatc**ggatcc**aatgacagggtcagatgaag) and HP1-NcoIR (gatc**ccatgg**ataagctgtacggtatcttag) into BamHI/NcoI digested pBcam-3xHA. To produce the PfHP1-GFP construct, full-length PFL1005c was amplified from 3D7 gDNA using primers HP1-GFP F (tat**ctgcag**atgacagggtcagatgaag) and HP1-GFP-R (tat**acgcgt**agctgtacggtatcttagtc) and cloned into BglII/MluI-digested pTGFP [3]. To obtain pHcamHP1-2xTy the *bsd* gene in pBcamHP1-3xHA was excised with *Xho*I and replaced with the h*dhfr* gene amplified from pHBcam^R. The 3xHA tag was removed by digestion with NcoI/SalI and replaced by insertion of annealed complementary oligonucleotides coding for a double Ty-tag.

1. Voss TS, Healer J, Marty AJ, Duffy MF, Thompson JK, et al. (2006) A var gene promoter controls allelic exclusion of virulence genes in Plasmodium falciparum malaria. Nature 439: 1004-1008.

2. Mullin KA, Lim L, Ralph SA, Spurck TP, Handman E, et al. (2006) Membrane transporters in the relict plastid of malaria parasites. Proc Natl Acad Sci U S A 103: 9572-9577

3. Gilson PR, O'Donnell RA, Nebl T, Sanders PR, Wickham ME, et al. (2008) MSP1(19) miniproteins can serve as targets for invasion inhibitory antibodies in Plasmodium falciparum provided they contain the correct domains for cell surface trafficking. Mol Microbiol 68: 124-138.
