# Supplementary material for: Plasmodium falciparum Heterochromatin Protein 1 Marks Genomic Loci Linked to Phenotypic Variation of Exported Virulence Factors
Source: PLoS Pathog. 2009 Sep 4;5(9):e1000569. doi: 10.1371/journal.ppat.1000569 (PMC2731224; doi:10.1371/journal.ppat.1000569)
Supplement: Protocol S2 — Nuclear fractionation. (0.09 MB PDF) [file ppat.1000569.s013.pdf]

Protocol S2 Nuclear fractionation. 2x10E9 Parasites were released from RBCs by saponin lysis and washed three times in PBS. Parasites were lysed in CLB (20mM HEPES (pH7.9), 10mM KCl, 1mM EDTA, 1mM EGTA, 0.65% NP-40, 1mM DTT, protease inhibitors (Complete TM, Roche Diagnostics)) for 5 min on ice. Nuclei were pelleted at 3000rpm and the cytoplasmic fraction saved. After three washes in CLB four equal aliquots were digested with either 20U DNAseI (Roche Diagnostics), 300U MNAse (Fermentas), RNAseA (Sigma-Aldrich), or a no-enzyme control by incubation of the pellet in digestion buffer DB (20mM Tris-HCl, pH7.5, 15mM NaCl, 60mM KCl, 1mM $CaCl_2$, 5mM $MgCl_2$, 5mM $MnCl_2$, 300mM sucrose, 0.4% NP-40, 1mM DTT, protease inhibitors) for 20 min at 37°C. Soluble fractions were recovered by centrifugation at 13'000 rpm. Nuclear debris was washed twice in DB followed by high salt extraction using HSB (20mM HEPES (pH7.9), 1M KCl, 1mM EDTA, 1mM EGTA, 1mM DTT, protease inhibitors) by vortexing for 20 min at 4°C. After centrifugation for 3 min at 13'000 rpm the high salt nuclear fraction was saved and the pellet washed twice in HSB. The insoluble material was solubilised in SDS extraction buffer (2%SDS, 10mM Tris-HCl (pH 7.5)) by vortexing for 20 min at room temperature. Nucleic acids from each sample were isolated by adjustment to 2%SDS followed by phenol:chloroform extraction and ethanol precipitation.
